# Supplementary material for: Inferring joint sequence-structural determinants of protein functional specificity
Source: eLife. 2018 Jan 16;7:e29880. doi: 10.7554/eLife.29880 (PMC5770160; doi:10.7554/eLife.29880)
Supplement: Figure 1—source data 1. [file elife-29880-fig1-data1.docx]

**Figure 1—Source data 1. Contrast alignments for GNAT superfamily and Gna1 family.**

This and other BPPS-generated contrast alignment files consist of several versions of the same aligned set of representative sequences from distinct phyla; these sequences are closely related to the first sequence, which corresponds to the protein of interest. Each version of the alignment is highlighted differently to reveal distinct sequence features. The first alignment highlights every residue position conserved among these sequences, the phylum for each of which is indicated on the far left, with phylum names colored red, tan, cyan, green, purple and blue to indicate metazoans, fungi, protozoans, plants, eubacteria and archaea, respectively. Below this are shown two or more copies of the same alignment; these correspond to subgroups along the lineage leading from the root to the protein of interest within the BPPS hierarchy. For these, the sequence identifiers in the leftmost column are colored yellow, red, orange, and green to indicate the superfamily, family, subfamily and sub-subfamily subgroups, respectively, along the lineage. The SIPRIS/BPPS-generated PyMol scripts also use this color scheme to classify pattern residues. Within each of these aligments, the residues most distinctive of the corresponding subgroup are highlighted to indicate amino acid biochemical properties based on the following color code: red font with yellow highlight, non-polar (AVILMWFY) ; blue font with yellow highlight, cysteine (C); red, acidic (DE); cyan, basic (KR); magenta, polar (STNQ); green, glycine (G); blue, histidine (H); black, proline (P). Non-distinguishing positions are shown in gray font. The red bars above highlighted columns quantify the selective pressures imposed on pattern residue positions using a semi-logarithmic scale. Directly below the aligned sequences, the characteristic foreground residues at each position are shown and, directly below these, corresponding frequencies are given in integer tenths. A ‘7’, for example, indicates that the corresponding residue occurs in 70-80% of the foreground sequences. Below the alignments are shown the background residues and frequencies (using the same format as for the foreground). Note that the superfamily’s background corresponds to standard amino acid frequencies and thus is not shown. Foreground and background sets are defined in **Fig. 7**.

**Nematoda**  22 **FKVRPLAKDDFSKGYVDLLSQLTSVGNLDQEAFEKRFEAMRTSvPNYHIVVIEDsnSQK.VVASASLVVEMKFIHGAGSRGRVEDVVVDTEMRRQKLGAVLLKTLVSLGKSLGVYKISLECVPELLPFYSQFGF.QDDCN.F------MTQRF** 165*

**Streptophyta**  7 **--IRKLEISDKRKGFIELLGQLTVTGSVTDEEFDRRFEEIRSYgDDHVICVIEEetSGK.IAATGSVMIEKKFLRNCGKAGHIEDVVVDSRFRGKQLGKKVVEFLMDHCKSMGCYKVILDCSVENKVFYEKCGM.SNKSI.Q------MSKYF** 148

**Ascomycota**  50 **--IRPLCRSDYKRGYLDVLRVLTTVGDINEEQWNSRYEWIRARsDEYYLLVVCDg.EGR.IVGTGSLVVERKFIHSLGMVGHIEDIAVEKGQQGKKLGLRIIQALDYVAEKVGCYKTILDCSEANEGFYIKCGF.KRAGL.E------MAHYY** 190

**Chordata**  39 **LVLRPLCTADLNRGFFKVLGQLTETGVVSPEQFMKSFEHMKKS.GDYYVTVVEDvtLGQ.IVATATLIIEHKFIHSCAKRGRVEDVVVSDECRGKQLGKLLLSTLTLLSKKLNCYKITLECLPQNVGFYKKFGY.TVSEEnY------MCRRF** 182

**Amoebozoa**  9 **ISFRPLDIDDFDKGYSECLQQLT-EAKFTKEQFIERFNQIKKQsDTYFLIVAVDvkLNK.IIACGSLFVEKKFIRNCGKCGHIEDIVVNNNYRGKNLGLRIIEQLKCIGSQAGCYKIILDCSEANVKFYEKCKF.ERKGV.Q------MSIYL** 151

**Annelida**  36 **LVYRPLSLGDFDKGFVNLLSQLTKVGEVSREQFEKRFSSMKSSnGTYYVTVIEDieKGE.IIGAATLVVEQKFIHKCACRGRIEDVIVSNVYRGKQLGKLLVEALTLLGKALGCYKMSLECKEENFKFYESFGY.SPDGQhF------MIQRF** 180

**Placozoa**  1 **LVVRPLQRDDFDKGYLQLLSNLTEVGDVTKEMFYKRFDHMKSWqNSHFVTVIEDtsTGK.IIGNTTLVIEQKFIHCATYRGRIEDVIVDDAYRGKQLAKILVGSMVLLSEKVDCYKLTLECTEDYMPFYQKFGLaTNQSR.Y------MQKKF** 145

**Porifera**  52 **LVLRSLHKNDYDKGYMTLLGQLTRTGDVTKERFEAQFDAMKQCpGIHYIMVIEDv.SNAiIVGSGTLVVERKFTHNTALRGRVEDIVVHSNYRGRHLGNLIVETATVLSQKLGCYKTSLDCLPSLKPFYEKFEF.ENTSViF------MSKRF** 196

**Arthropoda**  51 **LKVRPLQRSDYDKGFLQLLGQLTSVGNVSKKQFDERFTQMKQS.GGYYVTVIEDtrVGR.LIGAATLTVEQKFIHNCSLRGRLEDVVVNDTYRGKQLGKLIVVTVSLLAQEVGCYKMSLDCKDKLIKFYETLGY.KMEAG.N---SNAMNMRF** 196

**position**  . 30 . 40 . 50 . 60 . 70 . 80 . 90 . 100 . 110 . 120 . 130 . 140 . 150 . 160 .

**_**

**_**

**_ _ _**

**_ _ _**

**_ _ _**

**_ _ _**

**_ _ _**

**_ _ _**

**_ _ _**

**_ _ _**

**_ _ _**

**_ _ _**

**_ _ _**

**_ _ _**

**_ _ _**

**_ _ _**

**_ _ _ __**

**_ _ _ __**

**_ _ _ __**

**_ _ _ __**

**_ _ _ __**

**_ _ _ __**

**_ _ _ __**

**_ _ _ __**

**_ _ _ __**

**_ _ _ __**

**_ _ _ _ __**

**_ _ _ _ _ __**

**_ _ _ _ _ __**

**_ _ _ _ _ __**

**_ _ _ _ _ _ _ _ __**

**_ _ _ _ _ _ _ _ _ __**

**_ _ __ _ _ _ _ _ _ __**

**_ _ __ _ _ _ _ _ _ _ __**

**_ _ __ _ _ _ _ ___ _ __**

**_ _ __ _ _ _ _ ___ _ __**

**_ _ _ __ _ _ _ _ ___ _ __**

**_ _ _ __ _ _ _ _ ___ _ __**

**_ _ _ __ _ _ _ _ ___ _ _ _ __**

**_ __ _ __ _ _ _ _ ___ _ _ _ __**

**_ __ _ __ _ _ _ _ ___ _ _ __ __**

**_ __ _ __ _ _ _ _ ___ _ _ __ __**

**_ __ _ _ __ _ _ _ _ ___ _ _ _ __ __**

**_ __ _ _ _ __ _ _ _ _ ___ __ _ __ __ __**

**_ __ _ _ _ _ __ _ _ _ _ ___ __ _ _ __ __ __**

**_ __ _ _ _ _ __ _ _ _ _ ___ __ _ _ __ __ __**

**_ __ _ _ _ _ __ _ _ _ _ ___ __ _ _ __ __ __**

**_ __ _ _ _ _ __ _ _ _ _ ___ __ _ _ __ __ __**

**_ __ _ _ _ _ __ _ _ _ _ ___ __ _ _ __ __ __**

**_ __ _ _ _ _ __ _ _ _ _ ___ __ _ _ _ _ __ __ __**

**_ __ _ _ _ _ __ _ _ _ _ ___ __ _ _ _ _ __ __ __**

**GNAT** ● ●● ● ● ● ● ●● ● ● ● ● ●●● ●● ● ● ● ● ●● ●● ●●

**4ag7A_Gna1_cae**  22 **FKVRPLAKDDFSKGYVDLLSQLTSVGNLDQEAFEKRFEAMRTSvPNYHIVVIEDsnSQK.VVASASLVVEMKFIHGAGSRGRVEDVVVDTEMRRQKLGAVLLKTLVSLGKSLGVYKISLECVPELLPFYSQFGF.QDDCN.F------MTQRF** 165*

**3t90A_arath**  7 **--IRKLEISDKRKGFIELLGQLTVTGSVTDEEFDRRFEEIRSYgDDHVICVIEEetSGK.IAATGSVMIEKKFLRNCGKAGHIEDVVVDSRFRGKQLGKKVVEFLMDHCKSMGCYKVILDCSVENKVFYEKCGM.SNKSI.Q------MSKYF** 148

**2vxkA_Gna1_asp**  50 **--IRPLCRSDYKRGYLDVLRVLTTVGDINEEQWNSRYEWIRARsDEYYLLVVCDg.EGR.IVGTGSLVVERKFIHSLGMVGHIEDIAVEKGQQGKKLGLRIIQALDYVAEKVGCYKTILDCSEANEGFYIKCGF.KRAGL.E------MAHYY** 190

**2huzA_human**  39 **LVLRPLCTADLNRGFFKVLGQLTETGVVSPEQFMKSFEHMKKS.GDYYVTVVEDvtLGQ.IVATATLIIEHKFIHSCAKRGRVEDVVVSDECRGKQLGKLLLSTLTLLSKKLNCYKITLECLPQNVGFYKKFGY.TVSEEnY------MCRRF** 182

**GNA1_DICDI**  9 **ISFRPLDIDDFDKGYSECLQQLT-EAKFTKEQFIERFNQIKKQsDTYFLIVAVDvkLNK.IIACGSLFVEKKFIRNCGKCGHIEDIVVNNNYRGKNLGLRIIEQLKCIGSQAGCYKIILDCSEANVKFYEKCKF.ERKGV.Q------MSIYL** 151

**ELU10627.1**  36 **LVYRPLSLGDFDKGFVNLLSQLTKVGEVSREQFEKRFSSMKSSnGTYYVTVIEDieKGE.IIGAATLVVEQKFIHKCACRGRIEDVIVSNVYRGKQLGKLLVEALTLLGKALGCYKMSLECKEENFKFYESFGY.SPDGQhF------MIQRF** 180

**XP_002118004.1**  1 **LVVRPLQRDDFDKGYLQLLSNLTEVGDVTKEMFYKRFDHMKSWqNSHFVTVIEDtsTGK.IIGNTTLVIEQKFIHCATYRGRIEDVIVDDAYRGKQLAKILVGSMVLLSEKVDCYKLTLECTEDYMPFYQKFGLaTNQSR.Y------MQKKF** 145

**XP_003387706.1**  52 **LVLRSLHKNDYDKGYMTLLGQLTRTGDVTKERFEAQFDAMKQCpGIHYIMVIEDv.SNAiIVGSGTLVVERKFTHNTALRGRVEDIVVHSNYRGRHLGNLIVETATVLSQKLGCYKTSLDCLPSLKPFYEKFEF.ENTSViF------MSKRF** 196

**XP_011568872.1**  51 **LKVRPLQRSDYDKGFLQLLGQLTSVGNVSKKQFDERFTQMKQS.GGYYVTVIEDtrVGR.LIGAATLTVEQKFIHNCSLRGRLEDVVVNDTYRGKQLGKLIVVTVSLLAQEVGCYKMSLDCKDKLIKFYETLGY.KMEAG.N---SNAMNMRF** 196

**foreground (232892):**  **VTVRRATAADAAAGVAAILAAAFSAGNWSEADFAARIAAIAAA GSHLFWAAED GGE VAGTAGVFVEQKFIRGCGGEGEVGDYAVAEAAQGQGYGSAAVAAAEAYAKARGAKRVEAEADADNAAFYESLGY TEEGT LGEDAV MGK L**

**MRL PLRPE LPE LLRL REP VVPEPPPEELRRYLREMRKS DDY LLLL E EDR PLAFLRLLI H LHNLSR LYL RLYLLPEFRRR LARRLMRELLR LFEL LR LVL V PE SRL RR F VPV R R P L L**

**LEI ED IE I E DV ET DLDRD IEE E LKS T VFII D K LI YISII R SGAD A I I IH DY K I KK LE VIE R I I T E KV KK R E R**

**wt_res_freqs (73991): 1216122117112811211111111611211111111111121 4111111422 142 21812112391987324511131211251111251816121112211141115112211111116413531381 11131 111111 111 2**

**313 21121 211 3113 111 15111112121113115111 227 1211 1 111 111111314 1 152211 113 12111411412 22115111221 1122 11 214 3 11 223 12 6 111 1 1 1 1 1**

**214 11 11 2 1 11 11 31111 111 1 132 2 1211 3 1 22 11112 3 1121 2 3 1 11 12 2 2 11 31 111 2 1 2 2 1 11 13 1 1 1**

**insertions 8**

**deletions 6422222221111911111111199991119998887649999 9994432111 99999999999 111212 112334454222213332 5831111122 34666 799998754433**

**position**  . 30 . 40 . 50 . 60 . 70 . 80 . 90 . 100 . 110 . 120 . 130 . 140 . 150 . 160 .

**_**

**_**

**_**

**_**

**_**

**_**

**_**

**_**

**_**

**_**

**_**

**_**

**_**

**_**

**_**

**_**

**_**

**_**

**_**

**_**

**_**

**_ _**

**_ _**

**_ _ _**

**_ _ _**

**_ _ _**

**_ _ _**

**_ _ _**

**_ _ _**

**_ _ _**

**_ _ _ _**

**_ _ _ _**

**_ _ __ _ _ _**

**_ _ _ __ _ _ _ _**

**_ _ _ __ _ _ _ _**

**_ _ _ __ _ _ _ _**

**_ _ _ __ _ _ __ _**

**__ _ _ __ _ _ _ __ _**

**__ _ _ __ _ _ __ __ _**

**__ _ _ __ _ _ __ __ _**

**__ _ _ __ _ _ __ ___ _**

**_ __ _ _ __ _ _ __ ___ _**

**_ _ __ _ _ _ __ _ __ __ ___ _**

**__ _ __ _ _ _ ____ _ __ __ _ ___ _**

**__ _ __ _ _ _ __ ____ _ __ __ _ ___ _**

**_ __ _ __ _ _ _ _ _ __ ____ _ __ __ _ _ ___ _**

**_ __ _ __ _ _ _ _ _ __ ____ _ __ __ _ _ ___ _**

**_ __ _ __ _ _ _ _ _ __ ____ _ __ __ _ _ ___ _**

**_ __ _ __ _ _ _ _ _ __ ____ _ __ __ _ _ ___ _**

**_ __ _ __ _ _ _ _ _ __ ____ _ __ __ _ _ ___ _**

**_ __ _ __ _ _ _ _ _ __ ____ _ __ __ _ _ ___ _**

**Gna1 family**  ● ●● ● ●● ● ● ● ● ● ●● ●●●● ● ●● ●● ● ● ●●● ●

**4ag7A_Gna1_cae**  22 **FKVRPLAKDDFSKGYVDLLSQLTSVGNLDQEAFEKRFEAMRTSvPNYHIVVIEDsnSQK.VVASASLVVEMKFIHGAGSRGRVEDVVVDTEMRRQKLGAVLLKTLVSLGKSLGVYKISLECVPELLPFYSQFGF.QDDCN.F------MTQRF** 165*

**3t90A_arath**  7 **--IRKLEISDKRKGFIELLGQLTVTGSVTDEEFDRRFEEIRSYgDDHVICVIEEetSGK.IAATGSVMIEKKFLRNCGKAGHIEDVVVDSRFRGKQLGKKVVEFLMDHCKSMGCYKVILDCSVENKVFYEKCGM.SNKSI.Q------MSKYF** 148

**2vxkA_Gna1_asp**  50 **--IRPLCRSDYKRGYLDVLRVLTTVGDINEEQWNSRYEWIRARsDEYYLLVVCDg.EGR.IVGTGSLVVERKFIHSLGMVGHIEDIAVEKGQQGKKLGLRIIQALDYVAEKVGCYKTILDCSEANEGFYIKCGF.KRAGL.E------MAHYY** 190

**2huzA_human**  39 **LVLRPLCTADLNRGFFKVLGQLTETGVVSPEQFMKSFEHMKKS.GDYYVTVVEDvtLGQ.IVATATLIIEHKFIHSCAKRGRVEDVVVSDECRGKQLGKLLLSTLTLLSKKLNCYKITLECLPQNVGFYKKFGY.TVSEEnY------MCRRF** 182

**GNA1_DICDI**  9 **ISFRPLDIDDFDKGYSECLQQLT-EAKFTKEQFIERFNQIKKQsDTYFLIVAVDvkLNK.IIACGSLFVEKKFIRNCGKCGHIEDIVVNNNYRGKNLGLRIIEQLKCIGSQAGCYKIILDCSEANVKFYEKCKF.ERKGV.Q------MSIYL** 151

**ELU10627.1**  36 **LVYRPLSLGDFDKGFVNLLSQLTKVGEVSREQFEKRFSSMKSSnGTYYVTVIEDieKGE.IIGAATLVVEQKFIHKCACRGRIEDVIVSNVYRGKQLGKLLVEALTLLGKALGCYKMSLECKEENFKFYESFGY.SPDGQhF------MIQRF** 180

**XP_002118004.1**  1 **LVVRPLQRDDFDKGYLQLLSNLTEVGDVTKEMFYKRFDHMKSWqNSHFVTVIEDtsTGK.IIGNTTLVIEQKFIHCATYRGRIEDVIVDDAYRGKQLAKILVGSMVLLSEKVDCYKLTLECTEDYMPFYQKFGLaTNQSR.Y------MQKKF** 145

**XP_003387706.1**  52 **LVLRSLHKNDYDKGYMTLLGQLTRTGDVTKERFEAQFDAMKQCpGIHYIMVIEDv.SNAiIVGSGTLVVERKFTHNTALRGRVEDIVVHSNYRGRHLGNLIVETATVLSQKLGCYKTSLDCLPSLKPFYEKFEF.ENTSViF------MSKRF** 196

**XP_011568872.1**  51 **LKVRPLQRSDYDKGFLQLLGQLTSVGNVSKKQFDERFTQMKQS.GGYYVTVIEDtrVGR.LIGAATLTVEQKFIHNCSLRGRLEDVVVNDTYRGKQLGKLIVVTVSLLAQEVGCYKMSLDCKDKLIKFYETLGY.KMEAG.N---SNAMNMRF** 196

**foreground (1243):**  **IKVRALCSADYNKGHIDCLGQLTSAGNVSEAAWNAQYDEIAAA GSHYVTVVED SGQ VVGAGAVFVEQKFIRGCGSRGRVEDVAVASSQQGKQLGKKVVEAVTDVGESVGCYKSSLNCSDANEGFYEKCGY TKAGG NAARANAMAQRY**

**LIL P RID FER FLQL RV SVVPEITREQFLKRFEWMRKS DDYFIL IV TDR IIATATLLI H LHNLSLV HL IV DKEYR RK LLLIQTLVLLSRKLN II E KPKLIP V L F VLKPL YSIYLPS VIYF**

**VI QRT D Y EV S ET D KDE EE LKS T I NK S S I R SGAK I NDD G RI DYIAKE VT D EE VK F KREEV Q H S H**

**wt_res_freqs (366): 1129191119513811219149811613321111112211121 4115319267 241 36515122391987324513932995381112397389411313121112127899129194316129947593 12231 141216283131**

**712 6 211 113 5614 22 115113314371167115111 227132 61 413 624535614 1 1522125 61 46 33125 13 333422711321231 15 1 212223 1 1 5 11211 1215112 1144**

**23 111 2 2 22 2 11 3 111 12 132 2 2 13 1 2 2 3 1122 5 112 1 34 112631 21 6 21 21 1 32121 2 1 4 1**

**insertions 1 5 2 2 2 1 6 219 1**

**deletions 6621111111111111119987788888777665443222223 22211 1 11 111122334556566521168111111 11111 199988811198**

**background (231649):**  **VTVRRLTAADAAA VAAILAAAF WSEADFAARIAA LFWAAED GGE VAGTAAV GEGEVGDYAVAEAAQGQGYGSAAVAAAEAEAKARGAKRVEAEADADNAAFYESLGY TEEGT LGEDAV MGK L**

**MRL PARPE LPE LLRL REP PPPEELRRYLRE LLLL E EDR PLAFLSL R LYL RLYLLPEFRRR LARRLMRELLRYLFEL LR LVL V PE SRL RR F VPV R R P L L**

**LEI ED IE I E DV LDRD IEE E VFII D K LI YIRI D A I I IH DY K I KK LE VIE R I I T E KV KK R E R**

**wt_res_freqs (73625): 1216112117212 112111111 121111111111 1111422 142 2181211 11131211251111251816121112211141115112211111116413531381 11131 111111 111 2**

**413 22121 211 3113 111 111212111311 1211 1 111 1111113 1 113 12111411412 2211511122111122 11 214 3 11 223 12 6 111 1 1 1 1 1**

**214 11 11 2 1 11 1111 111 1 1211 3 1 22 1111 1 2 3 1 11 12 2 2 11 31 111 2 1 2 2 1 11 13 1 1 1**

**position**  . 30 . 40 . 50 . 60 . 70 . 80 . 90 . 100 . 110 . 120 . 130 . 140 . 150 . 160 .
