# Supplementary material for: Inferring joint sequence-structural determinants of protein functional specificity
Source: eLife. 2018 Jan 16;7:e29880. doi: 10.7554/eLife.29880 (PMC5770160; doi:10.7554/eLife.29880)
Supplement: Figure 2—source data 1. [file elife-29880-fig2-data1.docx]

**Figure 2— Source data 1. P-loop GTPase superfamily: R^4^ family, Rab subfamily and Rab8 sub-subfamily.**

**Chordata**  7 **YLFKLLLIGDSGVGKTCVLFRFSEDAFNSTFISTIGIDFKIRTIELDGKRIKLQIWDTAGQERFRTITTAYYRGAMGIMLVYDITNEKSFDNIRNWIRNIEEHASADVEKMILGNKCDV.N.D.KRQVSKERGEKLALDYGIKFMETSAKANINVENAFFTLARDI** 169*

**Chordata** 17 **YLFKLLLIGDSGVGKSCLLLRFADDTYTESYISTIGVDFKIRTIELDGKTIKLQIWDTAGQERFRTITSSYYRGAHGIIVVYDVTDQESFNNVKQWLQEIDRYASENVNKLLVGNKCDL.T.T.KKVVDYTTAKEFADSLGIPFLETSAKNATNVEQSFMTMAAEK** 179

**Ascomycota** 20 **-IMKILLIGDSGVGKSCLLVRFVEDKFNPSFITTIGIDFKIKTVDINGKKVKLQLWDTAGQERFRTITTAYYRGAMGIILVYDVTDERTFTNIKQWFKTVNEHANDEAQLLLVGNKSDM.E.T.-RVVTADQGEALAKELGIPFIESSAKNDDNVNEIFFTLAKLI** 180

**Mollusca**  7 **YLFKLLLIGDSGVGKTCLLFRFSEDAFNSTFISTIGIDFKIRTIELDGKKIKLQIWDTAGQERFRTITTAYYRGAMGIMLVYDITSEKSFENIRNWIRNIEEHASKDVEKMILANKCDM.N.D.RRQVSKERGENLAVEHGIKFMETSAKASINVDEAFFTLARD-** 168

**Stramenopiles**  10 **-LIKLLLIGDSGVGKSCVLLRYSDDSFTTSFITTIGIDFKVKTIDVDGKRIKLQIWDTAGQERFRTITTAYYRGAMGILLVYDVTDDHSFQNIRNWMTQIRQNASSNVNKILIGNKCDV.DpS.ERAVTTKQGQDLADEFGIKFFETSAKSNHNIDEAFRSIAVD-** 171

**Ascomycota**  9 **FLIKLLLIGDSGVGKSCCLLRFSEDSFTPSFITTIGIDFKIRTIDLDGKRIKLQIWDTAGQERFRTITTAYYRGAMGILLVYDVTDERSFNNIQTWFQNVEQHATEGVNKILIGNKCDW.E.E.KRVVSTERGQALANELGIPFMEVSAKANINVEEAFLLLARD-** 170

**Amoebozoa**  14 **YLIKLLLIGDSGVGKSCLLLRFSEDSFTPSFITTIGIDFKIRTIELEGKRIKLQIWDTAGQERFRTITTAYYRGAMGILLVYDVTDEKSFGNIRNWIRNIEQHATDSVNKMLIGNKCDM.A.E.KKVVDSSRGKSLADEYGIKFLETSAKNSINVEEAFISLAKD-** 175

**Echinodermata**  7 **FLFKLLLIGDSGVGKTCVLFRFSEDAFNSTFISTIGIDFKIRTIELDGKKIKLQIWDTAGQERFRTITTAYYRGAMGIMLVYDITNQKSFDNIRNWIRNIEEHASADVEKMILGNKCDM.D.D.RRAVSKEKGEQLAIEYGIKFMETSAKASINVEEAFVTLARD-** 168

**Placozoa**  7 **YLFKLLLIGDSGVGKTCVLFRFSEDAFNSTFISTIGIDFKIRTIELDGKKIKLQIWDTAGQERFRTITTAYYRGAMGIMLVFDITNERSFENIKTWIRNIEEHAASDVEKMILGNKCDV.V.D.KRQVSKEQAQQLAAEYGVKFSETSAKDGHNVEEAFMTLAKD-** 168

**Cnidaria**  7 **YLFKLLLIGDSGVGKTCVLFRFSEDAFNSTFISTIGIDFKIRTIDLDGKKIKLQIWDTAGQERFRTITTAYYRGAMGIMLVYDITNDKSFENIKNWIRNIEEHAAADVEKMILGNKCDM.N.D.KRQVSAERGQALAMDHNVKFMETSAKTSANVEEAFISLARD-** 168

**Heterolobosea**  16 **YLCKLLLIGDSGVGKSCLLLRFSDDTFTTNFITTIGIDFKIRTIELDGKRVKLQIWDTAGQERFRTITTAYYRGAMGIMLTYDVTDEQSFLNIRNWMKNIEEHAADNVNKMLIGNKCDL.I.E.KKIVETERGQSLAKSYGIPFMETSAKNNINVEEAFFTIARE-** 177

**Mortierellomyc**  11 **YLIKLLLIGDSGVGKSCLLLRFSDDSFTPSFITTIGIDFKIRTIELDGKRIKLQIWDTAGQERFRTITTAYYRGAMGILLVYDVTDERSFSNIRNWFSNVEQHASEGVNKILIGNKCDM.L.D.KKVVQKDQGQTLADEFGIKFLETSAKSNICVEEAFFSLARD-** 172

**Eustigmatophyc**  15 **HLIKLLLIGDSGVGKSCLLLRYSEDSFTPSFITTIGIDFKIKSLPLEDKKLKLQIWDTAGQERFRTITTAYYRGAMGILLVYDVTDERSFANVKNWMRQIEQHASESVNKILIGNKCDV.DpA.DRRVSLEQGRKLAEEYRIKFFETSAKENVNVDEAFYTVARD-** 177

**Opisthokonta**  7 **YLFKLLLIGDSGVGKTCVLFRFSDDAFNATFISTIGIDFKIRTIELDGKKIKLQIWDTAGQERFRTITTAYYRGAMGIMLVYDVTQDKTFENIKNWIRNIEQHASEDVEKMILGNKCDV.D.D.KRVVTKERGEQLAREYNVRFFETSAKANINVEEAFLTIARD-** 168

**Chlorophyta**  20 **HLLKLLLIGDSGVGKSCLLLRFSDDQFTTSFITTIGIDFKIKTVELGGKRVKLQIWDTAGQERFRTITTAYYRGAMGILLVYDVSDERSFENVRGWMRNIEQHASSNVNKILIGNKCDVkE.D.KRVISKARGQALADEFGIPFFETSAKSSVNVNDAFMTIAGD-** 182

**Cryptophyta**  13 **YLVKLLLIGDSGVGKSCLLLRFSDDSFTTSFITTIGIDFKIRTIEQEGKRLKLQIWDTAGQERFRTITTAYYRGAMGILLVYDVTDEQSFNNIRNWMRQIQQHASDNVNKILIGNKCDM.L.D.KKVIETARGQALADEFGIKFFETSAKNNINVEKAFTEIARDV** 175

**Porifera**  25 **FLFKLLLIGDSGVGKTCILFRFSEDAFNSTFISTIGIDFKIRTIELDGKKIKLQIWDTAGQERFRTITTAYYRGAMGIMLVYDITNDKSFDNIKNWIRNIEEHASADVERMILGNKCDM.D.E.KRAVSKERGEQLAVEYGVKFMETSAKASINVEEAFITLAKD-** 186

**Annelida**  6 **YLFKLLLIGDSGVGKTCVLFRFSEDAFNSTFISTIGIDFKIRTIELDGKKIKLQIWDTAGQERFRTITTAYYRGAMGIMLVYDITCEKSFENIRNWIRNIEEHASSDVEKMILGNKCDM.N.E.KRQVSKQRGEALAIEYGVKFLETSAKASINVEEGFFTLARD-** 167

**Bryozoa**  11 **HLFKLLLIGDSGVGKTCVLFRFSEDAFNATFISTIGIDFKIRTIELDGKKIKLQIWDTAGQERFRTITTAYYRGAMGIMLVYDITNERSFDNIKNWIRNIEEHASSDVEKMVLGNKCDM.N.D.RRVVSRDRGEQLAVEYGIKFMETSAKNADNVDEAFYSLARD-** 172

**Breviatea**  15 **YLIKLLLIGDSGVGKSCLLLRFSEDQFTPSFITTIGIDFKIRSIELDGKRIKLQIWDTAGQERFRTITTAYYRGAMGILLVYDVTEEKTFLNINNWIRNIEQHASENVSMILVGNKCDM.V.D.RKVVDPERAQQLANEYGIKFLETSAKSNHNVDEAFISLTRD-** 176

**Xenacoelomorph**  10 **-LYKLLLIGDSGVGKTSLLFRFAEDAFNATFISTIGIDFKIRTIDIDGKKIKLQVWDTAGQERFRTITTAYYRGAMGIILVYDVTCQTSFDNIRNWIKNTDEHASNDVEKMILGNKCDI.E.G.KRVITYEKGERLAQEYNVKFYETSARESVNVETAFIEFAR--** 169

**position**  10 . 20 . 30 . 40 . 50 . 60 . 70 . 80 . 90 . 100 . 110 . 120 . 130 . 140 . 150 . 160 .

**_**

**_**

**__**

**__**

**__**

**__**

**__**

**___ _**

**___ _ _**

**___ _ _ _**

**___ _ _ _**

**___ _ _ _**

**_ ___ _ _ _**

**_ ___ _ _ _**

**_ ___ _ _ _**

**_ ___ _ _ _**

**_ ___ _ _ _ _**

**_ ___ _ _ _ _**

**_ ___ _ _ __ _**

**_ ___ _ _ __ _**

**_ ___ _ _ __ _**

**_ ___ _ _ __ _**

**_ ___ _ _ __ _**

**_ ___ _ _ __ _**

**_ ___ _ _ __ _**

**_ ____ _ _ __ _**

**_ ____ _ _ __ _**

**_ ____ _ _ __ _**

**_ ____ _ _ __ _**

**_ ____ _ _ __ _**

**_ ____ _ _ __ _**

**_ ____ _ _ __ _**

**_ ____ _ _ __ _**

**_ _ ____ _ _ _ __ _**

**_ _ ____ _ _ _ _ __ _**

**_ _ ____ _ _ _ _ _ __ _**

**_ _ _ _ ____ _ _ _ _ _ __ _**

**_ _ _ _ ____ _ _ _ __ _ _ __ _ _**

**_ _ _ _ ____ _ _ _ __ _ _ __ _ _**

**_ ___ _ ____ _ _ _ __ _ _ __ _ _**

**_ ___ _ ____ _ _ _ __ _ __ __ _ _**

**_ ___ _ ____ _ _ _ _ __ _ __ __ _ _**

**_ ___ _ _____ _ _ _ _ __ _ __ __ _ _**

**_ ___ _ _____ _ _ _ _ __ _ __ __ _ _**

**_ ___ _ _____ _ _ _ _ __ _ __ _ __ _ _**

**_ ___ _ _____ _ _ _ _ __ _ __ _ __ _ _**

**_ ___ _ _____ _ _ _ _ __ _ __ ___ __ _ _**

**_ ___ _ _____ _ _ _ _ __ _ __ ___ __ _ _**

**_ ___ _ _____ _ _ _ _ __ _ __ ___ __ _ _**

**_ ___ _ _____ _ _ _ ____ __ ___ ___ __ _ __**

**_ ___ _ _____ _ _ _ ____ __ ___ ___ __ _ __**

**P-loop GTPases**  ● ●●● ● ●●●●● ● ● ● ●●●● ●● ●●● ●●● ●● ● ●●

**4lhwA_Rab8_hum**  7 **YLFKLLLIGDSGVGKTCVLFRFSEDAFNSTFISTIGIDFKIRTIELDGKRIKLQIWDTAGQERFRTITTAYYRGAMGIMLVYDITNEKSFDNIRNWIRNIEEHASADVEKMILGNKCDV.N.D.KRQVSKERGEKLALDYGIKFMETSAKANINVENAFFTLARDI** 169*

**4iruB_Rab1a_hu**  17 **YLFKLLLIGDSGVGKSCLLLRFADDTYTESYISTIGVDFKIRTIELDGKTIKLQIWDTAGQERFRTITSSYYRGAHGIIVVYDVTDQESFNNVKQWLQEIDRYASENVNKLLVGNKCDL.T.T.KKVVDYTTAKEFADSLGIPFLETSAKNATNVEQSFMTMAAEK** 179

**3cphA_Sec4_yea**  20 **-IMKILLIGDSGVGKSCLLVRFVEDKFNPSFITTIGIDFKIKTVDINGKKVKLQLWDTAGQERFRTITTAYYRGAMGIILVYDVTDERTFTNIKQWFKTVNEHANDEAQLLLVGNKSDM.E.T.-RVVTADQGEALAKELGIPFIESSAKNDDNVNEIFFTLAKLI** 180

**XP_009058470.1**  7 **YLFKLLLIGDSGVGKTCLLFRFSEDAFNSTFISTIGIDFKIRTIELDGKKIKLQIWDTAGQERFRTITTAYYRGAMGIMLVYDITSEKSFENIRNWIRNIEEHASKDVEKMILANKCDM.N.D.RRQVSKERGENLAVEHGIKFMETSAKASINVDEAFFTLARD-** 168

**XP_008892849.1**  10 **-LIKLLLIGDSGVGKSCVLLRYSDDSFTTSFITTIGIDFKVKTIDVDGKRIKLQIWDTAGQERFRTITTAYYRGAMGILLVYDVTDDHSFQNIRNWMTQIRQNASSNVNKILIGNKCDV.DpS.ERAVTTKQGQDLADEFGIKFFETSAKSNHNIDEAFRSIAVD-** 171

**EWC44734.1**  9 **FLIKLLLIGDSGVGKSCCLLRFSEDSFTPSFITTIGIDFKIRTIDLDGKRIKLQIWDTAGQERFRTITTAYYRGAMGILLVYDVTDERSFNNIQTWFQNVEQHATEGVNKILIGNKCDW.E.E.KRVVSTERGQALANELGIPFMEVSAKANINVEEAFLLLARD-** 170

**RAB8A_DICDI**  14 **YLIKLLLIGDSGVGKSCLLLRFSEDSFTPSFITTIGIDFKIRTIELEGKRIKLQIWDTAGQERFRTITTAYYRGAMGILLVYDVTDEKSFGNIRNWIRNIEQHATDSVNKMLIGNKCDM.A.E.KKVVDSSRGKSLADEYGIKFLETSAKNSINVEEAFISLAKD-** 175

**NP_001116975.1**  7 **FLFKLLLIGDSGVGKTCVLFRFSEDAFNSTFISTIGIDFKIRTIELDGKKIKLQIWDTAGQERFRTITTAYYRGAMGIMLVYDITNQKSFDNIRNWIRNIEEHASADVEKMILGNKCDM.D.D.RRAVSKEKGEQLAIEYGIKFMETSAKASINVEEAFVTLARD-** 168

**XP_002111764.1**  7 **YLFKLLLIGDSGVGKTCVLFRFSEDAFNSTFISTIGIDFKIRTIELDGKKIKLQIWDTAGQERFRTITTAYYRGAMGIMLVFDITNERSFENIKTWIRNIEEHAASDVEKMILGNKCDV.V.D.KRQVSKEQAQQLAAEYGVKFSETSAKDGHNVEEAFMTLAKD-** 168

**XP_002157639.1**  7 **YLFKLLLIGDSGVGKTCVLFRFSEDAFNSTFISTIGIDFKIRTIDLDGKKIKLQIWDTAGQERFRTITTAYYRGAMGIMLVYDITNDKSFENIKNWIRNIEEHAAADVEKMILGNKCDM.N.D.KRQVSAERGQALAMDHNVKFMETSAKTSANVEEAFISLARD-** 168

**XP_002682085.1**  16 **YLCKLLLIGDSGVGKSCLLLRFSDDTFTTNFITTIGIDFKIRTIELDGKRVKLQIWDTAGQERFRTITTAYYRGAMGIMLTYDVTDEQSFLNIRNWMKNIEEHAADNVNKMLIGNKCDL.I.E.KKIVETERGQSLAKSYGIPFMETSAKNNINVEEAFFTIARE-** 177

**KFH62976.1**  11 **YLIKLLLIGDSGVGKSCLLLRFSDDSFTPSFITTIGIDFKIRTIELDGKRIKLQIWDTAGQERFRTITTAYYRGAMGILLVYDVTDERSFSNIRNWFSNVEQHASEGVNKILIGNKCDM.L.D.KKVVQKDQGQTLADEFGIKFLETSAKSNICVEEAFFSLARD-** 172

**EWM27169.1**  15 **HLIKLLLIGDSGVGKSCLLLRYSEDSFTPSFITTIGIDFKIKSLPLEDKKLKLQIWDTAGQERFRTITTAYYRGAMGILLVYDVTDERSFANVKNWMRQIEQHASESVNKILIGNKCDV.DpA.DRRVSLEQGRKLAEEYRIKFFETSAKENVNVDEAFYTVARD-** 177

**XP_004347560.1**  7 **YLFKLLLIGDSGVGKTCVLFRFSDDAFNATFISTIGIDFKIRTIELDGKKIKLQIWDTAGQERFRTITTAYYRGAMGIMLVYDVTQDKTFENIKNWIRNIEQHASEDVEKMILGNKCDV.D.D.KRVVTKERGEQLAREYNVRFFETSAKANINVEEAFLTIARD-** 168

**XP_003079335.1**  20 **HLLKLLLIGDSGVGKSCLLLRFSDDQFTTSFITTIGIDFKIKTVELGGKRVKLQIWDTAGQERFRTITTAYYRGAMGILLVYDVSDERSFENVRGWMRNIEQHASSNVNKILIGNKCDVkE.D.KRVISKARGQALADEFGIPFFETSAKSSVNVNDAFMTIAGD-** 182

**XP_005824947.1**  13 **YLVKLLLIGDSGVGKSCLLLRFSDDSFTTSFITTIGIDFKIRTIEQEGKRLKLQIWDTAGQERFRTITTAYYRGAMGILLVYDVTDEQSFNNIRNWMRQIQQHASDNVNKILIGNKCDM.L.D.KKVIETARGQALADEFGIKFFETSAKNNINVEKAFTEIARDV** 175

**AM764450.1_EST**  25 **FLFKLLLIGDSGVGKTCILFRFSEDAFNSTFISTIGIDFKIRTIELDGKKIKLQIWDTAGQERFRTITTAYYRGAMGIMLVYDITNDKSFDNIKNWIRNIEEHASADVERMILGNKCDM.D.E.KRAVSKERGEQLAVEYGVKFMETSAKASINVEEAFITLAKD-** 186

**GO183057.1_EST**  6 **YLFKLLLIGDSGVGKTCVLFRFSEDAFNSTFISTIGIDFKIRTIELDGKKIKLQIWDTAGQERFRTITTAYYRGAMGIMLVYDITCEKSFENIRNWIRNIEEHASSDVEKMILGNKCDM.N.E.KRQVSKQRGEALAIEYGVKFLETSAKASINVEEGFFTLARD-** 167

**GW342194.1_EST**  11 **HLFKLLLIGDSGVGKTCVLFRFSEDAFNATFISTIGIDFKIRTIELDGKKIKLQIWDTAGQERFRTITTAYYRGAMGIMLVYDITNERSFDNIKNWIRNIEEHASSDVEKMVLGNKCDM.N.D.RRVVSRDRGEQLAVEYGIKFMETSAKNADNVDEAFYSLARD-** 172

**JZ553329.1_EST**  15 **YLIKLLLIGDSGVGKSCLLLRFSEDQFTPSFITTIGIDFKIRSIELDGKRIKLQIWDTAGQERFRTITTAYYRGAMGILLVYDVTEEKTFLNINNWIRNIEQHASENVSMILVGNKCDM.V.D.RKVVDPERAQQLANEYGIKFLETSAKSNHNVDEAFISLTRD-** 176

**EV602546.1_EST**  10 **-LYKLLLIGDSGVGKTSLLFRFAEDAFNATFISTIGIDFKIRTIDIDGKKIKLQVWDTAGQERFRTITTAYYRGAMGIILVYDVTCQTSFDNIRNWIKNTDEHASNDVEKMILGNKCDI.E.G.KRVITYEKGERLAQEYNVKFYETSARESVNVETAFIEFAR--** 169

**foreground (127192):**  **KIRNVGVVGDSGAGKSSLTNAFAG EGAVTDEAGTGRTATSATVEVGGGGVDFTFWDTAGQSDYRGEVEEGAAGADGAVFVYDASDGDSAETVAGWDEEVKEAALEGKPVVVAGTKADK A D ADGGAADEAEAFAEELGAPYFETSAKTGDGVDDVFDAVAELL**

**RPPRLLLL LPDV TT L RLLK LVL PTITPDI IKLL LE KKIKLVLI LP LRRF KLL RFLREV LLLLLLSITEPETLQELKKLLKQLLKLL LKL LLLLLN M L P E REVSELLLREL LLP I ILPI LK ENLEELLE LLR P**

**LLKIVII HVNS I KII KFI V INV VQ I ID ED INIV G HEK S YIKD VIIV I V R F DITE F IR HI DI IIIII L L K EIIK KK V VI V I IVK**

**wt_res_freqs (31893): 111131239113299526132112 1111111111312111112111111111111852911111111111111342221516312212111111111211111112141221128191 1 1 211111111111211111211116522314211111111112**

**11111121 1213 45 2 2421 111 1312121 1111 11 11112132 14 1112 121 112211 1133211121111111111111122112 111 122126 1 3 1 1 11111111112 111 1 1111 11 12112322 211 2**

**1123122 1131 1 111 111 1 211 11 1 11 11 1122 1 121 1 1111 1221 1 1 1 1 1111 1 11 11 11 12111 1 1 1 2111 11 1 22 2 2 211**

**insertions**

**deletions 421965533322221111 311 1 14 1 661176 55356632114 13111 8 71333 11 71132212221134919161221777 2 9 7 781111111175161118117 1112223344576771114**

**position**  10 . 20 . 30 . 40 . 50 . 60 . 70 . 80 . 90 . 100 . 110 . 120 . 130 . 140 . 150 . 160 .

**_**

**_**

**_ _**

**_ _**

**_ _**

**_ _**

**_ _**

**_ _**

**_ _ _**

**_ _ _**

**_ _ _**

**_ _ _**

**_ _ _ _**

**_ _ _ _ _**

**_ _ _ _ _**

**_ _ _ _ _**

**_ _ __ _ _**

**_ _ __ _ _ _**

**_ _ __ _ _ _**

**_ _ __ _ _ _**

**_ _ _ __ _ _ _**

**_ _ _ __ _ _ _**

**_ _ _ __ _ _ _ _**

**_ _ _ __ _ _ _ _**

**_ _ _ __ _ _ _ _**

**_ _ _ __ _ _ _ _ _**

**_ _ _ __ _ _ _ _ _**

**_ _ _ __ _ _ _ _ _**

**_ _ _ _ __ _ _ _ _ _**

**_ _ _ _ __ _ _ _ _ _**

**_ _ _ _ __ _ _ _ _ _**

**_ _ _ _ __ _ _ _ _ _**

**_ _ _ _ __ _ _ _ _ _ _ _**

**_ _ _ _ __ _ _ _ _ _ _ _**

**_ _ _ _ __ _ _ _ _ _ _ _**

**_ _ _ _ __ _ _ _ _ _ _ _**

**_ _ _ _ _ __ _ _ _ _ _ _ _ _**

**_ _ _ _ _ __ _ _ _ _ _ _ _ _**

**_ _ _ _ _ __ _ _ _ _ _ _ _ _ _**

**_ _ _ _ _ __ _ _ _ _ _ _ _ _ _**

**_ _ _ __ _ _ __ _ _ _ _ _ _ _ _ _**

**_ _ _ __ _ _ __ _ _ _ _ _ _ _ _ _**

**_ _ _ _ __ _ _ _ __ _ _ _ _ _ _ _ _ _**

**_ _ _ _ __ _ _ _ __ _ _ _ _ _ _ _ _ _**

**_ _ _ _ __ _ _ _ __ _ _ _ _ _ _ _ _ _**

**_ _ _ _ _ __ _ _ _ __ _ _ _ _ _ _ _ _ _ _**

**_ _ _ _ _ __ _ _ _ __ _ _ _ _ _ _ _ _ _ _**

**_ _ _ _ _ __ _ _ _ __ _ _ _ _ _ _ _ _ _ _**

**_ _ _ _ _ _ __ _ _ _ _ __ _ _ _ _ _ _ _ _ _ _**

**_ _ _ _ _ _ __ _ _ __ _ _ _ _ _ _ __ _ _ _ _ _ _ _ _ _ _**

**_ _ _ _ _ _ __ _ _ __ _ _ _ _ _ _ __ _ _ _ _ _ _ _ _ _ _**

**R^4^ family**  ● ● ● ● ● ● ●● ● ● ●● ● ● ● ● ● ● ●● ● ● ● ● ● ● ● ● ● ●

**4lhwA_Rab8_hum**  7 **YLFKLLLIGDSGVGKTCVLFRFSEDAFNSTFISTIGIDFKIRTIELDGKRIKLQIWDTAGQERFRTITTAYYRGAMGIMLVYDITNEKSFDNIRNWIRNIEEHASADVEKMILGNKCDV.N.D.KRQVSKERGEKLALDYGIKFMETSAKANINVENAFFTLARDI** 169*

**4iruB_Rab1a_hu**  17 **YLFKLLLIGDSGVGKSCLLLRFADDTYTESYISTIGVDFKIRTIELDGKTIKLQIWDTAGQERFRTITSSYYRGAHGIIVVYDVTDQESFNNVKQWLQEIDRYASENVNKLLVGNKCDL.T.T.KKVVDYTTAKEFADSLGIPFLETSAKNATNVEQSFMTMAAEK** 179

**3cphA_Sec4_yea**  20 **-IMKILLIGDSGVGKSCLLVRFVEDKFNPSFITTIGIDFKIKTVDINGKKVKLQLWDTAGQERFRTITTAYYRGAMGIILVYDVTDERTFTNIKQWFKTVNEHANDEAQLLLVGNKSDM.E.T.-RVVTADQGEALAKELGIPFIESSAKNDDNVNEIFFTLAKLI** 180

**XP_009058470.1**  7 **YLFKLLLIGDSGVGKTCLLFRFSEDAFNSTFISTIGIDFKIRTIELDGKKIKLQIWDTAGQERFRTITTAYYRGAMGIMLVYDITSEKSFENIRNWIRNIEEHASKDVEKMILANKCDM.N.D.RRQVSKERGENLAVEHGIKFMETSAKASINVDEAFFTLARD-** 168

**XP_008892849.1**  10 **-LIKLLLIGDSGVGKSCVLLRYSDDSFTTSFITTIGIDFKVKTIDVDGKRIKLQIWDTAGQERFRTITTAYYRGAMGILLVYDVTDDHSFQNIRNWMTQIRQNASSNVNKILIGNKCDV.DpS.ERAVTTKQGQDLADEFGIKFFETSAKSNHNIDEAFRSIAVD-** 171

**EWC44734.1**  9 **FLIKLLLIGDSGVGKSCCLLRFSEDSFTPSFITTIGIDFKIRTIDLDGKRIKLQIWDTAGQERFRTITTAYYRGAMGILLVYDVTDERSFNNIQTWFQNVEQHATEGVNKILIGNKCDW.E.E.KRVVSTERGQALANELGIPFMEVSAKANINVEEAFLLLARD-** 170

**RAB8A_DICDI**  14 **YLIKLLLIGDSGVGKSCLLLRFSEDSFTPSFITTIGIDFKIRTIELEGKRIKLQIWDTAGQERFRTITTAYYRGAMGILLVYDVTDEKSFGNIRNWIRNIEQHATDSVNKMLIGNKCDM.A.E.KKVVDSSRGKSLADEYGIKFLETSAKNSINVEEAFISLAKD-** 175

**NP_001116975.1**  7 **FLFKLLLIGDSGVGKTCVLFRFSEDAFNSTFISTIGIDFKIRTIELDGKKIKLQIWDTAGQERFRTITTAYYRGAMGIMLVYDITNQKSFDNIRNWIRNIEEHASADVEKMILGNKCDM.D.D.RRAVSKEKGEQLAIEYGIKFMETSAKASINVEEAFVTLARD-** 168

**XP_002111764.1**  7 **YLFKLLLIGDSGVGKTCVLFRFSEDAFNSTFISTIGIDFKIRTIELDGKKIKLQIWDTAGQERFRTITTAYYRGAMGIMLVFDITNERSFENIKTWIRNIEEHAASDVEKMILGNKCDV.V.D.KRQVSKEQAQQLAAEYGVKFSETSAKDGHNVEEAFMTLAKD-** 168

**XP_002157639.1**  7 **YLFKLLLIGDSGVGKTCVLFRFSEDAFNSTFISTIGIDFKIRTIDLDGKKIKLQIWDTAGQERFRTITTAYYRGAMGIMLVYDITNDKSFENIKNWIRNIEEHAAADVEKMILGNKCDM.N.D.KRQVSAERGQALAMDHNVKFMETSAKTSANVEEAFISLARD-** 168

**XP_002682085.1**  16 **YLCKLLLIGDSGVGKSCLLLRFSDDTFTTNFITTIGIDFKIRTIELDGKRVKLQIWDTAGQERFRTITTAYYRGAMGIMLTYDVTDEQSFLNIRNWMKNIEEHAADNVNKMLIGNKCDL.I.E.KKIVETERGQSLAKSYGIPFMETSAKNNINVEEAFFTIARE-** 177

**KFH62976.1**  11 **YLIKLLLIGDSGVGKSCLLLRFSDDSFTPSFITTIGIDFKIRTIELDGKRIKLQIWDTAGQERFRTITTAYYRGAMGILLVYDVTDERSFSNIRNWFSNVEQHASEGVNKILIGNKCDM.L.D.KKVVQKDQGQTLADEFGIKFLETSAKSNICVEEAFFSLARD-** 172

**EWM27169.1**  15 **HLIKLLLIGDSGVGKSCLLLRYSEDSFTPSFITTIGIDFKIKSLPLEDKKLKLQIWDTAGQERFRTITTAYYRGAMGILLVYDVTDERSFANVKNWMRQIEQHASESVNKILIGNKCDV.DpA.DRRVSLEQGRKLAEEYRIKFFETSAKENVNVDEAFYTVARD-** 177

**XP_004347560.1**  7 **YLFKLLLIGDSGVGKTCVLFRFSDDAFNATFISTIGIDFKIRTIELDGKKIKLQIWDTAGQERFRTITTAYYRGAMGIMLVYDVTQDKTFENIKNWIRNIEQHASEDVEKMILGNKCDV.D.D.KRVVTKERGEQLAREYNVRFFETSAKANINVEEAFLTIARD-** 168

**XP_003079335.1**  20 **HLLKLLLIGDSGVGKSCLLLRFSDDQFTTSFITTIGIDFKIKTVELGGKRVKLQIWDTAGQERFRTITTAYYRGAMGILLVYDVSDERSFENVRGWMRNIEQHASSNVNKILIGNKCDVkE.D.KRVISKARGQALADEFGIPFFETSAKSSVNVNDAFMTIAGD-** 182

**XP_005824947.1**  13 **YLVKLLLIGDSGVGKSCLLLRFSDDSFTTSFITTIGIDFKIRTIEQEGKRLKLQIWDTAGQERFRTITTAYYRGAMGILLVYDVTDEQSFNNIRNWMRQIQQHASDNVNKILIGNKCDM.L.D.KKVIETARGQALADEFGIKFFETSAKNNINVEKAFTEIARDV** 175

**AM764450.1_EST**  25 **FLFKLLLIGDSGVGKTCILFRFSEDAFNSTFISTIGIDFKIRTIELDGKKIKLQIWDTAGQERFRTITTAYYRGAMGIMLVYDITNDKSFDNIKNWIRNIEEHASADVERMILGNKCDM.D.E.KRAVSKERGEQLAVEYGVKFMETSAKASINVEEAFITLAKD-** 186

**GO183057.1_EST**  6 **YLFKLLLIGDSGVGKTCVLFRFSEDAFNSTFISTIGIDFKIRTIELDGKKIKLQIWDTAGQERFRTITTAYYRGAMGIMLVYDITCEKSFENIRNWIRNIEEHASSDVEKMILGNKCDM.N.E.KRQVSKQRGEALAIEYGVKFLETSAKASINVEEGFFTLARD-** 167

**GW342194.1_EST**  11 **HLFKLLLIGDSGVGKTCVLFRFSEDAFNATFISTIGIDFKIRTIELDGKKIKLQIWDTAGQERFRTITTAYYRGAMGIMLVYDITNERSFDNIKNWIRNIEEHASSDVEKMVLGNKCDM.N.D.RRVVSRDRGEQLAVEYGIKFMETSAKNADNVDEAFYSLARD-** 172

**JZ553329.1_EST**  15 **YLIKLLLIGDSGVGKSCLLLRFSEDQFTPSFITTIGIDFKIRSIELDGKRIKLQIWDTAGQERFRTITTAYYRGAMGILLVYDVTEEKTFLNINNWIRNIEQHASENVSMILVGNKCDM.V.D.RKVVDPERAQQLANEYGIKFLETSAKSNHNVDEAFISLTRD-** 176

**EV602546.1_EST**  10 **-LYKLLLIGDSGVGKTSLLFRFAEDAFNATFISTIGIDFKIRTIDIDGKKIKLQVWDTAGQERFRTITTAYYRGAMGIILVYDVTCQTSFDNIRNWIKNTDEHASNDVEKMILGNKCDI.E.G.KRVITYEKGERLAQEYNVKFYETSARESVNVETAFIEFAR--** 169

**foreground (18901):**  **YLYKVVVVGDGGVGKSCITVQYATGTFSDSYDATVGADNKSKTVTVNGETVTFEVWDTAGQEDYDAITSASYRGGDGAVVCYSVTSKSSFNSAEKWRDDVKEHCGENVVVVVVGTKCDL E S DRAVSTDDGEAYAESNGCKYYECSAKSGTGVDDAFETAAKAR**

**L LLLL SA TSLLLRFLKDR PEESIP I VEFFVR LELEDRPIKLQLL RFRRMRPLF DTHVILLVFDL RPETLENLRR LPQLLRVKDPDIPIMLLAN I R H K VIP EQARKL RKLKLPFM V LENINIKKL LELLRKL**

**I IIII A II LVENK V D VS F YT I IDNKR I E TL RSY NSQAVII I DRD D IK IKEIRKYAN LII V E Q Q T E KQF KEINIL L T D E EEV II Q**

**wt_res_freqs (4386): 43182522962479942111121121711151182815111423131411311116987977131122111653124221162352117711112711111111112311216819196 2 1 171732114211711141131829851212611381113111**

**1 2133 31 54631551121 121114 5 324111 1111111236321 453112221 11111356361 1112123211 2113111112124317117 1 1 1 1 111 313113 11112141 1 112161111 1131313**

**1 2112 1 11 12121 1 1 11 1 22 2 24131 5 1 14 125 1112122 4 231 1 21 114321121 111 1 1 1 1 2 4 112 221111 1 4 1 1 232 21 1**

**insertions**

**deletions 653111111111987765443221111111422333211 1 1111 1111122333333433333333211 1 1 1 1 1 3 6 1221111112 11 11233456778911111111225**

**background (108291):**  **KIRKVGVAGRVGAGKSSTFNAIAG EGAVTDEATVGRTAGTALVEVGGGGRDFTFVDTAGQEDFEGLWEEGAADADGAVVVVDVADGDSAETVAGLDEETKEAILEGKPTVVAATKADK A G ARGGAADVREALAELLGAPFFGTSAKTGDGVDDVLDAVADLE**

**RPPRLLLL LPDV TTLLERLLK LVL PFPEITPDIIIV L LE KKLKLILI LP LLKGRKTL RFLREL LLLLLL ATEPVR QELTE LLQLLKLL LKL LLLLLN M L P D DEL ELLLRE LL P I ILPI LK ENLEEL E LLK P**

**LLVIVII H NH FI K I KAI GT INVTVQ I ID EDI IVI G IRE AEV YIK V VIIH I SS DI FK IR NI IIIII L L T K EIIK V VI V R I IVE L**

**wt_res_freqs (27507): 111131218112299521132112 1111111131312111112111111111112852911221121111111241221546112211111111111111111112141231128191 1 1 211111111111111111211116521314211122211111**

**11111121 1312 465211521 111 111112122111 1 11 11112132 15 11111111 113111 112321 311111 11111 11122112 111 122126 1 3 1 1 111 111111 11 1 1 1112 11 111123 2 211 2**

**1113132 1 31 12 1 1 111 11 211111 1 11 111 112 1 111 111 111 1 1221 1 11 11 11 11 11 13111 1 1 1 1 2111 1 22 2 1 2 211 1**

**position**  10 . 20 . 30 . 40 . 50 . 60 . 70 . 80 . 90 . 100 . 110 . 120 . 130 . 140 . 150 . 160 .

**_**

**_**

**_**

**_**

**_**

**_**

**_**

**_**

**_**

**_ _**

**_ _**

**_ _**

**_ _**

**_ _**

**_ _**

**_ _**

**_ _ _**

**_ _ _**

**_ _ _**

**_ _ _ _**

**_ _ _ _**

**_ _ _ _**

**_ _ _ _ _**

**_ _ _ _ _ _**

**_ _ _ _ _ _ _ _**

**_ _ _ _ _ _ _ _**

**_ _ _ _ _ _ _ _**

**_ _ _ _ _ _ _ _**

**_ _ _ _ _ _ _ _**

**_ _ _ _ _ _ _ _**

**_ _ _ _ _ _ _ _**

**_ _ _ _ _ _ _ _**

**__ _ _ _ _ _ _ _**

**__ _ _ _ _ _ _ _ _**

**__ _ _ _ _ _ _ _ _**

**__ _ _ _ __ _ _ _ _**

**__ _ _ _ _ __ _ __ _ _**

**__ _ _ _ _ __ _ __ _ _**

**__ _ _ _ _ __ _ __ _ _**

**__ _ _ _ _ _ __ _ __ _ _**

**__ _ _ _ _ _ __ _ __ _ _ _ _ _**

**_ __ _ _ _ _ _ ____ __ _ _ _ _ _**

**_ __ _ _ _ _ _ ____ __ _ _ _ _ _**

**_ ____ _ _ _ _ ___ ____ ____ _ _ _ _**

**_ _ ____ _ _ _ _ _ ________ ______ _ _ _ _ _**

**_ _ ____ _ _ _ _ _ ________ ______ _ _ _ _ _**

**_ _ ____ _ _ _ _ _ ________ ______ _ _ _ _ _**

**_ _ ____ _ _ _ _ _ ________ ______ _ _ _ _ _**

**_ _ ____ _ _ _ _ _ ________ ______ _ _ _ _ _**

**_ _ ____ _ _ _ _ _ ________ ______ _ _ _ _ _**

**_ _ ____ _ _ _ _ _ ________ ______ _ _ _ _ _**

**Rab subfamily**  ● ● ●●●● ● ● ● ● ● ●●●●●●●● ●●●●●● ● ● ● ● ●

**4lhwA_Rab8_hum**  7 **YLFKLLLIGDSGVGKTCVLFRFSEDAFNSTFISTIGIDFKIRTIELDGKRIKLQIWDTAGQERFRTITTAYYRGAMGIMLVYDITNEKSFDNIRNWIRNIEEHASADVEKMILGNKCDV.N.D.KRQVSKERGEKLALDYGIKFMETSAKANINVENAFFTLARDI** 169*

**4iruB_Rab1a_hu**  17 **YLFKLLLIGDSGVGKSCLLLRFADDTYTESYISTIGVDFKIRTIELDGKTIKLQIWDTAGQERFRTITSSYYRGAHGIIVVYDVTDQESFNNVKQWLQEIDRYASENVNKLLVGNKCDL.T.T.KKVVDYTTAKEFADSLGIPFLETSAKNATNVEQSFMTMAAEK** 179

**3cphA_Sec4_yea**  20 **-IMKILLIGDSGVGKSCLLVRFVEDKFNPSFITTIGIDFKIKTVDINGKKVKLQLWDTAGQERFRTITTAYYRGAMGIILVYDVTDERTFTNIKQWFKTVNEHANDEAQLLLVGNKSDM.E.T.-RVVTADQGEALAKELGIPFIESSAKNDDNVNEIFFTLAKLI** 180

**XP_009058470.1**  7 **YLFKLLLIGDSGVGKTCLLFRFSEDAFNSTFISTIGIDFKIRTIELDGKKIKLQIWDTAGQERFRTITTAYYRGAMGIMLVYDITSEKSFENIRNWIRNIEEHASKDVEKMILANKCDM.N.D.RRQVSKERGENLAVEHGIKFMETSAKASINVDEAFFTLARD-** 168

**XP_008892849.1**  10 **-LIKLLLIGDSGVGKSCVLLRYSDDSFTTSFITTIGIDFKVKTIDVDGKRIKLQIWDTAGQERFRTITTAYYRGAMGILLVYDVTDDHSFQNIRNWMTQIRQNASSNVNKILIGNKCDV.DpS.ERAVTTKQGQDLADEFGIKFFETSAKSNHNIDEAFRSIAVD-** 171

**EWC44734.1**  9 **FLIKLLLIGDSGVGKSCCLLRFSEDSFTPSFITTIGIDFKIRTIDLDGKRIKLQIWDTAGQERFRTITTAYYRGAMGILLVYDVTDERSFNNIQTWFQNVEQHATEGVNKILIGNKCDW.E.E.KRVVSTERGQALANELGIPFMEVSAKANINVEEAFLLLARD-** 170

**RAB8A_DICDI**  14 **YLIKLLLIGDSGVGKSCLLLRFSEDSFTPSFITTIGIDFKIRTIELEGKRIKLQIWDTAGQERFRTITTAYYRGAMGILLVYDVTDEKSFGNIRNWIRNIEQHATDSVNKMLIGNKCDM.A.E.KKVVDSSRGKSLADEYGIKFLETSAKNSINVEEAFISLAKD-** 175

**NP_001116975.1**  7 **FLFKLLLIGDSGVGKTCVLFRFSEDAFNSTFISTIGIDFKIRTIELDGKKIKLQIWDTAGQERFRTITTAYYRGAMGIMLVYDITNQKSFDNIRNWIRNIEEHASADVEKMILGNKCDM.D.D.RRAVSKEKGEQLAIEYGIKFMETSAKASINVEEAFVTLARD-** 168

**XP_002111764.1**  7 **YLFKLLLIGDSGVGKTCVLFRFSEDAFNSTFISTIGIDFKIRTIELDGKKIKLQIWDTAGQERFRTITTAYYRGAMGIMLVFDITNERSFENIKTWIRNIEEHAASDVEKMILGNKCDV.V.D.KRQVSKEQAQQLAAEYGVKFSETSAKDGHNVEEAFMTLAKD-** 168

**XP_002157639.1**  7 **YLFKLLLIGDSGVGKTCVLFRFSEDAFNSTFISTIGIDFKIRTIDLDGKKIKLQIWDTAGQERFRTITTAYYRGAMGIMLVYDITNDKSFENIKNWIRNIEEHAAADVEKMILGNKCDM.N.D.KRQVSAERGQALAMDHNVKFMETSAKTSANVEEAFISLARD-** 168

**XP_002682085.1**  16 **YLCKLLLIGDSGVGKSCLLLRFSDDTFTTNFITTIGIDFKIRTIELDGKRVKLQIWDTAGQERFRTITTAYYRGAMGIMLTYDVTDEQSFLNIRNWMKNIEEHAADNVNKMLIGNKCDL.I.E.KKIVETERGQSLAKSYGIPFMETSAKNNINVEEAFFTIARE-** 177

**KFH62976.1**  11 **YLIKLLLIGDSGVGKSCLLLRFSDDSFTPSFITTIGIDFKIRTIELDGKRIKLQIWDTAGQERFRTITTAYYRGAMGILLVYDVTDERSFSNIRNWFSNVEQHASEGVNKILIGNKCDM.L.D.KKVVQKDQGQTLADEFGIKFLETSAKSNICVEEAFFSLARD-** 172

**EWM27169.1**  15 **HLIKLLLIGDSGVGKSCLLLRYSEDSFTPSFITTIGIDFKIKSLPLEDKKLKLQIWDTAGQERFRTITTAYYRGAMGILLVYDVTDERSFANVKNWMRQIEQHASESVNKILIGNKCDV.DpA.DRRVSLEQGRKLAEEYRIKFFETSAKENVNVDEAFYTVARD-** 177

**XP_004347560.1**  7 **YLFKLLLIGDSGVGKTCVLFRFSDDAFNATFISTIGIDFKIRTIELDGKKIKLQIWDTAGQERFRTITTAYYRGAMGIMLVYDVTQDKTFENIKNWIRNIEQHASEDVEKMILGNKCDV.D.D.KRVVTKERGEQLAREYNVRFFETSAKANINVEEAFLTIARD-** 168

**XP_003079335.1**  20 **HLLKLLLIGDSGVGKSCLLLRFSDDQFTTSFITTIGIDFKIKTVELGGKRVKLQIWDTAGQERFRTITTAYYRGAMGILLVYDVSDERSFENVRGWMRNIEQHASSNVNKILIGNKCDVkE.D.KRVISKARGQALADEFGIPFFETSAKSSVNVNDAFMTIAGD-** 182

**XP_005824947.1**  13 **YLVKLLLIGDSGVGKSCLLLRFSDDSFTTSFITTIGIDFKIRTIEQEGKRLKLQIWDTAGQERFRTITTAYYRGAMGILLVYDVTDEQSFNNIRNWMRQIQQHASDNVNKILIGNKCDM.L.D.KKVIETARGQALADEFGIKFFETSAKNNINVEKAFTEIARDV** 175

**AM764450.1_EST**  25 **FLFKLLLIGDSGVGKTCILFRFSEDAFNSTFISTIGIDFKIRTIELDGKKIKLQIWDTAGQERFRTITTAYYRGAMGIMLVYDITNDKSFDNIKNWIRNIEEHASADVERMILGNKCDM.D.E.KRAVSKERGEQLAVEYGVKFMETSAKASINVEEAFITLAKD-** 186

**GO183057.1_EST**  6 **YLFKLLLIGDSGVGKTCVLFRFSEDAFNSTFISTIGIDFKIRTIELDGKKIKLQIWDTAGQERFRTITTAYYRGAMGIMLVYDITCEKSFENIRNWIRNIEEHASSDVEKMILGNKCDM.N.E.KRQVSKQRGEALAIEYGVKFLETSAKASINVEEGFFTLARD-** 167

**GW342194.1_EST**  11 **HLFKLLLIGDSGVGKTCVLFRFSEDAFNATFISTIGIDFKIRTIELDGKKIKLQIWDTAGQERFRTITTAYYRGAMGIMLVYDITNERSFDNIKNWIRNIEEHASSDVEKMVLGNKCDM.N.D.RRVVSRDRGEQLAVEYGIKFMETSAKNADNVDEAFYSLARD-** 172

**JZ553329.1_EST**  15 **YLIKLLLIGDSGVGKSCLLLRFSEDQFTPSFITTIGIDFKIRSIELDGKRIKLQIWDTAGQERFRTITTAYYRGAMGILLVYDVTEEKTFLNINNWIRNIEQHASENVSMILVGNKCDM.V.D.RKVVDPERAQQLANEYGIKFLETSAKSNHNVDEAFISLTRD-** 176

**EV602546.1_EST**  10 **-LYKLLLIGDSGVGKTSLLFRFAEDAFNATFISTIGIDFKIRTIDIDGKKIKLQVWDTAGQERFRTITTAYYRGAMGIILVYDVTCQTSFDNIRNWIKNTDEHASNDVEKMILGNKCDI.E.G.KRVITYEKGERLAQEYNVKFYETSARESVNVETAFIEFAR--** 169

**foreground (7002):**  **YLFKVVVVGDSGVGKSCILSQYADGSFSESSKATVGVDFGSKTVTVDGKTVKAQIWDTAGQERYRAVTSAYYRGAAGCVVVYDVSSESSFNSVSSWIDDADTHAGSNVVKVLVGNKCDL A S ERAVSTEDGEAWCDENGVLYFETSAKSGTNVDEAFETVATDY** 301.0

**F L LLLL S TNLMLRFVRKR DPEFIP I IE LIR LQLEDRRI L L F TI RSF SM ILL F ITRRKT EHLRR LKELRQYSDPDIPIM L I E H L V P EAKKLARKLNMPFM LENI IEQ LEMLRKL**

**IIII A S TEDK NLDY T KV IEI K SL T V VII KKQ DNIKK E IEE NE VL I V D E K Q T QQF K Y L L DSE LVKE**

**wt_res_freqs (1341): 77692311977699953161121321821122291966911543134551471869999999827217338897817111889411116911311911111135113332285999296 1 1 182832414221113151112979961328813691115112**

**1 1 2352 1 41712661111 111111 8 23 113 21211123 6 2 7 35 141 11 246 1 472313 21312 516231212222123 1 1 2 1 1 1 1 45212811111271 2111 141 1111213**

**2225 1 3 2121 1112 1 21 322 1 42 1 1 121 111 24211 1 211 11 11 2 1 1 1 2 1 1 113 1 1 3 2 111 3112**

**insertions**

**deletions 4321111111111199876543211111114111111111111 1111 1111 2 1 6 31111111111111 12455678911111111111225**

**background (11899):**  **KSYKCVVVGSSGVGKSCIIVQYTTGEFVDDYDSTVGVDFYSKTVTVNGETYKFQVWDTAGQERYRAITSQYYRGANGAVVCYDVTSKASYDSAEKWREDVKEHAGENVVVVVVGTKCDL E S DREVSTDDGEAYAESIGVKYYECSAKTGEGVNDAFDDAAKAR**

**PRIRLLLL DGA TSLLLRLLKDR PEE IP L AESFV QLVLDDKPITLDLL DDFSSMRPMSIPDSHVILLVFSLDDPETLEELK LLPQLLHVKDPDIPLLLIAN I R E Q PIT EQARKL RKLKLPFL V LEN NIKKL EELLRKL**

**IQF IIII A TI FIEN I V I F N R IEI NQEVE EI K DRLADL KNTQAVII I NRD F NIQ IKEIRRFCN III V D E V E KQF KQ NC I T EEI II Q**

**wt_res_freqs (3045): 11281533911479941111131231711161182814231413131411111115976866241111114532413221254342117111112611111111112211316819197 2 1 171731114111711141141829753212611181122111**

**11212134 532 54621411111 222 15 1 11111 11114111215131 113122431211111213454411211222122 11113111112126117116 1 1 1 1 122 313113 11111131 1 112 52111 1231313**

**112 2111 2 11 4112 1 1 4 1 1 1 212 11131 24 1 115112 11112122 4 231 6 311 114322121 321 1 1 1 1 4 111 21 12 1 4 131 21 1**

**position**  10 . 20 . 30 . 40 . 50 . 60 . 70 . 80 . 90 . 100 . 110 . 120 . 130 . 140 . 150 . 160 .

**_**

**_**

**_**

**_**

**_**

**_**

**_**

**_**

**_**

**_**

**_**

**_**

**_**

**_**

**_ _**

**_ _**

**_ _**

**_ _**

**_ _**

**_ _**

**_ _**

**_ _**

**_ _**

**_ _**

**_ _**

**_ _**

**_ _**

**_ _**

**_ _**

**_ _**

**_ _**

**_ _**

**_ _**

**_ _**

**_ _**

**_ _**

**_ _**

**_ _**

**_ _**

**_ _**

**_ _**

**_ _**

**_ _ _ _**

**_ _ __ _ _ _**

**_ _ _ _ _ __ _ _ _**

**_ _ _ _ _ _ __ _ _ _ _**

**_ _ _ _ _ _ _ _ __ _ _ _ _**

**_ _ _ _ _ _ _ _ _ _ __ __ _ _ _ _ _**

**_ _ _ _ _ _ _ _ _ _ _ _ _ __ __ __ __ ___ _ _ _ _ _ _**

**_ _ _ _ _ _ _ _ _ _ _ _ _ __ __ __ __ ___ _ _ _ _ _ _**

**_ _ _ _ _ _ _ _ _ _ _ _ _ __ __ __ __ ___ _ _ _ _ _ _**

**Rab8**  ● ● ● ● ● ● ● ● ● ● ● ● ● ●● ●● ●● ●● ●●● ● ● ● ● ● ●

**4lhwA_Rab8_hum**  7 **YLFKLLLIGDSGVGKTCVLFRFSEDAFNSTFISTIGIDFKIRTIELDGKRIKLQIWDTAGQERFRTITTAYYRGAMGIMLVYDITNEKSFDNIRNWIRNIEEHASADVEKMILGNKCDV.N.D.KRQVSKERGEKLALDYGIKFMETSAKANINVENAFFTLARDI** 169*

**4iruB_Rab1a_hu**  17 **YLFKLLLIGDSGVGKSCLLLRFADDTYTESYISTIGVDFKIRTIELDGKTIKLQIWDTAGQERFRTITSSYYRGAHGIIVVYDVTDQESFNNVKQWLQEIDRYASENVNKLLVGNKCDL.T.T.KKVVDYTTAKEFADSLGIPFLETSAKNATNVEQSFMTMAAEK** 179

**3cphA_Sec4_yea**  20 **-IMKILLIGDSGVGKSCLLVRFVEDKFNPSFITTIGIDFKIKTVDINGKKVKLQLWDTAGQERFRTITTAYYRGAMGIILVYDVTDERTFTNIKQWFKTVNEHANDEAQLLLVGNKSDM.E.T.-RVVTADQGEALAKELGIPFIESSAKNDDNVNEIFFTLAKLI** 180

**XP_009058470.1**  7 **YLFKLLLIGDSGVGKTCLLFRFSEDAFNSTFISTIGIDFKIRTIELDGKKIKLQIWDTAGQERFRTITTAYYRGAMGIMLVYDITSEKSFENIRNWIRNIEEHASKDVEKMILANKCDM.N.D.RRQVSKERGENLAVEHGIKFMETSAKASINVDEAFFTLARD-** 168

**XP_008892849.1**  10 **-LIKLLLIGDSGVGKSCVLLRYSDDSFTTSFITTIGIDFKVKTIDVDGKRIKLQIWDTAGQERFRTITTAYYRGAMGILLVYDVTDDHSFQNIRNWMTQIRQNASSNVNKILIGNKCDV.DpS.ERAVTTKQGQDLADEFGIKFFETSAKSNHNIDEAFRSIAVD-** 171

**EWC44734.1**  9 **FLIKLLLIGDSGVGKSCCLLRFSEDSFTPSFITTIGIDFKIRTIDLDGKRIKLQIWDTAGQERFRTITTAYYRGAMGILLVYDVTDERSFNNIQTWFQNVEQHATEGVNKILIGNKCDW.E.E.KRVVSTERGQALANELGIPFMEVSAKANINVEEAFLLLARD-** 170

**RAB8A_DICDI**  14 **YLIKLLLIGDSGVGKSCLLLRFSEDSFTPSFITTIGIDFKIRTIELEGKRIKLQIWDTAGQERFRTITTAYYRGAMGILLVYDVTDEKSFGNIRNWIRNIEQHATDSVNKMLIGNKCDM.A.E.KKVVDSSRGKSLADEYGIKFLETSAKNSINVEEAFISLAKD-** 175

**NP_001116975.1**  7 **FLFKLLLIGDSGVGKTCVLFRFSEDAFNSTFISTIGIDFKIRTIELDGKKIKLQIWDTAGQERFRTITTAYYRGAMGIMLVYDITNQKSFDNIRNWIRNIEEHASADVEKMILGNKCDM.D.D.RRAVSKEKGEQLAIEYGIKFMETSAKASINVEEAFVTLARD-** 168

**XP_002111764.1**  7 **YLFKLLLIGDSGVGKTCVLFRFSEDAFNSTFISTIGIDFKIRTIELDGKKIKLQIWDTAGQERFRTITTAYYRGAMGIMLVFDITNERSFENIKTWIRNIEEHAASDVEKMILGNKCDV.V.D.KRQVSKEQAQQLAAEYGVKFSETSAKDGHNVEEAFMTLAKD-** 168

**XP_002157639.1**  7 **YLFKLLLIGDSGVGKTCVLFRFSEDAFNSTFISTIGIDFKIRTIDLDGKKIKLQIWDTAGQERFRTITTAYYRGAMGIMLVYDITNDKSFENIKNWIRNIEEHAAADVEKMILGNKCDM.N.D.KRQVSAERGQALAMDHNVKFMETSAKTSANVEEAFISLARD-** 168

**XP_002682085.1**  16 **YLCKLLLIGDSGVGKSCLLLRFSDDTFTTNFITTIGIDFKIRTIELDGKRVKLQIWDTAGQERFRTITTAYYRGAMGIMLTYDVTDEQSFLNIRNWMKNIEEHAADNVNKMLIGNKCDL.I.E.KKIVETERGQSLAKSYGIPFMETSAKNNINVEEAFFTIARE-** 177

**KFH62976.1**  11 **YLIKLLLIGDSGVGKSCLLLRFSDDSFTPSFITTIGIDFKIRTIELDGKRIKLQIWDTAGQERFRTITTAYYRGAMGILLVYDVTDERSFSNIRNWFSNVEQHASEGVNKILIGNKCDM.L.D.KKVVQKDQGQTLADEFGIKFLETSAKSNICVEEAFFSLARD-** 172

**EWM27169.1**  15 **HLIKLLLIGDSGVGKSCLLLRYSEDSFTPSFITTIGIDFKIKSLPLEDKKLKLQIWDTAGQERFRTITTAYYRGAMGILLVYDVTDERSFANVKNWMRQIEQHASESVNKILIGNKCDV.DpA.DRRVSLEQGRKLAEEYRIKFFETSAKENVNVDEAFYTVARD-** 177

**XP_004347560.1**  7 **YLFKLLLIGDSGVGKTCVLFRFSDDAFNATFISTIGIDFKIRTIELDGKKIKLQIWDTAGQERFRTITTAYYRGAMGIMLVYDVTQDKTFENIKNWIRNIEQHASEDVEKMILGNKCDV.D.D.KRVVTKERGEQLAREYNVRFFETSAKANINVEEAFLTIARD-** 168

**XP_003079335.1**  20 **HLLKLLLIGDSGVGKSCLLLRFSDDQFTTSFITTIGIDFKIKTVELGGKRVKLQIWDTAGQERFRTITTAYYRGAMGILLVYDVSDERSFENVRGWMRNIEQHASSNVNKILIGNKCDVkE.D.KRVISKARGQALADEFGIPFFETSAKSSVNVNDAFMTIAGD-** 182

**XP_005824947.1**  13 **YLVKLLLIGDSGVGKSCLLLRFSDDSFTTSFITTIGIDFKIRTIEQEGKRLKLQIWDTAGQERFRTITTAYYRGAMGILLVYDVTDEQSFNNIRNWMRQIQQHASDNVNKILIGNKCDM.L.D.KKVIETARGQALADEFGIKFFETSAKNNINVEKAFTEIARDV** 175

**AM764450.1_EST**  25 **FLFKLLLIGDSGVGKTCILFRFSEDAFNSTFISTIGIDFKIRTIELDGKKIKLQIWDTAGQERFRTITTAYYRGAMGIMLVYDITNDKSFDNIKNWIRNIEEHASADVERMILGNKCDM.D.E.KRAVSKERGEQLAVEYGVKFMETSAKASINVEEAFITLAKD-** 186

**GO183057.1_EST**  6 **YLFKLLLIGDSGVGKTCVLFRFSEDAFNSTFISTIGIDFKIRTIELDGKKIKLQIWDTAGQERFRTITTAYYRGAMGIMLVYDITCEKSFENIRNWIRNIEEHASSDVEKMILGNKCDM.N.E.KRQVSKQRGEALAIEYGVKFLETSAKASINVEEGFFTLARD-** 167

**GW342194.1_EST**  11 **HLFKLLLIGDSGVGKTCVLFRFSEDAFNATFISTIGIDFKIRTIELDGKKIKLQIWDTAGQERFRTITTAYYRGAMGIMLVYDITNERSFDNIKNWIRNIEEHASSDVEKMVLGNKCDM.N.D.RRVVSRDRGEQLAVEYGIKFMETSAKNADNVDEAFYSLARD-** 172

**JZ553329.1_EST**  15 **YLIKLLLIGDSGVGKSCLLLRFSEDQFTPSFITTIGIDFKIRSIELDGKRIKLQIWDTAGQERFRTITTAYYRGAMGILLVYDVTEEKTFLNINNWIRNIEQHASENVSMILVGNKCDM.V.D.RKVVDPERAQQLANEYGIKFLETSAKSNHNVDEAFISLTRD-** 176

**EV602546.1_EST**  10 **-LYKLLLIGDSGVGKTSLLFRFAEDAFNATFISTIGIDFKIRTIDIDGKKIKLQVWDTAGQERFRTITTAYYRGAMGIILVYDVTCQTSFDNIRNWIKNTDEHASNDVEKMILGNKCDI.E.G.KRVITYEKGERLAQEYNVKFYETSARESVNVETAFIEFAR--** 169

**foreground (3312):**  **YVFKVVIIGDSGVGKSCILSRYADNSYSDSSKATIGVDFATKSVTVDGKTVKAQIWDTAGQERYRAVTSAYYRGAQGAIVVYDVSNEESFNSVSNWLENIDTHASSNVNKILVGNKCDL T S EKAVSTDDGEAFADEEGISFFETSAKTATNVDEAFETVATDK**

**L LLL A TNL L FTRDEFNPEFIP IE KIRTLQLED RI L F TL TS M IML ITRHQT EHLRR RELRRYSDPDIVIM I S M R H LRV PKEEAKEL RRLNLP M LENI EK LEMLRKL**

**I S S T TLDY T QV IEI K I V LI KR DNIKK K EQ NE VL A E E K E T Q EKY L L DS Q Q LIKQY**

**wt_res_freqs (578): 81892419989699973193913341111243199975923312135682394899999999939419659999919431999511125921411961142156214414186999396 1 1 113934125125923253191979952238912791223211**

**7 457 1 137 3 833437212231 14 535521211 14 5 6 51 13 1 416 482114 21113 17431313123424 1 2 1 1 2 272 21534323 111131 2 3221 61 1212111**

**2 2 1 1 2212 1 11 332 1 7 3 41 23 26221 2 21 12 11 1 2 1 2 1 1 1 311 1 2 22 1 1 31114**

**insertions 2**

**deletions 3222111111111111987643321111118111111 1 1 111 3 3 2 23677891111111112222234**

**background (3690):**  **YLYKFVFIGDAGVGKSCFLNRYTNNTFSSNSDATIGVDFGTKTVTVDGKTVKLQIWDTAGQERYRTVTKAYYRGAAGAIVVYDVSSKSSFNSVDSWRDDIEEHAGDGFVKIVVGNKCDL A G ERAVSTEEGEAFCEENGVLFFETSAKSGENVDEAFETVAEAY**

**F L LLLL SS TSLMLQLVEDR DPQYKP V IE LVRILELEDRLIR L FQSLIPSFF DSHAILL F LTRRKT LHLRR LKELLQYSDPDIPIMLL I E E R VIPRK AKRLARKLNMPYM V LEN IKQL LRL RKP**

**F IIIV IIH FCDKK EDHQS A RS EI I N R T IGV N DCVVI I N Q ENI K IE AR L NENVIVL I V D D K Q TE QKW K Y L L DA E S KI KEL**

**wt_res_freqs (763): 76181213971589933141522111821111396956812623134431557859999999816115127795715321779311117811211911311115111121215989196 1 1 181842443313113241153969861418713581116111**

**1 2 1233 51 55611212111 111321 2 22 2121212122121 3 817311521 1111136 2 171312 11411 41411111142133172 1 2 1 1 11111 5112712121221 1 111 2111 114 311**

**4 2422 111 51212 11111 1 12 12 2 1 2 1 411 1 21121 4 3 1 231 1 12 13 1 1123111 2 1 1 2 2 2 11 112 2 1 2 1 11 3 1 12 115**

**position**  10 . 20 . 30 . 40 . 50 . 60 . 70 . 80 . 90 . 100 . 110 . 120 . 130 . 140 . 150 . 160 .

**Figure 2— Source data 1. R^4^ family and Rab subfamily for Rab4**

**Chordata**  7 **FLFKFLVIGNAGTGKSCLLHQFIEKKFKDDSNHTIGVEFGSKIINV.GGKYVKLQIWDTAGLERFRSVTRSYYRGAAGALLVYDITSRETYNALTNWLTDARMLASQNIVIILCGNKKDLDADREVTFLEASRFAQENELM.FLETSALTGEDVEEAFVQCARKIL** 170*

**Chordata**  5 **YIFKYIIIGDMGVGKSCLLHQFTEKKFMADCPHTIGVEFGTRIIEV.SGQKIKLQIWDTAGLERFRAVTRSYYRGAAGALMVYDITRRSTYNHLSSWLTDARNLTNPNTVIILIGNKADLEAQRDVTYEEAKQFAEENGLL.FLEASAKTGENVEDAFLEAAKKIY** 168

**Arthropoda**  9 **YLFKYIIIGDTGVGKSCLLLQFTDKRFQPVHDLTIGVEFGARMITI.DGKQIKLQIWDTAGLEAFRSITRSYYRGAAGALLVYDITRRETFNHLTTWLEDARQHSNSNMVIMLIGNKSDLDSRREVKKEEGEAFAREHGLV.FMETSARTAANVEEAFINTAKEIY** 172

**Arthropoda**  13 **RKFKLVFLGEQSVGKTSLITRFMYDSFDNTYQATIGIDFLSKTMYL.EDRTVRLQLWDTAGQERFRSLIPSYIRDSTVAVVVYDITNTNSFHQTSKWIDDVRTERGSDVIIMLVGNKTDLSDKRQVSTEEGERKAKELNVM.FIETSAKAGYNVKQLFRRVAAALP** 176

**Nematoda**  47 **YLLKFLIIGNAGTGKSCIMHQFIERKFKANSAHTIGVEFGSRMVAL.GGKKVKLQIWDTAGQERFRAVTRSYYRGAAGALLVYDITNRESYNALQQWLIEARNLASAHIVVVLVGNKKDLHEERQVMFLEASQFAQENDLT.FMETSALNGENVEETFLKCAKTIL** 210

**Amoebozoa**  7 **YIFKYIIIGDMGVGKSCLLHQFTENKFVPDSPHTIGVEFGTRIVDV.NNKKIKLQIWDTAGQERFRAVTRSYYRGAAGALLVYDITRRITYNHLTTWLTDARNLTNPNTVIMLIGNKKDLEGQRDVTYEEASAFAKQNGLI.FVESSAKTGENVEEAFLRTAKLIF** 170

**Basidiomycota**  55 **KKFKLVFLGDQNAGKTSLITQFMYGSFDATYQATIGIDFLSKTMYL.EDRTVRLQLWDTAGQERFRSLIPSYIRDSSVAVVVYDITNRSSFASTNKWIDDVRAERGSDVIIVLVGNKTDLNDKRQVTTEEAEKRAKDLGVM.FIETSAKAGHNVKTLFRKIAQALP** 218

**Streptophyta**  34 **YLFKYIIIGDTGVGKSCLLLQFTDKRFQPVHDLTIGVEFGARMINI.ENKAIKLQIWDTAGQESFRSITRSYYRGAAGALLVYDITRRETFNHLASWLEDARQHANSNMTIMLIGNKSDLQHRRAVSTEEGEQFAKENGLI.FMETSAKTAHNVEEAFINTAGSIY** 197

**Alveolata**  5 **YLFKYIIIGDTGVGKSCLLLQFTDKRFRPDHDLTIGVEFGARLVNV.DGKQIKLQIWDTAGQESFRSITRSYYRGATGALLVYDISRRDTFNHLTRWLEEARQNSNPNMVIMLIGNKCDLE-RREVSYDEGAKFARDNNLI.FLEASAKTAQNVEEAFLQTARKIY** 167

**Apicomplexa**  6 **YLFKYIIIGDTGVGKSCLLLQFTDKRFRADHDLTIGVEFGARLINL.DNKQIKLQIWDTAGQESFRSITRSYYRGAAGALLVYDITRRETFNHLNRWLDEVRQNSNPHMAIILVGNKCDLE-RREVSAEEGAQFARQNGLI.FLETSAKTAKNVEEAFLYTARKIY** 168

**Chromerida**  6 **YLFKYIIIGDTGVGKSCLLLQFTDKRFRADHDLTIGVEFGARLINI.DGKQIKLQIWDTAGQESFRSITRSYYRGAAGALLVYDITRRDTFSHLTRWLEEVRQNANPHMTIMLIGNKCDLE-RREVSTEEGASFARQHGLI.FLETSAKTAQNVEEAFIWTARKIY** 168

**Stramenopiles**  5 **YLFKYIIIGDTGVGKSCLLLQFTDKRFQPVHDLTIGVEFGARMISI.DNKQIKLQIWDTAGQESFRSITRSYYRGAAGALLVYDITRRETFNHLTRWLEEARQNSNSNMAIMLIGNKSDLEHRRAVSYKEGEVFAKENGLI.FLETSAKTAANVEDAFIKTAQKI-** 167

**Mortierellomyc**  9 **YIIKYIIIGDTGVGKSCLLLQFTDKRFQPAHDLTIGVEFGARFVTV.AGKQIKLQIWDTAGQESFRSITRSYYRGAAGALLVYDITRRDTFKHLTTWLEDARQHANANTTIMLIGNKSDLEAKRAVSTEEGEAFAKANGLF.FMETSAKTSANVEEAFVETATNIY** 172

**Rhizaria**  5 **YLFKYIIIGDTGVGKSCLLLQFTDKRFRPVHDLTIGVEFGARMITV.DNRQIKLQIWDTAGQESFRSITRSYYRGAAGALLVYDITRRETFAHLARWLEEARQNGNRNMTIMLIGNKSDLEHRRQISKEEGEAFAKDNGLI.FFETSAKTAANVEEAFIQTAEQIY** 168

**Heterolobosea**  6 **YIFKYIIIGDMGVGKSCLLHQFTEQKFQSDCPHTIGVEFGTRVIEV.EGKSIKLQIWDTAGQERFRAVTRSYYRGAAGALMVYDITRRNTFHHLASWLTDARNLTNPHTIIVLIGNKSDLEDQRQVSFEEASRFAKENGLV.YVETSAKTGENVTKAFMETASRIY** 169

**Rhodophyta**  5 **YLFKFIIIGDSGVGKSCLLLQFTDKRFQPIHDLTIGVEFGAKIVQM.DGKTIKLQIWDTAGQESFRSITRSYYRGAAGALLVYDITRRETFNHLATWLEDARQYSSPDMTVVLVGNKCDLEHRRQVSKEEGEAFARAHGLS.FLETSAKTAFHVEETFTEAARTIY** 168

**Bacillariophyt**  5 **YLFKYIIIGDTGVGKSCLLLQFTDKRFQPVHDLTIGVEFGARMISVaDDTQVKLQIWDTAGQESFRSITRSYYRGAAGALLVYDITRRDTFQHLSRWLEEAKSHAQPNMVILLIGNKNDLEHRRAVTAEEGQEFADANGLL.FLETSAKTAFNVEQAFLKTAKSI-** 168

**Glomeromycota**  9 **YIIKYIIIGDTGVGKSCLLLQFTDRRFQPAHDLTIGVEFGARFVDV.EGKKIKLQIWDTAGQESFRSITRSYYRGAAGALLVYDITRRETFDHLLIWLEDARQHSNSNTTIMLIGNKSDLESKRAVSYAEGEAFAQQHGLF.FMETSAKTADNVEESFVDTAKDIY** 172

**Intramacronucl**  6 **YLFKYIIIGDTGVGKSCLLLQFTDKRFRTQHDLTIGVEFGARAVEI.NGETIKLQIWDTAGQESFKSITRSYYRGAAGALLVYDITRRETFNHISTWLDEVRQNGNSDMVIILIGNKSDLDSKRQVSTEEGKSFAKENNLI.FMETSAKNSTNVEDAFLETSKFIY** 169

**Euglenozoa**  6 **YVFKYIIIGDSGVGKSCLLLQFTDKRFEPLHDLTIGVEFGARLISI.QGRSVKLQIWDTAGQESFRSITRSYYRGASGALLVYDVTRRDTFTHLQSWLEDAKANANTALVIMLIGNKCDLDSKRQVSREEGEAFARSNGLM.FMETSAKTSKNVDDAFLQTAALIY** 169

**Cnidaria**  7 **YLFKYLIIGSAGVGKSCILHQFMENKFKQDAHHTIGVEFGSKIVNI.GGKSVKLQIWDTAGQERFRSVTRSYYRGAAGALLVYDITSRETYNTLTTWLTDARTLASPNIVIILLGNKKDLEADREVTFLEASRFAQENELM.FLETSAATGENVEEAFLKCSRSIL** 170

**Placozoa**  8 **FLLKFLIIGSAGTGKSCLLHQFIENKFKQDSSHTIGVEFGSKVINC.SNKTVKLQIWDTAGQERFRSVTKSYYRGAAGALLVYDISNRDSYNALGAWLTDARTLASPQIVIILIGNKKDLESEREVTFLEASRFAQENELM.FLETSALSGENVEEAFLRCSKNIL** 171

**Parabasalia**  6 **YIFKFIIVGNSAVGKSCMLLRFDEDRFQPIHDVTVGVTFSIKMVSI.EGQDVKVQVWDTAGQEIFRSITRSYYRDSACAIIVYDITDQSSFAKVEDWIRDVRNLAPPDCLLALVGNKLDLAAQRAVQTSEGQELAEKHNLL.FFETSAATGENVQELFNE------** 163

**Hemichordata**  15 **FLFKIVLIGDAGVGKTCVVQRFKSGTFLEKQHSTIGVDFTMKTLHI.DGKKVKLQVWDTAGQERFRTITQSYYRSANGVIIAYDITKRESFNNVPRWVEDVKKYAGANVMQMLIGNKRDLESLREVTVSDAKALAHHHGMLeSLETSAKDSTNVEEAFGKLAKEL-** 178

**position**  10 . 20 . 30 . 40 . 50 . 60 . 70 . 80 . 90 . 100 . 110 . 120 . 130 . 140 . 150 . 160 . 170

**_**

**_**

**__**

**__**

**__**

**__**

**__**

**___**

**___ _ _**

**___ _ _ _**

**___ _ _ _**

**___ _ _ _**

**_ ___ _ _ _**

**_ ___ _ _ _**

**_ ___ _ _ _**

**_ ___ _ _ _**

**_ ___ _ _ _ _**

**_ ___ _ _ _ _**

**_ ___ _ _ __ _**

**_ ___ _ _ __ _**

**_ ___ _ _ __ _**

**_ ___ _ _ __ _**

**_ ___ _ _ __ _**

**_ ___ _ _ __ _**

**_ ___ _ _ __ _**

**_ ____ _ _ __ _**

**_ ____ _ _ __ _**

**_ ____ _ _ __ _**

**_ ____ _ _ __ _**

**_ ____ _ _ __ _**

**_ ____ _ _ __ _**

**_ ____ _ _ __ _**

**_ ____ _ _ __ _**

**_ ____ _ _ _ __ _**

**_ _ ____ _ _ _ _ __ _**

**_ _ ____ _ _ _ _ __ _**

**_ _ ____ _ _ _ _ _ __ _**

**_ _ _ _ ____ _ _ _ __ _ __ _**

**_ _ _ _ ____ _ _ _ __ _ _ __ _ _**

**_ ___ _ ____ _ _ _ __ _ _ __ _ _**

**_ ___ _ ____ _ _ _ __ _ __ __ _ _**

**_ ___ _ ____ _ _ _ _ __ _ __ __ _ _**

**_ ___ _ _____ _ _ _ _ __ _ __ __ _ _**

**_ ___ _ _____ _ _ _ _ __ _ __ __ _ _**

**_ ___ _ _____ _ _ _ _ __ _ __ _ __ _ _**

**_ ___ _ _____ _ _ _ _ __ _ __ _ __ _ _**

**_ ___ _ _____ _ _ _ _ __ _ __ __ __ _ _**

**_ ___ _ _____ _ _ _ _ __ _ __ ___ __ _ _**

**_ ___ _ _____ _ _ _ _ __ _ __ ___ __ _ _**

**_ ___ _ _____ _ _ _ ____ __ ___ ___ __ _ __**

**_ ___ _ _____ _ _ _ ____ __ ___ ___ __ _ __**

**GTPases**  ● ●●● ● ●●●●● ● ● ● ●●●● ●● ●●● ●●● ●● ● ●●

**1z0kA_Rab4_hum**  7 **FLFKFLVIGNAGTGKSCLLHQFIEKKFKDDSNHTIGVEFGSKIINV.GGKYVKLQIWDTAGLERFRSVTRSYYRGAAGALLVYDITSRETYNALTNWLTDARMLASQNIVIILCGNKKDLDADREVTFLEASRFAQENELM.FLETSALTGEDVEEAFVQCARKIL** 170*

**4d0gA_Rab14_hu**  5 **YIFKYIIIGDMGVGKSCLLHQFTEKKFMADCPHTIGVEFGTRIIEV.SGQKIKLQIWDTAGLERFRAVTRSYYRGAAGALMVYDITRRSTYNHLSSWLTDARNLTNPNTVIILIGNKADLEAQRDVTYEEAKQFAEENGLL.FLEASAKTGENVEDAFLEAAKKIY** 168

**4rkeA_Rab2_dro**  9 **YLFKYIIIGDTGVGKSCLLLQFTDKRFQPVHDLTIGVEFGARMITI.DGKQIKLQIWDTAGLEAFRSITRSYYRGAAGALLVYDITRRETFNHLTTWLEDARQHSNSNMVIMLIGNKSDLDSRREVKKEEGEAFAREHGLV.FMETSARTAANVEEAFINTAKEIY** 172

**2y8eA_Rab6_dro**  13 **RKFKLVFLGEQSVGKTSLITRFMYDSFDNTYQATIGIDFLSKTMYL.EDRTVRLQLWDTAGQERFRSLIPSYIRDSTVAVVVYDITNTNSFHQTSKWIDDVRTERGSDVIIMLVGNKTDLSDKRQVSTEEGERKAKELNVM.FIETSAKAGYNVKQLFRRVAAALP** 176

**ERG87123.1**  47 **YLLKFLIIGNAGTGKSCIMHQFIERKFKANSAHTIGVEFGSRMVAL.GGKKVKLQIWDTAGQERFRAVTRSYYRGAAGALLVYDITNRESYNALQQWLIEARNLASAHIVVVLVGNKKDLHEERQVMFLEASQFAQENDLT.FMETSALNGENVEETFLKCAKTIL** 210

**Rab14_dicdi**  7 **YIFKYIIIGDMGVGKSCLLHQFTENKFVPDSPHTIGVEFGTRIVDV.NNKKIKLQIWDTAGQERFRAVTRSYYRGAAGALLVYDITRRITYNHLTTWLTDARNLTNPNTVIMLIGNKKDLEGQRDVTYEEASAFAKQNGLI.FVESSAKTGENVEEAFLRTAKLIF** 170

**GAA93714.1**  55 **KKFKLVFLGDQNAGKTSLITQFMYGSFDATYQATIGIDFLSKTMYL.EDRTVRLQLWDTAGQERFRSLIPSYIRDSSVAVVVYDITNRSSFASTNKWIDDVRAERGSDVIIVLVGNKTDLNDKRQVTTEEAEKRAKDLGVM.FIETSAKAGHNVKTLFRKIAQALP** 218

**HO447309.1_EST**  34 **YLFKYIIIGDTGVGKSCLLLQFTDKRFQPVHDLTIGVEFGARMINI.ENKAIKLQIWDTAGQESFRSITRSYYRGAAGALLVYDITRRETFNHLASWLEDARQHANSNMTIMLIGNKSDLQHRRAVSTEEGEQFAKENGLI.FMETSAKTAHNVEEAFINTAGSIY** 197

**XP_002784324.1**  5 **YLFKYIIIGDTGVGKSCLLLQFTDKRFRPDHDLTIGVEFGARLVNV.DGKQIKLQIWDTAGQESFRSITRSYYRGATGALLVYDISRRDTFNHLTRWLEEARQNSNPNMVIMLIGNKCDLE-RREVSYDEGAKFARDNNLI.FLEASAKTAQNVEEAFLQTARKIY** 167

**ETW35364.1**  6 **YLFKYIIIGDTGVGKSCLLLQFTDKRFRADHDLTIGVEFGARLINL.DNKQIKLQIWDTAGQESFRSITRSYYRGAAGALLVYDITRRETFNHLNRWLDEVRQNSNPHMAIILVGNKCDLE-RREVSAEEGAQFARQNGLI.FLETSAKTAKNVEEAFLYTARKIY** 168

**CEM21914.1**  6 **YLFKYIIIGDTGVGKSCLLLQFTDKRFRADHDLTIGVEFGARLINI.DGKQIKLQIWDTAGQESFRSITRSYYRGAAGALLVYDITRRDTFSHLTRWLEEVRQNANPHMTIMLIGNKCDLE-RREVSTEEGASFARQHGLI.FLETSAKTAQNVEEAFIWTARKIY** 168

**XP_008604403.1**  5 **YLFKYIIIGDTGVGKSCLLLQFTDKRFQPVHDLTIGVEFGARMISI.DNKQIKLQIWDTAGQESFRSITRSYYRGAAGALLVYDITRRETFNHLTRWLEEARQNSNSNMAIMLIGNKSDLEHRRAVSYKEGEVFAKENGLI.FLETSAKTAANVEDAFIKTAQKI-** 167

**KFH66701.1**  9 **YIIKYIIIGDTGVGKSCLLLQFTDKRFQPAHDLTIGVEFGARFVTV.AGKQIKLQIWDTAGQESFRSITRSYYRGAAGALLVYDITRRDTFKHLTTWLEDARQHANANTTIMLIGNKSDLEAKRAVSTEEGEAFAKANGLF.FMETSAKTSANVEEAFVETATNIY** 172

**CEP00705.1**  5 **YLFKYIIIGDTGVGKSCLLLQFTDKRFRPVHDLTIGVEFGARMITV.DNRQIKLQIWDTAGQESFRSITRSYYRGAAGALLVYDITRRETFAHLARWLEEARQNGNRNMTIMLIGNKSDLEHRRQISKEEGEAFAKDNGLI.FFETSAKTAANVEEAFIQTAEQIY** 168

**XP_002671605.1**  6 **YIFKYIIIGDMGVGKSCLLHQFTEQKFQSDCPHTIGVEFGTRVIEV.EGKSIKLQIWDTAGQERFRAVTRSYYRGAAGALMVYDITRRNTFHHLASWLTDARNLTNPHTIIVLIGNKSDLEDQRQVSFEEASRFAKENGLV.YVETSAKTGENVTKAFMETASRIY** 169

**XP_005704285.1**  5 **YLFKFIIIGDSGVGKSCLLLQFTDKRFQPIHDLTIGVEFGAKIVQM.DGKTIKLQIWDTAGQESFRSITRSYYRGAAGALLVYDITRRETFNHLATWLEDARQYSSPDMTVVLVGNKCDLEHRRQVSKEEGEAFARAHGLS.FLETSAKTAFHVEETFTEAARTIY** 168

**XP_002182274.1**  5 **YLFKYIIIGDTGVGKSCLLLQFTDKRFQPVHDLTIGVEFGARMISVaDDTQVKLQIWDTAGQESFRSITRSYYRGAAGALLVYDITRRDTFQHLSRWLEEAKSHAQPNMVILLIGNKNDLEHRRAVTAEEGQEFADANGLL.FLETSAKTAFNVEQAFLKTAKSI-** 168

**ESA00154.1**  9 **YIIKYIIIGDTGVGKSCLLLQFTDRRFQPAHDLTIGVEFGARFVDV.EGKKIKLQIWDTAGQESFRSITRSYYRGAAGALLVYDITRRETFDHLLIWLEDARQHSNSNTTIMLIGNKSDLESKRAVSYAEGEAFAQQHGLF.FMETSAKTADNVEESFVDTAKDIY** 172

**ABY63655.1**  6 **YLFKYIIIGDTGVGKSCLLLQFTDKRFRTQHDLTIGVEFGARAVEI.NGETIKLQIWDTAGQESFKSITRSYYRGAAGALLVYDITRRETFNHISTWLDEVRQNGNSDMVIILIGNKSDLDSKRQVSTEEGKSFAKENNLI.FMETSAKNSTNVEDAFLETSKFIY** 169

**XP_010701899.1**  6 **YVFKYIIIGDSGVGKSCLLLQFTDKRFEPLHDLTIGVEFGARLISI.QGRSVKLQIWDTAGQESFRSITRSYYRGASGALLVYDVTRRDTFTHLQSWLEDAKANANTALVIMLIGNKCDLDSKRQVSREEGEAFARSNGLM.FMETSAKTSKNVDDAFLQTAALIY** 169

**XP_002158483.2**  7 **YLFKYLIIGSAGVGKSCILHQFMENKFKQDAHHTIGVEFGSKIVNI.GGKSVKLQIWDTAGQERFRSVTRSYYRGAAGALLVYDITSRETYNTLTTWLTDARTLASPNIVIILLGNKKDLEADREVTFLEASRFAQENELM.FLETSAATGENVEEAFLKCSRSIL** 170

**XP_002115883.1**  8 **FLLKFLIIGSAGTGKSCLLHQFIENKFKQDSSHTIGVEFGSKVINC.SNKTVKLQIWDTAGQERFRSVTKSYYRGAAGALLVYDISNRDSYNALGAWLTDARTLASPQIVIILIGNKKDLESEREVTFLEASRFAQENELM.FLETSALSGENVEEAFLRCSKNIL** 171

**XP_001299604.1**  6 **YIFKFIIVGNSAVGKSCMLLRFDEDRFQPIHDVTVGVTFSIKMVSI.EGQDVKVQVWDTAGQEIFRSITRSYYRDSACAIIVYDITDQSSFAKVEDWIRDVRNLAPPDCLLALVGNKLDLAAQRAVQTSEGQELAEKHNLL.FFETSAATGENVQELFNE------** 163

**XP_002730440.1**  15 **FLFKIVLIGDAGVGKTCVVQRFKSGTFLEKQHSTIGVDFTMKTLHI.DGKKVKLQVWDTAGQERFRTITQSYYRSANGVIIAYDITKRESFNNVPRWVEDVKKYAGANVMQMLIGNKRDLESLREVTVSDAKALAHHHGMLeSLETSAKDSTNVEEAFGKLAKEL-** 178

**foreground (127192):**  **KIRNVGVVGDSGAGKSSLTNAFAG EGAVTDEAGTGRTATSATVEV GGGGVDFTFWDTAGQSDYRGEVEEGAAGADGAVFVYDASDGDSAETVAGWDEEVKEAALEGKPVVVAGTKADKADADGGAADEAEAFAEELGAP YFETSAKTGDGVDDVFDAVAELAL**

**RPPRLLLL LPDV TT L RLLK LVL PTITPDI IKLL L E KKIKLVLI LP LRRF KLL RFLREV LLLLLLSITEPETLQELKKLLKQLLKLL LKL LLLLLN M LPE REVSELLLREL LLP I ILPI LK ENLEELLE LLR LP**

**LLKIVII HVNS I KII KFI V INV VQ I I D ED INIV G HEK S YIKD VIIV I V R F DITE F IR HI DI IIIII L L E EIIK KK V VI V I IVK I**

**wt_res_freqs (31864): 111131239113299526132112 111111111131211111211 1111111111852911111111111111342221516312212111111111211111112141221128191112111111111112111112 111165223142111111111112**

**11111121 1213 45 2 2421 111 1312121 1111 1 1 11112132 14 1112 121 112211 1133211121111111111111122112 111 122126 1 311 21111111112 111 1 1111 11 12112322 211 22**

**1123122 1131 1 111 111 1 211 11 1 1 1 11 1122 1 121 1 1111 1221 1 1 1 1 1111 1 11 11 11 12111 1 1 1 2111 11 1 22 2 2 211 1**

**insertions**

**deletions 421965533322221111 311 1 14 1 661176 55356 632114 13111 8 7 333 11 71132212221134919161221777 2977811111111751611181 17 11122233445767711124**

**position**  10 . 20 . 30 . 40 . 50 . 60 . 70 . 80 . 90 . 100 . 110 . 120 . 130 . 140 . 150 . 160 . 170

**_**

**_**

**_ _**

**_ _**

**_ _**

**_ _**

**_ _**

**_ _**

**_ _ _**

**_ _ _**

**_ _ _**

**_ _ _**

**_ _ _ _**

**_ _ _ _ _**

**_ _ _ _ _**

**_ _ _ _ _**

**_ _ __ _ _**

**_ _ __ _ _ _**

**_ _ __ _ _ _**

**_ _ __ _ _ _**

**_ _ _ __ _ _ _**

**_ _ _ __ _ _ _**

**_ _ _ __ _ _ _ _**

**_ _ _ __ _ _ _ _**

**_ _ _ __ _ _ _ _**

**_ _ _ __ _ _ _ _ _**

**_ _ _ __ _ _ _ _ _**

**_ _ _ __ _ _ _ _ _**

**_ _ _ _ __ _ _ _ _ _**

**_ _ _ _ __ _ _ _ _ _**

**_ _ _ _ __ _ _ _ _ _**

**_ _ _ _ __ _ _ _ _ _**

**_ _ _ _ __ _ _ _ _ _ _ _**

**_ _ _ _ __ _ _ _ _ _ _ _**

**_ _ _ _ __ _ _ _ _ _ _ _**

**_ _ _ _ __ _ _ _ _ _ _ _**

**_ _ _ _ _ __ _ _ _ _ _ _ _ _**

**_ _ _ _ _ __ _ _ _ _ _ _ _ _**

**_ _ _ _ _ __ _ _ _ _ _ _ _ _ _**

**_ _ _ _ _ __ _ _ _ _ _ _ _ _ _**

**_ _ _ __ _ _ __ _ _ _ _ _ _ _ _ _**

**_ _ _ __ _ _ __ _ _ _ _ _ _ _ _ _**

**_ _ _ _ __ _ _ _ __ _ _ _ _ _ _ _ _ _**

**_ _ _ _ __ _ _ _ __ _ _ _ _ _ _ _ _ _**

**_ _ _ _ __ _ _ _ __ _ _ _ _ _ _ _ _ _**

**_ _ _ _ _ __ _ _ _ __ _ _ _ _ _ _ _ _ _ _**

**_ _ _ _ _ __ _ _ _ __ _ _ _ _ _ _ _ _ _ _**

**_ _ _ _ _ __ _ _ _ __ _ _ _ _ _ _ _ _ _ _**

**_ _ _ _ _ _ _ __ _ _ _ _ __ _ _ _ _ _ _ _ _ _ _**

**_ _ _ _ _ _ __ _ _ __ _ _ _ _ _ _ __ _ _ _ _ _ _ _ _ _ _**

**_ _ _ _ _ _ __ _ _ __ _ _ _ _ _ _ __ _ _ _ _ _ _ _ _ _ _**

**R^4^ family**  ● ● ● ● ● ● ●● ● ● ●● ● ● ● ● ● ● ●● ● ● ● ● ● ● ● ● ● ●

**1z0kA_Rab4_hum**  7 **FLFKFLVIGNAGTGKSCLLHQFIEKKFKDDSNHTIGVEFGSKIINV.GGKYVKLQIWDTAGLERFRSVTRSYYRGAAGALLVYDITSRETYNALTNWLTDARMLASQNIVIILCGNKKDLDADREVTFLEASRFAQENELM.FLETSALTGEDVEEAFVQCARKIL** 170*

**4d0gA_Rab14_hu**  5 **YIFKYIIIGDMGVGKSCLLHQFTEKKFMADCPHTIGVEFGTRIIEV.SGQKIKLQIWDTAGLERFRAVTRSYYRGAAGALMVYDITRRSTYNHLSSWLTDARNLTNPNTVIILIGNKADLEAQRDVTYEEAKQFAEENGLL.FLEASAKTGENVEDAFLEAAKKIY** 168

**4rkeA_Rab2_dro**  9 **YLFKYIIIGDTGVGKSCLLLQFTDKRFQPVHDLTIGVEFGARMITI.DGKQIKLQIWDTAGLEAFRSITRSYYRGAAGALLVYDITRRETFNHLTTWLEDARQHSNSNMVIMLIGNKSDLDSRREVKKEEGEAFAREHGLV.FMETSARTAANVEEAFINTAKEIY** 172

**2y8eA_Rab6_dro**  13 **RKFKLVFLGEQSVGKTSLITRFMYDSFDNTYQATIGIDFLSKTMYL.EDRTVRLQLWDTAGQERFRSLIPSYIRDSTVAVVVYDITNTNSFHQTSKWIDDVRTERGSDVIIMLVGNKTDLSDKRQVSTEEGERKAKELNVM.FIETSAKAGYNVKQLFRRVAAALP** 176

**ERG87123.1**  47 **YLLKFLIIGNAGTGKSCIMHQFIERKFKANSAHTIGVEFGSRMVAL.GGKKVKLQIWDTAGQERFRAVTRSYYRGAAGALLVYDITNRESYNALQQWLIEARNLASAHIVVVLVGNKKDLHEERQVMFLEASQFAQENDLT.FMETSALNGENVEETFLKCAKTIL** 210

**Rab14_dicdi**  7 **YIFKYIIIGDMGVGKSCLLHQFTENKFVPDSPHTIGVEFGTRIVDV.NNKKIKLQIWDTAGQERFRAVTRSYYRGAAGALLVYDITRRITYNHLTTWLTDARNLTNPNTVIMLIGNKKDLEGQRDVTYEEASAFAKQNGLI.FVESSAKTGENVEEAFLRTAKLIF** 170

**GAA93714.1**  55 **KKFKLVFLGDQNAGKTSLITQFMYGSFDATYQATIGIDFLSKTMYL.EDRTVRLQLWDTAGQERFRSLIPSYIRDSSVAVVVYDITNRSSFASTNKWIDDVRAERGSDVIIVLVGNKTDLNDKRQVTTEEAEKRAKDLGVM.FIETSAKAGHNVKTLFRKIAQALP** 218

**HO447309.1_EST**  34 **YLFKYIIIGDTGVGKSCLLLQFTDKRFQPVHDLTIGVEFGARMINI.ENKAIKLQIWDTAGQESFRSITRSYYRGAAGALLVYDITRRETFNHLASWLEDARQHANSNMTIMLIGNKSDLQHRRAVSTEEGEQFAKENGLI.FMETSAKTAHNVEEAFINTAGSIY** 197

**XP_002784324.1**  5 **YLFKYIIIGDTGVGKSCLLLQFTDKRFRPDHDLTIGVEFGARLVNV.DGKQIKLQIWDTAGQESFRSITRSYYRGATGALLVYDISRRDTFNHLTRWLEEARQNSNPNMVIMLIGNKCDLE-RREVSYDEGAKFARDNNLI.FLEASAKTAQNVEEAFLQTARKIY** 167

**ETW35364.1**  6 **YLFKYIIIGDTGVGKSCLLLQFTDKRFRADHDLTIGVEFGARLINL.DNKQIKLQIWDTAGQESFRSITRSYYRGAAGALLVYDITRRETFNHLNRWLDEVRQNSNPHMAIILVGNKCDLE-RREVSAEEGAQFARQNGLI.FLETSAKTAKNVEEAFLYTARKIY** 168

**CEM21914.1**  6 **YLFKYIIIGDTGVGKSCLLLQFTDKRFRADHDLTIGVEFGARLINI.DGKQIKLQIWDTAGQESFRSITRSYYRGAAGALLVYDITRRDTFSHLTRWLEEVRQNANPHMTIMLIGNKCDLE-RREVSTEEGASFARQHGLI.FLETSAKTAQNVEEAFIWTARKIY** 168

**XP_008604403.1**  5 **YLFKYIIIGDTGVGKSCLLLQFTDKRFQPVHDLTIGVEFGARMISI.DNKQIKLQIWDTAGQESFRSITRSYYRGAAGALLVYDITRRETFNHLTRWLEEARQNSNSNMAIMLIGNKSDLEHRRAVSYKEGEVFAKENGLI.FLETSAKTAANVEDAFIKTAQKI-** 167

**KFH66701.1**  9 **YIIKYIIIGDTGVGKSCLLLQFTDKRFQPAHDLTIGVEFGARFVTV.AGKQIKLQIWDTAGQESFRSITRSYYRGAAGALLVYDITRRDTFKHLTTWLEDARQHANANTTIMLIGNKSDLEAKRAVSTEEGEAFAKANGLF.FMETSAKTSANVEEAFVETATNIY** 172

**CEP00705.1**  5 **YLFKYIIIGDTGVGKSCLLLQFTDKRFRPVHDLTIGVEFGARMITV.DNRQIKLQIWDTAGQESFRSITRSYYRGAAGALLVYDITRRETFAHLARWLEEARQNGNRNMTIMLIGNKSDLEHRRQISKEEGEAFAKDNGLI.FFETSAKTAANVEEAFIQTAEQIY** 168

**XP_002671605.1**  6 **YIFKYIIIGDMGVGKSCLLHQFTEQKFQSDCPHTIGVEFGTRVIEV.EGKSIKLQIWDTAGQERFRAVTRSYYRGAAGALMVYDITRRNTFHHLASWLTDARNLTNPHTIIVLIGNKSDLEDQRQVSFEEASRFAKENGLV.YVETSAKTGENVTKAFMETASRIY** 169

**XP_005704285.1**  5 **YLFKFIIIGDSGVGKSCLLLQFTDKRFQPIHDLTIGVEFGAKIVQM.DGKTIKLQIWDTAGQESFRSITRSYYRGAAGALLVYDITRRETFNHLATWLEDARQYSSPDMTVVLVGNKCDLEHRRQVSKEEGEAFARAHGLS.FLETSAKTAFHVEETFTEAARTIY** 168

**XP_002182274.1**  5 **YLFKYIIIGDTGVGKSCLLLQFTDKRFQPVHDLTIGVEFGARMISVaDDTQVKLQIWDTAGQESFRSITRSYYRGAAGALLVYDITRRDTFQHLSRWLEEAKSHAQPNMVILLIGNKNDLEHRRAVTAEEGQEFADANGLL.FLETSAKTAFNVEQAFLKTAKSI-** 168

**ESA00154.1**  9 **YIIKYIIIGDTGVGKSCLLLQFTDRRFQPAHDLTIGVEFGARFVDV.EGKKIKLQIWDTAGQESFRSITRSYYRGAAGALLVYDITRRETFDHLLIWLEDARQHSNSNTTIMLIGNKSDLESKRAVSYAEGEAFAQQHGLF.FMETSAKTADNVEESFVDTAKDIY** 172

**ABY63655.1**  6 **YLFKYIIIGDTGVGKSCLLLQFTDKRFRTQHDLTIGVEFGARAVEI.NGETIKLQIWDTAGQESFKSITRSYYRGAAGALLVYDITRRETFNHISTWLDEVRQNGNSDMVIILIGNKSDLDSKRQVSTEEGKSFAKENNLI.FMETSAKNSTNVEDAFLETSKFIY** 169

**XP_010701899.1**  6 **YVFKYIIIGDSGVGKSCLLLQFTDKRFEPLHDLTIGVEFGARLISI.QGRSVKLQIWDTAGQESFRSITRSYYRGASGALLVYDVTRRDTFTHLQSWLEDAKANANTALVIMLIGNKCDLDSKRQVSREEGEAFARSNGLM.FMETSAKTSKNVDDAFLQTAALIY** 169

**XP_002158483.2**  7 **YLFKYLIIGSAGVGKSCILHQFMENKFKQDAHHTIGVEFGSKIVNI.GGKSVKLQIWDTAGQERFRSVTRSYYRGAAGALLVYDITSRETYNTLTTWLTDARTLASPNIVIILLGNKKDLEADREVTFLEASRFAQENELM.FLETSAATGENVEEAFLKCSRSIL** 170

**XP_002115883.1**  8 **FLLKFLIIGSAGTGKSCLLHQFIENKFKQDSSHTIGVEFGSKVINC.SNKTVKLQIWDTAGQERFRSVTKSYYRGAAGALLVYDISNRDSYNALGAWLTDARTLASPQIVIILIGNKKDLESEREVTFLEASRFAQENELM.FLETSALSGENVEEAFLRCSKNIL** 171

**XP_001299604.1**  6 **YIFKFIIVGNSAVGKSCMLLRFDEDRFQPIHDVTVGVTFSIKMVSI.EGQDVKVQVWDTAGQEIFRSITRSYYRDSACAIIVYDITDQSSFAKVEDWIRDVRNLAPPDCLLALVGNKLDLAAQRAVQTSEGQELAEKHNLL.FFETSAATGENVQELFNE------** 163

**XP_002730440.1**  15 **FLFKIVLIGDAGVGKTCVVQRFKSGTFLEKQHSTIGVDFTMKTLHI.DGKKVKLQVWDTAGQERFRTITQSYYRSANGVIIAYDITKRESFNNVPRWVEDVKKYAGANVMQMLIGNKRDLESLREVTVSDAKALAHHHGMLeSLETSAKDSTNVEEAFGKLAKEL-** 178

**foreground (18901):**  **YLYKVVVVGDGGVGKSCITVQYATGTFSDSYDATVGADNKSKTVTV NGETVTFEVWDTAGQEDYDAITSASYRGGDGAVVCYSVTSKSSFNSAEKWRDDVKEHCGENVVVVVVGTKCDLESDRAVSTDDGEAYAESNGCK YYECSAKSGTGVDDAFETAAKAAR**

**L LLLL SA TSLLLRFLKDR PEESIP I VEFFVR LEL EDRPIKLQLL RFRRMRPLF DTHVILLVFDL RPETLENLRR LPQLLRVKDPDIPIMLLAN I RHK VIP EQARKL RKLKLP FM V LENINIKKL LELLRKLL**

**I IIII A II LVENK V D VS F YT I I DNKR I E TL RSY NSQAVII I DRD D IK IKEIRKYAN LII V EQ Q T E KQF KEINIL L T DAE EEV II QI**

**wt_res_freqs (4380): 4318252296247994211112112171115118281511142313 1411311116987977131122111653124221162352117711112711111111112311216819196211717311142117111411 318298512126113811131111**

**1 2133 31 54631551121 121114 5 324111 111 1111236321 453112221 11111356361 1112123211 2113111122124317117 1 111 111 313113 111121 41 1 112161111 11313123**

**1 2112 1 11 12121 1 1 11 1 22 2 2 4131 5 1 14 125 1112122 4 231 1 21 114321121 111 1 11 1 2 4 112 221111 1 4 111 232 21 13**

**insertions 1**

**deletions 653111111111987765443221111111322333211 1 11 11 111122333333433333333211 1 1 1 1 1361221111112 11 112334567789111111112235**

**background (108291):**  **KIRKVGVAGRVGAGKSSTFNAIAG EGAVTDEATVGRTAGTALVEV GGGGRDFTFVDTAGQEDFEGLWEEGAADADGAVVVVDVADGDSAETVAGLDEETKEAILEGKPTVVAATKADKAGARGGAADVREALAELLGAP FFGTSAKTGDGVDDVLDAVADLAE**

**RPPRLLLL LPDV TTLLERLLK LVL PFPEITPDIIIV L L E KKLKLILI LP LLKGRKTL RFLREL LLLLLL ATEPVR QELTE LLQLLKLL LKL LLLLLN M LPD DEL ELLLRE LL P I ILPI LK ENLEEL E LLK LP**

**LLVIVII H NH FI K I KAI GT INVTVQ I I D EDI IVI G IRE AEV YIK V VIIH I SS DI FK IR NI IIIII L LT K EIIK V VI V R I IVE IL**

**wt_res_freqs (27484): 111131218112299521132112 111111113131211111211 1111111112852911221121111111241221546112211111111111111111112141231128191112111111111111111112 111165213142111222111111**

**11111121 1312 465211521 111 111112122111 1 1 1 11112132 15 11111111 113111 112321 311111 11111 11122112 111 122126 1 311 111 111111 11 1 1 1112 11 111123 2 211 22**

**1113132 1 31 12 1 1 111 11 211111 1 1 1 111 112 1 111 111 111 1 1221 1 11 11 11 11 11 13111 1 11 1 2111 1 22 2 1 2 211 11**

**position**  10 . 20 . 30 . 40 . 50 . 60 . 70 . 80 . 90 . 100 . 110 . 120 . 130 . 140 . 150 . 160 . 170

**_**

**_**

**_**

**_**

**_**

**_**

**_**

**_**

**_**

**_ _**

**_ _**

**_ _**

**_ _**

**_ _**

**_ _**

**_ _**

**_ _ _**

**_ _ _**

**_ _ _**

**_ _ _ _**

**_ _ _ _**

**_ _ _ _**

**_ _ _ _ _**

**_ _ _ _ _ _**

**_ _ _ _ _ _ _ _**

**_ _ _ _ _ _ _ _**

**_ _ _ _ _ _ _ _**

**_ _ _ _ _ _ _ _**

**_ _ _ _ _ _ _ _**

**_ _ _ _ _ _ _ _**

**_ _ _ _ _ _ _ _**

**_ _ _ _ _ _ _ _**

**__ _ _ _ _ _ _ _**

**__ _ _ _ _ _ _ _ _**

**__ _ _ _ _ _ _ _ _**

**__ _ _ _ __ _ _ _ _**

**__ _ _ _ _ __ _ __ _ _**

**__ _ _ _ _ __ _ __ _ _**

**__ _ _ _ _ __ _ __ _ _**

**__ _ _ _ _ _ __ _ __ _ _**

**__ _ _ _ _ _ __ _ __ _ _ _ _ _**

**_ __ _ _ _ _ _ ____ __ _ _ _ _ _**

**_ __ _ _ _ _ _ ____ __ _ _ _ _ _**

**_ _ ____ _ _ _ _ ________ ____ _ _ _ _**

**_ _ ____ _ _ _ _ _ ________ ______ _ _ _ _ _**

**_ _ ____ _ _ _ _ _ ________ ______ _ _ _ _ _**

**_ _ ____ _ _ _ _ _ ________ ______ _ _ _ _ _**

**_ _ ____ _ _ _ _ _ ________ ______ _ _ _ _ _**

**_ _ ____ _ _ _ _ _ ________ ______ _ _ _ _ _**

**_ _ ____ _ _ _ _ _ ________ ______ _ _ _ _ _**

**_ _ ____ _ _ _ _ _ ________ ______ _ _ _ _ _**

**Rab subfamily**  ● ● ●●●● ● ● ● ● ● ●●●●●●●● ●●●●●● ● ● ● ● ●

**1z0kA_Rab4_hum**  7 **FLFKFLVIGNAGTGKSCLLHQFIEKKFKDDSNHTIGVEFGSKIINV.GGKYVKLQIWDTAGLERFRSVTRSYYRGAAGALLVYDITSRETYNALTNWLTDARMLASQNIVIILCGNKKDLDADREVTFLEASRFAQENELM.FLETSALTGEDVEEAFVQCARKIL** 170*

**4d0gA_Rab14_hu**  5 **YIFKYIIIGDMGVGKSCLLHQFTEKKFMADCPHTIGVEFGTRIIEV.SGQKIKLQIWDTAGLERFRAVTRSYYRGAAGALMVYDITRRSTYNHLSSWLTDARNLTNPNTVIILIGNKADLEAQRDVTYEEAKQFAEENGLL.FLEASAKTGENVEDAFLEAAKKIY** 168

**4rkeA_Rab2_dro**  9 **YLFKYIIIGDTGVGKSCLLLQFTDKRFQPVHDLTIGVEFGARMITI.DGKQIKLQIWDTAGLEAFRSITRSYYRGAAGALLVYDITRRETFNHLTTWLEDARQHSNSNMVIMLIGNKSDLDSRREVKKEEGEAFAREHGLV.FMETSARTAANVEEAFINTAKEIY** 172

**2y8eA_Rab6_dro**  13 **RKFKLVFLGEQSVGKTSLITRFMYDSFDNTYQATIGIDFLSKTMYL.EDRTVRLQLWDTAGQERFRSLIPSYIRDSTVAVVVYDITNTNSFHQTSKWIDDVRTERGSDVIIMLVGNKTDLSDKRQVSTEEGERKAKELNVM.FIETSAKAGYNVKQLFRRVAAALP** 176

**ERG87123.1**  47 **YLLKFLIIGNAGTGKSCIMHQFIERKFKANSAHTIGVEFGSRMVAL.GGKKVKLQIWDTAGQERFRAVTRSYYRGAAGALLVYDITNRESYNALQQWLIEARNLASAHIVVVLVGNKKDLHEERQVMFLEASQFAQENDLT.FMETSALNGENVEETFLKCAKTIL** 210

**Rab14_dicdi**  7 **YIFKYIIIGDMGVGKSCLLHQFTENKFVPDSPHTIGVEFGTRIVDV.NNKKIKLQIWDTAGQERFRAVTRSYYRGAAGALLVYDITRRITYNHLTTWLTDARNLTNPNTVIMLIGNKKDLEGQRDVTYEEASAFAKQNGLI.FVESSAKTGENVEEAFLRTAKLIF** 170

**GAA93714.1**  55 **KKFKLVFLGDQNAGKTSLITQFMYGSFDATYQATIGIDFLSKTMYL.EDRTVRLQLWDTAGQERFRSLIPSYIRDSSVAVVVYDITNRSSFASTNKWIDDVRAERGSDVIIVLVGNKTDLNDKRQVTTEEAEKRAKDLGVM.FIETSAKAGHNVKTLFRKIAQALP** 218

**HO447309.1_EST**  34 **YLFKYIIIGDTGVGKSCLLLQFTDKRFQPVHDLTIGVEFGARMINI.ENKAIKLQIWDTAGQESFRSITRSYYRGAAGALLVYDITRRETFNHLASWLEDARQHANSNMTIMLIGNKSDLQHRRAVSTEEGEQFAKENGLI.FMETSAKTAHNVEEAFINTAGSIY** 197

**XP_002784324.1**  5 **YLFKYIIIGDTGVGKSCLLLQFTDKRFRPDHDLTIGVEFGARLVNV.DGKQIKLQIWDTAGQESFRSITRSYYRGATGALLVYDISRRDTFNHLTRWLEEARQNSNPNMVIMLIGNKCDLE-RREVSYDEGAKFARDNNLI.FLEASAKTAQNVEEAFLQTARKIY** 167

**ETW35364.1**  6 **YLFKYIIIGDTGVGKSCLLLQFTDKRFRADHDLTIGVEFGARLINL.DNKQIKLQIWDTAGQESFRSITRSYYRGAAGALLVYDITRRETFNHLNRWLDEVRQNSNPHMAIILVGNKCDLE-RREVSAEEGAQFARQNGLI.FLETSAKTAKNVEEAFLYTARKIY** 168

**CEM21914.1**  6 **YLFKYIIIGDTGVGKSCLLLQFTDKRFRADHDLTIGVEFGARLINI.DGKQIKLQIWDTAGQESFRSITRSYYRGAAGALLVYDITRRDTFSHLTRWLEEVRQNANPHMTIMLIGNKCDLE-RREVSTEEGASFARQHGLI.FLETSAKTAQNVEEAFIWTARKIY** 168

**XP_008604403.1**  5 **YLFKYIIIGDTGVGKSCLLLQFTDKRFQPVHDLTIGVEFGARMISI.DNKQIKLQIWDTAGQESFRSITRSYYRGAAGALLVYDITRRETFNHLTRWLEEARQNSNSNMAIMLIGNKSDLEHRRAVSYKEGEVFAKENGLI.FLETSAKTAANVEDAFIKTAQKI-** 167

**KFH66701.1**  9 **YIIKYIIIGDTGVGKSCLLLQFTDKRFQPAHDLTIGVEFGARFVTV.AGKQIKLQIWDTAGQESFRSITRSYYRGAAGALLVYDITRRDTFKHLTTWLEDARQHANANTTIMLIGNKSDLEAKRAVSTEEGEAFAKANGLF.FMETSAKTSANVEEAFVETATNIY** 172

**CEP00705.1**  5 **YLFKYIIIGDTGVGKSCLLLQFTDKRFRPVHDLTIGVEFGARMITV.DNRQIKLQIWDTAGQESFRSITRSYYRGAAGALLVYDITRRETFAHLARWLEEARQNGNRNMTIMLIGNKSDLEHRRQISKEEGEAFAKDNGLI.FFETSAKTAANVEEAFIQTAEQIY** 168

**XP_002671605.1**  6 **YIFKYIIIGDMGVGKSCLLHQFTEQKFQSDCPHTIGVEFGTRVIEV.EGKSIKLQIWDTAGQERFRAVTRSYYRGAAGALMVYDITRRNTFHHLASWLTDARNLTNPHTIIVLIGNKSDLEDQRQVSFEEASRFAKENGLV.YVETSAKTGENVTKAFMETASRIY** 169

**XP_005704285.1**  5 **YLFKFIIIGDSGVGKSCLLLQFTDKRFQPIHDLTIGVEFGAKIVQM.DGKTIKLQIWDTAGQESFRSITRSYYRGAAGALLVYDITRRETFNHLATWLEDARQYSSPDMTVVLVGNKCDLEHRRQVSKEEGEAFARAHGLS.FLETSAKTAFHVEETFTEAARTIY** 168

**XP_002182274.1**  5 **YLFKYIIIGDTGVGKSCLLLQFTDKRFQPVHDLTIGVEFGARMISVaDDTQVKLQIWDTAGQESFRSITRSYYRGAAGALLVYDITRRDTFQHLSRWLEEAKSHAQPNMVILLIGNKNDLEHRRAVTAEEGQEFADANGLL.FLETSAKTAFNVEQAFLKTAKSI-** 168

**ESA00154.1**  9 **YIIKYIIIGDTGVGKSCLLLQFTDRRFQPAHDLTIGVEFGARFVDV.EGKKIKLQIWDTAGQESFRSITRSYYRGAAGALLVYDITRRETFDHLLIWLEDARQHSNSNTTIMLIGNKSDLESKRAVSYAEGEAFAQQHGLF.FMETSAKTADNVEESFVDTAKDIY** 172

**ABY63655.1**  6 **YLFKYIIIGDTGVGKSCLLLQFTDKRFRTQHDLTIGVEFGARAVEI.NGETIKLQIWDTAGQESFKSITRSYYRGAAGALLVYDITRRETFNHISTWLDEVRQNGNSDMVIILIGNKSDLDSKRQVSTEEGKSFAKENNLI.FMETSAKNSTNVEDAFLETSKFIY** 169

**XP_010701899.1**  6 **YVFKYIIIGDSGVGKSCLLLQFTDKRFEPLHDLTIGVEFGARLISI.QGRSVKLQIWDTAGQESFRSITRSYYRGASGALLVYDVTRRDTFTHLQSWLEDAKANANTALVIMLIGNKCDLDSKRQVSREEGEAFARSNGLM.FMETSAKTSKNVDDAFLQTAALIY** 169

**XP_002158483.2**  7 **YLFKYLIIGSAGVGKSCILHQFMENKFKQDAHHTIGVEFGSKIVNI.GGKSVKLQIWDTAGQERFRSVTRSYYRGAAGALLVYDITSRETYNTLTTWLTDARTLASPNIVIILLGNKKDLEADREVTFLEASRFAQENELM.FLETSAATGENVEEAFLKCSRSIL** 170

**XP_002115883.1**  8 **FLLKFLIIGSAGTGKSCLLHQFIENKFKQDSSHTIGVEFGSKVINC.SNKTVKLQIWDTAGQERFRSVTKSYYRGAAGALLVYDISNRDSYNALGAWLTDARTLASPQIVIILIGNKKDLESEREVTFLEASRFAQENELM.FLETSALSGENVEEAFLRCSKNIL** 171

**XP_001299604.1**  6 **YIFKFIIVGNSAVGKSCMLLRFDEDRFQPIHDVTVGVTFSIKMVSI.EGQDVKVQVWDTAGQEIFRSITRSYYRDSACAIIVYDITDQSSFAKVEDWIRDVRNLAPPDCLLALVGNKLDLAAQRAVQTSEGQELAEKHNLL.FFETSAATGENVQELFNE------** 163

**XP_002730440.1**  15 **FLFKIVLIGDAGVGKTCVVQRFKSGTFLEKQHSTIGVDFTMKTLHI.DGKKVKLQVWDTAGQERFRTITQSYYRSANGVIIAYDITKRESFNNVPRWVEDVKKYAGANVMQMLIGNKRDLESLREVTVSDAKALAHHHGMLeSLETSAKDSTNVEEAFGKLAKEL-** 178

**foreground (7002):**  **YLFKVVVVGDSGVGKSCILSQYADGSFSESSKATVGVDFGSKSVTV DGKTVKAQIWDTAGQERYRAVTSAYYRGAAGCVVVYDVSSESSFNSVSSWIDDADTHAGSNVVKVLVGNKCDLASERAVSTEDGEAWCDENGVL YFETSAKSGTNVDEAFETVATDAY**

**F L LLLL S TNLMLRFVRKR DPEFIP I IE LIRTLQL EDRRI L L F TI RSF SM ILL F ITRRKT EHLRR LKELRQYSDPDIPIM L I EHL V P EAKKLARKLNMP FM LENI IEQ LEMLRKLL**

**IIII A S TEDK NLDY S KV IEI K SL T V VII KKQ DNIKK E IEE NE VL I V DEK Q T QQF K Y L L DSE LVKEI**

**wt_res_freqs (1340): 7769231197769995316112131182112229196691151313 4552471869999999827217338897817111889411116911311911111135113332285999296111828324142211131511 129799613288136911151112**

**1 1 2352 1 41712661111 111111 8 23 1134212 11113 6 2 7 35 141 11 246 1 472313 21312 516231212222123 1 1 211 1 1 452128111112 71 2111 141 11112113**

**2225 1 3 2121 1112 3 21 322 1 42 1 1 121 111 24211 1 211 11 11 2 1 112 1 1 113 1 1 3 2 111 31125**

**insertions 1 2 1 2 1 1**

**deletions 4321111111111199876543211111 311111111111 1111 1 1 1 1 631111111111 1 124556789111111111112235**

**background (11899):**  **KSYKCVVVGSSGVGKSCIIVQYTTGEFVDDYDSTVGVDFYSKTVTV NGETYKFQVWDTAGQERYRAITSQYYRGANGAVVCYDVTSKASYDSAEKWREDVKEHAGENVVVVVVGTKCDLESDREVSTDDGEAYAESIGVK YYECSAKTGEGVNDAFDDAAKAAR**

**PRIRLLLL DGA TSLLLRLLKDR PEE IP L AESFV QLVL DDKPITLDLL DDFSSMRPMSIPDSHVILLVFSLDDPETLEELK LLPQLLHVKDPDIPLLLIAN I REQ PIT EQARKL RKLKLP FL V SLEN NIKKL EELLRKLL**

**IQF IIII A TI FIEN I V I F N R IEI NQEVE EI K DRLADL KNTQAVII I NRD F NIQ IKEIRRFCN III V DE V E KQF KQ NC I T EEI II QI**

**wt_res_freqs (3040): 1128153391147994111113123171116118281423141313 1411111115976866241111114532413221254342117111112611111111112211316819197211717311141117111411 418297532126111811221111**

**11212134 532 54621411111 222 15 1 11111 1111 4111215131 113122431211111213454411211222122 11113111112126117116 1 111 122 313113 111111 31 1 1112 52111 12313123**

**112 2111 2 11 4112 1 1 4 1 1 1 212 11131 24 1 115112 11112122 4 231 6 311 114322121 321 1 11 1 4 111 21 12 1 4 131 21 13**

**position**  10 . 20 . 30 . 40 . 50 . 60 . 70 . 80 . 90 . 100 . 110 . 120 . 130 . 140 . 150 . 160 . 170

**Figure 2— Source data 1. P-loop GTPase superfamily and R^4^ family for Rho1.**

**Amoebozoa**  19 **KALKIVVVGDGAVGKTCLLLAFSKGEIPTAYVPTV-FENFSHVMKY.KNEEFILHLWDTAGQEEYDRLRPLSYADSDVVLLCFAVNNRTSFDNIStKWEPEIKHYI-DTAKTVLVGLKVDL---Rkdgsd..............DVTKQEGDDLCQKLGcVAYIEASSVAKIG** 171

**Chordata**  34 **-SVKVVLVGDGGCGKTSLLMVFADGAFPESYTPTV-FERYMVNLQV.KGKPVHLHIWDTAGQDDYDRLRPLFYPDASVLLLCFDVTSPNSFDNIFnRWYPEVNHFC-KKVPIIVVGCKTDL---Rkdkslvnklrrngle....PVTYHRGQEMARSVGaVAYLECSARLHDN** 195

**Amoebozoa**  8 **-TVKVVVVGDGAVGKTSLLILYTTKAFPKDYVPTV-FDNFNCLEMY.DNKPVNLVLWDTAGQEDYDNLRPLSYPQTDVFIICYSVVKRDSLDNIKyKWLPEINQTN-QGTPIILVGTKTDL---Redkktlsqlqeskqe....PVSRDEGVALAKEIGaVQFFECSALTGNG** 169

**Platyhelminthe**  6 **--RKIVVVGDGMVGKTALLSAFVNGAFQDCYIPTV-FETSAKEVDLpDGRHLTLGLWDTGGQEEFDQIRQLAYPGASLILLCYAVDCPTSLENIVhTWLDEVKCYC-PQIPLILVGCKADK---RvviapgksnnltmnasqtaLIDPNDVEKVSKQIGaQIVIECSALTRSN** 171

**Ascomycota**  71 **YHLKIVVVGDGAVGKTCLLISYVQGTFPTDYIPTI-FENYVTNIEGpNGQIIELALWDTAGQEEYSRLRPLSYTNADVLMVCYSVGSKTSLRNVEdLWFPEVKHFC-PSTPIMLVGLKSDL---Yeadnlsd............LVEPSSAESLARRLGaFAHIQCSARLKEN** 226

**Streptophyta**  8 **KFIKCVTVGDGAVGKTCMLICYTSNKFPTDYIPTV-FDNFSANVAV.DGQIVNLGLWDTAGQEDYSRLRPLSYRGADIFVLAFSLISKASYENVLkKWMPELRRFAP-NVPIVLVGTKLDL---Rddkgyladhtn........VITSTQGEELRKQIGaAAYIECSSKTQQN** 166

**Nematoda**  2 **QAIKCVVVGDGAVGKTCLLLSYTTNAFPGEYILTV-FDTYSTNVMV.DGRPINLSLWDTAGQDDYDQFRHLSFPQTDVFLVCFALNNPASFENVRaKWYPEVSHHC-PNTPIILVGTKADL---Redrdtierlrerrlq....PVSHTQGYVMAKEIKaVKYLECSALTQIG** 164

**position** 20 . 30 . 40 . 50 . 60 . 70 . 80 . 90 . 100 . 110 . 120 . 130 . 140 . 150 . 160 . 170

**_**

**_**

**__**

**__**

**__**

**__**

**__**

**___ _ _**

**___ _ _ _**

**___ _ _ _**

**___ _ _ _**

**_ ___ _ _ _**

**_ ___ _ _ _**

**_ ___ _ _ _**

**_ ___ _ _ _**

**_ ___ _ _ _ _**

**_ ___ _ _ __ _**

**_ ___ _ _ __ _**

**_ ___ _ _ __ _**

**_ ___ _ _ __ _**

**_ ___ _ _ __ _**

**_ ___ _ _ __ _**

**_ ___ _ _ __ _**

**_ ___ _ _ __ _**

**_ ____ _ _ __ _**

**_ ____ _ _ __ _**

**_ ____ _ _ __ _**

**_ ____ _ _ __ _**

**_ ____ _ _ __ _**

**_ ____ _ _ __ _**

**_ ____ _ _ __ _**

**_ _ ____ _ _ _ __ _**

**_ _ ____ _ _ _ _ __ _**

**_ _ ____ _ _ _ _ _ __ _**

**_ _ _ _ ____ _ _ _ _ _ __ _**

**_ _ _ _ ____ _ _ _ __ _ _ __ _ _**

**_ _ _ _ ____ _ _ _ __ _ _ __ _ _**

**_ ___ _ ____ _ _ _ __ _ __ __ _ _**

**_ ___ _ ____ _ _ _ _ __ _ __ __ _ _**

**_ ___ _ _____ _ _ _ _ __ _ __ __ _ _**

**_ ___ _ _____ _ _ _ _ __ _ __ __ _ _**

**_ ___ _ _____ _ _ _ _ __ _ __ _ __ _ _**

**_ ___ _ _____ _ _ _ _ __ _ __ _ __ _ _**

**_ ___ _ _____ _ _ _ _ __ _ __ __ __ _ _**

**_ ___ _ _____ _ _ _ _ __ _ __ ___ __ _ _**

**_ ___ _ _____ _ _ _ _ __ _ __ ___ __ _ _**

**_ ___ _ _____ _ _ _ ____ _ __ ___ __ _ __**

**_ ___ _ _____ _ _ _ ____ ______ ___ __ _ __**

**_ ___ _ ______ _ _ _ ____ ______ ___ __ _ _ __**

**_ ___ _ ______ _ _ _ ____ ______ ___ __ _ _ __ _**

**_ ___ _ ______ _ _ _ ____ ______ ___ __ _ _ __ _**

**P-loop GTPases**  ● ●●● ● ●●●●●● ● ● ● ●●●● ●●●●●● ●●● ●● ● ● ●● ●

**3refB_Rho1_ent**  19 **KALKIVVVGDGAVGKTCLLLAFSKGEIPTAYVPTV-FENFSHVMKY.KNEEFILHLWDTAGQEEYDRLRPLSYADSDVVLLCFAVNNRTSFDNIStKWEPEIKHYI-DTAKTVLVGLKVDL---Rkdgsd..............DVTKQEGDDLCQKLGcVAYIEASSVAKIG** 171

**2j1lA_RhoD_hum**  34 **-SVKVVLVGDGGCGKTSLLMVFADGAFPESYTPTV-FERYMVNLQV.KGKPVHLHIWDTAGQDDYDRLRPLFYPDASVLLLCFDVTSPNSFDNIFnRWYPEVNHFC-KKVPIIVVGCKTDL---Rkdkslvnklrrngle....PVTYHRGQEMARSVGaVAYLECSARLHDN** 195

**RACD_DICDI**  8 **-TVKVVVVGDGAVGKTSLLILYTTKAFPKDYVPTV-FDNFNCLEMY.DNKPVNLVLWDTAGQEDYDNLRPLSYPQTDVFIICYSVVKRDSLDNIKyKWLPEINQTN-QGTPIILVGTKTDL---Redkktlsqlqeskqe....PVSRDEGVALAKEIGaVQFFECSALTGNG** 169

**AAN77581.1**  6 **--RKIVVVGDGMVGKTALLSAFVNGAFQDCYIPTV-FETSAKEVDLpDGRHLTLGLWDTGGQEEFDQIRQLAYPGASLILLCYAVDCPTSLENIVhTWLDEVKCYC-PQIPLILVGCKADK---RvviapgksnnltmnasqtaLIDPNDVEKVSKQIGaQIVIECSALTRSN** 171

**EDN59958.1**  71 **YHLKIVVVGDGAVGKTCLLISYVQGTFPTDYIPTI-FENYVTNIEGpNGQIIELALWDTAGQEEYSRLRPLSYTNADVLMVCYSVGSKTSLRNVEdLWFPEVKHFC-PSTPIMLVGLKSDL---Yeadnlsd............LVEPSSAESLARRLGaFAHIQCSARLKEN** 226

**2J0V|A**  8 **KFIKCVTVGDGAVGKTCMLICYTSNKFPTDYIPTV-FDNFSANVAV.DGQIVNLGLWDTAGQEDYSRLRPLSYRGADIFVLAFSLISKASYENVLkKWMPELRRFAP-NVPIVLVGTKLDL---Rddkgyladhtn........VITSTQGEELRKQIGaAAYIECSSKTQQN** 166

**RAC2_CAEEL**  2 **QAIKCVVVGDGAVGKTCLLLSYTTNAFPGEYILTV-FDTYSTNVMV.DGRPINLSLWDTAGQDDYDQFRHLSFPQTDVFLVCFALNNPASFENVRaKWYPEVSHHC-PNTPIILVGTKADL---Redrdtierlrerrlq....PVSHTQGYVMAKEIKaVKYLECSALTQIG** 164

**foreground (127234):**  **KIRNVGVVGDSGAGKSSLFNAFAG EGAVTDEAGTGRTATSATVEV GGGGVDFTFWDTAGQSDYRGEREEGAAGADGAVFVYDASDGDSAETVA GWDEEVKEAALEGKPVVVAGTKADKADAD GGAADEAEAFAEELG APYFETSAKTGDG**

**RPPRLLLL LPDV TT L RLLK LVL PTITPDI IKLL L E KKIKLVLI LP LRRF KLL RFLREV LLLLLLSITEPETLQELK KLLKQLLKLL LKL LLLLLN M LPE R EVSELLLREL LLPN L ILPI L EN**

**LLKIVII HVNS I KII KFI V INV VQ I I D ED INIV G HEK S YIKD VIIV I V R F DIT E F IR HI DI IIIII L L E EIIK KK I VI V**

**wt_res_freqs (34526): 111131239113299526132112 111111111131211111211 111111111185291111111111111134222151631221211111 11112111111121412211281911121 111111111121112 1211116522314**

**11111121 1213 45 2 2421 111 1312121 1111 1 1 11112132 14 1112 121 112211 113311112111111111 1111122111 111 123126 1 311 2 1111111112 1111 1 1111 1 12**

**1123122 1131 1 111 111 1 211 11 1 1 1 11 1122 1 121 1 1111 1221 1 1 1 1 111 1 1 11 11 11 12111 1 1 1 2111 11 1 22 2**

**insertions 15 14141172 1 3 1 23 155 395553 21 11217121 1 64 516 1241 2 4 2 14 1 7 16852117 331594 57 2 143811 144 412**

**deletions 421965433222211111 311 1 14 1 651177 55356 632114 3 1 8 7 333 11 71132212221 134919161221777 21888 111111117516111 8117 112223**

**position** 20 . 30 . 40 . 50 . 60 . 70 . 80 . 90 . 100 . 110 . 120 . 130 . 140 . 150 . 160 . 170

**_**

**_**

**_ _**

**_ _**

**_ _**

**_ _ _**

**_ _ _**

**_ _ _**

**_ _ _**

**_ _ _**

**_ _ _ _ _**

**_ _ _ _ _**

**_ _ _ _ _**

**_ _ _ _ _**

**_ _ __ _ _**

**_ _ __ _ _**

**_ _ __ _ _**

**_ _ _ __ _ _**

**_ _ _ __ _ _ _**

**_ _ _ __ _ _ _**

**_ _ _ __ _ _ _ _**

**_ _ _ __ _ _ _ _**

**_ _ _ __ _ _ _ _**

**_ _ _ __ _ _ _ _ _**

**_ _ _ __ _ _ _ _ _**

**_ _ _ _ __ _ _ _ _ _**

**_ _ _ _ __ _ _ _ _ _ _**

**_ _ _ _ __ _ _ _ _ _ _**

**_ _ _ _ __ _ _ _ _ _ _**

**_ _ _ _ __ _ _ _ _ _ _ _ _**

**_ _ _ _ __ _ _ _ _ _ _ _ _**

**_ _ _ _ _ __ _ _ _ _ _ _ _ _**

**_ _ __ _ _ __ _ _ _ _ _ _ _ _**

**_ _ __ _ _ __ _ _ _ _ _ _ _ _**

**_ _ _ _ __ _ _ __ _ _ _ _ _ _ _ _**

**_ _ _ _ _ __ _ _ __ _ _ _ _ _ _ _ _**

**_ _ _ _ _ __ _ _ __ _ _ _ _ _ _ _ _**

**_ _ _ _ _ __ _ _ _ __ _ _ _ _ _ _ _ _**

**_ _ _ _ _ __ _ _ _ _ __ _ _ _ _ _ _ _ _**

**_ _ _ _ _ __ _ _ _ _ __ _ _ _ _ _ _ _ _**

**_ _ _ _ _ __ _ _ _ _ __ _ _ _ _ _ _ _ _**

**_ _ _ _ _ _ _ __ _ _ _ _ __ _ _ _ _ _ _ _ _ _**

**_ _ _ _ _ _ _ _ __ _ _ _ _ __ _ _ _ _ _ _ _ _ _**

**_ _ _ _ _ _ _ _ __ _ _ _ _ __ _ _ _ _ _ _ _ _ _**

**_ _ _ _ _ __ _ _ __ _ _ _ _ _ __ _ _ _ _ _ _ _ _ _**

**_ _ _ _ _ _ __ _ _ __ _ _ _ _ _ _ __ _ _ _ _ _ _ _ _ _ _**

**_ _ _ _ _ _ __ _ _ __ _ _ _ _ _ _ __ _ _ _ _ _ _ _ _ _ _**

**_ _ _ _ _ _ __ _ _ __ _ _ _ _ _ _ __ _ _ _ _ _ _ _ _ _ _ _**

**_ _ _ _ _ _ __ _ _ __ _ _ _ _ _ _ __ _ _ _ _ _ _ _ __ _ _ _ _**

**_ _ _ _ _ _ _ __ _ _ __ _ _ _ _ _ _ __ _ _ _ _ _ _ _ __ _ _ _ _**

**_ _ _ _ _ _ _ __ _ _ __ _ _ _ _ _ _ __ _ _ _ _ _ _ _ __ _ _ _ _**

**R^4^ family**  ● ● ● ● ● ● ● ●● ● ● ●● ● ● ● ● ● ● ●● ● ● ● ● ● ● ● ●● ● ● ● ●

**3refB_Rho1_ent**  19 **KALKIVVVGDGAVGKTCLLLAFSKGEIPTAYVPTV-FENFSHVMKY.KNEEFILHLWDTAGQEEYDRLRPLSYADSDVVLLCFAVNNRTSFDNIStKWEPEIKHYI-DTAKTVLVGLKVDL---Rkdgsd..............DVTKQEGDDLCQKLGcVAYIEASSVAKIG** 171

**2j1lA_RhoD_hum**  34 **-SVKVVLVGDGGCGKTSLLMVFADGAFPESYTPTV-FERYMVNLQV.KGKPVHLHIWDTAGQDDYDRLRPLFYPDASVLLLCFDVTSPNSFDNIFnRWYPEVNHFC-KKVPIIVVGCKTDL---Rkdkslvnklrrngle....PVTYHRGQEMARSVGaVAYLECSARLHDN** 195

**RACD_DICDI**  8 **-TVKVVVVGDGAVGKTSLLILYTTKAFPKDYVPTV-FDNFNCLEMY.DNKPVNLVLWDTAGQEDYDNLRPLSYPQTDVFIICYSVVKRDSLDNIKyKWLPEINQTN-QGTPIILVGTKTDL---Redkktlsqlqeskqe....PVSRDEGVALAKEIGaVQFFECSALTGNG** 169

**AAN77581.1**  6 **--RKIVVVGDGMVGKTALLSAFVNGAFQDCYIPTV-FETSAKEVDLpDGRHLTLGLWDTGGQEEFDQIRQLAYPGASLILLCYAVDCPTSLENIVhTWLDEVKCYC-PQIPLILVGCKADK---RvviapgksnnltmnasqtaLIDPNDVEKVSKQIGaQIVIECSALTRSN** 171

**EDN59958.1**  71 **YHLKIVVVGDGAVGKTCLLISYVQGTFPTDYIPTI-FENYVTNIEGpNGQIIELALWDTAGQEEYSRLRPLSYTNADVLMVCYSVGSKTSLRNVEdLWFPEVKHFC-PSTPIMLVGLKSDL---Yeadnlsd............LVEPSSAESLARRLGaFAHIQCSARLKEN** 226

**2J0V|A**  8 **KFIKCVTVGDGAVGKTCMLICYTSNKFPTDYIPTV-FDNFSANVAV.DGQIVNLGLWDTAGQEDYSRLRPLSYRGADIFVLAFSLISKASYENVLkKWMPELRRFAP-NVPIVLVGTKLDL---Rddkgyladhtn........VITSTQGEELRKQIGaAAYIECSSKTQQN** 166

**RAC2_CAEEL**  2 **QAIKCVVVGDGAVGKTCLLLSYTTNAFPGEYILTV-FDTYSTNVMV.DGRPINLSLWDTAGQDDYDQFRHLSFPQTDVFLVCFALNNPASFENVRaKWYPEVSHHC-PNTPIILVGTKADL---Redrdtierlrerrlq....PVSHTQGYVMAKEIKaVKYLECSALTQIG** 164

**foreground (18423):**  **YEYKVVVVGDGGVGKSCITVQYATGTFSDSYDATVGADNKSKTVTV NGETVKFEVWDTAGQEDYDAITSASYRGGDGAVVCYSVSSKSSYNSAE KWVDDVKEHCGSNVVKVIVGTKCDLASER AVSTDDGEAYAESNG CKYYECSAKSGTG**

**LL LLLL SS TSLLLRFLKDR PEE IP I IEFLVR LEL EDRKI LQLL RFRRMRPLF DTHVILLVFDLTRPQTLENLR R LPQLLRVADPDIPIMLLAN I EHK VIT EQARKL RKLN LPFM V LENEN**

**I IIII A A II LVENK D VS V YT I I DNK I E TL RSY NSQAVII I DRE FD IK IKEIR Y NE VLVI V EQ Q E KQF KEI IL L T DA**

**wt_res_freqs (4994): 3118242296247994211112112171115118281411142313 141133111698787713112211165312421116231211711111 27111111211123112168191961118 173211421171114 1131819851312**

**31 2133 31 53631551111 121 13 5 124111 111 11112 6321 443112211 111113463615111212321 1 2113111212124317117 1 211 111 313113 1111 2141 1 11115**

**1 2112 1 1 11 12121 1 12 3 22 2 2 413 5 1 14 125 1112122 4 231 71 21 11432 1 11 1111 1 11 1 4 112 221 11 1 4 11**

**insertions 1 3 3214 1 1 2 1115772 141331 2 4 11 221 1**

**deletions 532111111198876554332111 111121112211 11 111111111111111 12121 1 11112 11 2 112234556**

**background (108811):**  **KIRKVGVAGRVGVGKSSTFNAIAG EGAVTDEATVGRTAGTALVEV GGGGRDFTFVDTAGQEDFEGLWEEGAADADGAVVVVDVADGDSAETVA GLDEEVKEAILEGKPVVIAATKADKAGAR GGAADVREALAELLG APFFGTSAKTGDG**

**RPPVLLLL LPDA TTLLERLLK LVL PFPEITPDIIIV L L E KKIKLILI LP LLK RKTL RFLREL LLLLLL ATEPVR QELT E LLQLLKLL LKL LLLLLN M LPD D EL ELLLRE L P I ILPI LK EN**

**LLRIVII H NH FI K I KAI GT INVTVQ I I D EDV IVI G IRE AEV YIK V VIIH I SS R DI FK IR DI IIVII L LT K EIIK V VI V R**

**wt_res_freqs (29532): 111131218112299521132112 111111112131211111211 111111111285291122112111111124122154611221111111 11111111111121412111281911121 111111111111111 1211116521314**

**11111121 1312 465211521 111 111112122111 1 1 1 11112132 15 111 1111 113111 113321 311111 1111 1 11122112 111 122126 1 311 1 11 111111 1 1 1 1112 11 11**

**1113132 1 31 11 1 1 111 11 211111 1 1 1 111 112 1 111 111 111 1 1221 1 11 1 11 11 11 11 13211 1 11 1 2111 1 22 2 1**

**position** 20 . 30 . 40 . 50 . 60 . 70 . 80 . 90 . 100 . 110 . 120 . 130 . 140 . 150 . 160 . 170

**Amoebozoa**  172 **LNEVFEKSVDCIF** 184

**Chordata**  196 **VHAVFQEAAEVAL** 208

**Amoebozoa**  170 **VNDIFAAAIKAA-** 181

**Platyhelminthe**  172 **VNSVFELAARIIL** 184

**Ascomycota**  227 **IDEVFETAIHTLL** 239

**Streptophyta**  167 **VKAVFDTAIKVVL** 179

**Nematoda**  165 **LKQVFDEAIRTGL** 177

**position**  180

**_**

**_**

**P-loop GTPases**  ●

**3refB_Rho1_ent**  172 **LNEVFEKSVDCIF** 184

**2j1lA_RhoD_hum**  196 **VHAVFQEAAEVAL** 208

**RACD_DICDI**  170 **VNDIFAAAIKAA-** 181

**AAN77581.1**  172 **VNSVFELAARIIL** 184

**EDN59958.1**  227 **IDEVFETAIHTLL** 239

**2J0V|A**  167 **VKAVFDTAIKVVL** 179

**RAC2_CAEEL**  165 **LKQVFDEAIRTGL** 177

**foreground (127234):**  **VDDVFDAVAELAL**

**LEELLE LLR LP**

**I IVK I**

**wt_res_freqs (34526): 2111111111112**

**112322 211 22**

**1 211 1**

**insertions**

**deletions 3444767711124**

**position**  180

**_**

**_**

**_**

**_**

**_**

**_**

**_**

**_**

**_**

**_**

**_**

**_**

**_**

**_**

**_**

**_**

**_**

**_**

**_**

**_**

**_**

**_**

**_**

**_**

**_**

**_**

**_**

**_**

**_**

**R^4^ family**  ●

**3refB_Rho1_ent**  172 **LNEVFEKSVDCIF** 184

**2j1lA_RhoD_hum**  196 **VHAVFQEAAEVAL** 208

**RACD_DICDI**  170 **VNDIFAAAIKAA-** 181

**AAN77581.1**  172 **VNSVFELAARIIL** 184

**EDN59958.1**  227 **IDEVFETAIHTLL** 239

**2J0V|A**  167 **VKAVFDTAIKVVL** 179

**RAC2_CAEEL**  165 **LKQVFDEAIRTGL** 177

**foreground (18423):**  **VDDAFETAAKAAR**

**IKKL LELLRKLL**

**EEV II QI**

**wt_res_freqs (4994): 6113811131111**

**1111 11313113**

**232 21 13**

**insertions**

**deletions 7889111111224**

**background (108811):**  **VDDVLDAVADLAE**

**LEEL E LLK LP**

**I IVE IL**

**wt_res_freqs (29532): 2111222111111**

**1123 2 211 22**

**2 211 11**

**position**  180
