# Supplementary material for: Inferring joint sequence-structural determinants of protein functional specificity
Source: eLife. 2018 Jan 16;7:e29880. doi: 10.7554/eLife.29880 (PMC5770160; doi:10.7554/eLife.29880)
Supplement: Figure 3—source data 1. [file elife-29880-fig3-data1.docx]

**Figure 3— Source data 1. GTPases: TF family, EFTu/CysN subfamily and EFTu sub-subfamily (1OB5)**

**Deinococcus**  10 **PHVNVGTIGHVDHGKTTLTAALTYVAAAE.NPnV.EVKDYGDIDKAPEERARGITINTAHVEYETAKRHYSHVDCPGHADYIKNMITGAAQMDGAILVVSAADGPMPQTREHILLARQVGVPYIVVFMNKVDMVDDPELLDLVEMEVRDLLNQYEFPGDEVPVIRGSALLALE** 180*

**Proteobacteria**  10 **PHVNVGTIGHVDHGKTTLTAAITTVLAKT.YG.G.AARAFDQIDNAPEEKARGITINTSHVEYDTPTRHYAHVDCPGHADYVKNMITGAAQMDGAILVVAATDGPMPQTREHILLGRQVGVPYIIVFLNKCDMVDDEELLELVEMEVRELLSQYDFPGDDTPIVRGSALKALE** 179

**Chordata**  13 **PHVNVGTIGHVDHGKTTLTAAITKILAEG.GG.A.KFKKYEEIDNAPEERARGITINAAHVEYSTAARHYAHTDCPGHADYVKNXITGTAPLDGCILVVAANDGPXPQTREHLLLARQIGVEHVVVYVNKADAVQDSEXVELVELEIRELLTEFGYKGEETPIIVGSALCALE** 182

**Bacteroidetes**  11 **PHVNVGTIGHVDHGKTTLTAAITKVLAERvGG.A.AEQTFEAIDNAPEERERGITIATSHVEYETENRHYAHVDCPGHADYVKNMVTGAAQMDGAILVVGSDDGPMPQTREHILLARQVGVPYLVVFMNKTDLVDDAELLELVEMEVRELLTEYEFPGDEVPVVRGSALQALE** 181

**Chlorophyta**  12 **-HVNIGTIGHVDHGKTTLTAAITMCLQSF.SK.N.KGKRYDEIDSAPEEKARGITINTAHVEYETENRHYAHVDCPGHADYVKNMITGAAQMDGAILVVSGADGPMPQTKEHLLLAKQVGVPTLVVFLNKEDQVDDPELLELVELEVRETLDKYEYPGDDIPIIAGSALLALE** 180

**Chloroflexi**  11 **PHCNVGTIGHVDHGKTTLTAAITRTLSTK.GW.A.DFRAYDQIDNAPEEKARGLTIAISHIEYQTETRHYAHIDCPGHADYIKNMITGAAQMDGAILVVSAPDGPMPQTREHVLLIHQVEVPAVVVALNKCDMMDDEELLELVELEVRELLTKNSFPGDEIPIVRVSAIKALE** 180

**Placozoa**  25 **PHINIGTIGHVDHGKTSLTAAITKLLQER.GQ.A.KYKAYDEIDNAPEEKARGITIKTANVEYETDQRHYGHIDCPGHADYIKNMITGAARMDGAILVVAATDGAMPQTKEHVLLAKQIGVKHMVVYVNKADTIDDNEMLELVELEIRDLLQEHGYD-EDTPVIIGSALCALE** 193

**Cnidaria**  59 **PHINIGTIGHVDHGKTTLTAAITKVLSEK.GG.S.KFKDYADIDNAPEERARGITINASHVEYETDTRHYGHIDCPGHADYIKNMITGAAQMDGAILVVAATDGQMPQTREHLLLANQIGVKNLCVFINKADMVDDKEIMDLVEMEIRELLTEYGYDGDNTPVIGGSALCALE** 228

**Phaeophyceae**  11 **PHINIGTIGHVDHGKTTLTAAITAVLALA.GD.A.NAKKYEDIDAAPEERARGITINTAHVEYETATRHYAHVDCPGHADYVKNMITGAAQMDGAILVVSAADGPMPQTREHILLSKQVGVPHIVVFLNKEDQVDDLELVELVELEVRELLSNYDFPGDDIPIVTGSALQALD** 180

**Acidobacteria**  11 **PHVNIGTIGHVDHGKTTLTAAITFILAKK.FG.G.ETKSYDQIDSAPEEKARGITINTAHVEYQTEKRHYAHVDCPGHADYVKNMITGAAQMDGAILVVAATDGPMPQTREHILLARQVGVPYIVVFMNKIDIA-DPELAELVEMEIRDLLSSYQFPGDETPIIKGSARLALD** 179

**Thermodesulfob**  11 **PHLNVGTIGHIDHGKTTLTSAITRVLSTK.GY.A.QWIPFDQIDKAPEEKARGITIQLAHVEYESDKRHYAHIDCPGHADYIKNMITGAAQMDGSILVVAATDGPMPQTREHVLLARQVNVPAMVVFMNKVDMVDDAELLDLVELEVRELLSKYGFPGDEVPVIRGSALKALE** 180

**Mortierellomyc**  78 **PHVNIGTIGHVDHGKTTLTAAITKTLASR.GG.A.EFKDYNQIDKAPEEKARGITISTAHVEYETENRHYAHVDCPGHADYIKNMITGAAQMDGAIIVVAATDGQMPQTREHLLLAKQVGIQKLVVFINKVDAVDDPEMLELVDMEMRDVLTTYGFDGENTPIIMGSALCALE** 247

**Fibrobacteres**  11 **PHCNIGTIGHVDHGKTTLTAAICTTLAAK.GL.A.AAKRFDEIDNAPEEKARGITINTSHVEYTTANRHYAHVDCPGHADYVKNMVTGAAQMDGAILVVAATDGPMPQTREHILLAHQVGVPKIVVFMNKCDMVDDAEILDLVEMEVRELLSKYDFDGDNTPIIRGSALKALE** 180

**Jakobida**  11 **PHCNIGTIGHVDHGKTTLTAAITKVLSET.GG.A.VFTDYDQIDKAPEEKKRGITISTSHVEYETTKRHYAHIDCPGHEDYVKNMITGAAQMDGAILVVSAVDGPMPQTREHILLSRQVGVPSLVVFLNKVDMVNDPEMLDLVEMEVRELLLSYKYPGDEIPIIRGSALKALQ** 180

**Euglenida**  11 **PHINIGTIGHVDHGKTTLTAAITMALSVT.GN.T.KSKKYEEIDSSPEEKARGITINTAHVEYETKNRHYAHVDCPGHADYIKNMITGAAQMDGAILVISATDGPMPQTKEHILLAKQVGVPNLVVFLNKEDQIDDNELLELIELEIRETLNNYEFPGDEIPIITGSALLAIE** 180

**Rhodophyta**  11 **PHVNIGTIGHVDHGKTTLTAAISAVLASK.DN.TvQLKKFEEIDSAPEERARGITINTSHVEYQTEKRHYAHVDCPGHADYVKNMITGAAQMDGAILVVSAADGPMPQTREHILLAKQVGVPSIVVFLNKADMVDDPELLELVELEVRELLSKYDFPGDTIPFVTGSALLALE** 181

**position**  . 20 . 30 . 40 . 50 . 60 . 70 . 80 . 90 . 100 . 110 . 120 . 130 . 140 . 150 . 160 . 170 . 180

**_**

**__**

**__**

**__**

**__**

**__**

**__**

**__**

**___ _ _**

**___ _ _ _**

**___ _ _ _**

**___ _ _ _**

**_ ___ _ _ _**

**_ ___ _ _ _**

**_ ___ _ _ _**

**_ ___ _ _ _**

**_ ___ _ _ _ _**

**_ ___ _ _ _ _**

**_ ___ _ _ __ _**

**_ ___ _ _ __ _**

**_ ___ _ _ __ _**

**_ ___ _ _ __ _**

**_ ___ _ _ __ _**

**_ ___ _ _ __ _**

**_ ___ _ _ __ _**

**_ ____ _ _ __ _**

**_ ____ _ _ __ _**

**_ ____ _ _ __ _**

**_ ____ _ _ __ _**

**_ ____ _ _ __ _**

**_ ____ _ _ __ _**

**_ ____ _ _ __ _**

**_ ____ _ _ __ _**

**_ _ ____ _ _ _ _ __ _**

**_ _ ____ _ _ _ _ _ __ _**

**_ _ ____ _ _ _ _ _ __ _**

**_ _ _ _ ____ _ _ _ _ _ __ _**

**_ _ _ _ ____ _ _ _ _ _ _ __ _ _**

**_ _ _ _ ____ _ _ _ __ _ _ __ _ _**

**_ ___ _ ____ _ _ _ __ _ _ __ _ _**

**_ ___ _ ____ _ _ _ __ _ __ __ _ _**

**_ ___ _ ____ _ _ _ _ __ _ __ __ _ _**

**_ ___ _ _____ _ _ _ _ __ _ __ __ _ _**

**_ ___ _ _____ _ _ _ _ __ _ __ _ __ _ _**

**_ ___ _ _____ _ _ _ _ __ _ __ _ __ _ _**

**_ ___ _ _____ _ _ _ _ __ _ __ __ __ _ _**

**_ ___ _ _____ _ _ _ _ __ _ __ ___ __ _ _**

**_ ___ _ _____ _ _ _ _ __ _ __ ___ __ _ _**

**_ ___ _ _____ _ _ _ _ __ _ __ ___ __ _ _**

**_ ___ _ ______ _ _ _ ____ _ ___ ___ __ _ __**

**_ ___ _ ______ _ _ _ ____ _ ___ ___ __ _ __**

**P-loop GTPases**  ● ●●● ● ●●●●●● ● ● ● ●●●● ● ●●● ●●● ●● ● ●●

**EFTu_1ob5A_the**  10 **PHVNVGTIGHVDHGKTTLTAALTYVAAAE.NPnV.EVKDYGDIDKAPEERARGITINTAHVEYETAKRHYSHVDCPGHADYIKNMITGAAQMDGAILVVSAADGPMPQTREHILLARQVGVPYIVVFMNKVDMVDDPELLDLVEMEVRDLLNQYEFPGDEVPVIRGSALLALE** 180*

**EFTu_1efuA_eco**  10 **PHVNVGTIGHVDHGKTTLTAAITTVLAKT.YG.G.AARAFDQIDNAPEEKARGITINTSHVEYDTPTRHYAHVDCPGHADYVKNMITGAAQMDGAILVVAATDGPMPQTREHILLGRQVGVPYIIVFLNKCDMVDDEELLELVEMEVRELLSQYDFPGDDTPIVRGSALKALE** 179

**EFTu_1xb2A_cow**  13 **PHVNVGTIGHVDHGKTTLTAAITKILAEG.GG.A.KFKKYEEIDNAPEERARGITINAAHVEYSTAARHYAHTDCPGHADYVKNXITGTAPLDGCILVVAANDGPXPQTREHLLLARQIGVEHVVVYVNKADAVQDSEXVELVELEIRELLTEFGYKGEETPIIVGSALCALE** 182

**EFTU_SALRD**  11 **PHVNVGTIGHVDHGKTTLTAAITKVLAERvGG.A.AEQTFEAIDNAPEERERGITIATSHVEYETENRHYAHVDCPGHADYVKNMVTGAAQMDGAILVVGSDDGPMPQTREHILLARQVGVPYLVVFMNKTDLVDDAELLELVEMEVRELLTEYEFPGDEVPVVRGSALQALE** 181

**EFTU_PSEAK**  12 **-HVNIGTIGHVDHGKTTLTAAITMCLQSF.SK.N.KGKRYDEIDSAPEEKARGITINTAHVEYETENRHYAHVDCPGHADYVKNMITGAAQMDGAILVVSGADGPMPQTKEHLLLAKQVGVPTLVVFLNKEDQVDDPELLELVELEVRETLDKYEYPGDDIPIIAGSALLALE** 180

**EFTU_DEHM1**  11 **PHCNVGTIGHVDHGKTTLTAAITRTLSTK.GW.A.DFRAYDQIDNAPEEKARGLTIAISHIEYQTETRHYAHIDCPGHADYIKNMITGAAQMDGAILVVSAPDGPMPQTREHVLLIHQVEVPAVVVALNKCDMMDDEELLELVELEVRELLTKNSFPGDEIPIVRVSAIKALE** 180

**XP_002115166.1**  25 **PHINIGTIGHVDHGKTSLTAAITKLLQER.GQ.A.KYKAYDEIDNAPEEKARGITIKTANVEYETDQRHYGHIDCPGHADYIKNMITGAARMDGAILVVAATDGAMPQTKEHVLLAKQIGVKHMVVYVNKADTIDDNEMLELVELEIRDLLQEHGYD-EDTPVIIGSALCALE** 193

**XP_002159524.1**  59 **PHINIGTIGHVDHGKTTLTAAITKVLSEK.GG.S.KFKDYADIDNAPEERARGITINASHVEYETDTRHYGHIDCPGHADYIKNMITGAAQMDGAILVVAATDGQMPQTREHLLLANQIGVKNLCVFINKADMVDDKEIMDLVEMEIRELLTEYGYDGDNTPVIGGSALCALE** 228

**YP_003289178.1**  11 **PHINIGTIGHVDHGKTTLTAAITAVLALA.GD.A.NAKKYEDIDAAPEERARGITINTAHVEYETATRHYAHVDCPGHADYVKNMITGAAQMDGAILVVSAADGPMPQTREHILLSKQVGVPHIVVFLNKEDQVDDLELVELVELEVRELLSNYDFPGDDIPIVTGSALQALD** 180

**WP_026853108.1**  11 **PHVNIGTIGHVDHGKTTLTAAITFILAKK.FG.G.ETKSYDQIDSAPEEKARGITINTAHVEYQTEKRHYAHVDCPGHADYVKNMITGAAQMDGAILVVAATDGPMPQTREHILLARQVGVPYIVVFMNKIDIA-DPELAELVEMEIRDLLSSYQFPGDETPIIKGSARLALD** 179

**WP_038549660.1**  11 **PHLNVGTIGHIDHGKTTLTSAITRVLSTK.GY.A.QWIPFDQIDKAPEEKARGITIQLAHVEYESDKRHYAHIDCPGHADYIKNMITGAAQMDGSILVVAATDGPMPQTREHVLLARQVNVPAMVVFMNKVDMVDDAELLDLVELEVRELLSKYGFPGDEVPVIRGSALKALE** 180

**KFH66023.1**  78 **PHVNIGTIGHVDHGKTTLTAAITKTLASR.GG.A.EFKDYNQIDKAPEEKARGITISTAHVEYETENRHYAHVDCPGHADYIKNMITGAAQMDGAIIVVAATDGQMPQTREHLLLAKQVGIQKLVVFINKVDAVDDPEMLELVDMEMRDVLTTYGFDGENTPIIMGSALCALE** 247

**EFTU_FIBSS**  11 **PHCNIGTIGHVDHGKTTLTAAICTTLAAK.GL.A.AAKRFDEIDNAPEEKARGITINTSHVEYTTANRHYAHVDCPGHADYVKNMVTGAAQMDGAILVVAATDGPMPQTREHILLAHQVGVPKIVVFMNKCDMVDDAEILDLVEMEVRELLSKYDFDGDNTPIIRGSALKALE** 180

**EFTU_RECAM**  11 **PHCNIGTIGHVDHGKTTLTAAITKVLSET.GG.A.VFTDYDQIDKAPEEKKRGITISTSHVEYETTKRHYAHIDCPGHEDYVKNMITGAAQMDGAILVVSAVDGPMPQTREHILLSRQVGVPSLVVFLNKVDMVNDPEMLDLVEMEVRELLLSYKYPGDEIPIIRGSALKALQ** 180

**EFTU_EUGLO**  11 **PHINIGTIGHVDHGKTTLTAAITMALSVT.GN.T.KSKKYEEIDSSPEEKARGITINTAHVEYETKNRHYAHVDCPGHADYIKNMITGAAQMDGAILVISATDGPMPQTKEHILLAKQVGVPNLVVFLNKEDQIDDNELLELIELEIRETLNNYEFPGDEIPIITGSALLAIE** 180

**EFTU_CYAME**  11 **PHVNIGTIGHVDHGKTTLTAAISAVLASK.DN.TvQLKKFEEIDSAPEERARGITINTSHVEYQTEKRHYAHVDCPGHADYVKNMITGAAQMDGAILVVSAADGPMPQTREHILLAKQVGVPSIVVFLNKADMVDDPELLELVELEVRELLSKYDFPGDTIPFVTGSALLALE** 181

**foreground (127070):**  **KIRNVGVVGDSGAGKSSLFNAFAG EGAK GG G EADAADETDGATDEAGRGRTATSATVEVGGEGVDFTFWDTAGQSDYRGEREEGAEGADGAVFVYDASSGDSAETVAEWDEAVKEGKPKVVVAGTKADKADADGGAADEAEAFAEKLSSYGYGGDGAPYFETSAKTGDG**

**RPPRLLLL LPDV TT L RLLK KVLL LL E KLKLFLLI VL PEITPDI IKLL LE KKIKLVLI LP LRRF SLL RFLREV LLLLLLSITEPVTFQDLKKLLLELLKKL YLLLLLN M LPE REVSELLLREL LLPNK EFPLENL ILPI L NEN**

**LLKIVII HVNS I KIV FII F D I FY QV RV TV INV VQ I ID D INIV G HEK A YIKD VIIV I V DRE NIE IFK I DI HIIIII L L E EIIK K TE D DI I VI V**

**wt_res_freqs (29569): 111131239113299526132112 1111 23 2 214132129111111113121111121111111111118529211111111111113422215163112121111111111111214112211281911121111111111121113174224311211116522314**

**11111121 1213 45 2 2411 1111 11 1 11121444 11 1112121 1111 11 11112132 14 1112 121 112211 1133111121111111111111222111 1123126 1 311 21211111112 11113 2731111 1111 1 112**

**1123122 1131 1 111 112 1 1 1 12 12 11 31 211 11 1 11 1 1122 1 121 1 2111 1221 1 1 211 111 111 1 11 312111 1 1 1 2111 1 22 1 21 1 22 2**

**insertions**

**deletions 421965433222211111 113 119 99 9 9999999998 1 651177 55355631194 3 1 8 7 333 11 71122191118912898857779 21888119191114516119999999918117 1112223**

**position**  . 20 . 30 . 40 . 50 . 60 . 70 . 80 . 90 . 100 . 110 . 120 . 130 . 140 . 150 . 160 . 170 . 180

**_**

**_ _**

**_ _**

**_ _**

**_ _**

**_ _**

**_ _ _ _**

**_ _ _ _**

**_ _ _ _**

**_ _ _ _**

**_ _ _ _**

**___ _ _**

**___ _ _ _**

**___ _ _ _**

**___ _ _ __**

**___ _ _ __**

**___ _ _ __**

**___ _ _ __**

**___ _ _ _ __**

**___ _ __ _ __**

**___ _ __ _ __**

**___ _ __ _ __**

**___ _ __ _ _ __**

**___ _ __ _ _ __**

**___ _ __ _ _ __**

**___ _ __ _ _ __**

**___ _ __ _ _ __**

**___ _ _ __ _ _ __**

**___ _ _ __ _ _ __**

**___ _ _ __ _ _ __**

**___ _ _ __ _ _ __**

**___ _ _ __ _ _ __**

**___ _ _ __ _ _ __**

**___ _ _ __ _ _ __**

**___ _ _ __ _ _ _ _ _ __**

**___ __ ____ _ _ _ _ _ __**

**___ __ ____ _ _ _ _ _ __**

**___ __ ____ _ _ _ _ _ __**

**___ __ _____ _ _ _ __ _ __**

**___ __ _____ _ _ _ __ _ __**

**___ __ _____ _ _ _ __ _ __**

**___ __ _____ _ _ _ __ _ _ __**

**___ ________ _ _ _ __ _ _ __**

**___ ________ _ _ _ __ _ _ __**

**___ ________ _ _ _ __ _ _ __**

**____ ________ _ _ _ __ _ _ _ _ __ _**

**____ _ ________ _ _ _ __ _ _ _ _ _ __ _**

**____ _ ________ _ _ _ __ _ _ _ _ _ __ _**

**____ _ ________ _ _ _ __ _ _ _ _ _ __ _**

**____ _ _ ________ _ _ _ _ __ _ _ _ _ __ ___ _**

**____ _ _ ________ _ _ _ _ __ _ _ _ _ __ ___ _**

**TF family**  ●●●● ● ● ●●●●●●●● ● ● ● ● ●● ● ● ● ● ●● ●●● ●

**EFTu_1ob5A_the**  10 **PHVNVGTIGHVDHGKTTLTAALTYVAAAE.NPnV.EVKDYGDIDKAPEERARGITINTAHVEYETAKRHYSHVDCPGHADYIKNMITGAAQMDGAILVVSAADGPMPQTREHILLARQVGVPYIVVFMNKVDMVDDPELLDLVEMEVRDLLNQYEFPGDEVPVIRGSALLALE** 180*

**EFTu_1efuA_eco**  10 **PHVNVGTIGHVDHGKTTLTAAITTVLAKT.YG.G.AARAFDQIDNAPEEKARGITINTSHVEYDTPTRHYAHVDCPGHADYVKNMITGAAQMDGAILVVAATDGPMPQTREHILLGRQVGVPYIIVFLNKCDMVDDEELLELVEMEVRELLSQYDFPGDDTPIVRGSALKALE** 179

**EFTu_1xb2A_cow**  13 **PHVNVGTIGHVDHGKTTLTAAITKILAEG.GG.A.KFKKYEEIDNAPEERARGITINAAHVEYSTAARHYAHTDCPGHADYVKNXITGTAPLDGCILVVAANDGPXPQTREHLLLARQIGVEHVVVYVNKADAVQDSEXVELVELEIRELLTEFGYKGEETPIIVGSALCALE** 182

**EFTU_SALRD**  11 **PHVNVGTIGHVDHGKTTLTAAITKVLAERvGG.A.AEQTFEAIDNAPEERERGITIATSHVEYETENRHYAHVDCPGHADYVKNMVTGAAQMDGAILVVGSDDGPMPQTREHILLARQVGVPYLVVFMNKTDLVDDAELLELVEMEVRELLTEYEFPGDEVPVVRGSALQALE** 181

**EFTU_PSEAK**  12 **-HVNIGTIGHVDHGKTTLTAAITMCLQSF.SK.N.KGKRYDEIDSAPEEKARGITINTAHVEYETENRHYAHVDCPGHADYVKNMITGAAQMDGAILVVSGADGPMPQTKEHLLLAKQVGVPTLVVFLNKEDQVDDPELLELVELEVRETLDKYEYPGDDIPIIAGSALLALE** 180

**EFTU_DEHM1**  11 **PHCNVGTIGHVDHGKTTLTAAITRTLSTK.GW.A.DFRAYDQIDNAPEEKARGLTIAISHIEYQTETRHYAHIDCPGHADYIKNMITGAAQMDGAILVVSAPDGPMPQTREHVLLIHQVEVPAVVVALNKCDMMDDEELLELVELEVRELLTKNSFPGDEIPIVRVSAIKALE** 180

**XP_002115166.1**  25 **PHINIGTIGHVDHGKTSLTAAITKLLQER.GQ.A.KYKAYDEIDNAPEEKARGITIKTANVEYETDQRHYGHIDCPGHADYIKNMITGAARMDGAILVVAATDGAMPQTKEHVLLAKQIGVKHMVVYVNKADTIDDNEMLELVELEIRDLLQEHGYD-EDTPVIIGSALCALE** 193

**XP_002159524.1**  59 **PHINIGTIGHVDHGKTTLTAAITKVLSEK.GG.S.KFKDYADIDNAPEERARGITINASHVEYETDTRHYGHIDCPGHADYIKNMITGAAQMDGAILVVAATDGQMPQTREHLLLANQIGVKNLCVFINKADMVDDKEIMDLVEMEIRELLTEYGYDGDNTPVIGGSALCALE** 228

**YP_003289178.1**  11 **PHINIGTIGHVDHGKTTLTAAITAVLALA.GD.A.NAKKYEDIDAAPEERARGITINTAHVEYETATRHYAHVDCPGHADYVKNMITGAAQMDGAILVVSAADGPMPQTREHILLSKQVGVPHIVVFLNKEDQVDDLELVELVELEVRELLSNYDFPGDDIPIVTGSALQALD** 180

**WP_026853108.1**  11 **PHVNIGTIGHVDHGKTTLTAAITFILAKK.FG.G.ETKSYDQIDSAPEEKARGITINTAHVEYQTEKRHYAHVDCPGHADYVKNMITGAAQMDGAILVVAATDGPMPQTREHILLARQVGVPYIVVFMNKIDIA-DPELAELVEMEIRDLLSSYQFPGDETPIIKGSARLALD** 179

**WP_038549660.1**  11 **PHLNVGTIGHIDHGKTTLTSAITRVLSTK.GY.A.QWIPFDQIDKAPEEKARGITIQLAHVEYESDKRHYAHIDCPGHADYIKNMITGAAQMDGSILVVAATDGPMPQTREHVLLARQVNVPAMVVFMNKVDMVDDAELLDLVELEVRELLSKYGFPGDEVPVIRGSALKALE** 180

**KFH66023.1**  78 **PHVNIGTIGHVDHGKTTLTAAITKTLASR.GG.A.EFKDYNQIDKAPEEKARGITISTAHVEYETENRHYAHVDCPGHADYIKNMITGAAQMDGAIIVVAATDGQMPQTREHLLLAKQVGIQKLVVFINKVDAVDDPEMLELVDMEMRDVLTTYGFDGENTPIIMGSALCALE** 247

**EFTU_FIBSS**  11 **PHCNIGTIGHVDHGKTTLTAAICTTLAAK.GL.A.AAKRFDEIDNAPEEKARGITINTSHVEYTTANRHYAHVDCPGHADYVKNMVTGAAQMDGAILVVAATDGPMPQTREHILLAHQVGVPKIVVFMNKCDMVDDAEILDLVEMEVRELLSKYDFDGDNTPIIRGSALKALE** 180

**EFTU_RECAM**  11 **PHCNIGTIGHVDHGKTTLTAAITKVLSET.GG.A.VFTDYDQIDKAPEEKKRGITISTSHVEYETTKRHYAHIDCPGHEDYVKNMITGAAQMDGAILVVSAVDGPMPQTREHILLSRQVGVPSLVVFLNKVDMVNDPEMLDLVEMEVRELLLSYKYPGDEIPIIRGSALKALQ** 180

**EFTU_EUGLO**  11 **PHINIGTIGHVDHGKTTLTAAITMALSVT.GN.T.KSKKYEEIDSSPEEKARGITINTAHVEYETKNRHYAHVDCPGHADYIKNMITGAAQMDGAILVISATDGPMPQTKEHILLAKQVGVPNLVVFLNKEDQIDDNELLELIELEIRETLNNYEFPGDEIPIITGSALLAIE** 180

**EFTU_CYAME**  11 **PHVNIGTIGHVDHGKTTLTAAISAVLASK.DN.TvQLKKFEEIDSAPEERARGITINTSHVEYQTEKRHYAHVDCPGHADYVKNMITGAAQMDGAILVVSAADGPMPQTREHILLAKQVGVPSIVVFLNKADMVDDPELLELVELEVRELLSKYDFPGDTIPFVTGSALLALE** 181

**foreground (25224):**  **NHRNFGTAGHVDHGKSSTADAITRDAGAK GG G EADAADETDGAAAEQERGISITAGAASFETDGRKYAFVDCPGHAAYSANVAAGAAACDGAVVVVAAADGVEAQSEAAWRQCDAAGVKKRVVAVNKVDKVGANEVKDEVEDEFAGLGSSYGYGGDDTPFVFGSGKTGDG**

**PPPVITIMA I A TTLLERLLFYLKVL LL E KLKLFLLI VMPL RG TQHLAYVYL YPKYHLNLL T VDFIYEMIRSLRVMEIVLLLID VK IMP TLEVLLLLRLLKLPYPLPVL M LPDYEPLLELLIELLLE LNK EFPLE I ILPI ALKNLN**

**RIL VAVI VG IYKT KI F D I FY QV RLKQ K KISHTQV WKDHRITII A E VKMRETA SQL I S DQ PQE ITHIYIAKRYNI HLILFI I R DDFIV QIR IRD ETE D DI V VI V K**

**wt_res_freqs (4165): 11562321596959931114231111511 23 2 214132129111191589917112311222122111119199921121241143211846316627129521911111221111441112423991912241222123211111223174224321413138122514**

**111141514 2 2 68712355111111 11 1 11121444 1211 41 811131211 211211421 6 3681143152422111226225 11 134 81322131212124111121 3 22212211211112212 113 27311 1 1121 732112**

**321 2214 11 1113 12 1 1 1 12 12 1211 1 1111211 111113235 1 2 1111221 211 3 1 11 111 113111521112 314133 2 3 13111 121 211 122 1 21 1 32 2 1**

**insertions 1**

**deletions 321843332221111 4 1 58 88 8 8888888882 1 22225 8 1 76 1 99999888 1111123**

**background (101846):**  **KLFKVGVVGDSGAGKSSFFNAFAGGEAA AVTDFAGTGRTAGTQTVEVGGGGVDFTFWDTPGQSDYDGTGDEAYEGADGAVHVYSASSGDSFDNAAEWDEEVKEGVP VVVAGTKADLATAEGGAADEGEAFAEEL GAPYFGTSAKTGDG**

**PPRLLLL LPNV TTLL RLLK KVL T PIPTLTPDVIIVLL LEDKKLKLVLI LG LLRFRSLLELFLREV LVLLLLDSTDPQTLEELKKLLK LLKKI LLLLLN L LEEREVSEE LREL LKP NL ILPI SL EN**

**L IVII RT S I KIV FI Y I VNF VK I ID E I IQIV IA IRKGEA W YIKD VIIV I V RE DI F I DK IIIII I D K IK K I VIEV R**

**wt_res_freqs (25404): 1112312491132995311421121111 211111112111111121112111111118539211111111111113311215112111122111111112111114 2221128183111111111111112111 21211126522314**

**1111131 1334 3453 2411 111 1 11241313111111 11111113132 11 111211211112211 1233116122111111111111 22111 123126 1 111212112 1112 111 11 1111 11 12**

**1 3121 11 1 2 111 11 1 2 111 11 1 12 1 1 1122 13 111111 1 2111 1221 1 1 11 11 1 2 11 12111 1 1 1 11 1 1 2221 1**

**position**  . 20 . 30 . 40 . 50 . 60 . 70 . 80 . 90 . 100 . 110 . 120 . 130 . 140 . 150 . 160 . 170 . 180

**_**

**_**

**_**

**_**

**_**

**_**

**_**

**_**

**_**

**_**

**_**

**_**

**_**

**_**

**_**

**_**

**_**

**_**

**_**

**_**

**_**

**_**

**_**

**_**

**_**

**_ _**

**_ _**

**_ _**

**_ _**

**_ _ _**

**_ _ _**

**_ _ _**

**_ _ _ _**

**_ _ _ _**

**_ _ _ _ _ _**

**_ _ _ _ _ _**

**_ _ _ _ _ _ _ _ _**

**_ _ _ _ _ __ _ _ _**

**_ _ _ _ _ __ _ __ _**

**_ _ _ _ _ __ _ __ _**

**_ _ _ _ _ _ __ _ __ _**

**_ _ _ _ _ _ __ _ __ _**

**_ _ _ _ _ _ _ __ _ __ _ _ _**

**_ _ _ _ _ _ _ __ _ __ _ _ _**

**_ _ _ _ _ _ _ _ _ __ _ _____ _ _**

**_ _ _ __ _ _ _ _ _ _ __ _ _ _____ _ _ _ _**

**_ _ _ __ _ _ _ _ _ _ __ _ _ _____ __ _ _ _ _ _ _**

**_ _ _ __ _ _ _ _ _ _ __ _ _ _____ __ _ __ _ _ _ _ _**

**_ _ _ __ _ _ _ _ _ _ __ _ _ _____ __ _ __ _ _ _ _ _**

**_ _ _ __ _ _ _ _ _ _ __ _ _ _____ __ _ __ _ _ _ _ _**

**_ _ _ __ _ _ _ _ _ _ __ _ _ _____ __ _ __ _ _ _ _ _**

**EF-Tu/CysN**  ● ● ● ●● ● ● ● ● ● ● ●● ● ● ●●●●● ●● ● ●● ● ● ● ● ●

**EFTu_1ob5A_the**  10 **PHVNVGTIGHVDHGKTTLTAALTYVAAAE.NPnV.EVKDYGDIDKAPEERARGITINTAHVEYETAKRHYSHVDCPGHADYIKNMITGAAQMDGAILVVSAADGPMPQTREHILLARQVGVPYIVVFMNKVDMVDDPELLDLVEMEVRDLLNQYEFPGDEVPVIRGSALLALE** 180*

**EFTu_1efuA_eco**  10 **PHVNVGTIGHVDHGKTTLTAAITTVLAKT.YG.G.AARAFDQIDNAPEEKARGITINTSHVEYDTPTRHYAHVDCPGHADYVKNMITGAAQMDGAILVVAATDGPMPQTREHILLGRQVGVPYIIVFLNKCDMVDDEELLELVEMEVRELLSQYDFPGDDTPIVRGSALKALE** 179

**EFTu_1xb2A_cow**  13 **PHVNVGTIGHVDHGKTTLTAAITKILAEG.GG.A.KFKKYEEIDNAPEERARGITINAAHVEYSTAARHYAHTDCPGHADYVKNXITGTAPLDGCILVVAANDGPXPQTREHLLLARQIGVEHVVVYVNKADAVQDSEXVELVELEIRELLTEFGYKGEETPIIVGSALCALE** 182

**EFTU_SALRD**  11 **PHVNVGTIGHVDHGKTTLTAAITKVLAERvGG.A.AEQTFEAIDNAPEERERGITIATSHVEYETENRHYAHVDCPGHADYVKNMVTGAAQMDGAILVVGSDDGPMPQTREHILLARQVGVPYLVVFMNKTDLVDDAELLELVEMEVRELLTEYEFPGDEVPVVRGSALQALE** 181

**EFTU_PSEAK**  12 **-HVNIGTIGHVDHGKTTLTAAITMCLQSF.SK.N.KGKRYDEIDSAPEEKARGITINTAHVEYETENRHYAHVDCPGHADYVKNMITGAAQMDGAILVVSGADGPMPQTKEHLLLAKQVGVPTLVVFLNKEDQVDDPELLELVELEVRETLDKYEYPGDDIPIIAGSALLALE** 180

**EFTU_DEHM1**  11 **PHCNVGTIGHVDHGKTTLTAAITRTLSTK.GW.A.DFRAYDQIDNAPEEKARGLTIAISHIEYQTETRHYAHIDCPGHADYIKNMITGAAQMDGAILVVSAPDGPMPQTREHVLLIHQVEVPAVVVALNKCDMMDDEELLELVELEVRELLTKNSFPGDEIPIVRVSAIKALE** 180

**XP_002115166.1**  25 **PHINIGTIGHVDHGKTSLTAAITKLLQER.GQ.A.KYKAYDEIDNAPEEKARGITIKTANVEYETDQRHYGHIDCPGHADYIKNMITGAARMDGAILVVAATDGAMPQTKEHVLLAKQIGVKHMVVYVNKADTIDDNEMLELVELEIRDLLQEHGYD-EDTPVIIGSALCALE** 193

**XP_002159524.1**  59 **PHINIGTIGHVDHGKTTLTAAITKVLSEK.GG.S.KFKDYADIDNAPEERARGITINASHVEYETDTRHYGHIDCPGHADYIKNMITGAAQMDGAILVVAATDGQMPQTREHLLLANQIGVKNLCVFINKADMVDDKEIMDLVEMEIRELLTEYGYDGDNTPVIGGSALCALE** 228

**YP_003289178.1**  11 **PHINIGTIGHVDHGKTTLTAAITAVLALA.GD.A.NAKKYEDIDAAPEERARGITINTAHVEYETATRHYAHVDCPGHADYVKNMITGAAQMDGAILVVSAADGPMPQTREHILLSKQVGVPHIVVFLNKEDQVDDLELVELVELEVRELLSNYDFPGDDIPIVTGSALQALD** 180

**WP_026853108.1**  11 **PHVNIGTIGHVDHGKTTLTAAITFILAKK.FG.G.ETKSYDQIDSAPEEKARGITINTAHVEYQTEKRHYAHVDCPGHADYVKNMITGAAQMDGAILVVAATDGPMPQTREHILLARQVGVPYIVVFMNKIDIA-DPELAELVEMEIRDLLSSYQFPGDETPIIKGSARLALD** 179

**WP_038549660.1**  11 **PHLNVGTIGHIDHGKTTLTSAITRVLSTK.GY.A.QWIPFDQIDKAPEEKARGITIQLAHVEYESDKRHYAHIDCPGHADYIKNMITGAAQMDGSILVVAATDGPMPQTREHVLLARQVNVPAMVVFMNKVDMVDDAELLDLVELEVRELLSKYGFPGDEVPVIRGSALKALE** 180

**KFH66023.1**  78 **PHVNIGTIGHVDHGKTTLTAAITKTLASR.GG.A.EFKDYNQIDKAPEEKARGITISTAHVEYETENRHYAHVDCPGHADYIKNMITGAAQMDGAIIVVAATDGQMPQTREHLLLAKQVGIQKLVVFINKVDAVDDPEMLELVDMEMRDVLTTYGFDGENTPIIMGSALCALE** 247

**EFTU_FIBSS**  11 **PHCNIGTIGHVDHGKTTLTAAICTTLAAK.GL.A.AAKRFDEIDNAPEEKARGITINTSHVEYTTANRHYAHVDCPGHADYVKNMVTGAAQMDGAILVVAATDGPMPQTREHILLAHQVGVPKIVVFMNKCDMVDDAEILDLVEMEVRELLSKYDFDGDNTPIIRGSALKALE** 180

**EFTU_RECAM**  11 **PHCNIGTIGHVDHGKTTLTAAITKVLSET.GG.A.VFTDYDQIDKAPEEKKRGITISTSHVEYETTKRHYAHIDCPGHEDYVKNMITGAAQMDGAILVVSAVDGPMPQTREHILLSRQVGVPSLVVFLNKVDMVNDPEMLDLVEMEVRELLLSYKYPGDEIPIIRGSALKALQ** 180

**EFTU_EUGLO**  11 **PHINIGTIGHVDHGKTTLTAAITMALSVT.GN.T.KSKKYEEIDSSPEEKARGITINTAHVEYETKNRHYAHVDCPGHADYIKNMITGAAQMDGAILVISATDGPMPQTKEHILLAKQVGVPNLVVFLNKEDQIDDNELLELIELEIRETLNNYEFPGDEIPIITGSALLAIE** 180

**EFTU_CYAME**  11 **PHVNIGTIGHVDHGKTTLTAAISAVLASK.DN.TvQLKKFEEIDSAPEERARGITINTSHVEYQTEKRHYAHVDCPGHADYVKNMITGAAQMDGAILVVSAADGPMPQTREHILLAKQVGVPSIVVFLNKADMVDDPELLELVELEVRELLSKYDFPGDTIPFVTGSALLALE** 181

**foreground (4429):**  **SHVNFGTCGSVDDGKSTLTGAITKDSAAK GG G EADAADETDGAQAEKAQGITIATAHREYATANRKYAHADCPGHADYTKNMATGAATADGAVVVVAAADGVLTQTKEHAFIASQVGVKHVVVAVNKCDQVGDSEARDDEEVEFAALLSSYGYGGDNTTFVKGSALKGDN**

**PLLRIL I H H T IARLLYVLKLL LL E KLKLFLLI KLPE RER DVSYVFFE PR HFIIV T EQ IR I SQM L ILLID RK PMP RR LLLSRLL IPYLILFM M M DFPQLLFLVIMALRE NK EFPLEEIPIIPI ALE**

**D VI A H TSKI F D I FY QV N R N Y S EK VI A V V I S T E IY K R I L V L YE EIEE L I D TE D DI DV V RV**

**wt_res_freqs (672): 16353592949949949954554232311 23 2 214132129551393249999137545519119354549599955945991999533858215729259321991591126255861327743991918152711111516221583174224312113149983434**

**625431 5 5 5 5 35444234311 11 1 11121444 1455 665 44144144 11 54424 3 33 24 5 455 2 76424 32 455 74 1561343 35121141 3 2 611143355431154 13 273111353641 543**

**1 21 1 1 1112 1 1 1 12 12 1 1 2 3 2 24 11 1 3 2 2 3 2 1 31 1 1 4 3 1 4 22 1131 1 1 1 22 1 11 42 2 21**

**insertions 1 1**

**deletions 2243222111 1 11 1 111 5 44444232 1**

**background (20795):**  **NIRNFGVIGHVDAGKSSTAGAIIRQAGAE GD A EAKSNDAIDTVAEEAGRGISQTAGAASFETNGRKVTFVDAPGHAAFGKNMAAGAAACDGAVVVVAGADGVEAQSEAHWRICDALGVESRVVAVNKVDKVGANEVKAAVEDLTAGGGKKYGYDGDDTPAFFGSGKTGDG**

**RPPVIVIMA I H TTLLERLLFYT VI VL R KPMKYEKLAVMKL RE TIHLK VQLFLPKYLLNLL T VD IYMRIRSLSVLEIVLLLIDAVQ IMP TLELLYLLRLAKLPHPLPVL M LPD EPILKQLRK LLELEIY FP H V ILPI ALKNKN**

**K VT VD YKS RK AY T QM Q S RLEI K KIS YTV YKDHRI II E TGEVE A RQV I I DE QE ITVI QAKRYDIKKLILFI L R DF VEEI E IIAID E F VI V E**

**wt_res_freqs (3493): 13662324495929931111221111522 31 2 114135245211191199811112411211121112219199911911121132121846316521129522912121211112341212423991911351122112112111132365325121411128122515**

**411141625 3 5 67613356114 22 11 1 2212212211212 45 86111 1111111211532 7 36 1111162513211226225621 124 81312122211124111121 3 231 231111211 2112122 34 2 1 1121 621212**

**2 21 14 111 11 12 3 21 2 1 1221 1 221 111 222114 35 1 31552 1 211 2 1 22 11 1231 24211111215123 1 4 31 1232 1 21111 2 1 31 2 1**

**position**  . 20 . 30 . 40 . 50 . 60 . 70 . 80 . 90 . 100 . 110 . 120 . 130 . 140 . 150 . 160 . 170 . 180

**_**

**_**

**_**

**_**

**_**

**_**

**_**

**_**

**_**

**_**

**_**

**_**

**_ _**

**_ _ _**

**_ _ _**

**_ _ _**

**_ _ _**

**_ _ _**

**_ _ _**

**_ _ _ _**

**_ _ _ _**

**_ _ _ _**

**_ _ _ _**

**_ _ _ _ _**

**_ _ _ _ _ _**

**_ _ _ _ _ _**

**_ _ _ _ _ _**

**_ _ _ _ _ _**

**_ _ _ _ _ _**

**_ _ _ _ _ _**

**_ _ _ _ _ _**

**_ _ _ _ _ _ _**

**_ _ _ _ _ _ _**

**_ _ _ _ _ _ _**

**_ _ _ __ _ _ _**

**_ _ _ __ _ _ _**

**_ _ _ _ __ _ _ _**

**_ _ _ _ __ _ _ _**

**_ _ _ _ _ _ __ _ __ _**

**_ _ _ _ _ _ __ _ __ _**

**_ _ ___ _ _ __ _ __ _**

**_ _ ___ _ _ __ _ __ _**

**_ _ ___ _ _ __ _ __ _**

**_ _ ___ _ _ _ _ __ _ __ _ _**

**_ _ ___ _ _ _ _ __ _ __ _ _ _**

**_ _ _ ___ _ _ _ _ _ __ _ __ _ _ _ _**

**_ _ _ ___ _ _ _ _ _ __ _ __ _ _ _ _ _**

**_ _ _ ___ _ _ _ _ _ __ _ __ _ _ _ _ _ _**

**_ _ _ ___ _ _ _ _ __ _ __ _ __ _ _ _ _ _ _ _ _**

**_ _ _ _ ___ _ ___ _ __ _ __ _ __ _ _ _ _ _ _ _ _ _ _**

**_ _ _ _ ___ _ ___ _ __ _ __ _ __ _ _ _ _ _ _ _ _ _ _**

**EF-Tu**  ● ● ● ● ●●● ● ●●● ● ●● ● ●● ● ●● ● ● ● ● ● ● ● ● ● ●

**EFTu_1ob5A_the**  10 **PHVNVGTIGHVDHGKTTLTAALTYVAAAE.NPnV.EVKDYGDIDKAPEERARGITINTAHVEYETAKRHYSHVDCPGHADYIKNMITGAAQMDGAILVVSAADGPMPQTREHILLARQVGVPYIVVFMNKVDMVDDPELLDLVEMEVRDLLNQYEFPGDEVPVIRGSALLALE** 180*

**EFTu_1efuA_eco**  10 **PHVNVGTIGHVDHGKTTLTAAITTVLAKT.YG.G.AARAFDQIDNAPEEKARGITINTSHVEYDTPTRHYAHVDCPGHADYVKNMITGAAQMDGAILVVAATDGPMPQTREHILLGRQVGVPYIIVFLNKCDMVDDEELLELVEMEVRELLSQYDFPGDDTPIVRGSALKALE** 179

**EFTu_1xb2A_cow**  13 **PHVNVGTIGHVDHGKTTLTAAITKILAEG.GG.A.KFKKYEEIDNAPEERARGITINAAHVEYSTAARHYAHTDCPGHADYVKNXITGTAPLDGCILVVAANDGPXPQTREHLLLARQIGVEHVVVYVNKADAVQDSEXVELVELEIRELLTEFGYKGEETPIIVGSALCALE** 182

**EFTU_SALRD**  11 **PHVNVGTIGHVDHGKTTLTAAITKVLAERvGG.A.AEQTFEAIDNAPEERERGITIATSHVEYETENRHYAHVDCPGHADYVKNMVTGAAQMDGAILVVGSDDGPMPQTREHILLARQVGVPYLVVFMNKTDLVDDAELLELVEMEVRELLTEYEFPGDEVPVVRGSALQALE** 181

**EFTU_PSEAK**  12 **-HVNIGTIGHVDHGKTTLTAAITMCLQSF.SK.N.KGKRYDEIDSAPEEKARGITINTAHVEYETENRHYAHVDCPGHADYVKNMITGAAQMDGAILVVSGADGPMPQTKEHLLLAKQVGVPTLVVFLNKEDQVDDPELLELVELEVRETLDKYEYPGDDIPIIAGSALLALE** 180

**EFTU_DEHM1**  11 **PHCNVGTIGHVDHGKTTLTAAITRTLSTK.GW.A.DFRAYDQIDNAPEEKARGLTIAISHIEYQTETRHYAHIDCPGHADYIKNMITGAAQMDGAILVVSAPDGPMPQTREHVLLIHQVEVPAVVVALNKCDMMDDEELLELVELEVRELLTKNSFPGDEIPIVRVSAIKALE** 180

**XP_002115166.1**  25 **PHINIGTIGHVDHGKTSLTAAITKLLQER.GQ.A.KYKAYDEIDNAPEEKARGITIKTANVEYETDQRHYGHIDCPGHADYIKNMITGAARMDGAILVVAATDGAMPQTKEHVLLAKQIGVKHMVVYVNKADTIDDNEMLELVELEIRDLLQEHGYD-EDTPVIIGSALCALE** 193

**XP_002159524.1**  59 **PHINIGTIGHVDHGKTTLTAAITKVLSEK.GG.S.KFKDYADIDNAPEERARGITINASHVEYETDTRHYGHIDCPGHADYIKNMITGAAQMDGAILVVAATDGQMPQTREHLLLANQIGVKNLCVFINKADMVDDKEIMDLVEMEIRELLTEYGYDGDNTPVIGGSALCALE** 228

**YP_003289178.1**  11 **PHINIGTIGHVDHGKTTLTAAITAVLALA.GD.A.NAKKYEDIDAAPEERARGITINTAHVEYETATRHYAHVDCPGHADYVKNMITGAAQMDGAILVVSAADGPMPQTREHILLSKQVGVPHIVVFLNKEDQVDDLELVELVELEVRELLSNYDFPGDDIPIVTGSALQALD** 180

**WP_026853108.1**  11 **PHVNIGTIGHVDHGKTTLTAAITFILAKK.FG.G.ETKSYDQIDSAPEEKARGITINTAHVEYQTEKRHYAHVDCPGHADYVKNMITGAAQMDGAILVVAATDGPMPQTREHILLARQVGVPYIVVFMNKIDIA-DPELAELVEMEIRDLLSSYQFPGDETPIIKGSARLALD** 179

**WP_038549660.1**  11 **PHLNVGTIGHIDHGKTTLTSAITRVLSTK.GY.A.QWIPFDQIDKAPEEKARGITIQLAHVEYESDKRHYAHIDCPGHADYIKNMITGAAQMDGSILVVAATDGPMPQTREHVLLARQVNVPAMVVFMNKVDMVDDAELLDLVELEVRELLSKYGFPGDEVPVIRGSALKALE** 180

**KFH66023.1**  78 **PHVNIGTIGHVDHGKTTLTAAITKTLASR.GG.A.EFKDYNQIDKAPEEKARGITISTAHVEYETENRHYAHVDCPGHADYIKNMITGAAQMDGAIIVVAATDGQMPQTREHLLLAKQVGIQKLVVFINKVDAVDDPEMLELVDMEMRDVLTTYGFDGENTPIIMGSALCALE** 247

**EFTU_FIBSS**  11 **PHCNIGTIGHVDHGKTTLTAAICTTLAAK.GL.A.AAKRFDEIDNAPEEKARGITINTSHVEYTTANRHYAHVDCPGHADYVKNMVTGAAQMDGAILVVAATDGPMPQTREHILLAHQVGVPKIVVFMNKCDMVDDAEILDLVEMEVRELLSKYDFDGDNTPIIRGSALKALE** 180

**EFTU_RECAM**  11 **PHCNIGTIGHVDHGKTTLTAAITKVLSET.GG.A.VFTDYDQIDKAPEEKKRGITISTSHVEYETTKRHYAHIDCPGHEDYVKNMITGAAQMDGAILVVSAVDGPMPQTREHILLSRQVGVPSLVVFLNKVDMVNDPEMLDLVEMEVRELLLSYKYPGDEIPIIRGSALKALQ** 180

**EFTU_EUGLO**  11 **PHINIGTIGHVDHGKTTLTAAITMALSVT.GN.T.KSKKYEEIDSSPEEKARGITINTAHVEYETKNRHYAHVDCPGHADYIKNMITGAAQMDGAILVISATDGPMPQTKEHILLAKQVGVPNLVVFLNKEDQIDDNELLELIELEIRETLNNYEFPGDEIPIITGSALLAIE** 180

**EFTU_CYAME**  11 **PHVNIGTIGHVDHGKTTLTAAISAVLASK.DN.TvQLKKFEEIDSAPEERARGITINTSHVEYQTEKRHYAHVDCPGHADYVKNMITGAAQMDGAILVVSAADGPMPQTREHILLAKQVGVPSIVVFLNKADMVDDPELLELVELEVRELLSKYDFPGDTIPFVTGSALLALE** 181

**foreground (2434):**  **PHVNVGTIGHVDHGKTTLTAAITTTLAAK GG G EAKAYADIDNAPEEKARGITIATAHVEYESATRHYAHVDCPGHADYVKNMITGAAQMDGAILVVAAADGPMPQTKEHVLLAKQVGVPAIVVYINKCDQVDDAELIDLVELEVRDLLSSYGYDGDNTPVVKGSALKALN**

**L I I KV SKR L A KFVKFEQ K RE NIS I QTEK I I S T R L SR N YLI FM V M P MLE M I E NK EFP EEI IIR E**

**E YRD DE S DN I H L A L E TE D DV**

**wt_res_freqs (366): 99693999998999999999998118513 53 2 233251299399995499999265979971119999979899999969999999999999999839499799991991998299888168911992928891972299929592993174238723933299984981**

**1 6 1 46 221 1 4 2211312 3 33 512 1 1833 1 3 5 4 8 1 16 1 221 71 3 4 3 166 5 2 7 13 275 113 553 7**

**2 111 52 2 22 6 1 6 1 1 3 22 1 41**

**insertions 1 1 1 1 2 11**

**deletions 1554433211 2 1 1 1111111 9 311 1**

**background (1995):**  **SLLKFATCGSVDDGKSTLVGRLLHDSKAV YG G AVDYALLTDGLAAEREQGITIDVAYRYFATAKRSFIVADAPGHEEYTRNMATGASTCDAAVVLVDARKGVQTQTKRHAAVCALLGVKHVVVCVNKIDLVGYSEARYNAIAAFAAQA GFASVTAVPVSALEGDN**

**E RLL A I FET ML L E EP LS L RS F T PR K L T IQ V NSQL IL I H LLP R SFLVS MRIP LILAI M DFDQEVFER VELRELL LKDLRVI M K**

**D I Y LI F D DL F V Q S E I V A V I IIE YIA R I D DK RD SK IENIQF L**

**wt_res_freqs (307): 27917195999999999919999285612 14 2 11916884999189999999999999793914918839919996199999399997161842959985961399199211119772254641699198725442211181255211 41113212929971989**

**1 812 3 8 113 12 2 2 21 53 1 21 2 1 22 6 1 7 18 5 1115 42 4 1 151 7 43224 1162 22382 7 523323721 2311116 1141116 1 2**

**3 4 5 14 3 2 12 3 3 2 4 1 5 1 7 1 5 113 254 3 2 1 21 11 11 211313 2**

**position**  . 20 . 30 . 40 . 50 . 60 . 70 . 80 . 90 . 100 . 110 . 120 . 130 . 140 . 150 . 160 . 170 . 180

***E. coli* versus cow EF-Ts with conserved regions of interaction with EF-Tu highlighted (pdb: 1efuB vs 1xb2B).**

QUERY: 7 LVKELRERTGAGMMDCKKALTEANGDIELAI----ENMRKSGAIKAAKKAGNVAADGVIKTKIDGNYG**IILEVNCQTDFVAKD**AGFQAFADKV------- 95

L+ +LR +TG ++CKKAL GD++ A + +K G KAA+ G +G+I +G+ **+++EVNC+TDFV+++** FQ +V

SBJCT: 7 LLMKLRRKTGYSFINCKKALETCGGDLKQAEswlhKQAQKEGWSKAARLHGRKTKEGLIGLLQEGDTT**VLVEVNCETDFVSRN**LKFQQLVQQValgtllh 106

QUERY: 96 ---------------LDAAVAGKITDVEVLKAQFEEERVALVA**KIGENI**NIRRVAALE---GDVLGSYQHGA------------RIGVLVAAKGAD---- 161

L+++ ++ + +++ + **K+GEN+** ++R A ++ G +GSY HGA + G LV + ++

SBJCT: 107 cqnlkdqlstyskgfLNSSELSELPAGPEREGSLKDQLALAIG**KLGENM**ILKRAAWVKvpaGFYVGSYVHGAmhspslhnlvlgKYGALVICETSElkan 206

QUERY: 162 -EELVKHIAMHVAASKPefikpedvsaevvekeyqvqLDIAMQSGKPKEIAEkmvegrmkkftgeVSLTGQPFVMEPSKTVGQLLKEHNAEVTGFIRFEV 260

+L + + HV P L + +P AE + QP++++PS T+GQ ++ H V F+RFE

SBJCT: 207 lADLGRRLGQHVVGMAP--------------------LSVGSLDDEPGGEAE-------------TKMLSQPYLLDPSITLGQYVQPHGVSVVDFVRFEC 273

QUERY: 261 GEGIEKVETD 270

GEG + + +

SBJCT: 274 GEGEDAADAE 283

**Figure 3— Source data 1. Helicase superfamily II (ATPase domain), RNA helicase family and eIF4AIII subfamily.**

**Chordata**  76 **RDVIAQSQSGTGKTATFSISVLQCLDIQVRETQALILAPTRELAVQIQKGLLALGDYMNVQCHACIGGTNVGEDIRKLDYGQHVVAGTPGRVFDMIRRRSLRTRAIKMLVLDEADEMLNKGFKEQIYDVYRYLPPATQVVLISATL** 221*

**Chytridiomycot**  69 **HDVIAQAQSGTGKTATFSISILQSLDMSKKECQALILAPTRELAQQIQKVLIALGDYMQVECHACIGGTNVREDMRRLEAGVHVVVGTPGRVFDMINRRALRSDSIKMFVLDEADEMLSRGFKEQIYDVFQLLPPATQVVLLSATM** 214

**Neocallimastig**  67 **HDVIAQAQSGTGKTATFSISILQSIDISIRKCQALVLSPTRELAQQIQKVVLALGDYMDCKCHACIGGTNVRDDMKILEAGVHVVVGTPGRVWDMINRRALNTENIKMFVLDEADEMLSRGFKDQIYEVFQLLPPQTQVVLLSATM** 212

**Streptophyta**  20 **HDVIAQAQSGTGKTATFSISILQQIDTSIRECQALILAPTRELAQQIQKVVIALGDFMQAQCHACIGGTNVREDMRKLEAGVHVVVGTPGRVYDMISRRALRANNIKLFVLDEADEMLSRGFKDQIHDVFKLLPAEVQVILLSATM** 165

**Arthropoda**  78 **HDVIAQAQSGTGKTATFSISILQQIDTSINECQALILAPTRELAQQIQKVVIALGDFMNAQCHACIGGTNVRDDMRKLEQGVHVVVGTPGRVYDMINRRALRTNNIKMFILDEADEMLSRGFKDQIHDVFKLLPTEVQVNLLSATM** 223

**Basidiomycota**  69 **HDVIAQAQSGTGKTATFSISILQQLDLSIKGCQALILAPTRELAQQIQKVVIALGDYMSIECHACVGGTNVREDMAKLQEGVHVVVGTPGRVYDMINRRALRTDNIKIFCLDEADEMLSRGFKDQIYELFQLLPQETQVVLLSATM** 214

**Mollusca**  17 **RDVIAQAQSGTGKTATFSVAILEKIDLKLTKCQALVLAPTRELAQQIQKVVLALGDYMNAHCHACIGGTVVREDMRKLESGVHVVVGTPGRVFDMINRRALDPANIKMFVLDEADEMLSRGFKDQIYDVFRFMPPEIQVILLSATM** 162

**Priapulida**  103 **-DVIAQAQSGTGKTATFSIAILQQVDISVRECQALILAPTRELAQQIQKVVIALGDYMSAQCMACIGGTNVREDMRKLELGVHVIVGTPGRVYDMINRRSLRVDKIKMFVLDEADEMLSRGFKDQIYDVFRYLPSTIQVILLSATM** 247

**Annelida**  92 **HDVIAQAQSGTGKTATFSIAILQQLDMNINECQALVLAPTRELAQQIQKVVIALGDYMEAQCHACIGGTNVREDMAKLQSGVHVVVGTPGRVFDMINRRALQTRSIKIFVLDEADEMLSRGFKDQIYDVFQTLPQDIQVILLSATM** 237

**Mortierellomyc**  62 **RDVIAQAQSGTGKTATFSISILQSIDTSIRETQALVLAPTRELAIQIQSVILALGDYMNVQCHACIGGTSIGEDIRQLDHGQHVVVGTPGRVFDMIRRKNLRTRNIKMMVLDEADELLNQGFKDQIYDVYRYLPPSTQVVILSATL** 207

**Glaucocystophy**  82 **-DCIAQAQSGTGKTATFAIGVLQNIDTSLKECQALLLAPTRELAQQIQKVVIALGDYMSAQCHACIGGTNVREDMKRLESGVHIVVGTPGRVFDMIQRRALRTDYMKMFILDEADEMLSRGFKDQIYDVFKHMPSKIQVGLFSATM** 226

**Nemertea**  87 **HDVIAQAQSGTGKTATFAISILQKIDLSIKDCQALVLAPTRELAQQIQKVVIALGDYMNASCHACIGGTNVREDGRKLEEGKHVIVGTPGRVYDMIKRGHLRTDEMKMFVLDEADEMLSRGFKDQIYDVFRELPATVQVVLLSATM** 232

**Porifera**  100 **KDVIAQAQSGTGKTATFSIAILQNLIVEQRECQALVLAPTRELAQQIQKVVMALGDYMSAHCHACIGGTNVRDDIRRLETGVHIVVGTPGRVYDMLQRRSLDPRNIKMFVLDEADEMLSRGFKDQIYDVFRLLPSDAQVILLSATM** 245

**Entomophthorom**  59 **RDVIAQAQSGTGKTATFSISILQRIDTKVRECQALVLSPTRELATQIQSVVLALGDYMNVQCHACIGGTNVGEDTRKLEHGQHVVSGTPGRVYDMLKRKSLRTKHIKMLVLDEADELLNKGFKDQIYDVYRFLPPSTQVVLLSATL** 204

**Nematoda**  99 **RDVIAQAQSGTGKTATFSISILQSLDTQVRETQALVLSPTRELAQQIQKVILALGDYMNVQCHACIGGTNVGEDIRKLDYGQHVVSGTPGRVFDMIRRRNLRTRSIKMLVLDEADEMLNKGFKEQLYDIYRYLPPGAQVVLLSATL** 244

**Chlorophyta**  76 **-DVIQQAQSGTGKTATFCAGILQNLDYTLVECQALVLAPTRELAQQIEKVMRALGDYLNVKCHACVGGTSVREDTRILQSGVHVVVGTPGRVYDMLRRRALRADAIKMFVLDEADEMLSRGFKDQIYDIFQLLPPKIQVGVFSATL** 220

**Apusozoa**  96 **RDVIAQAQSGTGKTATFTISILQSVDTSLNETQALVLAPTRELATQIQSVMIALGDYMSTTVHACIGGTLVREDMRTLEQGIHIIVGTPGRVFDMINRRALRVDHLKMFVLDEADEMLSRGFKDQIYDIFCLLPSKVQVVLLSATM** 241

**Platyhelminthe**  66 **RDVIAQAQSGTGKTATLGISILQMLDTQLRETQALVLSPTRELASQIQKVILALGDYMNVQCHACYGGTNIGEDIRKLDYGQHVISGTPGRVFDMIRRRSLRTRAVKLFVLDEADEMLDKGFKEQIYDVYRYLPPGTQVVLLSATM** 211

**Brachiopoda**  129 **RDVIAQAQSGTGKTATFAIAILQQIDVKRPECQALVLAPTRELAQQIQKVVIALGDYLNAECHACIGGTNVREDMGRLSQGVHVVVGTPGRVYDMIQRRALDPRCIKLFVLDEADEMLSRGFKDQIYDVFQYMPKETQVILLSATM** 274

**Amoebozoa**  46 **-DLIAQAQSGTGKTATFTIGILQRLDFSIPDCQALILAPTRELAQQIQKVVIALGDYLNAKCHACIGGTRVSDDITKLRNGVHLVVGTPGRVYDMLCRNVLRPDRIKMFILDEADEMLSRGFKDQIYDIFQALPPRTQVGLFSATM** 190

**Hemichordata**  88 **RDVIAQAQSGTGKTATFSIAILQQLEITRMSSQALILAPTRELAQQIQKVVIALGDYMGAQCHACIGGTNVREDMRKLESGQHVVVGTPGRVHDMINRRALNVSDIKIFVLDEADEMLSRGFKDQIYDVFRLLPHSVQVILLSATM** 233

**Echinodermata**  63 **RDVIAQAQSGTGKTATFSISILQCLDTQVRETQALILSPTRELANQIQKVILALGDYMSVQCHSCIGGTNVGEDIRKLDFGQHVVSGTPGRVFDMIRRRNLRTRAIKMLVLDEADEMLNKGFKEQIYDVYRYLPPATQVVLFSATL** 208

**Bryozoa**  68 **RDVIAQAQSGTGKTATFSIASLQALDTQIRETQVLVLSPTRELAIQIQKVILALGDYMSVQCHGCIGGTNIGEDIRKLDYGQHIVSGTPGRVFDMIRRRNLRTRSIKMFILDEADEMLNKGFKEQIYDVYRYLPPATQVVLLSATL** 213

**Bacillariophyt**  79 **KDLIAQAQSGTGKTATFAIGTLARLDPKLRECQALILAPTRELAQQIQKVVLALGDYMDIQVHACVGGTAVRDDIRTLQAGVHVVVGTPGRVFDMINRRALRLDSIRQFFLDEADEMLSRGFKDQIYDIFKFLPETVQVCLFSATM** 224

**Stramenopiles**  71 **HDCIAQAQSGTGKTATFAISILQQIDSALRETQALILAPTRELAQQIVKVILAIGDYMGCLVHACVGGTAVRDDIRTLQSGVHIVVGTPGRVGDMINRRAFRTDSVKLFVLDEADEMLSRGFKDQIYDVFRFLPEKVQVALFSATM** 216

**Cryptophyta**  71 **RDTIAQAQSGTGKTAAFSIGCLQRIDLNEKDCQALLLAPTRELAQQIQKVVLALGDYMGITCHACIGGTNVRDDIRKVEAGQQVVVGTPGRVHDMINRRALRTDGMKIFVLDEADEMLSRGFKDQIYDVFKFLPSKVQVGLFSATM** 216

**Myzostomida**  60 **RDVIAQAQSGTGKTATFSISVLQAIDTQLRETQALVLSPTRELAVQIQKVILALGDYMSIQVHACIGGTNIGEDLKKLDYGQHVISGTPGRVIDMIKRRSLRTNSIKMLVLDEADEMLNKGFKEQIYDVYRYLPPATQVCLLSATM** 205

**Chromerida**  135 **HDTIGQAQSGTGKTATFAIAILQKIDYEIRECQALVLAPTRELAQQIQKVVLALGDYLKVKCHACVGGTAVRDDIQKLSQGVHMVVGTPGRVYDMIDKKYLRLDSMNLFVLDEADEMLSRGFKDQIYDVFRKLPSSIQVALFSATM** 280

**Rotifera**  64 **RDVIAQAQSGTGKTATFSISILQQLDMSIKSCQALILAPTRELAQQIQKVVLALGDFMGVTCHACVGGTNVKEDARKLEVGAQIIVGTPGRVSDMINRKALSPKTIKLFVLDEADEMLSRGFKEQIHDVFTKMPXHIQVILLSATM** 209

**position**  80 . 90 . 100 . 110 . 120 . 130 . 140 . 150 . 160 . 170 . 180 . 190 . 200 . 210 . 220

**_**

**_**

**_ __**

**__ __**

**__ __**

**__ __**

**__ __**

**___ __**

**___ __**

**___ __**

**___ __**

**_ ___ __**

**_ ___ __**

**_ ___ __**

**_ ___ __**

**_ ___ __**

**_ ___ __ _**

**_ ___ __ _ _**

**_ ___ _ __ _ _**

**_ ___ _ __ _ _**

**_ ___ _ __ _ _**

**_ ___ _ __ _ _**

**_ ___ _ __ ___**

**_ ___ _ __ ___**

**_ ___ _ __ ___**

**_ ___ _ __ ___**

**_ ___ _ __ ___**

**_ ___ _ _ __ ___**

**_ ___ _ _ __ _ ___**

**_ ___ _ _ __ _ ___**

**_ ___ _ _ __ _ ___**

**_ ___ _ _ __ _ ___**

**_ ___ _ _ __ _ ___**

**_ ___ _ _ __ __ _ ___**

**_ _ ___ _ _ _ _____ _ ___**

**_ _ ___ _ _ _ _ _____ _ ___**

**_ _ ___ _ _ _ _ _____ _ ___**

**_ _ ___ _ _ _ _ _ _____ _ ___**

**_ _ ___ __ _ _ _ _ _____ _ ___**

**__ _ ___ _ ___ _ _ _ _ _____ _ ___**

**__ _ ___ _ ___ _ _ _ _ _ _____ _ ___**

**__ _ ___ _ ___ _ _ _ _ _ _____ _ _ ___**

**__ _ ___ _ ___ _ _ _ _ _ _____ _ _ ___**

**__ __ ___ _ ___ _ _ _ _ _ _____ _ _ ___**

**__ __ ___ _ ___ _ _ _ _ _ _____ _ _ ___**

**__ __ ___ _ ___ _ _ _ _ _ _____ _ _ ____**

**__ __ ___ _ ___ _ _ _ _ _ _____ _ _ ____**

**__ __ ___ _ ___ _ _ ___ _ _ _____ _ _ ____**

**__ __ ___ _ ___ _ _ ___ _ _ _____ _ _ ____**

**__ __ ___ _ ___ _ _ ___ _ _ _______ _ ____**

**_ __ ______ _ ___ _ _ _ ___ _ _ _______ _ ____**

**Helicase SF II** ● ●● ●●●●●● ● ●●● ● ● ● ●●● ● ● ●●●●●●● ● ●●●●

**3ex7A_human**  76 **RDVIAQSQSGTGKTATFSISVLQCLDIQVRETQALILAPTRELAVQIQKGLLALGDYMNVQCHACIGGTNVGEDIRKLDYGQHVVAGTPGRVFDMIRRRSLRTRAIKMLVLDEADEMLNKGFKEQIYDVYRYLPPATQVVLISATL** 221*

**KND04363.1**  69 **HDVIAQAQSGTGKTATFSISILQSLDMSKKECQALILAPTRELAQQIQKVLIALGDYMQVECHACIGGTNVREDMRRLEAGVHVVVGTPGRVFDMINRRALRSDSIKMFVLDEADEMLSRGFKEQIYDVFQLLPPATQVVLLSATM** 214

**GT913350.1_EST**  67 **HDVIAQAQSGTGKTATFSISILQSIDISIRKCQALVLSPTRELAQQIQKVVLALGDYMDCKCHACIGGTNVRDDMKILEAGVHVVVGTPGRVWDMINRRALNTENIKMFVLDEADEMLSRGFKDQIYEVFQLLPPQTQVVLLSATM** 212

**AIZ74337.1**  20 **HDVIAQAQSGTGKTATFSISILQQIDTSIRECQALILAPTRELAQQIQKVVIALGDFMQAQCHACIGGTNVREDMRKLEAGVHVVVGTPGRVYDMISRRALRANNIKLFVLDEADEMLSRGFKDQIHDVFKLLPAEVQVILLSATM** 165

**XP_008557452.2**  78 **HDVIAQAQSGTGKTATFSISILQQIDTSINECQALILAPTRELAQQIQKVVIALGDFMNAQCHACIGGTNVRDDMRKLEQGVHVVVGTPGRVYDMINRRALRTNNIKMFILDEADEMLSRGFKDQIHDVFKLLPTEVQVNLLSATM** 223

**KIJ15730.1**  69 **HDVIAQAQSGTGKTATFSISILQQLDLSIKGCQALILAPTRELAQQIQKVVIALGDYMSIECHACVGGTNVREDMAKLQEGVHVVVGTPGRVYDMINRRALRTDNIKIFCLDEADEMLSRGFKDQIYELFQLLPQETQVVLLSATM** 214

**FK714841.1_EST**  17 **RDVIAQAQSGTGKTATFSVAILEKIDLKLTKCQALVLAPTRELAQQIQKVVLALGDYMNAHCHACIGGTVVREDMRKLESGVHVVVGTPGRVFDMINRRALDPANIKMFVLDEADEMLSRGFKDQIYDVFRFMPPEIQVILLSATM** 162

**XP_014666414.1**  103 **-DVIAQAQSGTGKTATFSIAILQQVDISVRECQALILAPTRELAQQIQKVVIALGDYMSAQCMACIGGTNVREDMRKLELGVHVIVGTPGRVYDMINRRSLRVDKIKMFVLDEADEMLSRGFKDQIYDVFRYLPSTIQVILLSATM** 247

**JZ396214.1_EST**  92 **HDVIAQAQSGTGKTATFSIAILQQLDMNINECQALVLAPTRELAQQIQKVVIALGDYMEAQCHACIGGTNVREDMAKLQSGVHVVVGTPGRVFDMINRRALQTRSIKIFVLDEADEMLSRGFKDQIYDVFQTLPQDIQVILLSATM** 237

**KFH66111.1**  62 **RDVIAQAQSGTGKTATFSISILQSIDTSIRETQALVLAPTRELAIQIQSVILALGDYMNVQCHACIGGTSIGEDIRQLDHGQHVVVGTPGRVFDMIRRKNLRTRNIKMMVLDEADELLNQGFKDQIYDVYRYLPPSTQVVILSATL** 207

**EC660356.1_EST**  82 **-DCIAQAQSGTGKTATFAIGVLQNIDTSLKECQALLLAPTRELAQQIQKVVIALGDYMSAQCHACIGGTNVREDMKRLESGVHIVVGTPGRVFDMIQRRALRTDYMKMFILDEADEMLSRGFKDQIYDVFKHMPSKIQVGLFSATM** 226

**JZ519882.1_EST**  87 **HDVIAQAQSGTGKTATFAISILQKIDLSIKDCQALVLAPTRELAQQIQKVVIALGDYMNASCHACIGGTNVREDGRKLEEGKHVIVGTPGRVYDMIKRGHLRTDEMKMFVLDEADEMLSRGFKDQIYDVFRELPATVQVVLLSATM** 232

**AM763992.1_EST**  100 **KDVIAQAQSGTGKTATFSIAILQNLIVEQRECQALVLAPTRELAQQIQKVVMALGDYMSAHCHACIGGTNVRDDIRRLETGVHIVVGTPGRVYDMLQRRSLDPRNIKMFVLDEADEMLSRGFKDQIYDVFRLLPSDAQVILLSATM** 245

**KXN74587.1**  59 **RDVIAQAQSGTGKTATFSISILQRIDTKVRECQALVLSPTRELATQIQSVVLALGDYMNVQCHACIGGTNVGEDTRKLEHGQHVVSGTPGRVYDMLKRKSLRTKHIKMLVLDEADELLNKGFKDQIYDVYRFLPPSTQVVLLSATL** 204

**KKA75822.1**  99 **RDVIAQAQSGTGKTATFSISILQSLDTQVRETQALVLSPTRELAQQIQKVILALGDYMNVQCHACIGGTNVGEDIRKLDYGQHVVSGTPGRVFDMIRRRNLRTRSIKMLVLDEADEMLNKGFKEQLYDIYRYLPPGAQVVLLSATL** 244

**XP_005845171.1**  76 **-DVIQQAQSGTGKTATFCAGILQNLDYTLVECQALVLAPTRELAQQIEKVMRALGDYLNVKCHACVGGTSVREDTRILQSGVHVVVGTPGRVYDMLRRRALRADAIKMFVLDEADEMLSRGFKDQIYDIFQLLPPKIQVGVFSATL** 220

**XP_013753804.1**  96 **RDVIAQAQSGTGKTATFTISILQSVDTSLNETQALVLAPTRELATQIQSVMIALGDYMSTTVHACIGGTLVREDMRTLEQGIHIIVGTPGRVFDMINRRALRVDHLKMFVLDEADEMLSRGFKDQIYDIFCLLPSKVQVVLLSATM** 241

**CCD77170.1**  66 **RDVIAQAQSGTGKTATLGISILQMLDTQLRETQALVLSPTRELASQIQKVILALGDYMNVQCHACYGGTNIGEDIRKLDYGQHVISGTPGRVFDMIRRRSLRTRAVKLFVLDEADEMLDKGFKEQIYDVYRYLPPGTQVVLLSATM** 211

**XP_013396865.1**  129 **RDVIAQAQSGTGKTATFAIAILQQIDVKRPECQALVLAPTRELAQQIQKVVIALGDYLNAECHACIGGTNVREDMGRLSQGVHVVVGTPGRVYDMIQRRALDPRCIKLFVLDEADEMLSRGFKDQIYDVFQYMPKETQVILLSATM** 274

**EC758385.1_EST**  46 **-DLIAQAQSGTGKTATFTIGILQRLDFSIPDCQALILAPTRELAQQIQKVVIALGDYLNAKCHACIGGTRVSDDITKLRNGVHLVVGTPGRVYDMLCRNVLRPDRIKMFILDEADEMLSRGFKDQIYDIFQALPPRTQVGLFSATM** 190

**XP_006825894.1**  88 **RDVIAQAQSGTGKTATFSIAILQQLEITRMSSQALILAPTRELAQQIQKVVIALGDYMGAQCHACIGGTNVREDMRKLESGQHVVVGTPGRVHDMINRRALNVSDIKIFVLDEADEMLSRGFKDQIYDVFRLLPHSVQVILLSATM** 233

**XP_785431.3**  63 **RDVIAQAQSGTGKTATFSISILQCLDTQVRETQALILSPTRELANQIQKVILALGDYMSVQCHSCIGGTNVGEDIRKLDFGQHVVSGTPGRVFDMIRRRNLRTRAIKMLVLDEADEMLNKGFKEQIYDVYRYLPPATQVVLFSATL** 208

**JZ489050.1_EST**  68 **RDVIAQAQSGTGKTATFSIASLQALDTQIRETQVLVLSPTRELAIQIQKVILALGDYMSVQCHGCIGGTNIGEDIRKLDYGQHIVSGTPGRVFDMIRRRNLRTRSIKMFILDEADEMLNKGFKEQIYDVYRYLPPATQVVLLSATL** 213

**XP_002186399.1**  79 **KDLIAQAQSGTGKTATFAIGTLARLDPKLRECQALILAPTRELAQQIQKVVLALGDYMDIQVHACVGGTAVRDDIRTLQAGVHVVVGTPGRVFDMINRRALRLDSIRQFFLDEADEMLSRGFKDQIYDIFKFLPETVQVCLFSATM** 224

**XP_008605124.1**  71 **HDCIAQAQSGTGKTATFAISILQQIDSALRETQALILAPTRELAQQIVKVILAIGDYMGCLVHACVGGTAVRDDIRTLQSGVHIVVGTPGRVGDMINRRAFRTDSVKLFVLDEADEMLSRGFKDQIYDVFRFLPEKVQVALFSATM** 216

**XP_005834643.1**  71 **RDTIAQAQSGTGKTAAFSIGCLQRIDLNEKDCQALLLAPTRELAQQIQKVVLALGDYMGITCHACIGGTNVRDDIRKVEAGQQVVVGTPGRVHDMINRRALRTDGMKIFVLDEADEMLSRGFKDQIYDVFKFLPSKVQVGLFSATM** 216

**FN424495.1_EST**  60 **RDVIAQAQSGTGKTATFSISVLQAIDTQLRETQALVLSPTRELAVQIQKVILALGDYMSIQVHACIGGTNIGEDLKKLDYGQHVISGTPGRVIDMIKRRSLRTNSIKMLVLDEADEMLNKGFKEQIYDVYRYLPPATQVCLLSATM** 205

**CEM14666.1**  135 **HDTIGQAQSGTGKTATFAIAILQKIDYEIRECQALVLAPTRELAQQIQKVVLALGDYLKVKCHACVGGTAVRDDIQKLSQGVHMVVGTPGRVYDMIDKKYLRLDSMNLFVLDEADEMLSRGFKDQIYDVFRKLPSSIQVALFSATM** 280

**FM919287.1_EST**  64 **RDVIAQAQSGTGKTATFSISILQQLDMSIKSCQALILAPTRELAQQIQKVVLALGDFMGVTCHACVGGTNVKEDARKLEVGAQIIVGTPGRVSDMINRKALSPKTIKLFVLDEADEMLSRGFKEQIHDVFTKMPXHIQVILLSATM** 209

**foreground (174196):**  **QGGVVAGDTGSGKSAAAAAAAAEAILRSGKGGQAVYVAPTSAAANDQAEEFAAFGGETGVKAGYFTGGGS KEQEAALSGGADVVVGSYGRVIDDI RL LLFSNWGYVVVDEAQEISN GF LRIRAKARAARKARQTVGFSGTL**

**RDLLLVDPM T TLVFLLPLLQLL R R PPILILV LRLLVQQLY RLRKLLP M LRIALLVR LD R RRRL RKV LLLTTPE LLRLL L RLDLLIL VHRMLD Q LLLRLLLLLPPDPRLLLMTA P**

**KNVIIQAE L IQYI LI K KRVIVIT RKV WI KE FK L I V VI S ET IIIA S I KISVI I D LK KII K L V IIAL**

**wt_res_freqs (40757): 1111222159399111211121111111112112112283111311113111111111211111113211 1111112111122231112211111 11 2111111134299411111 21 11111111111111211114181**

**221331121 1 821122213122 1 1 1115231 121611411 1211111 1 11111211 11 1 1111 111 1212532 42112 2 1212242 162111 1 11211211111111132136 4**

**11221121 1 1111 12 1 1221311 111 11 11 11 2 1 3 11 1 11 3121 1 1 11112 2 1 11 111 1 1 1 1213**

**insertions**

**deletions 421644332211112211111112413831121 11221482222222333333412222174321 111222261231661 111232221332933444333222222222**

**position**  80 . 90 . 100 . 110 . 120 . 130 . 140 . 150 . 160 . 170 . 180 . 190 . 200 . 210 . 220

**_**

**_**

**_**

**_**

**_**

**_**

**_**

**_ _**

**_ _**

**_ _**

**_ _**

**_ _**

**_ _ _**

**_ _ _**

**_ _ _**

**_ _ _**

**_ _ _**

**_ _ _ _**

**_ _ _ _**

**_ _ _ _**

**_ _ __ _ _**

**_ _ __ _ _**

**_ _ __ _ _**

**_ _ __ _ _**

**_ _ __ _ _**

**_ _ __ _ _ _**

**_ _ __ _ _ _**

**_ _ __ _ _ _**

**_ _ __ _ _ _ _**

**_ _ __ _ _ _ _**

**__ _ __ _ _ _ _**

**__ _ __ _ _ _ _**

**_ __ _ __ _ _ _ _**

**_ __ _ __ _ _ _ _**

**_ __ _ ___ _ _ _ _**

**_ __ _ ___ _ _ _ _**

**_ __ _ ___ _ _ _ _**

**_ _ _ __ __ ___ _ _ _ _**

**_ _ _ _ __ __ ___ _ _ _ _**

**_ _ _ _ __ __ ___ _ _ _ _ _**

**_ _ _ _ ___ __ ___ _ _ _ _ _**

**_ _ _ _ ___ __ _ ___ _ _ _ __ _**

**_ _ _ _ ___ __ _ ___ _ _ __ __ _**

**_ _ __ _ ___ _ __ _ ___ _ _ __ __ _ _**

**_ _ __ _ ___ _ __ _ ___ _ _ __ __ __ _**

**_ _ __ _ ___ _ __ _ ___ _ _ __ __ __ _**

**_ _ __ _ ___ _ __ _ _ ___ _ _ __ __ __ _**

**_ _ __ __ ___ __ __ _ _ ___ _ _ __ __ __ _**

**_ _ __ __ ___ __ __ _ _ ___ _ _ __ __ __ _**

**_ _ __ __ _ ___ __ __ _ _ _ ___ _ _ __ __ __ _ _**

**__ _ ___ _ __ __ _ _ _ _____ __ __ _ __ _ _ _ _______ _ _ _ __ __ ______ __ _ _ ___ ___ _**

**RNA helicases**  ●● ● ●●● ● ●● ●● ● ● ● ●●●●● ●● ●● ● ●● ● ● ● ●●●●●●● ● ● ● ●● ●● ●●●●●● ●● ● ● ●●● ●●● ●

**3ex7A_human**  76 **RDVIAQSQSGTGKTATFSISVLQCLDIQVRETQALILAPTRELAVQIQKGLLALGDYMNVQCHACIGGTNVGEDIRKLDYGQHVVAGTPGRVFDMIRRRSLRTRAIKMLVLDEADEMLNKGFKEQIYDVYRYLPPATQVVLISATL** 221*

**KND04363.1**  69 **HDVIAQAQSGTGKTATFSISILQSLDMSKKECQALILAPTRELAQQIQKVLIALGDYMQVECHACIGGTNVREDMRRLEAGVHVVVGTPGRVFDMINRRALRSDSIKMFVLDEADEMLSRGFKEQIYDVFQLLPPATQVVLLSATM** 214

**GT913350.1_EST**  67 **HDVIAQAQSGTGKTATFSISILQSIDISIRKCQALVLSPTRELAQQIQKVVLALGDYMDCKCHACIGGTNVRDDMKILEAGVHVVVGTPGRVWDMINRRALNTENIKMFVLDEADEMLSRGFKDQIYEVFQLLPPQTQVVLLSATM** 212

**AIZ74337.1**  20 **HDVIAQAQSGTGKTATFSISILQQIDTSIRECQALILAPTRELAQQIQKVVIALGDFMQAQCHACIGGTNVREDMRKLEAGVHVVVGTPGRVYDMISRRALRANNIKLFVLDEADEMLSRGFKDQIHDVFKLLPAEVQVILLSATM** 165

**XP_008557452.2**  78 **HDVIAQAQSGTGKTATFSISILQQIDTSINECQALILAPTRELAQQIQKVVIALGDFMNAQCHACIGGTNVRDDMRKLEQGVHVVVGTPGRVYDMINRRALRTNNIKMFILDEADEMLSRGFKDQIHDVFKLLPTEVQVNLLSATM** 223

**KIJ15730.1**  69 **HDVIAQAQSGTGKTATFSISILQQLDLSIKGCQALILAPTRELAQQIQKVVIALGDYMSIECHACVGGTNVREDMAKLQEGVHVVVGTPGRVYDMINRRALRTDNIKIFCLDEADEMLSRGFKDQIYELFQLLPQETQVVLLSATM** 214

**FK714841.1_EST**  17 **RDVIAQAQSGTGKTATFSVAILEKIDLKLTKCQALVLAPTRELAQQIQKVVLALGDYMNAHCHACIGGTVVREDMRKLESGVHVVVGTPGRVFDMINRRALDPANIKMFVLDEADEMLSRGFKDQIYDVFRFMPPEIQVILLSATM** 162

**XP_014666414.1**  103 **-DVIAQAQSGTGKTATFSIAILQQVDISVRECQALILAPTRELAQQIQKVVIALGDYMSAQCMACIGGTNVREDMRKLELGVHVIVGTPGRVYDMINRRSLRVDKIKMFVLDEADEMLSRGFKDQIYDVFRYLPSTIQVILLSATM** 247

**JZ396214.1_EST**  92 **HDVIAQAQSGTGKTATFSIAILQQLDMNINECQALVLAPTRELAQQIQKVVIALGDYMEAQCHACIGGTNVREDMAKLQSGVHVVVGTPGRVFDMINRRALQTRSIKIFVLDEADEMLSRGFKDQIYDVFQTLPQDIQVILLSATM** 237

**KFH66111.1**  62 **RDVIAQAQSGTGKTATFSISILQSIDTSIRETQALVLAPTRELAIQIQSVILALGDYMNVQCHACIGGTSIGEDIRQLDHGQHVVVGTPGRVFDMIRRKNLRTRNIKMMVLDEADELLNQGFKDQIYDVYRYLPPSTQVVILSATL** 207

**EC660356.1_EST**  82 **-DCIAQAQSGTGKTATFAIGVLQNIDTSLKECQALLLAPTRELAQQIQKVVIALGDYMSAQCHACIGGTNVREDMKRLESGVHIVVGTPGRVFDMIQRRALRTDYMKMFILDEADEMLSRGFKDQIYDVFKHMPSKIQVGLFSATM** 226

**JZ519882.1_EST**  87 **HDVIAQAQSGTGKTATFAISILQKIDLSIKDCQALVLAPTRELAQQIQKVVIALGDYMNASCHACIGGTNVREDGRKLEEGKHVIVGTPGRVYDMIKRGHLRTDEMKMFVLDEADEMLSRGFKDQIYDVFRELPATVQVVLLSATM** 232

**AM763992.1_EST**  100 **KDVIAQAQSGTGKTATFSIAILQNLIVEQRECQALVLAPTRELAQQIQKVVMALGDYMSAHCHACIGGTNVRDDIRRLETGVHIVVGTPGRVYDMLQRRSLDPRNIKMFVLDEADEMLSRGFKDQIYDVFRLLPSDAQVILLSATM** 245

**KXN74587.1**  59 **RDVIAQAQSGTGKTATFSISILQRIDTKVRECQALVLSPTRELATQIQSVVLALGDYMNVQCHACIGGTNVGEDTRKLEHGQHVVSGTPGRVYDMLKRKSLRTKHIKMLVLDEADELLNKGFKDQIYDVYRFLPPSTQVVLLSATL** 204

**KKA75822.1**  99 **RDVIAQAQSGTGKTATFSISILQSLDTQVRETQALVLSPTRELAQQIQKVILALGDYMNVQCHACIGGTNVGEDIRKLDYGQHVVSGTPGRVFDMIRRRNLRTRSIKMLVLDEADEMLNKGFKEQLYDIYRYLPPGAQVVLLSATL** 244

**XP_005845171.1**  76 **-DVIQQAQSGTGKTATFCAGILQNLDYTLVECQALVLAPTRELAQQIEKVMRALGDYLNVKCHACVGGTSVREDTRILQSGVHVVVGTPGRVYDMLRRRALRADAIKMFVLDEADEMLSRGFKDQIYDIFQLLPPKIQVGVFSATL** 220

**XP_013753804.1**  96 **RDVIAQAQSGTGKTATFTISILQSVDTSLNETQALVLAPTRELATQIQSVMIALGDYMSTTVHACIGGTLVREDMRTLEQGIHIIVGTPGRVFDMINRRALRVDHLKMFVLDEADEMLSRGFKDQIYDIFCLLPSKVQVVLLSATM** 241

**CCD77170.1**  66 **RDVIAQAQSGTGKTATLGISILQMLDTQLRETQALVLSPTRELASQIQKVILALGDYMNVQCHACYGGTNIGEDIRKLDYGQHVISGTPGRVFDMIRRRSLRTRAVKLFVLDEADEMLDKGFKEQIYDVYRYLPPGTQVVLLSATM** 211

**XP_013396865.1**  129 **RDVIAQAQSGTGKTATFAIAILQQIDVKRPECQALVLAPTRELAQQIQKVVIALGDYLNAECHACIGGTNVREDMGRLSQGVHVVVGTPGRVYDMIQRRALDPRCIKLFVLDEADEMLSRGFKDQIYDVFQYMPKETQVILLSATM** 274

**EC758385.1_EST**  46 **-DLIAQAQSGTGKTATFTIGILQRLDFSIPDCQALILAPTRELAQQIQKVVIALGDYLNAKCHACIGGTRVSDDITKLRNGVHLVVGTPGRVYDMLCRNVLRPDRIKMFILDEADEMLSRGFKDQIYDIFQALPPRTQVGLFSATM** 190

**XP_006825894.1**  88 **RDVIAQAQSGTGKTATFSIAILQQLEITRMSSQALILAPTRELAQQIQKVVIALGDYMGAQCHACIGGTNVREDMRKLESGQHVVVGTPGRVHDMINRRALNVSDIKIFVLDEADEMLSRGFKDQIYDVFRLLPHSVQVILLSATM** 233

**XP_785431.3**  63 **RDVIAQAQSGTGKTATFSISILQCLDTQVRETQALILSPTRELANQIQKVILALGDYMSVQCHSCIGGTNVGEDIRKLDFGQHVVSGTPGRVFDMIRRRNLRTRAIKMLVLDEADEMLNKGFKEQIYDVYRYLPPATQVVLFSATL** 208

**JZ489050.1_EST**  68 **RDVIAQAQSGTGKTATFSIASLQALDTQIRETQVLVLSPTRELAIQIQKVILALGDYMSVQCHGCIGGTNIGEDIRKLDYGQHIVSGTPGRVFDMIRRRNLRTRSIKMFILDEADEMLNKGFKEQIYDVYRYLPPATQVVLLSATL** 213

**XP_002186399.1**  79 **KDLIAQAQSGTGKTATFAIGTLARLDPKLRECQALILAPTRELAQQIQKVVLALGDYMDIQVHACVGGTAVRDDIRTLQAGVHVVVGTPGRVFDMINRRALRLDSIRQFFLDEADEMLSRGFKDQIYDIFKFLPETVQVCLFSATM** 224

**XP_008605124.1**  71 **HDCIAQAQSGTGKTATFAISILQQIDSALRETQALILAPTRELAQQIVKVILAIGDYMGCLVHACVGGTAVRDDIRTLQSGVHIVVGTPGRVGDMINRRAFRTDSVKLFVLDEADEMLSRGFKDQIYDVFRFLPEKVQVALFSATM** 216

**XP_005834643.1**  71 **RDTIAQAQSGTGKTAAFSIGCLQRIDLNEKDCQALLLAPTRELAQQIQKVVLALGDYMGITCHACIGGTNVRDDIRKVEAGQQVVVGTPGRVHDMINRRALRTDGMKIFVLDEADEMLSRGFKDQIYDVFKFLPSKVQVGLFSATM** 216

**FN424495.1_EST**  60 **RDVIAQAQSGTGKTATFSISVLQAIDTQLRETQALVLSPTRELAVQIQKVILALGDYMSIQVHACIGGTNIGEDLKKLDYGQHVISGTPGRVIDMIKRRSLRTNSIKMLVLDEADEMLNKGFKEQIYDVYRYLPPATQVCLLSATM** 205

**CEM14666.1**  135 **HDTIGQAQSGTGKTATFAIAILQKIDYEIRECQALVLAPTRELAQQIQKVVLALGDYLKVKCHACVGGTAVRDDIQKLSQGVHMVVGTPGRVYDMIDKKYLRLDSMNLFVLDEADEMLSRGFKDQIYDVFRKLPSSIQVALFSATM** 280

**FM919287.1_EST**  64 **RDVIAQAQSGTGKTATFSISILQQLDMSIKSCQALILAPTRELAQQIQKVVLALGDFMGVTCHACVGGTNVKEDARKLEVGAQIIVGTPGRVSDMINRKALSPKTIKLFVLDEADEMLSRGFKEQIHDVFTKMPXHIQVILLSATM** 209

**foreground (40286):**  **KDVVGQAQSGSGKTAAYGIPAIEQVDPSKNGTQAIVVAPTRELAQQTADEAEAYGKHTGVKSACCYGGASYGEDAEALEKGADVVVGTPGRVIDHIENGAFSLSSVETFVLDEADEMLDLGFEDDVEEVFAATPADRQTLLFSATF**

**R LLAISRT T L FLL MLQHLL RPREPRVLILV L IEKVLRKLSRFMNLRILLLI VPMRPQLRR RR PHILIA LL MLRRRKLR RRLRMLII RL EM LPQLRRLLRLL PK VMM M**

**IIVR K V L RI K K I IT I V E IK FA YL I VVVIV TDIKREIKK K VQ I I L KKKTIK KQIKIV S IEEIKKIIKQV KQ L**

**wt_res_freqs (6179): 29414284195999471127112112111121351413999998191112111132112221111399121111112712613246499991183221311162131118799892775188213221111116116854689991**

**5 2231118 4 3 825 153151 11114217561 1 51122124111113211111 111116121 22 225325 63 13131141 11211511 51 14 12221113113 11 111 3**

**1311 1 1 2 22 1 1 1 11 1 3 2 11 22 22 2 21122 112111211 1 31 1 1 3 111111 112211 1 12141161111 21 2**

**insertions**

**deletions 26554433333222222211111111 1 1 11 12 1113 111122223333455555666667776**

**background (133910):**  **QDVVVAADTGSGKSTTYALPALEALERSGRGGQAVVVAPTRALANQVAEEFAEFGGGCGIAAGYFTGSGS KEREAILSGDADVVVGTPG LISDILRD LLF NVGVVVLDEAQEFGNPGGALRPLALARAARKAAQRVLLSGTL**

**MGGLLVDPM T TLVALAALA L R K RPILILV LILAVQWLVRRLRKLPPEM LRIALLVR LD RQ RRLR RKTRLLIASYQ LREL L L KLDLLIV VHRLK QR L LKLLLLLPDPRLLGMTA P**

**RN II GEV L IQ I LI K KRVILIS RSV DQY K AL L V V HS E IILT E DWS I I IS D RRI K NL IIA**

**wt_res_freqs (34577): 1111221159399111112122112112112111132272125213113111111111211111112111 1111111111122231521 41111111 211 111133199411111111111121111111111111244271**

**111441131 1 721211211 2 1 1 1115221 1111112111121111112 11111211 11 11 1121 11111221211 2112 1 1 1113242 17211 11 1 11211111111131146 5**

**11 22 211 2 11 1 12 1 1221121 111 111 1 11 2 3 1 12 1 3112 2 111 2 3 11 1 111 1 11 121**

**position**  80 . 90 . 100 . 110 . 120 . 130 . 140 . 150 . 160 . 170 . 180 . 190 . 200 . 210 . 220

**_**

**_**

**_**

**_**

**_**

**_**

**_**

**_**

**_**

**_**

**_**

**_**

**_**

**_**

**_**

**_**

**_**

**_**

**_**

**_ _**

**_ _**

**_ _**

**_ _**

**_ _**

**_ _**

**_ _**

**_ _ _**

**_ _ _**

**_ _ _ _**

**_ _ _ _**

**_ _ _ _ _ _**

**_ _ _ _ _ _**

**_ _ _ _ _ _ _ _**

**_ _ _ _ _ _ _ _**

**_ _ _ _ _ _ _ _**

**_ _ _ _ _ _ _ _ _**

**_ _ _ _ _ _ _ _ _ _ _**

**_ _ _ _ _ _ _ _ _ _ _**

**_ _ _ _ _ _ _ _ _ _ _**

**_ _ _ _ _ ___ _ __ _ _ _ _**

**_ _ _ _ _ _ ___ _ __ _ _ __ _**

**_ _ _ __ _ _ ___ _ __ _ _ _ __ _**

**_ _ _ _ _ ___ _ _ ___ __ __ _ _ _ __ _ _**

**_ _ _ _ _ ___ _ __ ___ _ __ __ _ _ _ _ __ _ _**

**_ _ _ _ _ _ ___ _ __ ____ _ __ __ _ _ _ _ __ _ _ _**

**_ _ _ _ _ _ ___ _ __ ____ _ __ __ _ _ _ _ __ _ _ _**

**_ _ _ _ _ _ ___ _ __ ____ _ __ __ _ _ _ _ __ _ _ _**

**_ _ _ _ _ _ ___ _ __ ____ _ __ __ _ _ _ _ __ _ _ _**

**_ _ _ _ _ _ ___ _ __ ____ _ __ __ _ _ _ _ __ _ _ _**

**_ _ _ _ _ _ ___ _ __ ____ _ __ __ _ _ _ _ __ _ _ _**

**_ _ __ _ __ _ _ _ _ _ ___ _ ____ ______ _ _____ ___ __ _ _ _ __ _ __ ___ ___ _ __ _ _ __ _ _**

**eIF4AIII subfamily**  ● ● ●● ● ●● ● ● ● ● ● ●●● ● ●●●● ●●●●●● ● ●●●●● ●●● ●● ● ● ● ●● ● ●● ●●● ●●● ● ●● ● ● ●● ● ●

**3ex7A_human**  76 **RDVIAQSQSGTGKTATFSISVLQCLDIQVRETQALILAPTRELAVQIQKGLLALGDYMNVQCHACIGGTNVGEDIRKLDYGQHVVAGTPGRVFDMIRRRSLRTRAIKMLVLDEADEMLNKGFKEQIYDVYRYLPPATQVVLISATL** 221*

**KND04363.1**  69 **HDVIAQAQSGTGKTATFSISILQSLDMSKKECQALILAPTRELAQQIQKVLIALGDYMQVECHACIGGTNVREDMRRLEAGVHVVVGTPGRVFDMINRRALRSDSIKMFVLDEADEMLSRGFKEQIYDVFQLLPPATQVVLLSATM** 214

**GT913350.1_EST**  67 **HDVIAQAQSGTGKTATFSISILQSIDISIRKCQALVLSPTRELAQQIQKVVLALGDYMDCKCHACIGGTNVRDDMKILEAGVHVVVGTPGRVWDMINRRALNTENIKMFVLDEADEMLSRGFKDQIYEVFQLLPPQTQVVLLSATM** 212

**AIZ74337.1**  20 **HDVIAQAQSGTGKTATFSISILQQIDTSIRECQALILAPTRELAQQIQKVVIALGDFMQAQCHACIGGTNVREDMRKLEAGVHVVVGTPGRVYDMISRRALRANNIKLFVLDEADEMLSRGFKDQIHDVFKLLPAEVQVILLSATM** 165

**XP_008557452.2**  78 **HDVIAQAQSGTGKTATFSISILQQIDTSINECQALILAPTRELAQQIQKVVIALGDFMNAQCHACIGGTNVRDDMRKLEQGVHVVVGTPGRVYDMINRRALRTNNIKMFILDEADEMLSRGFKDQIHDVFKLLPTEVQVNLLSATM** 223

**KIJ15730.1**  69 **HDVIAQAQSGTGKTATFSISILQQLDLSIKGCQALILAPTRELAQQIQKVVIALGDYMSIECHACVGGTNVREDMAKLQEGVHVVVGTPGRVYDMINRRALRTDNIKIFCLDEADEMLSRGFKDQIYELFQLLPQETQVVLLSATM** 214

**FK714841.1_EST**  17 **RDVIAQAQSGTGKTATFSVAILEKIDLKLTKCQALVLAPTRELAQQIQKVVLALGDYMNAHCHACIGGTVVREDMRKLESGVHVVVGTPGRVFDMINRRALDPANIKMFVLDEADEMLSRGFKDQIYDVFRFMPPEIQVILLSATM** 162

**XP_014666414.1**  103 **-DVIAQAQSGTGKTATFSIAILQQVDISVRECQALILAPTRELAQQIQKVVIALGDYMSAQCMACIGGTNVREDMRKLELGVHVIVGTPGRVYDMINRRSLRVDKIKMFVLDEADEMLSRGFKDQIYDVFRYLPSTIQVILLSATM** 247

**JZ396214.1_EST**  92 **HDVIAQAQSGTGKTATFSIAILQQLDMNINECQALVLAPTRELAQQIQKVVIALGDYMEAQCHACIGGTNVREDMAKLQSGVHVVVGTPGRVFDMINRRALQTRSIKIFVLDEADEMLSRGFKDQIYDVFQTLPQDIQVILLSATM** 237

**KFH66111.1**  62 **RDVIAQAQSGTGKTATFSISILQSIDTSIRETQALVLAPTRELAIQIQSVILALGDYMNVQCHACIGGTSIGEDIRQLDHGQHVVVGTPGRVFDMIRRKNLRTRNIKMMVLDEADELLNQGFKDQIYDVYRYLPPSTQVVILSATL** 207

**EC660356.1_EST**  82 **-DCIAQAQSGTGKTATFAIGVLQNIDTSLKECQALLLAPTRELAQQIQKVVIALGDYMSAQCHACIGGTNVREDMKRLESGVHIVVGTPGRVFDMIQRRALRTDYMKMFILDEADEMLSRGFKDQIYDVFKHMPSKIQVGLFSATM** 226

**JZ519882.1_EST**  87 **HDVIAQAQSGTGKTATFAISILQKIDLSIKDCQALVLAPTRELAQQIQKVVIALGDYMNASCHACIGGTNVREDGRKLEEGKHVIVGTPGRVYDMIKRGHLRTDEMKMFVLDEADEMLSRGFKDQIYDVFRELPATVQVVLLSATM** 232

**AM763992.1_EST**  100 **KDVIAQAQSGTGKTATFSIAILQNLIVEQRECQALVLAPTRELAQQIQKVVMALGDYMSAHCHACIGGTNVRDDIRRLETGVHIVVGTPGRVYDMLQRRSLDPRNIKMFVLDEADEMLSRGFKDQIYDVFRLLPSDAQVILLSATM** 245

**KXN74587.1**  59 **RDVIAQAQSGTGKTATFSISILQRIDTKVRECQALVLSPTRELATQIQSVVLALGDYMNVQCHACIGGTNVGEDTRKLEHGQHVVSGTPGRVYDMLKRKSLRTKHIKMLVLDEADELLNKGFKDQIYDVYRFLPPSTQVVLLSATL** 204

**KKA75822.1**  99 **RDVIAQAQSGTGKTATFSISILQSLDTQVRETQALVLSPTRELAQQIQKVILALGDYMNVQCHACIGGTNVGEDIRKLDYGQHVVSGTPGRVFDMIRRRNLRTRSIKMLVLDEADEMLNKGFKEQLYDIYRYLPPGAQVVLLSATL** 244

**XP_005845171.1**  76 **-DVIQQAQSGTGKTATFCAGILQNLDYTLVECQALVLAPTRELAQQIEKVMRALGDYLNVKCHACVGGTSVREDTRILQSGVHVVVGTPGRVYDMLRRRALRADAIKMFVLDEADEMLSRGFKDQIYDIFQLLPPKIQVGVFSATL** 220

**XP_013753804.1**  96 **RDVIAQAQSGTGKTATFTISILQSVDTSLNETQALVLAPTRELATQIQSVMIALGDYMSTTVHACIGGTLVREDMRTLEQGIHIIVGTPGRVFDMINRRALRVDHLKMFVLDEADEMLSRGFKDQIYDIFCLLPSKVQVVLLSATM** 241

**CCD77170.1**  66 **RDVIAQAQSGTGKTATLGISILQMLDTQLRETQALVLSPTRELASQIQKVILALGDYMNVQCHACYGGTNIGEDIRKLDYGQHVISGTPGRVFDMIRRRSLRTRAVKLFVLDEADEMLDKGFKEQIYDVYRYLPPGTQVVLLSATM** 211

**XP_013396865.1**  129 **RDVIAQAQSGTGKTATFAIAILQQIDVKRPECQALVLAPTRELAQQIQKVVIALGDYLNAECHACIGGTNVREDMGRLSQGVHVVVGTPGRVYDMIQRRALDPRCIKLFVLDEADEMLSRGFKDQIYDVFQYMPKETQVILLSATM** 274

**EC758385.1_EST**  46 **-DLIAQAQSGTGKTATFTIGILQRLDFSIPDCQALILAPTRELAQQIQKVVIALGDYLNAKCHACIGGTRVSDDITKLRNGVHLVVGTPGRVYDMLCRNVLRPDRIKMFILDEADEMLSRGFKDQIYDIFQALPPRTQVGLFSATM** 190

**XP_006825894.1**  88 **RDVIAQAQSGTGKTATFSIAILQQLEITRMSSQALILAPTRELAQQIQKVVIALGDYMGAQCHACIGGTNVREDMRKLESGQHVVVGTPGRVHDMINRRALNVSDIKIFVLDEADEMLSRGFKDQIYDVFRLLPHSVQVILLSATM** 233

**XP_785431.3**  63 **RDVIAQAQSGTGKTATFSISILQCLDTQVRETQALILSPTRELANQIQKVILALGDYMSVQCHSCIGGTNVGEDIRKLDFGQHVVSGTPGRVFDMIRRRNLRTRAIKMLVLDEADEMLNKGFKEQIYDVYRYLPPATQVVLFSATL** 208

**JZ489050.1_EST**  68 **RDVIAQAQSGTGKTATFSIASLQALDTQIRETQVLVLSPTRELAIQIQKVILALGDYMSVQCHGCIGGTNIGEDIRKLDYGQHIVSGTPGRVFDMIRRRNLRTRSIKMFILDEADEMLNKGFKEQIYDVYRYLPPATQVVLLSATL** 213

**XP_002186399.1**  79 **KDLIAQAQSGTGKTATFAIGTLARLDPKLRECQALILAPTRELAQQIQKVVLALGDYMDIQVHACVGGTAVRDDIRTLQAGVHVVVGTPGRVFDMINRRALRLDSIRQFFLDEADEMLSRGFKDQIYDIFKFLPETVQVCLFSATM** 224

**XP_008605124.1**  71 **HDCIAQAQSGTGKTATFAISILQQIDSALRETQALILAPTRELAQQIVKVILAIGDYMGCLVHACVGGTAVRDDIRTLQSGVHIVVGTPGRVGDMINRRAFRTDSVKLFVLDEADEMLSRGFKDQIYDVFRFLPEKVQVALFSATM** 216

**XP_005834643.1**  71 **RDTIAQAQSGTGKTAAFSIGCLQRIDLNEKDCQALLLAPTRELAQQIQKVVLALGDYMGITCHACIGGTNVRDDIRKVEAGQQVVVGTPGRVHDMINRRALRTDGMKIFVLDEADEMLSRGFKDQIYDVFKFLPSKVQVGLFSATM** 216

**FN424495.1_EST**  60 **RDVIAQAQSGTGKTATFSISVLQAIDTQLRETQALVLSPTRELAVQIQKVILALGDYMSIQVHACIGGTNIGEDLKKLDYGQHVISGTPGRVIDMIKRRSLRTNSIKMLVLDEADEMLNKGFKEQIYDVYRYLPPATQVCLLSATM** 205

**CEM14666.1**  135 **HDTIGQAQSGTGKTATFAIAILQKIDYEIRECQALVLAPTRELAQQIQKVVLALGDYLKVKCHACVGGTAVRDDIQKLSQGVHMVVGTPGRVYDMIDKKYLRLDSMNLFVLDEADEMLSRGFKDQIYDVFRKLPSSIQVALFSATM** 280

**FM919287.1_EST**  64 **RDVIAQAQSGTGKTATFSISILQQLDMSIKSCQALILAPTRELAQQIQKVVLALGDFMGVTCHACVGGTNVKEDARKLEVGAQIIVGTPGRVSDMINRKALSPKTIKLFVLDEADEMLSRGFKEQIHDVFTKMPXHIQVILLSATM** 209

**foreground (2998):**  **RDVIAQAQSGTGKTATFCSGVCQQVDTGVKECQALVLAPTRELATQTEKVVRAIGDYLGAQCHACVGGTSVGDDQKKLSAGQQVVSGTPGRVYDMINRQALRTDAIKLFVLDEADEMLSKGFKDQIYDVYQYLPSATQVGVFSATL**

**H Q SMISLSIL KL YNLVQV V I S Q IQ ML L FMNIKV I KNIRE MRI QY VHI V F LR RV KPRY MLI S NR E IFRL PKL ILL M**

**AIA I SIR T II V A I ES C K T H K QEI V I**

**wt_res_freqs (467): 69896999999999888223219419312255989496999999191389429198632134999399857219214911911792999999199619229654189267999899997199869988434298113992229893**

**2 2 11141358 14 213111 1 4 2 6 85 33 7 243133 6 13167 162 21 681 6 5 33 51 1231 531 1 17 2 5643 421 174 5**

**151 4 213 2 21 6 1 3 32 1 1 1 1 1 112 4 1**

**insertions 2**

**deletions 4111111119987765554333221114 4 2 111111223678111111122222233333444444**

**background (37288):**  **KDVIGQAQTGTGKTAAYAIPAVQKIDAEGNGVQAVVVAPTRELAQQTAKVVEALGKYLGVKVAAVVGGTSYGEEAAALEKGAQVVVGTPGRVIDHIERRALSLSSIKTFVIDEADELLDLGFEDQVEEIVAATPADRQTLLFSATF**

**R LLVISR S L FLL MLEHLLPRPR PRVLILS L IYDEIRKYSRFTNIRILLLI VNMKPQLRR RR PHILIA LL LMRNGKFK KRLRILIL RM EM LPDLRRLLKLL PK MM M**

**IVAR K V LI R EKRK I I IT I VSQ LK FA H L SVVIY ADIER IKK K VD I I LKKKTID DQVEVV IEEIKK IEQI KQ L**

**wt_res_freqs (5712): 29435284994999471128112121111121251413999998191112111333122122113299121111112712611246399991183222113162112118199891276188212221611116116855689991**

**5 231111 5 3 835 1521521111 4217561 1 41121121111112311111 211117111 22 226335 63 41113111 11211517 56 15 12421113113 11 11 2**

**1131 2 1 22 2 1111 1 1 11 1 311 21 22 1 3 11223 11211 211 2 33 2 1 3111112 113111 121311 1111 21 2**

**position**  80 . 90 . 100 . 110 . 120 . 130 . 140 . 150 . 160 . 170 . 180 . 190 . 200 . 210 . 220

**Figure 3— Source data 1. eIF4AIII C-terminal domain**

**_**

**Chordata**  262 **EWKFDTLCDLYDTLTITQAVIFCNTKRKVDWLTEKMREANFTVSSMHGDMPQKERESIMKEFRSGASRVLISTDVWARGLDVPQVSLIINYDLPNNRELYIHRIGRSGRYGRKGVAINFVKNDDIRILRDIEQ** 394*

**Arthropoda**  265 **KYKDVYLVHILNELAGNSFMIFCSTCNNTVKTALMLRALGLAAIPLHGQMSQNKRLAALNKFKAKNRSILISTDVASRGLDIPHVDVVVNFDIPTHSKDYIHRVGRTARAGRSGKAITLVSQYDIELYQRIEH** 397

**Nematoda**  233 **EWKFDCLCDLYNVVNVTQAVIFCNTRRKVDTLTEKMTENQFTVSCLHGDMDQAERDTIMREFRSGSSRVLITTDILARGIDVQQVSLVINYDLPSNRENYIHRIGRSGRFGRKGVAINFVTENDARQLKEIES** 365

**Ascomycota**  322 **RGKERKLLELLKKYKNEKVLIFALYKKEAARVERNLKYNGYNVAAIHGDLSQQQRTQALNEFKSGKSNLLLATDVAARGLDIPNVKTVINLTFPLTVEDYVHRIGRTGRAGQTGTAHTLFTEQEKHLAGGLVN** 454

**Streptophyta**  245 **EWKLETLCDLYETLAITQSVIFVNTRRKVDWLTDKMRSRDHTVSATHGDMDQNTRDIIMREFRSGSSRVLITTDLLARGIDVQQVSLVINFDLPTQPENYLHRIGRSGRFGRKGVAINFMTSEDERMMADIQR** 377

**Amoebozoa**  221 **KYRDGYLISILKETEGKTIIIFTMKCSGCTKLVMMLRQMGYAAIPLHGKMSQQKRLIALEKFKSGKRGILVATDVASRGLDIPNVDIVINYDCPLEPKDYVHRVGRTARAGKSGYAITLVTQYSIELYQRIET** 353

**position**  . 270 . 280 . 290 . 300 . 310 . 320 . 330 . 340 . 350 . 360 . 370 . 380 . 390

**_**

**_ _**

**_ _ _**

**_ _ _**

**_ _ _**

**_ _ _**

**_ _ _**

**_ _ _**

**_ _ _**

**_ _ _**

**_ _ _**

**_ _ _**

**_ _ _**

**_ _ _**

**_ _ _ _**

**_ _ _ _**

**_ _ _ _**

**_ _ _ _**

**_ _ _ _**

**_ _ _ _**

**_ _ _ _**

**_ _ _ _**

**_ _ __ _**

**_ _ __ _**

**_ _ __ _**

**_ _ _ __ _**

**_ _ _ __ _**

**_ _ _ __ _**

**_ _ _ __ _**

**_ _ _ __ _**

**_ _ _ __ _**

**_ _ _ __ _**

**_ _ _ __ _**

**_ _ _ __ _**

**_ _ _ _ _ __ _**

**_ _ _ _ _ __ _**

**_ _ _ _ _ _ _ _ __ _**

**__ _ _ _ _ _ _ _ _ __ _**

**___ _ _ _ _ _ _ _ _ _ __ _**

**___ _ _ _ __ _ _ _ _ _ __ _**

**___ _ _ _ _ __ _ _ _ _ _ __ _**

**___ _ _ _ _ __ _ _ _ _ _ __ _**

**_ ___ _ _ _ _ __ _ _ _ _ _ __ _**

**_ ___ _ _ _ _ __ _ ___ _ _ __ _**

**_ _ ___ _ _ _ _ __ _ ___ __ _ __ _**

**_ _ _ ___ _ _ _ _ __ _ ____ __ _ __ _ _**

**_ _ _ ___ _ _ _ _ ___ _ ____ __ _ __ _ _**

**_ _ _ ___ _ _ _ _ ___ _ ____ __ _ __ _ _**

**_ _ _ ___ _ _ _ _ ___ _ ____ __ _ __ _ _**

**_ _ _ ___ _ _ _ _ _ _ _____ ____ _ __ _ __ _ _ _**

**_ _ _ ___ _ _ _ _ _ _ _____ ____ _ __ _ __ _ _ _**

**Helicase SF II**  ● ● ● ●●● ● ● ● ● ● ● ●●●●● ●●●● ● ●● ● ●● ● ● ●

**3EX7_HUMAN**  262 **EWKFDTLCDLYDTLTITQAVIFCNTKRKVDWLTEKMREANFTVSSMHGDMPQKERESIMKEFRSGASRVLISTDVWARGLDVPQVSLIINYDLPNNRELYIHRIGRSGRYGRKGVAINFVKNDDIRILRDIEQ** 394*

**Q8MRB7_DROME**  265 **KYKDVYLVHILNELAGNSFMIFCSTCNNTVKTALMLRALGLAAIPLHGQMSQNKRLAALNKFKAKNRSILISTDVASRGLDIPHVDVVVNFDIPTHSKDYIHRVGRTARAGRSGKAITLVSQYDIELYQRIEH** 397

**IF4A_CAEEL**  233 **EWKFDCLCDLYNVVNVTQAVIFCNTRRKVDTLTEKMTENQFTVSCLHGDMDQAERDTIMREFRSGSSRVLITTDILARGIDVQQVSLVINYDLPSNRENYIHRIGRSGRFGRKGVAINFVTENDARQLKEIES** 365

**DBP3_YEAST**  322 **RGKERKLLELLKKYKNEKVLIFALYKKEAARVERNLKYNGYNVAAIHGDLSQQQRTQALNEFKSGKSNLLLATDVAARGLDIPNVKTVINLTFPLTVEDYVHRIGRTGRAGQTGTAHTLFTEQEKHLAGGLVN** 454

**IF4A3_ARATH**  245 **EWKLETLCDLYETLAITQSVIFVNTRRKVDWLTDKMRSRDHTVSATHGDMDQNTRDIIMREFRSGSSRVLITTDLLARGIDVQQVSLVINFDLPTQPENYLHRIGRSGRFGRKGVAINFMTSEDERMMADIQR** 377

**C4M5I5_ENTHI**  221 **KYRDGYLISILKETEGKTIIIFTMKCSGCTKLVMMLRQMGYAAIPLHGKMSQQKRLIALEKFKSGKRGILVATDVASRGLDIPNVDIVINYDCPLEPKDYVHRVGRTARAGKSGYAITLVTQYSIELYQRIET** 353

**foreground (114244):**  **EGKIAAVVDIVEEEEGGQAIVFCNSKDTADEVADLLKARGYSAGAYDGGTSQADKDAAVDAFNAGEVDVVVATNAGGEGVNVPGASTVVNYGADKSLASYVQAAGRAGRAGQTGTCYTYYSEGDEEIIEAIEE**

**ERLELLLELLRKLR EPILL VPRIRELERLEK RKL LPILVLHSDMP KQRQRLMRR RE KLRILLS DLLER LDL DLRLIIILEPPWDPEDLLHLI VH I RPSKVLLLLDPEE RMLRRLQR**

**D D II IK K RV I TQTVK V I E E IRVARI ASLD EE EKILKK KN IK IIG IIS I I NIDV HFDL RN S I RV TA KK EAIIFIT D KL KK K**

**wt_res_freqs (19887): 1161121111112113111346112211221121161114111111152122111111111611521131447121219121412116222111121113171189359162151111111113211111222**

**11211512241111 11141 111111131411 111 11111341121 116111111 21 1112612 21112 352 111114111141121121212 11 1 1111312311113 11211311**

**1 1 11 21 1 13 3 11211 1 1 2 1 212111 1121 13 212211 11 11 121 111 3 3 1111 1142 12 1 1 31 21 11 1221211 1 11 11 1**

**insertions 4**

**deletions 211117543221453 33321 22121 75 11 11 112236791111222**

**position**  . 270 . 280 . 290 . 300 . 310 . 320 . 330 . 340 . 350 . 360 . 370 . 380 . 390

**_**

**_**

**_**

**_**

**_**

**_**

**_**

**_**

**_**

**_**

**_**

**_**

**_ _**

**_ _**

**_ _**

**_ _**

**_ _**

**_ _**

**_ _**

**_ _ _**

**_ _ _**

**_ _ _**

**_ _ _**

**_ _ _ _**

**_ _ _ _ _**

**_ _ _ __ _**

**_ _ _ __ _**

**_ _ _ _ __ _**

**_ _ _ _ _ __ _**

**_ _ _ _ _ __ _**

**_ _ _ _ _ __ _**

**_ _ _ _ _ _ __ _**

**_ _ _ _ _ _ __ _**

**_ _ __ _ _ _ __ _**

**_ _ __ _ _ _ __ _**

**_ _ __ _ _ _ __ _ _**

**_ _ __ _ _ _ __ _ _**

**_ _ __ _ _ _ __ _ _**

**_ _ __ _ _ _ __ _ _**

**_ __ __ _ _ _ _ __ _ _**

**_ __ __ _ _ _ _ __ _ _ _ _**

**_ __ __ _ _ _ ____ _ _ _ _**

**_ __ __ _ _ _ ____ _ _ _ _**

**_ _ __ __ _ _ _ ____ __ _ _ _ _**

**_ _ __ __ _ _ _ ____ __ _ _ _ _**

**_ _ _ __ __ _ _ _ ____ __ _ _ _ _**

**_ _ _ _____ _ _ _ ____ __ _ _ _ _**

**_ _ _ _____ _ _ _ _ ____ __ _ _ _ _**

**_ _ _ _ _____ ___ _ _ _ ____ __ _ _ _ _ _**

**_ _ _ _ _ _____ ___ _ _ __ _ _____ __ _ _ _ _ _**

**_ _ _ _ _ _____ ___ _ _ __ _ _____ __ _ _ _ _ _**

**RNA helicases**  ● ● ● ● ● ●●●●● ●●● ● ● ●● ● ●●●●● ●● ● ● ● ● ●

**3EX7_HUMAN**  262 **EWKFDTLCDLYDTLTITQAVIFCNTKRKVDWLTEKMREANFTVSSMHGDMPQKERESIMKEFRSGASRVLISTDVWARGLDVPQVSLIINYDLPNNRELYIHRIGRSGRYGRKGVAINFVKNDDIRILRDIEQ** 394*

**Q8MRB7_DROME**  265 **KYKDVYLVHILNELAGNSFMIFCSTCNNTVKTALMLRALGLAAIPLHGQMSQNKRLAALNKFKAKNRSILISTDVASRGLDIPHVDVVVNFDIPTHSKDYIHRVGRTARAGRSGKAITLVSQYDIELYQRIEH** 397

**IF4A_CAEEL**  233 **EWKFDCLCDLYNVVNVTQAVIFCNTRRKVDTLTEKMTENQFTVSCLHGDMDQAERDTIMREFRSGSSRVLITTDILARGIDVQQVSLVINYDLPSNRENYIHRIGRSGRFGRKGVAINFVTENDARQLKEIES** 365

**DBP3_YEAST**  322 **RGKERKLLELLKKYKNEKVLIFALYKKEAARVERNLKYNGYNVAAIHGDLSQQQRTQALNEFKSGKSNLLLATDVAARGLDIPNVKTVINLTFPLTVEDYVHRIGRTGRAGQTGTAHTLFTEQEKHLAGGLVN** 454

**IF4A3_ARATH**  245 **EWKLETLCDLYETLAITQSVIFVNTRRKVDWLTDKMRSRDHTVSATHGDMDQNTRDIIMREFRSGSSRVLITTDLLARGIDVQQVSLVINFDLPTQPENYLHRIGRSGRFGRKGVAINFMTSEDERMMADIQR** 377

**C4M5I5_ENTHI**  221 **KYRDGYLISILKETEGKTIIIFTMKCSGCTKLVMMLRQMGYAAIPLHGKMSQQKRLIALEKFKSGKRGILVATDVASRGLDIPNVDIVINYDCPLEPKDYVHRVGRTARAGKSGYAITLVTQYSIELYQRIET** 353

**foreground (32603):**  **DDKFAALVDFIEEE PGSAVVFCSSKEGCDEVADKLEAAGYSAAAIHGGKSQAARTKAIAAFKAGSVDVLVCTDVAARGIDVDGVSHVVNYDFPNSADSYVHRVGRTGRAGASGTAVSFVSEDDAKYAKAIEE**

**RERLEL LHLLRKL KRVLI VRTRHRVERLTRL RRL LPVVSL SDMP RK LRIMKR RK KIRI IA L S L IPDLDL IQFEP RDPKD L I A RK LSLTLLTPEERRLLRRL R**

**K D RI K EKTI TN R A I K KR IR NLD KQ E VLEK D E I ENIT L NIET I KE V IL I E V K K**

**wt_res_freqs (4382): 12711171111111 1112149211511131131171116113123771128119112111831611138519867799492115239275619111118499299869681191623441113111111541**

**111112 1155111 11234 2251113115111 111 112115 1321 11 121111 31 2123 26 1 1 5 532212 51211 13212 1 5 3 22 11133123141114113 1**

**1 1 11 1 1214 13 1 3 1 1 11 21 121 12 2 1511 1 1 1 1111 3 1151 2 11 1 41 1 1 1 1 2**

**insertions 2**

**deletions 3211111 552 2 11122233456**

**background (81641):**  **AGKVDAVVDIVEEEEGGQAIVFCNSKDTADEVADALKERGVKAGRYDGGTSAADREAAEDAFNAGDVDFVVGTSVAARGIDVTGVSTVVHYGAPKSLASYIQRIGRAGRDGDTGEAYRYTSEGDEEIYEAIEE**

**EERLE LLKLLRKLR RPILLYLPFIRMLELLEKL RKL LPILVLHSKMPPKEQQRLLRRLREDKLRILLS KILEM LTIPDIRLIILFEPDWNPEQLL LAD VH I KPKKVLLLLDKEE KMLRRLLK**

**A I IIE IK K ERV I VQRVKEI I E IRVA I ADLD E K KIIKD KN EIKVIIA NMIGV VNLQNADV ILDL R S D AR QKS CIIIIT D IKKAK**

**wt_res_freqs (15505): 1151121111112113111245112111211121161113111111142111115211111611411111316112118251112116211112131211192179449151131121111113211111112**

**11121 411231111 1115111111112313111 111 11111331121113111111111111112522 11221 3124111114111122221121 111 11 1 1111312311112 11211311**

**1 1 112 31 1 113 3 1111111 1 2 2121 1 2121 1 1 12111 11 2113123 11131 23311311 2131 1 1 1 21 211 121111 1 11111**

**position**  . 270 . 280 . 290 . 300 . 310 . 320 . 330 . 340 . 350 . 360 . 370 . 380 . 390
