# Supplementary material for: Inferring joint sequence-structural determinants of protein functional specificity
Source: eLife. 2018 Jan 16;7:e29880. doi: 10.7554/eLife.29880 (PMC5770160; doi:10.7554/eLife.29880)
Supplement: Figure 4—source data 1. [file elife-29880-fig4-data1.docx]

**Figure 4— Source data 1. EEP superfamily and exoIII_AP-endo family for APE1.**

**Chordata**  64 **ICSWNVDGLRAWIKKKGLDWVKEEAPDILCLQETKCSENKLPAELQELPGLSHQYWSApSDKE-GYSGVGLL.SRQ....CPLKVSYGIGDEEHDQEGRVIVAEFDSFVLVTAYVPNAGRGLVRLEYR.QR.WDEAFRKFLKGLASRKPLVLCGDLNVAHEEIDLRNPKGN.K** 227*

**Arthropoda**  429 **ICSWNVAGLRAWLKKDGLQLIDLEEPDIFCLQETKCANDQLPEEVTRLP-GYHPYWLC.MP-G-GYAGVAIY.SKI....MPIHVEYGIGNEEFDDVGRMITAEYEKFYLINVYVPNSGRKLVNLEPR.MR.WEKLFQAYVKKLDALKPVVICGDMNVSHMPIDLENPKNN.T** 589

**Brachiopoda**  50 **IVSWNINGIRAWIKKNGHEYVRKENPDIICFQEIKCAEDKLPPECS--IPDYYPYWLT.ADKE-GYAGTGIL.SKT....KPLSIKYGLGIEEHDNEGRAITAEYEKFYLVTSYVPNSGRGLVRLNYRtKE.WDEAFREYLLGLEEKKPVILCGDLNVAHKAIDLANPKSNyN** 212

**Mollusca**  100 **IASWNINGVRAWLDKEGLSYLNAEQPDVLCVQELKCDVSKIPAAAE--VDGYSTHWLS.GDTE-GYSGVGMY.FKK....KPIKITDGIGISKHDKEGRVITAEFEKFFLVNTYIPNSGRGLVRLKYRsEE.WDKDFRNYIKSLDAKKPVVWCGDLNVSHQEIDIKNAKGN.K** 261

**Annelida**  56 **FSSWNVNGIRAWVEKNGHSYVTAEDPDIFCVQETKCAKDLIPDDAN--IEGYHAYWLS.GDKD-GYSGTGLY.SKQ....EPLSVTYGIDKEEHDKEGRVITAEFDKFYFVTAYVPNAGRGLPRLSYR.SEkWDPDFREYLKNLDAKKPVVMCGDLNVAHKEIDIANPKSN.K** 217

**Cnidaria**  54 **IVSWNVNGVKAWLKCKPSTFVSREKPDLICLQEIKCDEDKFPSEAE--FKGYNVYLNS.ADQK-GYAGTGIL.SKT....EPLEVKYGIGKKEHDNEGRVITAEYKKFYLVNAYVPNSGRGLPRLDYR.QD.WDKDFTSYLKRLDKKKPVILCGDLNVAHKEIDLANPGXN.K** 214

**Echinodermata**  91 **ISAWNVGGMKAWIKKGGIDYLTKESPDIFFAQETKIDATKPPPEAD--LDDYHITYNA.AEKK-GYSGVALF.SKK....EPLSVTKGMGIEEHDKEGRLITAEYDSFYFVGVYVPNSSRKLVRLDYR.QE.WDKDFHAYLKKLDAKKPVICCGDMNVAHEEIDLKNPKSN.R** 251

**Priapulida**  65 **VASWNVNGLRAWIEKGGLDYLRKENPDVFFLQETKCSKDKLPAGGERCGRL-HGALAG.GRQGR------LLrCRDvhedCTLSIKHGIGMSEHDKEGRAITAEYDKFFVVGVYVPNSQKKLARLDYR.QK.WDKDFRDYLKKLENSKPVILCGDLNVCHEEIDLARPANN.H** 226

**position**  . 70 . 80 . 90 . 100 . 110 . 120 . 130 . 140 . 150 . 160 . 170 . 180 . 190 . 200 . 210 . 220 .

**_**

**_**

**_ _**

**_ _ _**

**_ _ _ _**

**_ _ _ _ _**

**_ _ _ __ _**

**_ _ _ __ _**

**_ _ _ __ _**

**_ _ _ __ _**

**_ _ _ __ _**

**_ _ _ __ _**

**_ _ _ __ _**

**_ _ _ __ _**

**_ _ _ __ _**

**_ _ _ __ _**

**_ _ _ __ _**

**_ _ _ __ _**

**_ _ _ __ _**

**_ _ _ __ _**

**_ _ _ __ _**

**_ _ _ __ _**

**_ _ _ __ _**

**_ _ _ __ _**

**_ _ _ __ _**

**_ _ _ __ _**

**_ __ _ __ _**

**_ _ __ _ __ _**

**_ _ __ _ __ _**

**_ _ __ _ __ _**

**_ _ __ _ __ _**

**_ _ __ _ ____**

**_ _ _ __ _ ____**

**_ ___ __ __ _ _ ____**

**_ ___ __ ___ _ _ _ ____**

**_ ___ __ ___ _ _ _ ____**

**_ ___ __ ___ _ _ _ ____**

**_ ___ ___ ___ _ _ _ _ ____**

**_ ___ ___ ___ _ _ _ _ ____**

**_ ___ ___ ___ _ _ _ _ ____**

**_ ____ ___ ___ _ _ _ _ ____**

**_ ____ ___ ___ _ _ _ _ ____**

**_ ____ ___ ___ _ _ _ _ ____**

**_ ____ ___ ___ _ _ _ __ ____**

**_ ____ ___ ___ _ _ _ _ _ __ ____**

**_ ____ ___ ___ _ _ _ _ _ __ ____**

**_ ____ ___ ___ _ _ _ _ _ __ ____**

**_ ____ ___ ___ _ _ _ _ _ __ ____**

**_ ____ ___ ___ _ _ _ _ _ __ ____**

**_ ____ ___ ___ __ _ _ _ _ __ ____**

**_ ____ ___ ___ __ _ _ _ _ __ ____**

**EEP** ● ●●●● ●●● ●●● ●● ● ● ● ● ●● ●●●●

**5DFF|A**  64 **ICSWNVDGLRAWIKKKGLDWVKEEAPDILCLQETKCSENKLPAELQELPGLSHQYWSApSDKE-GYSGVGLL.SRQ....CPLKVSYGIGDEEHDQEGRVIVAEFDSFVLVTAYVPNAGRGLVRLEYR.QR.WDEAFRKFLKGLASRKPLVLCGDLNVAHEEIDLRNPKGN.K** 227*

**RRP1_DROME**  429 **ICSWNVAGLRAWLKKDGLQLIDLEEPDIFCLQETKCANDQLPEEVTRLP-GYHPYWLC.MP-G-GYAGVAIY.SKI....MPIHVEYGIGNEEFDDVGRMITAEYEKFYLINVYVPNSGRKLVNLEPR.MR.WEKLFQAYVKKLDALKPVVICGDMNVSHMPIDLENPKNN.T** 589

**XP_013416767.1**  50 **IVSWNINGIRAWIKKNGHEYVRKENPDIICFQEIKCAEDKLPPECS--IPDYYPYWLT.ADKE-GYAGTGIL.SKT....KPLSIKYGLGIEEHDNEGRAITAEYEKFYLVTSYVPNSGRGLVRLNYRtKE.WDEAFREYLLGLEEKKPVILCGDLNVAHKAIDLANPKSNyN** 212

**XP_005101256.1**  100 **IASWNINGVRAWLDKEGLSYLNAEQPDVLCVQELKCDVSKIPAAAE--VDGYSTHWLS.GDTE-GYSGVGMY.FKK....KPIKITDGIGISKHDKEGRVITAEFEKFFLVNTYIPNSGRGLVRLKYRsEE.WDKDFRNYIKSLDAKKPVVWCGDLNVSHQEIDIKNAKGN.K** 261

**ELU05263.1**  56 **FSSWNVNGIRAWVEKNGHSYVTAEDPDIFCVQETKCAKDLIPDDAN--IEGYHAYWLS.GDKD-GYSGTGLY.SKQ....EPLSVTYGIDKEEHDKEGRVITAEFDKFYFVTAYVPNAGRGLPRLSYR.SEkWDPDFREYLKNLDAKKPVVMCGDLNVAHKEIDIANPKSN.K** 217

**KXJ27510.1**  54 **IVSWNVNGVKAWLKCKPSTFVSREKPDLICLQEIKCDEDKFPSEAE--FKGYNVYLNS.ADQK-GYAGTGIL.SKT....EPLEVKYGIGKKEHDNEGRVITAEYKKFYLVNAYVPNSGRGLPRLDYR.QD.WDKDFTSYLKRLDKKKPVILCGDLNVAHKEIDLANPGXN.K** 214

**XP_789515.3**  91 **ISAWNVGGMKAWIKKGGIDYLTKESPDIFFAQETKIDATKPPPEAD--LDDYHITYNA.AEKK-GYSGVALF.SKK....EPLSVTKGMGIEEHDKEGRLITAEYDSFYFVGVYVPNSSRKLVRLDYR.QE.WDKDFHAYLKKLDAKKPVICCGDMNVAHEEIDLKNPKSN.R** 251

**XP_014672642.1**  65 **VASWNVNGLRAWIEKGGLDYLRKENPDVFFLQETKCSKDKLPAGGERCGRL-HGALAG.GRQGR------LLrCRDvhedCTLSIKHGIGMSEHDKEGRAITAEYDKFFVVGVYVPNSQKKLARLDYR.QK.WDKDFRDYLKKLENSKPVILCGDLNVCHEEIDLARPANN.H** 226

**foreground (45579):**  **VASWNVNGFNAARADAVAAWVAAENADVVCVQETKDSDGAFLAALAAALGGY SYYSG AKGGGGGGGVGVF SKK SVVAV TGLGGDGADGSGGAVAATFEPFTFASAHFPAGGGGTEKLAAA LA QRAAFAARVAALA GDPVFVCGDFNAAHDSDAYENLEGN E**

**LMTF LLSLR LPRLLRLLRRLDP LLAL L DQAQLPLLPLL P L VFP R K YE LALL VRY PPLE V PSEE L ERRVLVLRLR LVLINLYL N SSDLPR EER R WLEELLELLKK L DE LLLM L IRP E DLKDPRSL L**

**IL Y IR I KK I E IKE IIG I E E L S I Y KL D L NK I VEI IRIV V S E D K E D I D I E IIIL V D I KAF R**

**wt_res_freqs (12726): 4233942311113111111111111274321791111111111111111123 11112 1111121163111 321 11111 111111112111221131211211111612121131211111 11 2111111111111 1133121995911112111111114 1**

**1141 11122 1112111311123 1216 1 11111121111 1 1 111 1 1 11 1423 131 2111 1 1111 1 1321211121 21113124 1 111222 113 1 11112211311 1 11 1131 2 113 1 21111111 1**

**31 2 21 1 11 2 1 211 322 1 1 1 1 1 3 1 11 1 1 11 1 211 1112 2 2 1 1 2 1 1 1 1 1 1 1411 1 1 1 311 1**

**insertions**

**deletions 2111111111131187666655111121111113546777613331135552222333 1113421122111 111 12222334431233314222222222211 1 13511277711555 54 422222333445175431 12223332344677 7**

**position**  . 70 . 80 . 90 . 100 . 110 . 120 . 130 . 140 . 150 . 160 . 170 . 180 . 190 . 200 . 210 . 220 .

**_**

**_**

**_ _**

**_ _**

**_ _**

**_ _ _**

**_ _ _**

**_ _ _**

**_ _ _**

**_ _ _**

**_ _ _ _ _**

**_ _ _ _ _**

**_ _ _ _ _**

**_ _ _ _ _ _**

**_ _ _ _ _ _**

**_ _ _ _ _ _**

**_ _ _ _ _ _**

**_ _ _ _ _ _**

**_ _ _ _ _ _ _**

**_ _ _ _ _ _ _**

**_ _ _ _ _ _ _**

**_ _ _ _ _ _ _**

**_ _ _ _ _ _ _**

**_ _ _ _ _ _ _ _ _**

**_ _ _ _ _ _ _ _ _**

**_ _ _ _ _ _ _ _ _ _**

**_ _ _ _ _ _ _ _ _ _**

**_ _ _ _ _ _ _ _ _ _**

**_ _ _ _ _ _ _ _ _ _ _**

**_ _ _ _ _ _ _ _ _ _ _**

**_ _ _ _ _ _ _ _ _ _ _ _**

**_ _ _ _ _ _ __ _ _ _ _ _ __ _**

**_ _ _ _ _ _ _ __ _ _ _ _ _ __ _**

**_ _ _ _ _ _ _ ___ _ _ _ _ _ __ _**

**___ _ _ _ _ _ _ ___ _ _ _ _ _ __ _**

**___ _ _ _ _ _ _ ___ _ _ _ _ _ __ _**

**___ _ _ _ _ _ _ _ ___ _ _ _ _ _ __ _**

**___ _ _ _ _ _ _ _ ___ _ _ _ _ _ __ _**

**____ _ _ _ _ _ _ _ ___ _ _ _ _ _ __ _**

**____ _ _ _ _ _ _ _ ___ _ _ _ _ _ __ _**

**____ _ _ _ _ _ _ _ ___ _ _ _ _ _ __ _**

**____ _ _ _ __ _ _ _ ___ _ _ _ _ _ __ _**

**____ _ _ _ __ _ _ _ ___ _ _ _ _ _ __ _**

**exoIII_AP-endo**  ●●●● ● ● ● ●● ● ● ● ●●● ● ● ● ● ● ●● ●

**5DFF|A**  64 **ICSWNVDGLRAWIKKKGLDWVKEEAPDILCLQETKCSENKLPAELQELPGLSHQYWSApSDKE-GYSGVGLL.SRQ....CPLKVSYGIGDEEHDQEGRVIVAEFDSFVLVTAYVPNAGRGLVRLEYR.QR.WDEAFRKFLKGLASRKPLVLCGDLNVAHEEIDLRNPKGN.K** 227*

**RRP1_DROME**  429 **ICSWNVAGLRAWLKKDGLQLIDLEEPDIFCLQETKCANDQLPEEVTRLP-GYHPYWLC.MP-G-GYAGVAIY.SKI....MPIHVEYGIGNEEFDDVGRMITAEYEKFYLINVYVPNSGRKLVNLEPR.MR.WEKLFQAYVKKLDALKPVVICGDMNVSHMPIDLENPKNN.T** 589

**XP_013416767.1**  50 **IVSWNINGIRAWIKKNGHEYVRKENPDIICFQEIKCAEDKLPPECS--IPDYYPYWLT.ADKE-GYAGTGIL.SKT....KPLSIKYGLGIEEHDNEGRAITAEYEKFYLVTSYVPNSGRGLVRLNYRtKE.WDEAFREYLLGLEEKKPVILCGDLNVAHKAIDLANPKSNyN** 212

**XP_005101256.1**  100 **IASWNINGVRAWLDKEGLSYLNAEQPDVLCVQELKCDVSKIPAAAE--VDGYSTHWLS.GDTE-GYSGVGMY.FKK....KPIKITDGIGISKHDKEGRVITAEFEKFFLVNTYIPNSGRGLVRLKYRsEE.WDKDFRNYIKSLDAKKPVVWCGDLNVSHQEIDIKNAKGN.K** 261

**ELU05263.1**  56 **FSSWNVNGIRAWVEKNGHSYVTAEDPDIFCVQETKCAKDLIPDDAN--IEGYHAYWLS.GDKD-GYSGTGLY.SKQ....EPLSVTYGIDKEEHDKEGRVITAEFDKFYFVTAYVPNAGRGLPRLSYR.SEkWDPDFREYLKNLDAKKPVVMCGDLNVAHKEIDIANPKSN.K** 217

**KXJ27510.1**  54 **IVSWNVNGVKAWLKCKPSTFVSREKPDLICLQEIKCDEDKFPSEAE--FKGYNVYLNS.ADQK-GYAGTGIL.SKT....EPLEVKYGIGKKEHDNEGRVITAEYKKFYLVNAYVPNSGRGLPRLDYR.QD.WDKDFTSYLKRLDKKKPVILCGDLNVAHKEIDLANPGXN.K** 214

**XP_789515.3**  91 **ISAWNVGGMKAWIKKGGIDYLTKESPDIFFAQETKIDATKPPPEAD--LDDYHITYNA.AEKK-GYSGVALF.SKK....EPLSVTKGMGIEEHDKEGRLITAEYDSFYFVGVYVPNSSRKLVRLDYR.QE.WDKDFHAYLKKLDAKKPVICCGDMNVAHEEIDLKNPKSN.R** 251

**XP_014672642.1**  65 **VASWNVNGLRAWIEKGGLDYLRKENPDVFFLQETKCSKDKLPAGGERCGRL-HGALAG.GRQGR------LLrCRDvhedCTLSIKHGIGMSEHDKEGRAITAEYDKFFVVGVYVPNSQKKLARLDYR.QK.WDKDFRDYLKKLENSKPVILCGDLNVCHEEIDLARPANN.H** 226

**foreground (13711):**  **VASWNVNGVRACAKKGFLAWFAAANADVVCIQETKCSDGDFDAAAFEIAAGWEAAWSG AEGKGGWSGTAVY SKK ELDAVETGFGGDDEDAEGRAVAATYGGFSVASVYTPAGGSGTEKQAYK LA WYAAFQARAAALDAGKEVAVCGDWNVAHTDIDVANWEGN E**

**LITF I SL SVVLPRL ELLKRLQP ILAL LRLPEEQLPLELL LPP YHVVFHS QKR E YN V LL TRV PPLEIRY LPIEPL EQA ILELELPKLVLINL L NSRPDLPRLPFR MR FMERMLEYLRK LKEPPLLLM L IIPKPL LYDPRSL L**

**IV I AR QV DFIEKQD I I VQ D I EDEI LL SY N S K RA IF K ID R I EF S VITVDVEDIYIV V SAQD E FE K LDELRDHIKE REDR IIIV F QE IW KAF R**

**wt_res_freqs (2355): 1355969617511133231511111395261994811311411112321181111214 3118191391811 541 211151162332117155911141113214132919172131241147 41 411231211115112213123991946422392141114 1**

**2241 2 33 1112113 11511114 3316 1111123251211 411 7321221 331 5 63 8 14 251 2511121 321111 121 12212111212232 2 511112242112 12 41211113411 211132131 2 414111 2124111 1**

**51 4 54 12 2111112 2 1 12 2 1 1111 12 13 1 1 2 11 51 1 12 1 1 22 1 151121111122 3 1111 1 21 1 3214111112 1112 1411 2 13 21 311 1**

**insertions**

**deletions 7765555444453333333222222111111111 11111 51119111 595 2253 9 54 5 1 1142**

**background (31868):**  **VGSWNVRGLG DAAAVAAAVAELNADVVCFQEVENSQAADIAALAAAYGGYASYYSG RTGGGGGGGVGFF SKK SVVS STVLDG GAAGSNRAVAATFETFCFASAYFPAGGG RVAAA LA DAAAQAARVAALADGAPVFVCGDFNATPGSDAYEFL**

**LMTF LLSGN RLKLLLRLLLR DP LLAL I DD L LLLP L LA L YV P S R I LLLL VRY PLLE LS T L PRGPLVIRLRPLVLIVVHLAPPSS LEER R QLDLLLELL E L DE LLLM L DR DEPP L**

**IL Y I NF ERI EEIR I IIGV N E L Y NAII Y DI F A DK I V IK IRIVNT DSK D E ER EI D I I D IIIL S**

**wt_res_freqs (10372): 5132931211 1111121111111273321794111111111111111112111111 1121111152111 321 1111 111111 1111111211212111111111112111 21111 11 111111111111111133121995921212111111**

**1141 11111 31112111211 22 1216 1 11 1 2211 1 11 1 11 1 1 1 1 1123 131 2111 11 1 1 132131122112111118511111 1114 1 211122113 1 1 11 1131 2 11 1111 1**

**22 3 3 11 112 1121 1 3221 1 1 1 1 1431 1 11 1 1 12 1 2 11 111232 121 1 1 11 12 1 1 1 1 1311 1**

**position**  . 70 . 80 . 90 . 100 . 110 . 120 . 130 . 140 . 150 . 160 . 170 . 180 . 190 . 200 . 210 . 220 .

**Chordata**  228 **.KNAGFTPQERQGFGELLQAvPLADSFRHLYPNTPYAYTFWTYMMNARSKNVGWRLDYFLLSHSLLPALCDSKIRSKALGSDHCPITLYL** 316*

**Arthropoda**  590 **.KNAGFTQEERDKMTELLGL.GFVDTFRHLYPDRKGAYTFWTYMANARARNVGWRLDYCLVSERFVPKVVEHEIRSQCLGSDHCPITIFF** 677

**Brachiopoda**  213 **.KTPGYTQAEIDGLSKLLDK.GFVDSFRHLYPDVTGAYSFWTYMGNARSKNVGWRLDYFLISKKLLPSLCDSLIRKEVMGSDHCPIALLL** 300

**Mollusca**  262 **.KNAGFTQEERDGFTEMLNE.GFIDSFRHLYPEEEGAYTFWTYFMNARAKNVGWRLDYFVLSERFKEQMCDSVIRSKVLGSDHCPIVLHL** 349

**Annelida**  218 **.KSAGFTPQERQGFSELLEA.GFVDAFRELYPEETKKYSYWTYMGNARGKNVGWRLDYFVVSEKIKDGICDSLIRSEVMGSDHCPVVLLM** 305

**Cnidaria**  215 **.RTAGFTIEERDGFTKLLSQ.GFVDTFRELYPDKKNAYSFWTYMRNARAKNVGWRLDYFVVSERFLGKVSDSVIKTRVMGSDHCPISLLL** 302

**Echinodermata**  252 **nKTPGFTDQEREGFTSLLDM.GFVDSFRHLYPEEADAYSFWTYMGNCRAKNVGWRLDYGVISKALVPKLCDNQMRLQTFGSDHCPMVVSL** 340

**Priapulida**  227 **.RNAGFSDEEREGFTQLLNA.GFIDTYRSLHPAQAGAYTFWTYMMNARSKDIGWRLDYFVISESLLPDLCDSVIRKHTMGSDHCPIALLL** 314

**position**  230 . 240 . 250 . 260 . 270 . 280 . 290 . 300 . 310 .

**_**

**_ _**

**_ _**

**_ _**

**_ _**

**_ _**

**_ _**

**_ _**

**_ __**

**_ __**

**_ __**

**_ __**

**_ __**

**_ ___**

**_ ___**

**_ ___**

**_ ___**

**_ ___**

**_ _ ___**

**_ _ ___**

**_ _ ___**

**_ _ ___**

**_ _ ___**

**_ _ ___**

**_ _ ___**

**_ _ ___**

**_ _ ___**

**_ _ ___**

**_ _ ___**

**_ _ ___**

**_ _ ___**

**_ _ ___ _**

**_ _ ___ _**

**_ _ ___ __**

**_ _ ___ __**

**_ _ ___ __**

**__ _ ___ __**

**__ _ _ ___ __ _**

**_ __ _ _ ___ __ _**

**_ __ __ _ ___ __ _**

**_ __ __ _ ___ __ _**

**EEP**  ● ●● ●● ● ●●● ●● ●

**5DFF|A**  228 **.KNAGFTPQERQGFGELLQAvPLADSFRHLYPNTPYAYTFWTYMMNARSKNVGWRLDYFLLSHSLLPALCDSKIRSKALGSDHCPITLYL** 316*

**RRP1_DROME**  590 **.KNAGFTQEERDKMTELLGL.GFVDTFRHLYPDRKGAYTFWTYMANARARNVGWRLDYCLVSERFVPKVVEHEIRSQCLGSDHCPITIFF** 677

**XP_013416767.1**  213 **.KTPGYTQAEIDGLSKLLDK.GFVDSFRHLYPDVTGAYSFWTYMGNARSKNVGWRLDYFLISKKLLPSLCDSLIRKEVMGSDHCPIALLL** 300

**XP_005101256.1**  262 **.KNAGFTQEERDGFTEMLNE.GFIDSFRHLYPEEEGAYTFWTYFMNARAKNVGWRLDYFVLSERFKEQMCDSVIRSKVLGSDHCPIVLHL** 349

**ELU05263.1**  218 **.KSAGFTPQERQGFSELLEA.GFVDAFRELYPEETKKYSYWTYMGNARGKNVGWRLDYFVVSEKIKDGICDSLIRSEVMGSDHCPVVLLM** 305

**KXJ27510.1**  215 **.RTAGFTIEERDGFTKLLSQ.GFVDTFRELYPDKKNAYSFWTYMRNARAKNVGWRLDYFVVSERFLGKVSDSVIKTRVMGSDHCPISLLL** 302

**XP_789515.3**  252 **nKTPGFTDQEREGFTSLLDM.GFVDSFRHLYPEEADAYSFWTYMGNCRAKNVGWRLDYGVISKALVPKLCDNQMRLQTFGSDHCPMVVSL** 340

**XP_014672642.1**  227 **.RNAGFSDEEREGFTQLLNA.GFIDTYRSLHPAQAGAYTFWTYMMNARSKDIGWRLDYFVISESLLPDLCDSVIRKHTMGSDHCPIALLL** 314

**foreground (45579):**   **GSAGFSDEERAAFRRLLDA GFTDAYRAANGDAGGGYSWKSDRGGGRAKGPGARIDRVFASGGLADKVVSAGVDDD RGSDHAPVVATF**

**KKPLVLPP ELL L L LVELF LYPEVEPPPTFPYY SRAFPRR PL L YILVRPD LLRLLDVRILPE P L LLLEL**

**NDVS T DW V H K FAF YWD AK WQ N S H YT S K YE L F I VDI**

**wt_res_freqs (12726): 1135511474111113211 42142121111312112221112121311112144911213112211211212111 118881641312**

**31111351 112 1 1 31111 11621111161111 1152111 11 2 2442111 111111112111 1 1 11113**

**1111 3 11 1 3 2 111 211 11 11 1 1 2 11 1 1 11 1 1 1 111**

**insertions**

**deletions 7777777774443222333 322335641777924432233337777735322211112253444254444444443232222211111**

**position**  230 . 240 . 250 . 260 . 270 . 280 . 290 . 300 . 310 .

**_**

**_**

**_**

**_**

**_**

**_**

**_**

**_**

**_**

**_**

**_**

**_**

**_**

**_ _**

**_ _**

**_ _ _**

**_ _ _**

**_ _ _**

**_ _ _**

**_ _ _**

**_ _ _**

**_ _ _**

**_ _ _**

**_ _ _**

**_ _ _**

**_ _ _**

**_ _ _**

**_ _ _**

**_ _ _**

**_ _ _ _**

**_ _ _ _ _**

**_ _ _ _ _**

**_ _ _ _ _**

**_ _ _ _ _ _**

**__ _ _ _ _ _ _**

**__ _ _ _ _ _ _**

**__ _ _ _ _ _ _**

**__ _ _ _ _ _ _**

**__ _ _ _ _ _ _ _**

**__ _ _ _ _ _ _ _**

**__ _ _ _ _ _ _ _**

**__ _ _ _ _ _ _ _ _**

**__ _ _ _ _ _ _ _ _**

**__ _ _ _ _ _ _ _ _ _**

**__ _ _ _ _ _ _ _ _ _ _**

**__ _ _ _ _ _ _ _ _ _ _**

**__ _ _ _ _ _ _ _ _ _ _**

**__ _ _ _ _ _ _ _ _ _ _**

**__ _ _ _ _ _ _ _ _ _ _**

**__ _ _ _ _ _ _ _ _ _ _**

**__ _ _ _ _ _ _ _ _ _ _**

**exoIII_AP-endo**  ●● ● ● ● ● ● ● ● ● ● ●

**5DFF|A**  228 **.KNAGFTPQERQGFGELLQAvPLADSFRHLYPNTPYAYTFWTYMMNARSKNVGWRLDYFLLSHSLLPALCDSKIRSKALGSDHCPITLYL** 316*

**RRP1_DROME**  590 **.KNAGFTQEERDKMTELLGL.GFVDTFRHLYPDRKGAYTFWTYMANARARNVGWRLDYCLVSERFVPKVVEHEIRSQCLGSDHCPITIFF** 677

**XP_013416767.1**  213 **.KTPGYTQAEIDGLSKLLDK.GFVDSFRHLYPDVTGAYSFWTYMGNARSKNVGWRLDYFLISKKLLPSLCDSLIRKEVMGSDHCPIALLL** 300

**XP_005101256.1**  262 **.KNAGFTQEERDGFTEMLNE.GFIDSFRHLYPEEEGAYTFWTYFMNARAKNVGWRLDYFVLSERFKEQMCDSVIRSKVLGSDHCPIVLHL** 349

**ELU05263.1**  218 **.KSAGFTPQERQGFSELLEA.GFVDAFRELYPEETKKYSYWTYMGNARGKNVGWRLDYFVVSEKIKDGICDSLIRSEVMGSDHCPVVLLM** 305

**KXJ27510.1**  215 **.RTAGFTIEERDGFTKLLSQ.GFVDTFRELYPDKKNAYSFWTYMRNARAKNVGWRLDYFVVSERFLGKVSDSVIKTRVMGSDHCPISLLL** 302

**XP_789515.3**  252 **nKTPGFTDQEREGFTSLLDM.GFVDSFRHLYPEEADAYSFWTYMGNCRAKNVGWRLDYGVISKALVPKLCDNQMRLQTFGSDHCPMVVSL** 340

**XP_014672642.1**  227 **.RNAGFSDEEREGFTQLLNA.GFIDTYRSLHPAQAGAYTFWTYMMNARSKDIGWRLDYFVISESLLPDLCDSVIRKHTMGSDHCPIALLL** 314

**foreground (13711):**   **GSAGFSDEERAAFTAFIGA GWTDAYRAFNGDAEGAYSWWSYRGGGRANNAGWRIDHQLASEGIAAKATACGVDKAAKGSDHCPVTATY** i

**KKPLVLPP EWMRRLLEL LI VV HLYPEVPKPFTF D SRAFPRDV M L YL LTPRLKPRLVDSEILREPRP V LILEL**

**NDVS T KLDKI DS FV TF H K D Y AK WQK R L I V D E IRSA DI F A IVIDI**

**wt_res_freqs (2355): 1135511478323111111 922921912113123174684761213116194979315261114111111123111229993961111**

**31111351 231125411 21 21 131621111251 3 11521221 1 1 41 12316113212116132123 1 11234**

**1111 3 13111 21 33 24 3 2 1 1 11 112 1 1 2 2 1 1 1114 21 1 4 11121**

**insertions**

**deletions 22222 138 21 111111111111111111111222222222222222333333333332**

**background (31868):**   **RDLL RLTFA GFRDAWIAA GFGYSWKAD GGPPGRIDRVFASGGLALKVVSAEVVDD RGSDHRAVVATF**

**EME L L LVELF EPPPTFPF KR SWL YILVRPD L RPL VRILP P LPLLVEL**

**Y A Y Y A H Y KS L Y L F I DI**

**wt_res_freqs (10372): 1111 12111 311321112 211111111 1111133822213111111211112111 117881141312**

**111 1 1 31111 11126111 11 113 2432111 1 111 11111 1 1521113**

**1 1 2 1 1 1 1 11 1 1 1 1 1 11**

**position**  230 . 240 . 250 . 260 . 270 . 280 . 290 . 300 . 310 .
