# Supplementary material for: Inferring joint sequence-structural determinants of protein functional specificity
Source: eLife. 2018 Jan 16;7:e29880. doi: 10.7554/eLife.29880 (PMC5770160; doi:10.7554/eLife.29880)
Supplement: Figure 5—source data 1. [file elife-29880-fig5-data1.docx]

**Figure 5— Source data 1. EEP superfamily, INPP5 family and INPP5b subfamily.**

**_**

**Chordata**  269 **AGTYNVNGQSP.KE.CLRLWLS..NGiQ.APDVYCVGFQELDLSKEAFFFHDTPKEEEWFKAVSEGLHPDAKYAK.VKLIRLVGIMLLLYVKQEHAAYISEVEAETVGTGIMGRMGNKGGVAIRFQFHNTSICVVNSHLAAHIEEYERRNQDYKDICSRMQFCQP...DPslP** 432*

**Arthropoda**  215 **TCTWNVNGQPP.NGiKLDQWLS..SDeT.PPDIYAIGFQELDLSKEAFLFHETPREEEWRQVVVNSLHPGGVYTQ.VALVRLVGMMLLVYALEPHIPFI-EYSTDTVGTGIMGKLGNKGGVAVSCRIHNTSICFVNAHLAAHCEEFERRNQDYADICARLSFAKF...VP..-** 375

**Echinodermata**  432 **VGTWNVNGKGA.TE.DLRNWLA..ADpK.PPDMYAIGFQELDLSKEAFLFNDSIREEEWHKRVIMCLHQDGVYIK.LKLIRLVGMMLLVFIQERHYPYIDEVIAGTVGTGIMGKMGNKGAVAVRFNFHNTSFCFINSHLAAHMEEYERRNQDYHDICARMKFERE...HH..Q** 593

**Cnidaria**  342 **TGTWNVNGQLA.IE.CLKPWID..CNtE.PPDVLAIGFQELDLSAEALVFNDSSREDLWIKAIENALPKRADYFK.LKHVRLVGMMLVVYVQTKHKMQVLNLEAETNATGIMGMMGNKGGVAIRFQLHNSTICFVNSHLAAHQTEYERRNQDYREVYNKIKFTQF...QP..-** 502

**Hemichordata**  250 **IGTWNVNGKSC.AE.DLGPWLA..CDaE.PPDIYAVGFQELDLSKEAFLFNDSPREEEWLRGVYKALHPRANYKK.IKLIRLVGMMLIVFAKHELASYITDVAAETVGTGIMGKMGNKGGVGVRLVFHNTTFVFINSHLAAHVEEYERRNQDYNDICSRMLFKDF...GS..-** 410

**Brachiopoda**  276 **CGTWNTNGQPPgDT.DLSPWLA..FD.QyPPDIYAIGFQELDLSNQAFIFAESQREQEWFAAVRQGLHPQAAYKK.LCLIRLVGMMLILFVKKELTHHIKKVKAEQVGTGLLGMMGNKGGVAVRFELFDTSFCIINSHLAAHMEEYERRNQDYRDINSRLQFRDLlgyDN..-** 440

**Priapulida**  256 **TGTWNVNGQPA.SV.ALQAWLA..GDdE.PPDMYAVGFQELDLSKEAFLFNDSPREDEWLQAVTNALHRGATYTL.VRTIRLVGMMLVTYVAEPLRERVIEVAAETVGTGIMGKMGNKGGVAVRFLLHNTSLCVVNAHLAAHTDDCERRNQDHADICERLRFARF...DP..-** 416

**Mollusca**  168 **CGTWNVNGQSP.TV.SIHTWLA..SDpE.PPDIYAVGFQELDLSKEAFIFSESPKEGEWLQIVTSCLHPKAKYKK.VKLIRLVGVMLIVFVTKELSPFVKYIDADYVATGIMGLLGNKGGVAVRMTIHNTSLCFINSHLAAHQEEFERRNQDYRDIVSRLRFRQF...VP..-** 328

**Annelida**  30 **CGTWNVNGQQA.SE.AINEWLCpaEDsE.PPDIYAIGFQELDLSKNAYIFSESVREEDWYKRIKEALHPKAKYKKvVKLVRLVGMMLVVFIRKGLELEIYDVMSGTVGTGIMGVMGNKGGVAVRFGLRDSTLCFVNSHLAAHTVEFERPHTDFKDINSRMLFGNR...--..-** 191

**position**  . 280 . 290 . 300 . 310 . 320 . 330 . 340 . 350 . 360 . 370 . 380 . 390 . 400 . 410 . 420 . 430

**_**

**_**

**_ _ _**

**_ _ _**

**_ _ _**

**_ _ _**

**_ _ _**

**_ _ _**

**_ _ _**

**_ _ _**

**_ _ _**

**_ _ _**

**_ _ _**

**_ _ _**

**_ _ _**

**_ _ _**

**_ _ _**

**_ _ _**

**_ _ _**

**_ _ _**

**_ __ _**

**_ _ _ __ _**

**_ _ _ __ _**

**_ _ _ __ _**

**_ _ _ __ _**

**_ _ _ __ _**

**_ ___ __ __ _**

**_ ___ __ __ _**

**_ ___ __ ___ _ _**

**_ ___ __ ___ _ _**

**_ ___ ___ ___ _ _**

**_ ___ ___ ___ _ _ _**

**_ ___ ___ ___ _ _ _**

**_ ____ ___ ___ _ _ _**

**_ ____ ___ ___ _ _ _**

**_ ____ ___ ___ _ _ _**

**_ ____ ___ ___ _ _ _ _**

**_ ____ ___ ___ _ _ _ _ _**

**_ ____ ___ ___ _ _ _ _ _**

**_ ____ ___ ___ _ _ _ _ _**

**_ ____ ___ ___ __ _ _ _ _**

**_ ____ ___ ___ __ _ _ _ _**

**______ ___ ___ __ _ _ _ _**

**______ _ ___ ___ __ _ _ _ _**

**______ _ ___ ___ _ __ _ _ _ _ _**

**______ _ ___ ___ _ __ _ _ _ _ _ _**

**______ _ ___ ___ _ __ _ _ _ _ _ _**

**______ _ ___ ___ _ __ _ _ _ _ _ _ _ _**

**______ _ ___ ___ _ __ _ _ _ _ _ _ _ _**

**EEP** ●●●●●● ● ●●● ●●● ● ●● ● ● ● ● ● ● ● ●

**4cmlA_human**  269 **AGTYNVNGQSP.KE.CLRLWLS..NGiQ.APDVYCVGFQELDLSKEAFFFHDTPKEEEWFKAVSEGLHPDAKYAK.VKLIRLVGIMLLLYVKQEHAAYISEVEAETVGTGIMGRMGNKGGVAIRFQFHNTSICVVNSHLAAHIEEYERRNQDYKDICSRMQFCQP...DPslP** 432*

**XP_011146720.1**  215 **TCTWNVNGQPP.NGiKLDQWLS..SDeT.PPDIYAIGFQELDLSKEAFLFHETPREEEWRQVVVNSLHPGGVYTQ.VALVRLVGMMLLVYALEPHIPFI-EYSTDTVGTGIMGKLGNKGGVAVSCRIHNTSICFVNAHLAAHCEEFERRNQDYADICARLSFAKF...VP..-** 375

**XP_011671046.1**  432 **VGTWNVNGKGA.TE.DLRNWLA..ADpK.PPDMYAIGFQELDLSKEAFLFNDSIREEEWHKRVIMCLHQDGVYIK.LKLIRLVGMMLLVFIQERHYPYIDEVIAGTVGTGIMGKMGNKGAVAVRFNFHNTSFCFINSHLAAHMEEYERRNQDYHDICARMKFERE...HH..Q** 593

**XP_012563558.1**  342 **TGTWNVNGQLA.IE.CLKPWID..CNtE.PPDVLAIGFQELDLSAEALVFNDSSREDLWIKAIENALPKRADYFK.LKHVRLVGMMLVVYVQTKHKMQVLNLEAETNATGIMGMMGNKGGVAIRFQLHNSTICFVNSHLAAHQTEYERRNQDYREVYNKIKFTQF...QP..-** 502

**XP_006812142.1**  250 **IGTWNVNGKSC.AE.DLGPWLA..CDaE.PPDIYAVGFQELDLSKEAFLFNDSPREEEWLRGVYKALHPRANYKK.IKLIRLVGMMLIVFAKHELASYITDVAAETVGTGIMGKMGNKGGVGVRLVFHNTTFVFINSHLAAHVEEYERRNQDYNDICSRMLFKDF...GS..-** 410

**XP_013382922.1**  276 **CGTWNTNGQPPgDT.DLSPWLA..FD.QyPPDIYAIGFQELDLSNQAFIFAESQREQEWFAAVRQGLHPQAAYKK.LCLIRLVGMMLILFVKKELTHHIKKVKAEQVGTGLLGMMGNKGGVAVRFELFDTSFCIINSHLAAHMEEYERRNQDYRDINSRLQFRDLlgyDN..-** 440

**XP_014679357.1**  256 **TGTWNVNGQPA.SV.ALQAWLA..GDdE.PPDMYAVGFQELDLSKEAFLFNDSPREDEWLQAVTNALHRGATYTL.VRTIRLVGMMLVTYVAEPLRERVIEVAAETVGTGIMGKMGNKGGVAVRFLLHNTSLCVVNAHLAAHTDDCERRNQDHADICERLRFARF...DP..-** 416

**XP_014778567.1**  168 **CGTWNVNGQSP.TV.SIHTWLA..SDpE.PPDIYAVGFQELDLSKEAFIFSESPKEGEWLQIVTSCLHPKAKYKK.VKLIRLVGVMLIVFVTKELSPFVKYIDADYVATGIMGLLGNKGGVAVRMTIHNTSLCFINSHLAAHQEEFERRNQDYRDIVSRLRFRQF...VP..-** 328

**ELT95411.1**  30 **CGTWNVNGQQA.SE.AINEWLCpaEDsE.PPDIYAIGFQELDLSKNAYIFSESVREEDWYKRIKEALHPKAKYKKvVKLVRLVGMMLVVFIRKGLELEIYDVMSGTVGTGIMGVMGNKGGVAVRFGLRDSTLCFVNSHLAAHTVEFERPHTDFKDINSRMLFGNR...--..-** 191

**foreground (45254):**  **VASWNVNGFNA A DADGVAA EV A AENADVVCVQETKDSDGAFLAGASESDVEWRQAVSNGLALAAALGG YHSYYSGGVGVFSKKKKSHAPRVFIVESA VGLGGDGADGSGGAVAATFEVGGTTFTFASAHFPAGGGKLAAALAQAAAFAARVAALAA DQ Q**

**LMTF LLSLR RLKALLR LL R RLDP LLAL L DQAQLPLDLVPELPW LKV ERS L LLE P L VFP LALLVRYPLKLL FL LPLEV PSEE L ERRVLVLRLRLP RPLVLINLYL N SSR EYR RFLELLLELLKK LK VP P**

**IL Y IR I KE I E WI K E IIG I NT KTK K F TKA E L I Y RE I HI L D L NK I VEIKIK K IRIV V S DEK EWRDEI D I E**

**wt_res_freqs (10787): 42339423111 1 1111111 11 1 1112743217911111111111111111191178211911111112 31111126311132111121212111111 11111111211122113121113112111116131211111111121111111111211 41 1**

**1141 11122 3111221 13 1 1123 1216 1 111111211111111 231 214 1 111 1 1 111 142413121131 11 11111 1111 1 132121112111 1121213124 1 112 113 111113211311 11 15 7**

**32 2 21 1 11 2 1 12 1 1 322 1 11 111 1 2 122 1 1 3 1 11 1 14 1 1 1 11 2 211111 1 1112 2 2 112 111111 1 1 1**

**insertions**

**deletions 21111111191 42 8776555 55 4 1111211111135467776139999999999999999963889665 222233322111111149999995312222233312333143222222223333211 1 135112115555442222233345526 99 9**

**position**  . 280 . 290 . 300 . 310 . 320 . 330 . 340 . 350 . 360 . 370 . 380 . 390 . 400 . 410 . 420 . 430

**_**

**_**

**_**

**_**

**_**

**_**

**_**

**_**

**_**

**_**

**_**

**_ _**

**_ _ _**

**_ _ _ _**

**_ _ _ _**

**_ _ _ _**

**_ _ _ _**

**_ _ _ _**

**_ _ _ _**

**_ _ _ _**

**_ _ _ _**

**_ _ _ _**

**_ _ _ _ _**

**_ _ _ _ _**

**_ _ __ _ _**

**_ _ ___ _ _**

**_ _ ___ _ _**

**_ _ _ ___ _ _**

**_ _ _ ____ _ _**

**_ _ _ _ ____ _ _**

**_ _ _ _ _ ____ _ _**

**_ _ _ _ _ ____ _ _**

**_ _ _ _ _ ____ _ _**

**_ _ _ __ _ ____ _ _**

**_ _ _ __ _ ____ _ _ _**

**_ _ _ __ _ ____ _ _ _**

**_ _ _ __ _ ____ _ _ _ _**

**_ _ _ _ ___ _ ____ _ _ _ _ _**

**__ _ _ _ ___ _ ____ _ _ _ _ _ _ _**

**__ _ _ _ ___ _ ____ _ _ _ _ _ _ _**

**__ _ _ _ ___ _ _____ _ _ _ _ _ _ _**

**__ _ _ _ ___ _ _____ _ _ _ _ _ _ _**

**__ _ _ _ ___ _ _____ _ _ _ _ _ _ _**

**__ _ _ _ ___ _ _____ _ _ _ _ _ _ _**

**__ _ _ _ ___ _ _____ _ _ _ _ _ _ _**

**__ _ _ _ ___ _ _____ _ _ _ _ _ _ _**

**__ _ _ _ ___ _ _ _____ _ _ _ _ _ _ _**

**__ _ _ _ ___ _ _ _____ _ _ _ _ _ _ _**

**INPP5 family**  ●● ● ● ● ●●● ● ● ●●●●● ● ● ● ● ● ● ●

**4cmlA_human**  269 **AGTYNVNGQSP.KE.CLRLWLS..NGiQ.APDVYCVGFQELDLSKEAFFFHDTPKEEEWFKAVSEGLHPDAKYAK.VKLIRLVGIMLLLYVKQEHAAYISEVEAETVGTGIMGRMGNKGGVAIRFQFHNTSICVVNSHLAAHIEEYERRNQDYKDICSRMQFCQP...DPslP** 432*

**XP_011146720.1**  215 **TCTWNVNGQPP.NGiKLDQWLS..SDeT.PPDIYAIGFQELDLSKEAFLFHETPREEEWRQVVVNSLHPGGVYTQ.VALVRLVGMMLLVYALEPHIPFI-EYSTDTVGTGIMGKLGNKGGVAVSCRIHNTSICFVNAHLAAHCEEFERRNQDYADICARLSFAKF...VP..-** 375

**XP_011671046.1**  432 **VGTWNVNGKGA.TE.DLRNWLA..ADpK.PPDMYAIGFQELDLSKEAFLFNDSIREEEWHKRVIMCLHQDGVYIK.LKLIRLVGMMLLVFIQERHYPYIDEVIAGTVGTGIMGKMGNKGAVAVRFNFHNTSFCFINSHLAAHMEEYERRNQDYHDICARMKFERE...HH..Q** 593

**XP_012563558.1**  342 **TGTWNVNGQLA.IE.CLKPWID..CNtE.PPDVLAIGFQELDLSAEALVFNDSSREDLWIKAIENALPKRADYFK.LKHVRLVGMMLVVYVQTKHKMQVLNLEAETNATGIMGMMGNKGGVAIRFQLHNSTICFVNSHLAAHQTEYERRNQDYREVYNKIKFTQF...QP..-** 502

**XP_006812142.1**  250 **IGTWNVNGKSC.AE.DLGPWLA..CDaE.PPDIYAVGFQELDLSKEAFLFNDSPREEEWLRGVYKALHPRANYKK.IKLIRLVGMMLIVFAKHELASYITDVAAETVGTGIMGKMGNKGGVGVRLVFHNTTFVFINSHLAAHVEEYERRNQDYNDICSRMLFKDF...GS..-** 410

**XP_013382922.1**  276 **CGTWNTNGQPPgDT.DLSPWLA..FD.QyPPDIYAIGFQELDLSNQAFIFAESQREQEWFAAVRQGLHPQAAYKK.LCLIRLVGMMLILFVKKELTHHIKKVKAEQVGTGLLGMMGNKGGVAVRFELFDTSFCIINSHLAAHMEEYERRNQDYRDINSRLQFRDLlgyDN..-** 440

**XP_014679357.1**  256 **TGTWNVNGQPA.SV.ALQAWLA..GDdE.PPDMYAVGFQELDLSKEAFLFNDSPREDEWLQAVTNALHRGATYTL.VRTIRLVGMMLVTYVAEPLRERVIEVAAETVGTGIMGKMGNKGGVAVRFLLHNTSLCVVNAHLAAHTDDCERRNQDHADICERLRFARF...DP..-** 416

**XP_014778567.1**  168 **CGTWNVNGQSP.TV.SIHTWLA..SDpE.PPDIYAVGFQELDLSKEAFIFSESPKEGEWLQIVTSCLHPKAKYKK.VKLIRLVGVMLIVFVTKELSPFVKYIDADYVATGIMGLLGNKGGVAVRMTIHNTSLCFINSHLAAHQEEFERRNQDYRDIVSRLRFRQF...VP..-** 328

**ELT95411.1**  30 **CGTWNVNGQQA.SE.AINEWLCpaEDsE.PPDIYAIGFQELDLSKNAYIFSESVREEDWYKRIKEALHPKAKYKKvVKLVRLVGMMLVVFIRKGLELEIYDVMSGTVGTGIMGVMGNKGGVAVRFGLRDSTLCFVNSHLAAHTVEFERPHTDFKDINSRMLFGNR...--..-** 191

**foreground (3601):**  **VGSWNVGGAGS DD SIASWFG SS D APDVYAFGFQEVDSGAAGNVLGASKSDPSWRQAVSNGLASGEGYEK VASESLGGVAICVWAKKSHAPRVSNVAAASTGCGFGGYWGNKGGVGVSFSYGGSSFCFVCCHFAAGDSAVAERNANYATIVSGTSFSDG DQ Q**

**IVTF MNARPP PQ DLTPLLL PE E P LLVL L LVPLEEKLLMDLDPEL W LKV ERS LLPPKFVL LRTVRMV MLLLLFIRPELL FLRHLQVDTVRT MMNMM AISIRMVLFETTLL INS LTSHQEKLER QDVKE LRRMVLPPP VP P**

**Y A KS E N SE F LD D II I I LSN EFI NT FTK E F TKA T D D I KQ Q LF SIYV RD V HIKEIETS K L L A LRIYD IT TA E E DQ E F D KKLR RN**

**wt_res_freqs (905): 55169534111 11 1112511 11 1 1481421849921111111111111111191178211911221712 32411517112151231121212224112212191371188993723241113143586139166411131296113118111117111 41 1**

**2181 131114 21 4611261 21 1 2 2152 3 2314111121111111 1 231 214 12111133 3111224 125215131131 112111112515 12111 51136113115121 253 8123222123 17111 322111211 15 7**

**1 1 11 2 1 11 1 11 1 51 3 2 111 112 11 111 1 2 122 1 1 1 1 13 1 31 1214 12 1 14112222 3 3 1 3 21112 11 12 1 1 11 1 2 1 1141 11**

**insertions 6 4**

**deletions 22111111111 11 1111111 11 1 11999999888887655432133551111999999999 1 1111111222111111111231111111112223221222211111111 1 1111111112222111 11111 1 1 3 99 9**

**background (41653):**  **VASWNVNGFNA AA RADGVAA EV A AENADVVCVQETKDSDGADLAGHVDDFFK AEAAAAGG YHAYYSGGVGFFSKKSKKNVTSTFIVLSVE GLGGDTADSSGGAVAATFEVGGKTFTVASAHFPAGGGKLAARLAQAAAIAARVAALAA**

**LMTF LLNLR LKLLLR LL R RLDP LLAL IR DQDQLPLR KK IW LLLLELP YVFPY LALLYRYPL DIPPMVLP E PSEE L ERRPLVLRLRLP RPLVLINLYL N RSR EYA RFLELLLELLKK LK**

**IL Y IRSI KERI E WI K E IIG V N ADF Q EE VR E L I R VTI L D L VI VEIKIK IRIV V S S DEK EWRDEQ DYI E I**

**wt_res_freqs (9882): 42439422111 11 3111111 11 1 11128433179121111111115621713 11111113 311111263111331114331132111111 1111111211111113121114111141116131211111131121111111111211**

**1141 11122 111211 13 1 1123 1226 11 11111121 11 11 1111111 11111 151413131 13121111 1 1111 1 132131112111 1121213134 1 113 111 111113111311 11**

**32 2 3111 1112 1 12 1 1 321 3 1 111 1 31 52 1 1 3 1 321 1 1 1 12 211111 1112 2 2 1 112 111111 111 1 1**

**position**  . 280 . 290 . 300 . 310 . 320 . 330 . 340 . 350 . 360 . 370 . 380 . 390 . 400 . 410 . 420 . 430

**_**

**_**

**_**

**_**

**_**

**_**

**_**

**_**

**_**

**_**

**_**

**_**

**_**

**_**

**_**

**_**

**_**

**_**

**_**

**_**

**_**

**_**

**_**

**_ _**

**_ _**

**_ _**

**_ _**

**_ _**

**_ _ _**

**_ _ _**

**_ _ _**

**_ _ _**

**_ _ _**

**_ _ _ _**

**_ _ _ _**

**_ _ _ _ _**

**_ _ _ _ _**

**_ _ _ _ _**

**_ _ _ _ _**

**_ _ _ _ _ _**

**__ _ _ _ _ _ _**

**__ _ _ _ _ _ _**

**__ _ _ _ _ _ _ _**

**__ _ _ _ _ _ _ _ _**

**__ _ _ _ __ _ __ _ _ _**

**_ ___ _ _ _ _ __ _ _ __ _ _ _ _ _ _**

**_ ___ ___ _ _ __ _ _ __ _ _ _ __ __ _ _ _**

**_ ____ ___ _ _ __ __ _ __ _ _ _ _ _ __ __ _ _ _**

**_ ____ _____ _ __ __ __ ___ _ ___ _ _ _ __ __ ___ _ _ ___**

**_ _ ____ _____ _ __ __ __ ___ _ ___ _ _ _ __ __ ___ _ _ ___ _**

**_ _ ____ _____ _ __ __ __ ___ _ ___ _ _ _ __ __ ___ _ _ ___ _**

**INPP5b subfamily**  ● ● ●●●● ●●●●● ● ●● ●● ●● ●●● ● ●●● ● ● ● ●● ●● ●●● ● ● ●●● ●

**4cmlA_human**  269 **AGTYNVNGQSP.KE.CLRLWLS..NGiQ.APDVYCVGFQELDLSKEAFFFHDTPKEEEWFKAVSEGLHPDAKYAK.VKLIRLVGIMLLLYVKQEHAAYISEVEAETVGTGIMGRMGNKGGVAIRFQFHNTSICVVNSHLAAHIEEYERRNQDYKDICSRMQFCQP...DPslP** 432*

**XP_011146720.1**  215 **TCTWNVNGQPP.NGiKLDQWLS..SDeT.PPDIYAIGFQELDLSKEAFLFHETPREEEWRQVVVNSLHPGGVYTQ.VALVRLVGMMLLVYALEPHIPFI-EYSTDTVGTGIMGKLGNKGGVAVSCRIHNTSICFVNAHLAAHCEEFERRNQDYADICARLSFAKF...VP..-** 375

**XP_011671046.1**  432 **VGTWNVNGKGA.TE.DLRNWLA..ADpK.PPDMYAIGFQELDLSKEAFLFNDSIREEEWHKRVIMCLHQDGVYIK.LKLIRLVGMMLLVFIQERHYPYIDEVIAGTVGTGIMGKMGNKGAVAVRFNFHNTSFCFINSHLAAHMEEYERRNQDYHDICARMKFERE...HH..Q** 593

**XP_012563558.1**  342 **TGTWNVNGQLA.IE.CLKPWID..CNtE.PPDVLAIGFQELDLSAEALVFNDSSREDLWIKAIENALPKRADYFK.LKHVRLVGMMLVVYVQTKHKMQVLNLEAETNATGIMGMMGNKGGVAIRFQLHNSTICFVNSHLAAHQTEYERRNQDYREVYNKIKFTQF...QP..-** 502

**XP_006812142.1**  250 **IGTWNVNGKSC.AE.DLGPWLA..CDaE.PPDIYAVGFQELDLSKEAFLFNDSPREEEWLRGVYKALHPRANYKK.IKLIRLVGMMLIVFAKHELASYITDVAAETVGTGIMGKMGNKGGVGVRLVFHNTTFVFINSHLAAHVEEYERRNQDYNDICSRMLFKDF...GS..-** 410

**XP_013382922.1**  276 **CGTWNTNGQPPgDT.DLSPWLA..FD.QyPPDIYAIGFQELDLSNQAFIFAESQREQEWFAAVRQGLHPQAAYKK.LCLIRLVGMMLILFVKKELTHHIKKVKAEQVGTGLLGMMGNKGGVAVRFELFDTSFCIINSHLAAHMEEYERRNQDYRDINSRLQFRDLlgyDN..-** 440

**XP_014679357.1**  256 **TGTWNVNGQPA.SV.ALQAWLA..GDdE.PPDMYAVGFQELDLSKEAFLFNDSPREDEWLQAVTNALHRGATYTL.VRTIRLVGMMLVTYVAEPLRERVIEVAAETVGTGIMGKMGNKGGVAVRFLLHNTSLCVVNAHLAAHTDDCERRNQDHADICERLRFARF...DP..-** 416

**XP_014778567.1**  168 **CGTWNVNGQSP.TV.SIHTWLA..SDpE.PPDIYAVGFQELDLSKEAFIFSESPKEGEWLQIVTSCLHPKAKYKK.VKLIRLVGVMLIVFVTKELSPFVKYIDADYVATGIMGLLGNKGGVAVRMTIHNTSLCFINSHLAAHQEEFERRNQDYRDIVSRLRFRQF...VP..-** 328

**ELT95411.1**  30 **CGTWNVNGQQA.SE.AINEWLCpaEDsE.PPDIYAIGFQELDLSKNAYIFSESVREEDWYKRIKEALHPKAKYKKvVKLVRLVGMMLVVFIRKGLELEIYDVMSGTVGTGIMGVMGNKGGVAVRFGLRDSTLCFVNSHLAAHTVEFERPHTDFKDINSRMLFGNR...--..-** 191

**foreground (252):**  **TGTWNVNGQSP NG CLEPWLS CG D APDFYCVGFQELDLSTEAFFYNDSSKEEEWRQAVSNGLHPGGKYAQ VALVRLVGIMLVVYAKADQAANVSNVAAESVGTGIMGKMGNKGGVAVSCQFHNSSFCFVNAHLAAHCEDYERRNQDYADICARLSFCTY DQ P**

**I Y P KE S R V HD Q P I AI K LFFETPR Q LKV ERS SKA KK RMI M LLFVRKELLPFIREIMTDT R IRFVL TTI I S V EF FR S MQ VVP VP Q**

**V DS G Q N S E V H F TKA D K II Q H SY KD E RI V V T K HQL**

**wt_res_freqs (53): 19959999979 11 1937994 51 1 1991964999999993999733731694791178211997117931 81669999499135351213211417248199998995999999995111799163929919999919349999997199949239211 41 7**

**1 3 1 34 4 2 1 17 1 8 4 34 6 2611552 3 231 214 138 36 113 5 6345243111172511317 3 48831 823 4 8 4 64 11 4 73 113 15 1**

**5 33 1 1 1 1 4 3 2 2 122 3 4 12 1 5 12 12 3 11 1 3 2 7 141**

**insertions 1 8 7 1 6**

**deletions 64444444444 44 4333333 33 3 33333333 111111111111111111 1 1**

**background (3349):**  **VGSWNVGGGGS DD SIASWFG SS S AADIYAFGFQELVSSAAAFVTGASKSDKSWQQAVDNALASGEGYEK VASVRLGGVAICVWAKKSHAPRVSNVSAASTGCGFMGAMGNKGGVGVSFSYHGSSFCFVCCHFAAGDSAVEERNQNYATIVSRTSFGDG SE**

**IVTF MNARPP PQ DLTELLL PE E PP LLVL L V PLE KLLMDLDLELPW RVIVKS LLPPRFIL LRTESMV LLLLLFIRPELL FLRHLQVDTVRT MGNMW AISLRMVLFETTLL INS LTSHQKKLAR ADV E LRGLVLPPN VP**

**Y A KS E N SD F LD D D I I I EGN ENILST FTK T D K V IS KQ Q IF SIYV RD V HIKEIETS K L YL AI LRIYD IT TA EEQ DQ E F KKQR SRS**

**wt_res_freqs (852): 54169534111 11 1112511 11 1 1175421839913111111111111111144144414511221711 32411517112151231121212224112212191171188993633241113143585139166511132295113118111116111 44**

**2181 131114 31 4611261 21 1 24 2152 4 2 241 112111111111 411141 22111214 3111134 425215131132 112111111515 13111 51116113115121 242 8123212113 271 1 321411211 11**

**1 2 11 1 1 12 1 11 1 1 1 3 2 111 112111 111 1 1 1 3 11 13 1 21 1214 12 1 14112222 3 3 21 23 21112 11 12 121 21 1 2 1111 111**

**position**  . 280 . 290 . 300 . 310 . 320 . 330 . 340 . 350 . 360 . 370 . 380 . 390 . 400 . 410 . 420 . 430

**Figure 5— Source data 1. INPP5b subfamily (continued)**

**Chordata**  433 **PLTISNHDVI...................LWLGDLNYRIEELDVEKVKKLIEEKDFQMLYAYDQLKIQVAAKTVFEGFTEGELTFQPTYKYDTGSDDWDTSEKCRAPAWCDRILWKGKNITQLSYQSHMALKTSDHKPVSSVF** 556*

**Arthropoda**  376 **PKSFKDHDQI...................YWLGDLNYRITEMDVNVAKHHIAEGNYSPVLTYDQLGQQRKAGRVFHGFHEAEIDFKPTYKYDPGTDNWDSSEKCRAPAWCDRVLWKGDAIKSISYKSYMELKISDHKPVSASF** 499

**Echinodermata**  594 **PLGVMQHDVV...................IWMGDLNYRINDLAVDIVKALIDNNHFKELLLQDQLNRQRELSRVFKGFDEAPIGFLPTYKYNSGSDDWDSSEKQRVPAWCDRILHRGACIVPKVYRSHMKLRLSDHKPVSALY** 717

**Cnidaria**  503 **HLSISDHDVI...................IWMGDLNYRFEELDPDQVKKLSDDMDYDKLYLNDQLNWQRSLGKVFEGFSEGQINFKPTYKYDPGTDNWDTSEKFRAPAWCDRILWKGKNIQQITYRSHVELRLSDHKPVSSLF** 626

**Hemichordata**  411 **PLHISQHDAI...................FWLGDLNYRISELSADEVKGFIRKDMLRDVYEYDQLNRQMRIQKAFIGFTEGEINFLPTYKYDSGTDSWDSSEKNRAPAWCDRILYRGESIRQLKYRGHMALKISDHKPVSSIF** 534

**Brachiopoda**  441 **PQSITDHERI...................FWLGDLNYRISGLDLERVKKECDAGNYSVLYEYDQLIQQRSLKNVFLDYEEAPIKFKPTYKYDTGTDSWDTSEKCRCPAWCDRVMWKGEHIENEAYRSHPILKTSDHKPVSAYF** 564

**Priapulida**  417 **PLPIWQHDVV...................LWIGDLNYRLDQLAYDQVMLLMEKGAFAKLLEYDQLYTQKKKGRAFKTYEEGPITFRPTYKFDPGSDTYDTSEKCRVPAWCDRVLWKGQHVKQLTYRSHPTLMISDHKPVSALF** 540

**Mollusca**  329 **HLTMHDHEVI...................FWIGDLNYRLSEIDINDVKKYIDSKVYSKLFPYDQLYKQLGKSDVFKGFTEGDISFRPTYKYDTGTDNWDSSEKCRAPAWCDRILWKGSAVCQSCYRSHNELKISDHKPVSSIF** 452

**Annelida**  192 **--TISDHELVpcfffffssensllfcsivIWLGDLNYRLSDIDNDACKKLISDGRLQELMKYDQLVRQISQKNVFLDYLEGKISFIPTYKYDPGTDTWDTSEKCRTPAWCDRILWRGSNVGQLCYRSHMACKLSDHKPVSSIF** 332

**position**  . 440 . 450 . 460 . 470 . 480 . 490 . 500 . 510 . 520 . 530 . 540 . 550 .

**_**

**_**

**_ _**

**_ _**

**_ _**

**__ _**

**__ _ _ _**

**__ _ _ _**

**__ _ _ _**

**__ _ _ _**

**__ _ _ _**

**__ _ _ _**

**__ _ _ _**

**__ _ _ __**

**__ _ _ __**

**__ _ _ __**

**__ _ _ __**

**__ _ _ ___**

**__ _ _ ___**

**__ _ _ ___**

**__ _ _ ___**

**__ _ _ ___**

**__ _ _ _ ___**

**__ _ _ _ ___**

**__ _ _ _ ___**

**__ _ _ _ ___**

**__ _ _ _ ___**

**____ _ _ ___**

**____ _ _ ___**

**_ ____ _ _ ___**

**_ ____ _ _ ___**

**_ ____ _ _ ___**

**_ ____ _ _ ___**

**_ ____ _ _ ___**

**_ ____ _ _ ___**

**_ ____ _ _ ___ _**

**__ ____ _ _ ___ __**

**__ ____ _ _ ___ __**

**__ ____ _ _ ___ __**

**__ ____ __ _ ___ __ _**

**__ ____ __ __ _ ___ __ _**

**__ ____ __ __ _ ___ __ _**

**__ ____ __ __ _ ___ __ _**

**__ ____ __ __ __ ___ __ _**

**__ ____ __ __ __ ___ __ _**

**__ ____ _ __ __ __ ___ __ _**

**__ ____ __ __ __ __ ___ __ _**

**__ ____ __ __ __ __ ___ __ _**

**__ ____ _ ___ _ _ __ __ __ ___ __ _**

**_______ _ ___ _ _ __ _ __ __ ___ __ _**

**_______ _ ___ _ _ __ _ __ __ ___ __ _**

**EEP**  ●●●●●●● ● ●●● ● ● ●● ● ●● ●● ●●● ●● ●

**4cmlA_human**  433 **PLTISNHDVI...................LWLGDLNYRIEELDVEKVKKLIEEKDFQMLYAYDQLKIQVAAKTVFEGFTEGELTFQPTYKYDTGSDDWDTSEKCRAPAWCDRILWKGKNITQLSYQSHMALKTSDHKPVSSVF** 556*

**XP_011146720.1**  376 **PKSFKDHDQI...................YWLGDLNYRITEMDVNVAKHHIAEGNYSPVLTYDQLGQQRKAGRVFHGFHEAEIDFKPTYKYDPGTDNWDSSEKCRAPAWCDRVLWKGDAIKSISYKSYMELKISDHKPVSASF** 499

**XP_011671046.1**  594 **PLGVMQHDVV...................IWMGDLNYRINDLAVDIVKALIDNNHFKELLLQDQLNRQRELSRVFKGFDEAPIGFLPTYKYNSGSDDWDSSEKQRVPAWCDRILHRGACIVPKVYRSHMKLRLSDHKPVSALY** 717

**XP_012563558.1**  503 **HLSISDHDVI...................IWMGDLNYRFEELDPDQVKKLSDDMDYDKLYLNDQLNWQRSLGKVFEGFSEGQINFKPTYKYDPGTDNWDTSEKFRAPAWCDRILWKGKNIQQITYRSHVELRLSDHKPVSSLF** 626

**XP_006812142.1**  411 **PLHISQHDAI...................FWLGDLNYRISELSADEVKGFIRKDMLRDVYEYDQLNRQMRIQKAFIGFTEGEINFLPTYKYDSGTDSWDSSEKNRAPAWCDRILYRGESIRQLKYRGHMALKISDHKPVSSIF** 534

**XP_013382922.1**  441 **PQSITDHERI...................FWLGDLNYRISGLDLERVKKECDAGNYSVLYEYDQLIQQRSLKNVFLDYEEAPIKFKPTYKYDTGTDSWDTSEKCRCPAWCDRVMWKGEHIENEAYRSHPILKTSDHKPVSAYF** 564

**XP_014679357.1**  417 **PLPIWQHDVV...................LWIGDLNYRLDQLAYDQVMLLMEKGAFAKLLEYDQLYTQKKKGRAFKTYEEGPITFRPTYKFDPGSDTYDTSEKCRVPAWCDRVLWKGQHVKQLTYRSHPTLMISDHKPVSALF** 540

**XP_014778567.1**  329 **HLTMHDHEVI...................FWIGDLNYRLSEIDINDVKKYIDSKVYSKLFPYDQLYKQLGKSDVFKGFTEGDISFRPTYKYDTGTDNWDSSEKCRAPAWCDRILWKGSAVCQSCYRSHNELKISDHKPVSSIF** 452

**ELT95411.1**  192 **--TISDHELVpcfffffssensllfcsivIWLGDLNYRLSDIDNDACKKLISDGRLQELMKYDQLVRQISQKNVFLDYLEGKISFIPTYKYDPGTDTWDTSEKCRTPAWCDRILWRGSNVGQLCYRSHMACKLSDHKPVSSIF** 332

**foreground (45254):**  **GSSTA GDPV VVCGDFNAAHGSDDYENPKDLVSAGNWSALLANDQLTRAAFRRLL AGFTDAWRAGGYSWKSDRGSSNYDSGEKKGPGSRIDRVFASGGLDKVVSAGVDDD RGSDHAPVVATF**

**PRTL DE L LLM L IRPDE PLKD LR LKKKELEK KF E L EML L L LVELF PPPTFPYY NTDEF TY RR PL L YILVRPD LRLLDVRILPE P L LLLEL**

**D I I IIL V D I VK IEQNDFDE EY R L VY FAF YWD ND S N H YTKS YE L F I VDI**

**wt_res_freqs (10787): 11111 1133 221995911112121111114211111118511989131111132 1421421211122211124311562137111214491121311211211212111 118881641312**

**1111 11 1 131 2 11311 1111 11 21111111 11 1 1 111 1 1 31111 11161111 13311 31 11 11 2 2442111 11111112111 1 1 21113**

**1 4 1 411 1 1 1 11 31112111 31 1 1 11 111 211 11 5 1 2 1111 11 1 1 1 111**

**insertions**

**deletions 9999375431 12212332344999999999999999999944443234443223356434322333399999999993433221111224334254444444433232222222221**

**position**  . 440 . 450 . 460 . 470 . 480 . 490 . 500 . 510 . 520 . 530 . 540 . 550 .

**_**

**_**

**_**

**_ _**

**_ _**

**_ _**

**_ _**

**__ _ _**

**__ _ _ _**

**__ _ _ _ _**

**__ _ _ _ _**

**__ _ _ _ _**

**__ _ _ _ _ _**

**__ __ _ _ _ _ _ _**

**__ __ _ _ _ _ _ _ _**

**__ __ _ _ _ _ _ _ _ _**

**__ __ _ _ _ _ _ _ _ _**

**__ __ _ _ _ _ _ _ _ _**

**__ __ _ _ _ _ _ _ _ _**

**__ __ _ _ _ _ _ _ _ _**

**__ __ _ _ _ _ _ _ _ _ _**

**__ __ _ _ _ _ _ _ _ _ _**

**__ ___ _ _ _ _ _ _ _ _ _**

**__ ___ _ _ _ _ _ _ _ _ _**

**__ ___ _ _ _ _ _ _ _ _ _ _**

**__ ___ _ _ _ _ _ _ _ _ _ _**

**__ ___ _ _ _ _ _ _ _ _ _ _**

**__ ___ _ _ _ _ _ _ _ _ _ _**

**___ ___ _ _ _ _ _ __ _ _ _ _ _**

**___ ___ _ _ _ _ _ __ _ _ _ _ _**

**___ ___ _ _ _ _ _ __ _ _ _ _ _**

**___ ___ _ _ _ _ _ __ _ _ _ _ _**

**___ ___ _ _ _ _ _ __ _ ___ _ _**

**___ _ ___ _ _ _ _ _ __ _ ___ _ _**

**___ _ ___ _ _ _ _ _ __ _ ___ _ _**

**___ _ ___ _ _ _ _ _ __ _ ___ _ _**

**___ _ ___ _ _ _ _ _ __ _ ___ _ _**

**___ _ ___ _ _ _ _ _ __ _ ___ _ _**

**_ ___ _ ___ _ _ _ _ _ __ _ ___ _ _**

**_ ___ _ ___ _ _ _ _ _ ___ _ ___ _ _**

**_ ___ _ ___ _ _ _ _ _ ___ _ ___ _ _**

**_ ___ _ ___ _ _ _ _ _ ___ _ ___ _ _**

**_ ___ _ ___ _ _ _ _ _ ___ _ _ ___ _ _**

**_ ___ _ ___ _ _ _ _ _ ___ _ _ ___ _ _ _**

**_ ___ _ ___ _ _ _ _ _ ___ _ _ ___ _ _ _**

**_ ___ _ ___ _ _ _ _ _ ___ _ _ ___ _ _ _ _**

**_ ___ _ ___ _ _ _ _ _ ___ _ _ ___ _ _ _ _**

**_ ___ _ ___ _ _ _ _ _ ___ _ _ _ ___ _ _ _ _**

**_ ___ _ ___ _ _ _ _ _ ___ __ _ _ ___ _ _ _ _**

**_ _ ___ _ ___ _ _ _ _ _ ___ __ _ _ ___ _ _ _ _**

**_ _ ___ _ ___ _ _ _ _ _ ___ __ _ _ ___ _ _ _ _**

**INPP5**  ● ● ●●● ● ●●● ● ● ● ● ● ●●● ●● ● ● ●●● ● ● ● ●

**4cmlA_human**  433 **PLTISNHDVI...................LWLGDLNYRIEELDVEKVKKLIEEKDFQMLYAYDQLKIQVAAKTVFEGFTEGELTFQPTYKYDTGSDDWDTSEKCRAPAWCDRILWKGKNITQLSYQSHMALKTSDHKPVSSVF** 556*

**XP_011146720.1**  376 **PKSFKDHDQI...................YWLGDLNYRITEMDVNVAKHHIAEGNYSPVLTYDQLGQQRKAGRVFHGFHEAEIDFKPTYKYDPGTDNWDSSEKCRAPAWCDRVLWKGDAIKSISYKSYMELKISDHKPVSASF** 499

**XP_011671046.1**  594 **PLGVMQHDVV...................IWMGDLNYRINDLAVDIVKALIDNNHFKELLLQDQLNRQRELSRVFKGFDEAPIGFLPTYKYNSGSDDWDSSEKQRVPAWCDRILHRGACIVPKVYRSHMKLRLSDHKPVSALY** 717

**XP_012563558.1**  503 **HLSISDHDVI...................IWMGDLNYRFEELDPDQVKKLSDDMDYDKLYLNDQLNWQRSLGKVFEGFSEGQINFKPTYKYDPGTDNWDTSEKFRAPAWCDRILWKGKNIQQITYRSHVELRLSDHKPVSSLF** 626

**XP_006812142.1**  411 **PLHISQHDAI...................FWLGDLNYRISELSADEVKGFIRKDMLRDVYEYDQLNRQMRIQKAFIGFTEGEINFLPTYKYDSGTDSWDSSEKNRAPAWCDRILYRGESIRQLKYRGHMALKISDHKPVSSIF** 534

**XP_013382922.1**  441 **PQSITDHERI...................FWLGDLNYRISGLDLERVKKECDAGNYSVLYEYDQLIQQRSLKNVFLDYEEAPIKFKPTYKYDTGTDSWDTSEKCRCPAWCDRVMWKGEHIENEAYRSHPILKTSDHKPVSAYF** 564

**XP_014679357.1**  417 **PLPIWQHDVV...................LWIGDLNYRLDQLAYDQVMLLMEKGAFAKLLEYDQLYTQKKKGRAFKTYEEGPITFRPTYKFDPGSDTYDTSEKCRVPAWCDRVLWKGQHVKQLTYRSHPTLMISDHKPVSALF** 540

**XP_014778567.1**  329 **HLTMHDHEVI...................FWIGDLNYRLSEIDINDVKKYIDSKVYSKLFPYDQLYKQLGKSDVFKGFTEGDISFRPTYKYDTGTDNWDSSEKCRAPAWCDRILWKGSAVCQSCYRSHNELKISDHKPVSSIF** 452

**ELT95411.1**  192 **--TISDHELVpcfffffssensllfcsivIWLGDLNYRLSDIDNDACKKLISDGRLQELMKYDQLVRQISQKNVFLDYLEGKISFIPTYKYDPGTDTWDTSEKCRTPAWCDRILWRGSNVGQLCYRSHMACKLSDHKPVSSIF** 332

**foreground (3601):**  **GSSTLSHDHV FWFGDFNYRVCSGSGDDAKDLVSNGNWSSLLANDQLTQAKEAGKAFQGWSEGEITFAPTYKYDRGSSNYDSGSKQRAPAWCDRVLWKGKGVSQLSYGACESYTTSDHKPVSGTF**

**PRTL DFEVL ILL L F LEELPYEHILR LKKRELEK KF E LLQMRKNPILH FR APLK P RFEPNTDEF TSE R I SYT I FRSRPLRL RRHPLLRI R FSL**

**D I YI F ITDDDR EVRK IEQKDFDE EY RIERK RV R YQ N L L ND K V Y RIK DSAMEIKF YAI**

**wt_res_freqs (905): 1111215614 563992969121111211114211111118511989111112512715119416392999855153115621181919685992833311213329111111129994992117**

**1111 41111 213 7 2 32132121111 21111111 11 1 11221111111 51 2311 3 131113311 354 1 1 314 7 14211211 12221111 4 111**

**1 4 12 1 413111 2421 31112111 31 11322 13 1 11 1 1 1 11 3 1 3 122 14122111 151**

**insertions**

**deletions 2322211111 1111111 19 1111111111111111111111111 111 111 11442121113223344444432222322333327111289911111998**

**background (41653):**  **TNQRAAGAPV VVCGDFNAAHGSDAYGNPTKCASAGNNTALREYDQLERAAFRRLLAAGFTDAWRAGGYSYWAYRNSSEGDSYMKNGPGSRIDRVFASGGLDKVKSAGVDDD RGSDHAAVVATF**

**IRRIPKDE L LLM L ITPDEIDLKD VLLIKKKMFRW LKF L ELL L EL LV LF P FTFPD DPDQPKQ NRD L L YILLRPD LRLVDVEILPE P LPLLLEL**

**ED V I IIL V D I KRTVEETEQKS Q K D VY W S IN TD QKN W H VTKS S R L F IIVDI**

**wt_res_freqs (9882): 1133111143 22199591111211111111211111122814397813111113211411521211122111121222411772111214491121311311211213111 118881141212**

**11111111 1 131 2 1131112111 23212221113 524 1 111 1 11 31 11 1 15111 6112212 311 1 3 3431111 11111112111 1 1621123**

**24 1 1 421 1 1 1 21111121111 1 4 1 11 3 1 11 11 111 1 2 2111 1 1 1 1 11211**

**position**  . 440 . 450 . 460 . 470 . 480 . 490 . 500 . 510 . 520 . 530 . 540 . 550 .

**_**

**_**

**_**

**_**

**_**

**_**

**_**

**_**

**_**

**_**

**_**

**_**

**_**

**_**

**_**

**_ _**

**_ _ _**

**_ _ _ _**

**_ _ _ _ _ _ _ _**

**_ __ _ _ _ _ _ _**

**_ __ _ _ _ _ _ _**

**INPP5b subfamily**  ● ●● ● ● ● ● ● ●

**4cmlA_human**  433 **PLTISNHDVI...................LWLGDLNYRIEELDVEKVKKLIEEKDFQMLYAYDQLKIQVAAKTVFEGFTEGELTFQPTYKYDTGSDDWDTSEKCRAPAWCDRILWKGKNITQLSYQSHMALKTSDHKPVSSVF** 556*

**XP_011146720.1**  376 **PKSFKDHDQI...................YWLGDLNYRITEMDVNVAKHHIAEGNYSPVLTYDQLGQQRKAGRVFHGFHEAEIDFKPTYKYDPGTDNWDSSEKCRAPAWCDRVLWKGDAIKSISYKSYMELKISDHKPVSASF** 499

**XP_011671046.1**  594 **PLGVMQHDVV...................IWMGDLNYRINDLAVDIVKALIDNNHFKELLLQDQLNRQRELSRVFKGFDEAPIGFLPTYKYNSGSDDWDSSEKQRVPAWCDRILHRGACIVPKVYRSHMKLRLSDHKPVSALY** 717

**XP_012563558.1**  503 **HLSISDHDVI...................IWMGDLNYRFEELDPDQVKKLSDDMDYDKLYLNDQLNWQRSLGKVFEGFSEGQINFKPTYKYDPGTDNWDTSEKFRAPAWCDRILWKGKNIQQITYRSHVELRLSDHKPVSSLF** 626

**XP_006812142.1**  411 **PLHISQHDAI...................FWLGDLNYRISELSADEVKGFIRKDMLRDVYEYDQLNRQMRIQKAFIGFTEGEINFLPTYKYDSGTDSWDSSEKNRAPAWCDRILYRGESIRQLKYRGHMALKISDHKPVSSIF** 534

**XP_013382922.1**  441 **PQSITDHERI...................FWLGDLNYRISGLDLERVKKECDAGNYSVLYEYDQLIQQRSLKNVFLDYEEAPIKFKPTYKYDTGTDSWDTSEKCRCPAWCDRVMWKGEHIENEAYRSHPILKTSDHKPVSAYF** 564

**XP_014679357.1**  417 **PLPIWQHDVV...................LWIGDLNYRLDQLAYDQVMLLMEKGAFAKLLEYDQLYTQKKKGRAFKTYEEGPITFRPTYKFDPGSDTYDTSEKCRVPAWCDRVLWKGQHVKQLTYRSHPTLMISDHKPVSALF** 540

**XP_014778567.1**  329 **HLTMHDHEVI...................FWIGDLNYRLSEIDINDVKKYIDSKVYSKLFPYDQLYKQLGKSDVFKGFTEGDISFRPTYKYDTGTDNWDSSEKCRAPAWCDRILWKGSAVCQSCYRSHNELKISDHKPVSSIF** 452

**ELT95411.1**  192 **--TISDHELVpcfffffssensllfcsivIWLGDLNYRLSDIDNDACKKLISDGRLQELMKYDQLVRQISQKNVFLDYLEGKISFIPTYKYDPGTDTWDTSEKCRTPAWCDRILWRGSNVGQLCYRSHMACKLSDHKPVSSIF** 332

**foreground (252):**  **QKSFSKHDQV YWLGDLNYRICDLDAAEAKSLVAEGAYQAVYAYDQLGQQRAAGAAFAGFTEGDISFQPTYKYDAGSDNWDSSGKCRAPAWCDRVLWKGGNVTSISYQSHMALKTSDHKPVSAVF**

**PLNIMD VI L LEMP VEKV K IEKKEL PLLKF KI MKLKRV VD M AELK I PKT R T E V I R K IKQLH R PE I SL**

**T V I TLM D Q D NDF K E NR VDQNK E N K T D N R D**

**wt_res_freqs (53): 1111179815 198999999421484131917113121411225998119512123925849818193999999152829959388939999992995916321139299739869999999329**

**662831 74 3 5212 4228 2 722313 17411 34 111515 14 1 1712 3 235 3 3 6 4 7 4 3 61871 5 13 2 55**

**4 1 3 111 1 2 1 224 2 1 31 11213 2 1 1 3 2 2 1 1**

**insertions 1 1 1 1**

**deletions 11111111111 1 1 33333333333333222112223333333333344434444333332333223333333344443**

**background (3349):**  **GSSTLSHDHV FWFGDFNYRVTSGSGDDAKDLVSNGNWSSLLANDELTQQKEAGKAFQGWSEGEITFAPTYKYDRGSSNYDSGSKQRAPAWCDRVLWKGKGVSCLSYGACESLTTSDHKPVSGTF**

**PRVL DFELL ILL L F LEELPYEHILR LKKRELEK KY Q LLAMRKNPILH FR APLN P RFEPNTDEF TSE R I SYT I FRSRPLRP RRHPLYRI R FSLL**

**DTI YI VF IDDDTR EVRK IEQKDFDE EH RIERKS RV R YQ L L ND K V Y RIKL DSAMEIKV YAI**

**wt_res_freqs (852): 1111215614 563992969111121211113211111118511919111112512715119416392999855153115621171919585992832211211329111111129993991217**

**1111 41111 212 6 3 32132121111 21111111 11 8 11121111111 51 2311 3 131113311 354 1 1 314 7 13211211 12221111 5 1111**

**114 12 11 423111 2421 31112111 31 114221 13 1 11 1 1 11 3 1 3 1222 14122211 151**

**position**  . 440 . 450 . 460 . 470 . 480 . 490 . 500 . 510 . 520 . 530 . 540 . 550 .

**Figure 5— Source data 1. EEP superfamily, INPP5 family and INPP5e subfamily.**

**Mammal**  303 **VATWNMQGQKELPPSLDEFLLPAEADYAQDLYVIGVQEGCSDRREWETRLQETLGPHYVLLSSAAHGVLYMSLFIRRDLIWFCSEVECSTVTTRIVSQIKTKGALGISFTFFGTSFLFITSHFTSGDGKVAERLLDYTRTVQALVLPRNVPDTNPYRSSAADVTTRFDEVFWF** 475*

**Reptile**  287 **VATWNMQGQKELPENLDDLLLPTDPDYAQDMYIIGVQEGCPDRREWEIRLQETLGPHYVMLFSAAHGVLYMSVFIRRDLIWFCSEVEYSTVTTRIVSQIKTKGALGISFTFFGTSFLFITSHFTSGDSKVYERILDYNKIIQALALPKNVPDTNPYRSSPSDVTTRFDEVFWF** 459

**Fish**  61 **IATWNMQGEKGLPYNLDDLLLPTDTDFAQDVYVIGVQEGCPDRREWEIRLQETLGPYYVMLYAAAHGVLYLTVFVRRDLIWFCSEVEHATVTTRIISQIKTKGAVGIGFTFFGTSFLFVTSHFTSGDSKVYERILDYNKIIEALALPRNLPDTNPYRSTTSDVTTRFDEVFWF** 233

**Bird**  318 **VATWNMQGQKELPVNLDDFLLPTDPDYAQDMYVIGVQEGCPDRREWEIRLQETLGPHYVMLYSAAHGVLYMSVFIRRDLIWFCSEVEYATVTTRIVSQIKTKGALGICFTFFGTSFLFITSHFTSGDSKVNERKMDYSKTIQALALPRNVPDTNPYRSSSSDVTTRFDEVFWF** 490

**Amphibian**  265 **IATWNMQGRKELPESLDDFLLPSDDDFAQDMYVIGVQEGCPDRREWEIRLQETLGPHYVLLHSSGHGVLYLSVFLRRDLIWFCSEVESDTVTTRIVSQIKTKGALAVSFTFFGTSFLFITSHFTSGDSKVCDRILDYKKIIEGLQLPRMIPDTNPYRSNSADVTSRFDEVFWF** 437

**Echinodermata**  591 **VATWNMHEDKEIPENLDDLLLPEDIEYMQDVYVIGTQESSPDIAEWEIRLQETLGPSHVLLHSAAHGVLQCVVFIRRDLIWFCSPVEESRVSTRPGSMIKTKGAIAVSFNFFGTSFIFIVSHFTSGDENMKERLLDYEKILKGISLPEKVPPTRKFNVLTSDVTTRFDCVFWC** 763

**position**  . 310 . 320 . 330 . 340 . 350 . 360 . 370 . 380 . 390 . 400 . 410 . 420 . 430 . 440 . 450 . 460 . 470 .

**_**

**_ _ _**

**_ _ _**

**_ _ _**

**_ _ _**

**_ _ _**

**_ _ _**

**_ _ _**

**_ _ _**

**_ _ _**

**_ _ _**

**_ _ _**

**_ _ _**

**_ _ _**

**_ _ _**

**_ _ _**

**_ _ _**

**_ _ _**

**_ _ _**

**_ _ _**

**_ _ __ _**

**_ _ _ __ _**

**_ _ _ __ _**

**_ _ _ __ _**

**_ _ _ __ _**

**_ _ _ _ _ __ _**

**_ ___ _ _ __ _**

**_ ___ _ _ ___ _ _**

**_ ___ _ _ ___ _ _ _**

**_ ___ _ _ ___ _ _ _**

**_ ___ _ __ ___ _ _ _**

**_ ___ _ __ ___ _ _ _ _**

**_ ___ _ __ ___ _ _ _ _**

**_ ____ _ __ ___ _ _ _ _**

**_ ____ _ __ ___ _ _ _ _**

**_ ____ _ __ ___ _ _ _ __**

**_ ____ _ __ ___ _ _ _ _ __**

**_ ____ _ __ ___ _ _ _ _ _ _ __**

**_ ____ _ __ ___ _ _ _ _ _ _ __**

**_ ____ _ _ __ ___ __ _ _ _ _ _ __**

**_ ____ _ _ __ ___ __ _ _ _ _ _ __**

**_ ____ _ _ __ ___ __ _ _ _ _ _ __**

**______ _ _ _ __ ___ __ _ _ _ _ _ __**

**______ _ _ _ __ ___ __ _ _ _ _ _ __**

**______ _ _ _ __ ___ __ _ _ _ _ _ __**

**______ _ _ _ __ ___ __ _ _ _ _ _ _ __**

**______ _ _ __ __ ___ __ _ _ _ _ _ _ __**

**______ _ _ __ _ __ ___ __ _ _ _ _ _ _ _ __**

**______ _ _ __ _ __ ___ __ _ _ _ _ _ _ _ __**

**EEP** ●●●●●● ● ● ●● ● ●● ●●● ●● ● ● ● ● ● ● ● ●●

**2xswA_human**  303 **VATWNMQGQKELPPSLDEFLLPAEADYAQDLYVIGVQEGCSDRREWETRLQETLGPHYVLLSSAAHGVLYMSLFIRRDLIWFCSEVECSTVTTRIVSQIKTKGALGISFTFFGTSFLFITSHFTSGDGKVAERLLDYTRTVQALVLPRNVPDTNPYRSSAADVTTRFDEVFWF** 475*

**XP_006278611.1**  287 **VATWNMQGQKELPENLDDLLLPTDPDYAQDMYIIGVQEGCPDRREWEIRLQETLGPHYVMLFSAAHGVLYMSVFIRRDLIWFCSEVEYSTVTTRIVSQIKTKGALGISFTFFGTSFLFITSHFTSGDSKVYERILDYNKIIQALALPKNVPDTNPYRSSPSDVTTRFDEVFWF** 459

**AAI50239.1**  61 **IATWNMQGEKGLPYNLDDLLLPTDTDFAQDVYVIGVQEGCPDRREWEIRLQETLGPYYVMLYAAAHGVLYLTVFVRRDLIWFCSEVEHATVTTRIISQIKTKGAVGIGFTFFGTSFLFVTSHFTSGDSKVYERILDYNKIIEALALPRNLPDTNPYRSTTSDVTTRFDEVFWF** 233

**XP_013801144.1**  318 **VATWNMQGQKELPVNLDDFLLPTDPDYAQDMYVIGVQEGCPDRREWEIRLQETLGPHYVMLYSAAHGVLYMSVFIRRDLIWFCSEVEYATVTTRIVSQIKTKGALGICFTFFGTSFLFITSHFTSGDSKVNERKMDYSKTIQALALPRNVPDTNPYRSSSSDVTTRFDEVFWF** 490

**XP_004916697.1**  265 **IATWNMQGRKELPESLDDFLLPSDDDFAQDMYVIGVQEGCPDRREWEIRLQETLGPHYVLLHSSGHGVLYLSVFLRRDLIWFCSEVESDTVTTRIVSQIKTKGALAVSFTFFGTSFLFITSHFTSGDSKVCDRILDYKKIIEGLQLPRMIPDTNPYRSNSADVTSRFDEVFWF** 437

**XP_011670872.1**  591 **VATWNMHEDKEIPENLDDLLLPEDIEYMQDVYVIGTQESSPDIAEWEIRLQETLGPSHVLLHSAAHGVLQCVVFIRRDLIWFCSPVEESRVSTRPGSMIKTKGAIAVSFNFFGTSFIFIVSHFTSGDENMKERLLDYEKILKGISLPEKVPPTRKFNVLTSDVTTRFDCVFWC** 763

**foreground (45175):**  **VASWNVNGFNARADGVAAWVAAENADDDPDFYVVCVQETKDSDGAFLALAAAAGGYHYYYSGAKGGGGSGVGVFSKKSKAPRFIVVSVETGLGGDGGADGSGGAVAATFEVGGTTFTFASAHFPAGGGKLAAALAQAAAFAARVAALAAVPDTNPYRSSAGSSTA GDPVVVC**

**LMTF LLSLR PPALLRLLRRLDP EP LLLLAL L DQAQLPL LLELP VFP R K YI LALLVRYPLL FILPLE V PSEEN L ERRVLVLRLRLP RPLVLINLYL N SSR EER RFLEELLELLKK LKL F DPPRTL DE LLLM**

**IL Y IR I LK I E IKE IIIIG I L V S E I Y ERI HV L D L NK I VEIKIK K IRIV V S D K EWRD I D I E TT D I IIIL**

**wt_res_freqs (10915): 423394231113111111111111271147144321791111111111111111331111121111221531123311112111111111111211612111221131211141121111161312111111111211111111112115989788784211111 1133221**

**1141 11122 1113111311123 11 211216 1 1111112 11111 111 1 1 11 1413131211 111111 1 11111 1 132121112111 1121213124 1 112 113 111113211311 112 1 231111 11 1131**

**32 2 21 1 11 2 1 211 41322 1 1 1 1 1 3 1 111 11 1 1 1 11 1 211111 1 1112 2 2 1 2 1111 1 1 1 1 11 1 4 1411**

**insertions**

**deletions 211111111118777555544122229999991111113546777613388965522223333432263221111111499953122222333123933143222222223333211 1 135112115555442222233345526999999999999999375431**

**position**  . 310 . 320 . 330 . 340 . 350 . 360 . 370 . 380 . 390 . 400 . 410 . 420 . 430 . 440 . 450 . 460 . 470 .

**_**

**_ _**

**_ _**

**_ _**

**_ _**

**_ _**

**_ _**

**_ _**

**_ _**

**_ _**

**_ _**

**_ _ _**

**__ _ _**

**__ _ _**

**_ ___ _ _**

**_ ___ _ _**

**_ ____ _ _**

**_ ____ _ _**

**_ ____ _ _**

**_ _ ____ _ _**

**_ _ _ ____ _ _**

**_ _ _ ____ _ _**

**_ _ _ ____ _ _**

**_ _ _ ____ _ _ _**

**_ _ _ ____ _ _ _ _**

**_ _ _ _ ____ _ _ _ _ _**

**_ _ _ _ ____ _ _ _ _ _ _**

**_ _ _ _ ____ _ _ _ _ _ _ _ _**

**_ _ _ _ _ ____ _ _ _ _ _ _ _ _**

**_ _ _ _ _ _____ _ _ _ _ _ _ _ _**

**_ _ _ _ _ _____ _ _ _ _ _ _ _ _**

**_ _ _ _ _ _ _____ _ _ _ _ _ _ _ _**

**_ _ _ _ _ _ _ _____ _ _ _ _ _ _ _ _**

**_ _ _ _ _ _ _ _____ _ _ _ _ _ _ _ _**

**_ _ _ _ _ _ _ _____ _ _ _ _ _ _ _ _**

**_ _ _ _ _ _ _ _ _ _____ _ _ _ __ _ _ _ _ _**

**_ _ _ _ _ _ _ _ _ _____ _ _ _ __ _ _ _ _ _**

**INPP5 family**  ● ● ● ● ● ● ● ● ● ●●●●● ● ● ● ●● ● ● ● ● ●

**2xswA_human**  303 **VATWNMQGQKELPPSLDEFLLPAEADYAQDLYVIGVQEGCSDRREWETRLQETLGPHYVLLSSAAHGVLYMSLFIRRDLIWFCSEVECSTVTTRIVSQIKTKGALGISFTFFGTSFLFITSHFTSGDGKVAERLLDYTRTVQALVLPRNVPDTNPYRSSAADVTTRFDEVFWF** 475*

**XP_006278611.1**  287 **VATWNMQGQKELPENLDDLLLPTDPDYAQDMYIIGVQEGCPDRREWEIRLQETLGPHYVMLFSAAHGVLYMSVFIRRDLIWFCSEVEYSTVTTRIVSQIKTKGALGISFTFFGTSFLFITSHFTSGDSKVYERILDYNKIIQALALPKNVPDTNPYRSSPSDVTTRFDEVFWF** 459

**AAI50239.1**  61 **IATWNMQGEKGLPYNLDDLLLPTDTDFAQDVYVIGVQEGCPDRREWEIRLQETLGPYYVMLYAAAHGVLYLTVFVRRDLIWFCSEVEHATVTTRIISQIKTKGAVGIGFTFFGTSFLFVTSHFTSGDSKVYERILDYNKIIEALALPRNLPDTNPYRSTTSDVTTRFDEVFWF** 233

**XP_013801144.1**  318 **VATWNMQGQKELPVNLDDFLLPTDPDYAQDMYVIGVQEGCPDRREWEIRLQETLGPHYVMLYSAAHGVLYMSVFIRRDLIWFCSEVEYATVTTRIVSQIKTKGALGICFTFFGTSFLFITSHFTSGDSKVNERKMDYSKTIQALALPRNVPDTNPYRSSSSDVTTRFDEVFWF** 490

**XP_004916697.1**  265 **IATWNMQGRKELPESLDDFLLPSDDDFAQDMYVIGVQEGCPDRREWEIRLQETLGPHYVLLHSSGHGVLYLSVFLRRDLIWFCSEVESDTVTTRIVSQIKTKGALAVSFTFFGTSFLFITSHFTSGDSKVCDRILDYKKIIEGLQLPRMIPDTNPYRSNSADVTSRFDEVFWF** 437

**XP_011670872.1**  591 **VATWNMHEDKEIPENLDDLLLPEDIEYMQDVYVIGTQESSPDIAEWEIRLQETLGPSHVLLHSAAHGVLQCVVFIRRDLIWFCSPVEESRVSTRPGSMIKTKGAIAVSFNFFGTSFIFIVSHFTSGDENMKERLLDYEKILKGISLPEKVPPTRKFNVLTSDVTTRFDCVFWC** 763

**foreground (3585):**  **VGSWNVGGATEGSDDIASWFGSADASDAPDVYAFGFQEVDSGAAGNVLGAAKASGEGYVKVASESLGGVAICVWAKKSHAPRVSNVAAASTGCGFGGYWGNKGGVGVSFSYGGSSFCFVCCHFAAGESAVAERNANYATIVSGTSFSDGVPDTNPYRSSAGSSTLSHDHVFWF**

**IVTF MNAKKPPPPQLTPLLLPTEPEEP LLVL L LVPLEEKLLMDLDPLLPPKF LLRTVRMV MLLLLFIRPELL FLRHLQVDTVRT MMNMM AISIRMVLFETTLT INS LTSHQEKLER QDVKE LRRMVLPPPL F DPPRTL DFEVLILL**

**Y A Q RS E SE FLS SD D II I I LSN EFI NTSFT D D I KQ Q LF IYV RD V HIKEIETS K L L A LRIYD I TA E DQ E F D KKLR RN TT D I YI F**

**wt_res_freqs (885): 55169534121111111251113451114814218499211111111111111112173232411517112151231121212224112213191371188993723241113143586139166411131296113118111117111598978878421111215614563**

**2181 13114214216112612351112 2152 3 23141111211111121111 33111224 125215131131 112111112515 12111 51136113115121 253 8123221123 17111 3221112112 1 231111 41111213**

**1 1 1 11 2 11 111 11 1 41 3 2 111 112 11111 1 1 1 13 1 31 214 12 1 15112222 3 3 1 3 21112 1 12 1 11 1 2 1 1131 11 11 1 4 12 1**

**insertions**

**deletions 2211111117511111111111999111199999988888765443213355 11111111222111111111231111111112223221222111 1111 11111222111 11 1 113 999999999992322221111111**

**background (41590):**  **VASWNVNGFNARADGVAAEVAAENADHDPDFVVVCVQETKDSDGADLAEAAAAGGYHYYYSGAKGGGGGGVGFFSKKEKVTSVIVLSVEVGLGGDTGADSSGGAVAATFEVGGKTFTVASAHFPAGGGKLAARLAQAAAIAARVAALAA TNQRAAGAPVVVC**

**LMTF LLNLR LKLLLRLLRRLDP LK HILLLLL IR DQDQLPLLLLELP AVFPYR K YE LALLYRYPLLPPFLP E PSEEF L ERRPLVLRLRLP RPLVLINLYL N RSR EYA RFLELLLELLKK LK IRRIPKDE LLLM**

**IL Y IRSI KERI EWIKE K IIIA V N ADF L S I RIVT L D L C VI VEIKIK IRIV V S S DEK EWRDEQ DYI E I ED IIIL**

**wt_res_freqs (10030): 42439422111311111111111128217271433179121111111111111133111112111121163111331113111111111111111111211111113121114111141116131211111131121111111111211 1122111143221**

**1141 11122 1112111311123 23 1121216 11 1111112111111 111111 1 11 151413131113211 1 11111 1 132131112111 1121213134 1 113 111 111113111311 11 11111111 1131**

**32 2 3111 1112 11211 2 3322 3 1 111 1 1 3 1231 1 1 1 5 12 211111 1112 2 2 1 112 111111 111 1 1 23 1421**

**position**  . 310 . 320 . 330 . 340 . 350 . 360 . 370 . 380 . 390 . 400 . 410 . 420 . 430 . 440 . 450 . 460 . 470 .

**_**

**_**

**_**

**_**

**_**

**_**

**_**

**_**

**_**

**_**

**_**

**_**

**_ _**

**_ _**

**_ _**

**_ _**

**_ _**

**_ _**

**_ _**

**_ _ _**

**_ _ _**

**_ _ _**

**_ _ _**

**_ _ _**

**_ _ _**

**_ _ _ _**

**_ _ _ _**

**_ _ _ _**

**_ _ _ _**

**_ _ _ _**

**_ _ _ _**

**_ _ _ _**

**_ _ _ _**

**_ _ _ _ _ _**

**_ _ _ _ _ _ _**

**_ _ _ _ _ _ _ _**

**_ _ _ _ _ _ _ _**

**_ _ _ _ _ _ _ _ _**

**_ _ _ _ _ _ _ _ _ _**

**_ _ _ _ _ _ _ _ _ _**

**_ _ _ _ _ _ _ _ _ __**

**_ _ __ _ _ _ _ _ _ _ _ __**

**_ _ __ _ _ _ _ _ _ _ _ __ _**

**_ _ __ _ _ _ _ _ _ _ _ __ __ _ _**

**_ _ __ _ _ _ _ _ _ _ _ _ _ ___ __ _ _ _**

**_ _ __ ___ _ _ _ _ _ _ _ _ _ ___ __ _ _ _**

**_ _ __ ___ _ _ __ _ _ _ _ _ _ ___ __ _ _ _ __ _**

**_ _ __ ___ _ _ __ _ _ _ _ _ _ ___ __ _ ___ __ _ _**

**_ _ _ __ ___ __ ____ _ _ _ _ _ _ _ _ ___ __ _ _ _ ___ ____ _ __**

**_ _ _ _ __ ___ __ _____ _ __ _ _ __ _ _ _ ___ __ _ _ _ _ ___ ____ _ __**

**_ _ _ _ __ ___ __ _____ _ __ _ _ __ _ _ _ ___ __ _ _ _ _ ___ ____ _ __**

**INPP5e subfamily**  ● ● ● ● ●● ●●● ●● ●●●●● ● ●● ● ● ●● ● ● ● ●●● ●● ● ● ● ● ●●● ●●●● ● ●●

**2xswA_human**  303 **VATWNMQGQKELPPSLDEFLLPAEADYAQDLYVIGVQEGCSDRREWETRLQETLGPHYVLLSSAAHGVLYMSLFIRRDLIWFCSEVECSTVTTRIVSQIKTKGALGISFTFFGTSFLFITSHFTSGDGKVAERLLDYTRTVQALVLPRNVPDTNPYRSSAADVTTRFDEVFWF** 475*

**XP_006278611.1**  287 **VATWNMQGQKELPENLDDLLLPTDPDYAQDMYIIGVQEGCPDRREWEIRLQETLGPHYVMLFSAAHGVLYMSVFIRRDLIWFCSEVEYSTVTTRIVSQIKTKGALGISFTFFGTSFLFITSHFTSGDSKVYERILDYNKIIQALALPKNVPDTNPYRSSPSDVTTRFDEVFWF** 459

**AAI50239.1**  61 **IATWNMQGEKGLPYNLDDLLLPTDTDFAQDVYVIGVQEGCPDRREWEIRLQETLGPYYVMLYAAAHGVLYLTVFVRRDLIWFCSEVEHATVTTRIISQIKTKGAVGIGFTFFGTSFLFVTSHFTSGDSKVYERILDYNKIIEALALPRNLPDTNPYRSTTSDVTTRFDEVFWF** 233

**XP_013801144.1**  318 **VATWNMQGQKELPVNLDDFLLPTDPDYAQDMYVIGVQEGCPDRREWEIRLQETLGPHYVMLYSAAHGVLYMSVFIRRDLIWFCSEVEYATVTTRIVSQIKTKGALGICFTFFGTSFLFITSHFTSGDSKVNERKMDYSKTIQALALPRNVPDTNPYRSSSSDVTTRFDEVFWF** 490

**XP_004916697.1**  265 **IATWNMQGRKELPESLDDFLLPSDDDFAQDMYVIGVQEGCPDRREWEIRLQETLGPHYVLLHSSGHGVLYLSVFLRRDLIWFCSEVESDTVTTRIVSQIKTKGALAVSFTFFGTSFLFITSHFTSGDSKVCDRILDYKKIIEGLQLPRMIPDTNPYRSNSADVTSRFDEVFWF** 437

**XP_011670872.1**  591 **VATWNMHEDKEIPENLDDLLLPEDIEYMQDVYVIGTQESSPDIAEWEIRLQETLGPSHVLLHSAAHGVLQCVVFIRRDLIWFCSPVEESRVSTRPGSMIKTKGAIAVSFNFFGTSFIFIVSHFTSGDENMKERLLDYEKILKGISLPEKVPPTRKFNVLTSDVTTRFDCVFWC** 763

**foreground (105):**  **VATWNMQGEKGLPASLDDFLLPADADTAQDFYVVGVQEGCSDRREWETRLQETLGPQYVLLSAAAHGVLYLSVFVRRDLIWFCSEVECATVTTRIVSQIKTKGAVGVCFTFFGTSFLFITSHFTSGDGKVAERILDYSKTVEGLALPKSVPDTNPYRSSAADVTTRFDGVFWF**

**IV Q E PN EL TEPEFT M II P I Y M YS S A MTLLL YS I L IS S Y L NRIIQA T RNL F DPS Q**

**V S S Y L H H I H G N T TT E**

**wt_res_freqs (24): 88999999291991298549993358179929729899995999999499999999199492287998994668199998899999925999999798999999295199999999999999999996993992899266321979941598978878423989999919999**

**11 6 7 35 45 451121 2 17 4 3 1 5 56 1 1 42211 14 2 7 34 2 3 5 332667 1 462 1 234 1**

**1 1 1 4 3 5 1 6 2 2 1 2 11 5**

**insertions 3**

**deletions 6666666663222233333333333333333555555555552 11111111111111111111112222222222222222321222222222222222222111111111111111111111 111111111111111111111111112222222223444444444444**

**background (3480):**  **VGSWNVGGGTRGSDDIASWFGSFDISDDPDVYAFGFQEVDSGAAGNVLGAAKASGEGYEKVASEQLGGVAICVWAKKSHAPRVSNVAAASTGCGFGGYWGNKGGVGVSFSYGGSSFCFVCCHFAAGESAVDERNANYATIVSGTSFSDG GSSILSHDHVFWF**

**IVTF MNARKPPPPQLTPLLLP EEP LLVL L LVPLEEKLLMDTDPLLP KFVLLRTVRMV MLLLLFIRPELL FLRHLQVDTVRT MMNMM AISIRMVLYETTLT INS LTSHQEKLER QDVKE LRRMVLPPP PRTL DFEVLILL**

**Y A K S E SD FL D II I I LSN EFI NSTFS K D I K Q LF IYV RD V YIKEIETS K L L A LRIHD I TA Q Q E F D KKLR R D YIVF**

**wt_res_freqs (861): 55169534121111111251118881114814218499211111111111111112171232413517112151241121212224112213191371189993723241112143586139167411121296113118111117111 1114215614562**

**2181 13114214216112612 112 2152 4 23141111211111121 11333111224 125215131132 112111111515 12112 51136113115121 252 8113221123 17111 322111211 1111 41111213**

**1 2 1 1 2 12 11 1 41 3 2 111 112 11111 1 1 1 1 1 31 214 12 1 15112222 3 3 1 3 21112 1 12 1 1 1 2 1 1131 1 1 1211**

**position**  . 310 . 320 . 330 . 340 . 350 . 360 . 370 . 380 . 390 . 400 . 410 . 420 . 430 . 440 . 450 . 460 . 470 .

**Mammal**  476 **GDFNFRLSGGRTVVDALLCQGLVv...DVPALLQHDQLIREMRKGSIFKGFQEPDIHFLPSYKFDIGKDTYDSTSKQRTPSYTDRVLYRSRHKGDICPVSYSSCPGIKTSDHRPVYGLF** 591*

**Reptile**  460 **GDFNFRLNQDREAVNLILNQNLEm...DMSKLLQYDQLLKEMKNGSVFKGFQEAAIHFHPSYKFDIGQDCYDTTSKQRTPSYTDRVVYRSRHKNDIHAVKYSSCPIIRTSDHRPVYGLF** 575

**Fish**  234 **GDFNFRLNKARGDVEAILNQGVGv...DMSPLLQHDQLTREMKEGSIFKGFQEASIHFPPTYKFDIGCDVYDTTTKQRTPSYTDRILYRNRQSDDIRVIKYTSCSSIKTSDHRPVIGMF** 349

**Bird**  491 **GDFNFRLNKDRETVDSILKQNLEk...DMSKLLEYDQLISEMSRGSIFKGFQEADIHFCPSYKFDIGKDSYDTTSKQRTPSYTDRVVYRSRHKDDIHAVKYSSCPVIKTSDHRPVFALF** 606

**Amphibian**  438 **GDFNFRLNKERSAVNSILQSKLEk...DMSRLLQYDQLTKEMNGESIFKGFQEAEIQFLPTYKFDIGCDVYDSTSKQRTPSYTDRVVYKSRHKDDIRVVKYASCSLIKTSDHRPVFGLF** 553

**Echinodermata**  764 **GDFNFRLAENKARIEGWIDQLRRgnkdDYDILLQYDQLNKAMQRNEIFQGFHEGKIEFLPTYKFDIGEDIYDTSAKARVPSYTDRVLYKSRKVSDISLIHYSSCEEIKTSDHRPVYALF** 882

**position**  480 . 490 . 500 . 510 . 520 . 530 . 540 . 550 . 560 . 570 . 580 . 590

**_**

**_**

**_ _**

**_ _**

**__ _**

**__ _ _ _**

**__ _ _ _**

**__ _ _ _**

**__ _ _ _**

**__ _ _ _**

**__ _ _ _**

**__ _ _ _**

**__ _ _ __**

**__ _ _ __**

**__ _ _ __**

**__ _ _ __**

**__ _ _ ___**

**__ _ _ ___**

**__ _ _ ___**

**__ _ _ ___**

**__ _ _ ___**

**__ _ _ _ ___**

**__ _ _ _ ___**

**__ _ _ _ ___**

**__ _ _ _ ___**

**__ _ _ _ ___**

**____ _ _ ___**

**____ _ _ ___**

**____ _ _ ___**

**____ _ _ ___**

**____ _ _ ___**

**____ _ _ ___**

**____ _ _ ___**

**____ _ _ ___**

**____ _ _ ___ _**

**____ _ _ ___ __**

**____ _ _ ___ __**

**____ _ _ ___ __**

**____ __ _ ___ __**

**____ __ _ ___ __ _**

**____ __ __ _ ___ __ _**

**____ __ __ _ ___ __ _**

**____ __ __ _ ___ __ _**

**____ __ __ __ ___ __ _**

**____ __ __ __ ___ __ _**

**____ _ __ __ __ ___ __ _**

**____ __ __ __ __ ___ __ _**

**____ __ _ __ __ __ ___ __ _**

**____ _ ___ _ _ __ __ __ ___ __ _**

**____ _ ___ _ _ __ _ __ __ ___ __ _**

**____ _ ___ _ _ __ _ __ __ ___ __ _**

**EEP** ●●●● ● ●●● ● ● ●● ● ●● ●● ●●● ●● ●

**2xswA_human**  476 **GDFNFRLSGGRTVVDALLCQGLVv...DVPALLQHDQLIREMRKGSIFKGFQEPDIHFLPSYKFDIGKDTYDSTSKQRTPSYTDRVLYRSRHKGDICPVSYSSCPGIKTSDHRPVYGLF** 591*

**XP_006278611.1**  460 **GDFNFRLNQDREAVNLILNQNLEm...DMSKLLQYDQLLKEMKNGSVFKGFQEAAIHFHPSYKFDIGQDCYDTTSKQRTPSYTDRVVYRSRHKNDIHAVKYSSCPIIRTSDHRPVYGLF** 575

**AAI50239.1**  234 **GDFNFRLNKARGDVEAILNQGVGv...DMSPLLQHDQLTREMKEGSIFKGFQEASIHFPPTYKFDIGCDVYDTTTKQRTPSYTDRILYRNRQSDDIRVIKYTSCSSIKTSDHRPVIGMF** 349

**XP_013801144.1**  491 **GDFNFRLNKDRETVDSILKQNLEk...DMSKLLEYDQLISEMSRGSIFKGFQEADIHFCPSYKFDIGKDSYDTTSKQRTPSYTDRVVYRSRHKDDIHAVKYSSCPVIKTSDHRPVFALF** 606

**XP_004916697.1**  438 **GDFNFRLNKERSAVNSILQSKLEk...DMSRLLQYDQLTKEMNGESIFKGFQEAEIQFLPTYKFDIGCDVYDSTSKQRTPSYTDRVVYKSRHKDDIRVVKYASCSLIKTSDHRPVFGLF** 553

**XP_011670872.1**  764 **GDFNFRLAENKARIEGWIDQLRRgnkdDYDILLQYDQLNKAMQRNEIFQGFHEGKIEFLPTYKFDIGEDIYDTSAKARVPSYTDRVLYKSRKVSDISLIHYSSCEEIKTSDHRPVYALF** 882

**foreground (45175):**  **GDFNAAHDSDAYENPDLVSAGNW DVSALLANDQLTRAAFRRLL AGFTDAWRAGGYSWKADRGSSNYDSGSKKGPGSRIDRVFASGGLADKVTVVSAGVDDD RGSDHAPVVATF** i

**L IRP EPDLKD R LKKREL MEK KF E L EML L L LVELF PPPTFPYY NTDEF TYE RR PL L YILVRPD LLRLRLLDVRILPE P L LLLEL**

**V D I K IEQKDF DE EY R L VY FAF YWD ND S N H YTKS K IK YE L F I VDI**

**wt_res_freqs (10915): 99591111211111114211111 73118511989131111132 1421421211122211125311562117111214491121311221121211212111 118881641312**

**2 113 112111 1 211111 311 11 1 1 111 1 1 31111 11161111 13311 313 11 11 2 2442111 11121111112111 1 1 21113**

**1 1 1 1 311121 11 31 1 1 11 111 211 11 5 1 2 1111 1 22 11 1 1 1 111**

**insertions**

**deletions 1222233234499999999 99999999999944443234343223356434322333399999999993433221111224334431994444444433232222222221**

**position**  480 . 490 . 500 . 510 . 520 . 530 . 540 . 550 . 560 . 570 . 580 . 590

**_**

**_**

**_**

**_ _**

**_ _**

**_ _**

**_ _**

**__ _**

**__ _ _ _**

**__ _ _ _ _**

**__ _ _ _ _**

**__ _ _ _ _**

**__ __ _ _ _ _**

**__ __ _ _ _ _ _ _ _**

**__ __ _ _ _ _ _ _ _**

**__ __ _ _ _ _ _ _ _ _**

**__ __ _ _ _ _ _ _ _ _**

**__ __ _ _ _ _ _ _ _ _**

**__ __ _ _ _ _ _ _ _ _**

**__ __ _ _ _ _ _ _ _ _**

**__ __ _ _ _ _ _ _ _ _**

**__ __ _ _ _ _ _ _ _ _ _**

**__ ___ _ _ _ _ _ _ _ _ _**

**__ ___ _ _ _ _ _ _ _ _ _**

**__ ___ _ _ _ _ _ _ _ _ _**

**__ ___ _ _ _ _ _ _ _ _ _ _**

**__ ___ _ _ _ _ _ _ _ _ _ _**

**__ ___ _ _ _ _ __ _ _ _ _ _**

**___ ___ _ _ _ _ _ __ _ _ _ _ _**

**___ ___ _ _ _ _ _ __ _ _ _ _ _**

**___ ___ _ _ _ _ _ __ _ _ _ _ _**

**___ ___ _ _ _ _ _ __ _ _ _ _ _**

**___ ___ _ _ _ _ _ __ _ ___ _ _**

**___ _ ___ _ _ _ _ _ __ _ ___ _ _**

**___ _ ___ _ _ _ _ _ __ _ ___ _ _**

**___ _ ___ _ _ _ _ _ __ _ ___ _ _**

**___ _ ___ _ _ _ _ _ __ _ ___ _ _**

**___ _ ___ _ _ _ _ _ __ _ ___ _ _**

**___ _ ___ _ _ _ _ _ __ _ ___ _ _**

**___ _ ___ _ _ _ _ _ ___ _ ___ _ _**

**___ _ ___ _ _ _ _ _ ___ _ ___ _ _**

**___ _ ___ _ _ _ _ _ ___ _ _ ___ _ _**

**___ _ ___ _ _ _ _ _ ___ _ _ ___ _ _ _**

**___ _ ___ _ _ _ _ _ ___ _ _ ___ _ _ _**

**___ _ ___ _ _ _ _ _ ___ _ _ ___ _ _ _ _**

**___ _ ___ _ _ _ _ _ ___ _ _ ___ _ _ _ _**

**___ _ ___ _ _ _ _ _ ___ _ _ _ ___ _ _ _ _**

**___ _ ___ _ _ _ _ _ ___ __ _ _ ___ _ _ _ _**

**___ _ ___ _ _ _ _ _ ___ __ _ _ ___ _ _ _ _**

**___ _ ___ _ _ _ _ _ ___ __ _ _ ___ _ _ _ _**

**___ _ ___ _ _ _ _ _ ___ __ _ _ ___ _ _ _ _**

**INPP5 family**  ●●● ● ●●● ● ● ● ● ● ●●● ●● ● ● ●●● ● ● ● ●

**2xswA_human**  476 **GDFNFRLSGGRTVVDALLCQGLVv...DVPALLQHDQLIREMRKGSIFKGFQEPDIHFLPSYKFDIGKDTYDSTSKQRTPSYTDRVLYRSRHKGDICPVSYSSCPGIKTSDHRPVYGLF** 591*

**XP_006278611.1**  460 **GDFNFRLNQDREAVNLILNQNLEm...DMSKLLQYDQLLKEMKNGSVFKGFQEAAIHFHPSYKFDIGQDCYDTTSKQRTPSYTDRVVYRSRHKNDIHAVKYSSCPIIRTSDHRPVYGLF** 575

**AAI50239.1**  234 **GDFNFRLNKARGDVEAILNQGVGv...DMSPLLQHDQLTREMKEGSIFKGFQEASIHFPPTYKFDIGCDVYDTTTKQRTPSYTDRILYRNRQSDDIRVIKYTSCSSIKTSDHRPVIGMF** 349

**XP_013801144.1**  491 **GDFNFRLNKDRETVDSILKQNLEk...DMSKLLEYDQLISEMSRGSIFKGFQEADIHFCPSYKFDIGKDSYDTTSKQRTPSYTDRVVYRSRHKDDIHAVKYSSCPVIKTSDHRPVFALF** 606

**XP_004916697.1**  438 **GDFNFRLNKERSAVNSILQSKLEk...DMSRLLQYDQLTKEMNGESIFKGFQEAEIQFLPTYKFDIGCDVYDSTSKQRTPSYTDRVVYKSRHKDDIRVVKYASCSLIKTSDHRPVFGLF** 553

**XP_011670872.1**  764 **GDFNFRLAENKARIEGWIDQLRRgnkdDYDILLQYDQLNKAMQRNEIFQGFHEGKIEFLPTYKFDIGEDIYDTSAKARVPSYTDRVLYKSRKVSDISLIHYSSCEEIKTSDHRPVYALF** 882

**foreground (3585):**  **GDFNYRVSGSGDDAKDLVSNGNW DVSSLLANDQLTQAKEAGKAFQGWSEGEITFAPTYKYDRGSSNYDSGSKQRAPAWCDRVLWKGKGDGDVTQLSYGACESYTTSDHKPVSGTF**

**L F LELPYEHIRR LKRREL MEK KY E LLQMRKNPILH FR APLK P FEPNTDEF TSE R I SYT I FRSRP D LRL RRHPLLRI R FSLL**

**IDDDR EV K IEKKDF DE EH RIERKS RV R YQ N L L ND K V Y R IK DSAMEIKF YAI**

**wt_res_freqs (885): 99296911111211114211111 73118511989111112512715119416392999855153115621181919685992843311139213329111111129994992117**

**7 2 3132121131 211111 311 11 1 11221111111 51 2311 3 31113311 454 1 1 314 7 14111 4 211 12221111 4 1111**

**43111 24 1 311121 11 31 113221 13 1 11 1 1 1 11 3 1 3 1 22 14122111 151**

**insertions 2**

**deletions 1111111 11111111111 99111111 1111111 111 1 11442111113223344444432222299322333327111288911111998**

**background (41590):**  **GDFNAAHGSDAYGNPKCASAGNN TALKEYDQLERAAFRRLLAAGFTDAWRAGGYSYWAYRNSSEYDSYMKGGPGSRIDRVFYSGGLADKSSVKSAGVDDD RGSDHAAVVATF**

**L ITPDE DLKD LLLKKKMI KW LKF E L ELL L EL LV LF P FTFPD DIDQPKQ NRD L L YILLRPD LPRIKLVDVRILPE P LPLLLEL**

**V D I RTIEETEF QK R K D VY W S VN GTD QKN W H VTKS KL E SE R L F IIVDI**

**wt_res_freqs (10030): 99591111211111112111121 22814395813111113211411521211122111121122111772111214491121411321111211213111 118881141212**

**2 11311 2111 32132211 12 424 1 2 111 1 11 31 11 1 15111 6112212 311 1 3 3431111 11113111112111 1 1621123**

**1 1 1 11111111 11 1 2 1 11 3 1 11 311 111 1 2 2111 11 2 11 1 1 1 11211**

**position**  480 . 490 . 500 . 510 . 520 . 530 . 540 . 550 . 560 . 570 . 580 . 590

**_**

**_**

**_**

**_**

**_**

**_**

**__ _ _**

**__ _ _ _**

**__ _ _ _**

**INPP5e subfamily**  ●● ● ● ●

**2xswA_human**  476 **GDFNFRLSGGRTVVDALLCQGLVv...DVPALLQHDQLIREMRKGSIFKGFQEPDIHFLPSYKFDIGKDTYDSTSKQRTPSYTDRVLYRSRHKGDICPVSYSSCPGIKTSDHRPVYGLF** 591*

**XP_006278611.1**  460 **GDFNFRLNQDREAVNLILNQNLEm...DMSKLLQYDQLLKEMKNGSVFKGFQEAAIHFHPSYKFDIGQDCYDTTSKQRTPSYTDRVVYRSRHKNDIHAVKYSSCPIIRTSDHRPVYGLF** 575

**AAI50239.1**  234 **GDFNFRLNKARGDVEAILNQGVGv...DMSPLLQHDQLTREMKEGSIFKGFQEASIHFPPTYKFDIGCDVYDTTTKQRTPSYTDRILYRNRQSDDIRVIKYTSCSSIKTSDHRPVIGMF** 349

**XP_013801144.1**  491 **GDFNFRLNKDRETVDSILKQNLEk...DMSKLLEYDQLISEMSRGSIFKGFQEADIHFCPSYKFDIGKDSYDTTSKQRTPSYTDRVVYRSRHKDDIHAVKYSSCPVIKTSDHRPVFALF** 606

**XP_004916697.1**  438 **GDFNFRLNKERSAVNSILQSKLEk...DMSRLLQYDQLTKEMNGESIFKGFQEAEIQFLPTYKFDIGCDVYDSTSKQRTPSYTDRVVYKSRHKDDIRVVKYASCSLIKTSDHRPVFGLF** 553

**XP_011670872.1**  764 **GDFNFRLAENKARIEGWIDQLRRgnkdDYDILLQYDQLNKAMQRNEIFQGFHEGKIEFLPTYKFDIGEDIYDTSAKARVPSYTDRVLYKSRKVSDISLIHYSSCEEIKTSDHRPVYALF** 882

**foreground (105):**  **GDFNFRLSGGRAAVDAILNCGEG DVSALLEHDQLSQEMKDGSVFKGFQEADIHFRPSYKFDVGCGSYASTSKQRTPSYTDRVVYKSRQAGDICAVKYSSCSGIKTSDHRPVYGVF**

**NKD VDIESMMRQDPE MPP QY LR RR I R PP L T L KDI DT IMFRN HKD HPL T PV R IAL**

**E L K NL V IK K I T L N QV S F**

**wt_res_freqs (24): 99999995459248264611114 73539836999319952981989989559791979999283128138899999999997271892139832679689258699999996719**

**332 211611215213 311 41 14 12 8 1 42 6 2 1 472 75 21281 654 141 1 51 3 217**

**1 1 4 12 2 13 3 6 3 5 1 22 1 1**

**insertions 9 51**

**deletions 44333332244211333322222 33344444444443221 1121 444444333444433332111113444444444333**

**background (3480):**  **GDFNYRVSGSGDDAKDLVSNGNW SSLLANDELTQAKEAGKAFQGWSEGEITFAPTYKYDRGSSNYDSGEKQRAPAWCDRVLWKGKGDIQVTQLSYGAAESYTTSDHKPVSGTF**

**L F LELPYEHIRR LKRREL EK KF Q LLQMRKNPILH FR APLK P RFEPNTDEF TS R I S T I FRSRP LRL RRHPLLRI R FSLL**

**IDDTR EV K IEKKDF DE EY RIERKS RV R YQ N L L ND K V Y R IK DS MEIKV YAI**

**wt_res_freqs (861): 99197911111211114211111 118511919111112512715119516392999865153115621471919695992843321188213329111111129994992117**

**7 2 3132121131 211111 11 11 8 11221111111 51 2311 3 131113311 35 1 1 3 4 7 13111 211 12221111 4 1111**

**43111 24 1 311121 11 31 113221 13 1 11 1 1 1 11 3 1 3 1 22 14 22111 151**

**position**  480 . 490 . 500 . 510 . 520 . 530 . 540 . 550 . 560 . 570 . 580 . 590

**Figure 5— Source data 1. EEP superfamily, INPP5 family and SHIP2 subfamily.**

**_________ ____ _______ _______________ __________ ___ ___ ________________________________________________________________________ _______ ____ ______ ___ __**

**Mammal**  428 **IGTWNMGSV--PPPKNVTSWFTSkglgktldevtvTIPHDIYVFGTQENSVGDREWLDLLRGGLKELTDLDYRPIAMQSLWNIKVAVLVKPEHENRISHVSTSSVKTGIANTLGNKGAVGVSFMFNGTSFGFVNCHLTSGNEKTARRNQNYLDILRLLSLGDRqlnaFDISLR** 598*

**Reptile**  390 **IGTWNMGSV--FPPKSVTSWFTSkglgktldeatvTIPHDIYVFGTQENSMGDKEWVDFLRGALKDFTDIEYRPIAMQSLWYIKIVVLVKPEHENRISHISTSSVKTGIANTLGNKGAVGVSFMFNGTSFGFVNCHLTSGNEKTARRNQNYVDILRLLSLGDKqlssFDISLR** 560

**Fish**  412 **IGTWNMGSV--PAPKPLGSWILSrglgktldemavTIPHDIYVFGTQENSVCDKEWVETLRCSLKEYTDMEYKPIAVQTLWNIKIVVLVKAEHENRISHVGMSSVKTGIANTLGNKGAVGVSFMFNGTSFGFVNCHLTSGNEKIHRRNQNYLDILRQLSLGDKqlnsFDISLR** 582

**Bird**  406 **IGTWNMGAA--PPPKKITSWFLSkgqgktrddtadYIPHDIYVIGTQEDPQGEKEWLETLRQSLQEITSISFKVIAIHTLWNIRIVVLAKPEHENRISHICTDNVKTGIANTLGNKGAVGVSFMFNGTSFGFVNSHLTSGSEKKHRRNQNYMNILRFLTLGDKklspFNITHR** 576

**Amphibian**  294 **VGTWNMGGS--PPPRSISSWLSSrglgrsledtgpCVSHDLYMVGTQENPQGDREWAEFLRLALISHTGKQFKVVSMHSLGGVKLVLLVKQEYESLISHVQISSVRTGMSNTLGHRGAVGASLDFCGISLGFVTCHLVSGNEKVQKRNQSYGEILRGLTLGDEslkcFQLPLR** 464

**position** 430 . 440 . 450 . 460 . 470 . 480 . 490 . 500 . 510 . 520 . 530 . 540 . 550 . 560 . 570 . 580 . 590 .

**_**

**_ _ _**

**_ _ _**

**_ _ _**

**_ _ _**

**_ _ _**

**_ _ _**

**_ _ _**

**_ _ _**

**_ _ _**

**_ _ _**

**_ _ _**

**_ _ _**

**_ _ _**

**_ _ _**

**_ _ _ _**

**_ _ _ _**

**_ _ _ _**

**_ _ _ _**

**_ _ _ _**

**_ _ _ __ _**

**_ _ _ __ _**

**_ _ _ __ _**

**_ _ __ __ _**

**_ _ __ __ _**

**_ _ _ __ __ _**

**_ ___ __ __ _**

**_ ___ __ ___ _**

**_ ___ __ ___ _ _**

**_ ___ __ ___ _ _**

**_ ___ __ _ ___ _ _ _**

**_ ___ __ _ ___ _ _ _**

**_ ____ __ _ ___ _ _ _**

**_ ____ __ _ ___ _ _ _**

**_ ____ __ _ ___ _ _ _**

**_ ____ __ _ ___ _ _ _**

**_ ____ __ _ ___ _ _ _ _ _**

**_ ____ __ _ ___ _ _ _ _ _**

**_ ____ __ _ ___ _ _ _ _ _**

**_ ____ _ __ _ ___ _ _ _ _ _**

**_ ____ _ __ _ ___ __ _ _ _ _**

**______ _ _ __ _ ___ __ _ _ _ _**

**______ _ _ __ _ ___ __ _ _ _ _**

**______ _ _ __ _ ___ _ __ _ _ _ _ _**

**______ _ _ __ _ ___ _ __ _ _ _ _ _**

**______ _ _ __ _ ___ _ __ _ _ _ _ _**

**______ _ _ ___ _ ___ _ __ _ _ _ _ _ _ _**

**______ _ _ ___ _ ___ _ __ _ _ _ _ _ _ _**

**EEP** ●●●●●● ● ● ●●● ● ●●● ● ●● ● ● ● ● ● ● ●

**3nr8B_Ship2_hu**  428 **IGTWNMGSV--PPPKNVTSWFTSkglgktldevtvTIPHDIYVFGTQENSVGDREWLDLLRGGLKELTDLDYRPIAMQSLWNIKVAVLVKPEHENRISHVSTSSVKTGIANTLGNKGAVGVSFMFNGTSFGFVNCHLTSGNEKTARRNQNYLDILRLLSLGDRqlnaFDISLR** 598*

**XP_006038756.1**  390 **IGTWNMGSV--FPPKSVTSWFTSkglgktldeatvTIPHDIYVFGTQENSMGDKEWVDFLRGALKDFTDIEYRPIAMQSLWYIKIVVLVKPEHENRISHISTSSVKTGIANTLGNKGAVGVSFMFNGTSFGFVNCHLTSGNEKTARRNQNYVDILRLLSLGDKqlssFDISLR** 560

**SHP2A_DANRE**  412 **IGTWNMGSV--PAPKPLGSWILSrglgktldemavTIPHDIYVFGTQENSVCDKEWVETLRCSLKEYTDMEYKPIAVQTLWNIKIVVLVKAEHENRISHVGMSSVKTGIANTLGNKGAVGVSFMFNGTSFGFVNCHLTSGNEKIHRRNQNYLDILRQLSLGDKqlnsFDISLR** 582

**KFM10957.1**  406 **IGTWNMGAA--PPPKKITSWFLSkgqgktrddtadYIPHDIYVIGTQEDPQGEKEWLETLRQSLQEITSISFKVIAIHTLWNIRIVVLAKPEHENRISHICTDNVKTGIANTLGNKGAVGVSFMFNGTSFGFVNSHLTSGSEKKHRRNQNYMNILRFLTLGDKklspFNITHR** 576

**XP_002934956.3**  294 **VGTWNMGGS--PPPRSISSWLSSrglgrsledtgpCVSHDLYMVGTQENPQGDREWAEFLRLALISHTGKQFKVVSMHSLGGVKLVLLVKQEYESLISHVQISSVRTGMSNTLGHRGAVGASLDFCGISLGFVTCHLVSGNEKVQKRNQSYGEILRGLTLGDEslkcFQLPLR** 464

**foreground (45237):**  **VASWNVNGFNAAAADGVAAWVAS AENADVYAVCVQETKDSDGAFLAGASESALAAALGGY LYYSGA GGVGVFSKKKKSHAPRVSNFEVLSV TGIGGADGSGGAVAATFEVGGTTFTFASAHFPAGGGKLAAALAQAAAFAARVAALAA GSSTA**

**LMTF LLSLR LKALLRLLRP RLDP LLVLAL L DQAQLPLDLVPEL LLL P VFP R LALLVRYPLKLL FLREVLP E LPS L ERRVLVLRLRLP RPLVLINLYL N SSR EYR RFLELLLELLKK L PRTL**

**IL Y IR I KE I E IKL E II IG I ST KTE E L Q I Y RE I HIKD IL D L NK I VEIKIK K IRIV V S DEK EWRDEI D I E D I**

**wt_res_freqs (11217): 42339423111111111111111 1112834332179111111111111111111111134 111121 1631113211112121221111112 121111211122113121114112111116131211111111121111111111211 11111**

**1141 11122 1113111312 1123 114216 1 1111112111111 111 1 111 1 142413121131 1121111 1 111 1 132121112111 1121213124 1 112 113 111113211311 1 1111**

**32 2 21 1 11 2 1 211 1 41 22 1 11 111 1 1 1 3 1 11 1 1412 11 1 1 11 1 211111 1 1112 2 2 112 111111 1 1 1 1 4**

**insertions 1 5 6 2 6**

**deletions 21111111111427775555449 111121991111135467776139999963989655222233334322111111149999999953122222333133143222222223333211 1 135112115555442222233345526 999937**

**position** 430 . 440 . 450 . 460 . 470 . 480 . 490 . 500 . 510 . 520 . 530 . 540 . 550 . 560 . 570 . 580 . 590 .

**_**

**_ _**

**_ _**

**_ _**

**_ _**

**_ _**

**_ _**

**_ _**

**_ _**

**_ _ _**

**_ _ _**

**_ _ _**

**_ __ _**

**_ __ _**

**_ __ _**

**_ __ _**

**_ __ _**

**_ __ _**

**_ __ _ _**

**_ __ _ _**

**__ __ _ _**

**__ ___ _ _**

**__ ___ _ _**

**__ ____ _ _**

**_ __ ____ _ _ _**

**_ __ ____ _ _ _**

**_ __ ____ _ _ _**

**_ __ ____ _ _ _**

**_ __ ____ _ _ _ _**

**_ ___ _ ____ _ _ _ _**

**_ ___ _ ____ _ _ _ _**

**_ ___ _ ____ _ _ _ _ _ _**

**_ ___ _ ____ _ _ _ _ _ _ _**

**_ ___ _ ____ _ _ _ _ _ _ _**

**_ _ ___ _ _____ _ _ _ _ _ _ _**

**_ _ _ ___ _ _____ _ _ _ _ __ _ _**

**_ _ _ ___ _ _ _____ _ _ _ _ __ _ _**

**_ _ _ ___ _ _ _____ _ _ _ _ __ _ _**

**INPP5 family**  ● ● ● ●●● ● ● ●●●●● ● ● ● ● ●● ● ●

**3nr8B_Ship2_hu**  428 **IGTWNMGSV--PPPKNVTSWFTSkglgktldevtvTIPHDIYVFGTQENSVGDREWLDLLRGGLKELTDLDYRPIAMQSLWNIKVAVLVKPEHENRISHVSTSSVKTGIANTLGNKGAVGVSFMFNGTSFGFVNCHLTSGNEKTARRNQNYLDILRLLSLGDRqlnaFDISLR** 598*

**XP_006038756.1**  390 **IGTWNMGSV--FPPKSVTSWFTSkglgktldeatvTIPHDIYVFGTQENSMGDKEWVDFLRGALKDFTDIEYRPIAMQSLWYIKIVVLVKPEHENRISHISTSSVKTGIANTLGNKGAVGVSFMFNGTSFGFVNCHLTSGNEKTARRNQNYVDILRLLSLGDKqlssFDISLR** 560

**SHP2A_DANRE**  412 **IGTWNMGSV--PAPKPLGSWILSrglgktldemavTIPHDIYVFGTQENSVCDKEWVETLRCSLKEYTDMEYKPIAVQTLWNIKIVVLVKAEHENRISHVGMSSVKTGIANTLGNKGAVGVSFMFNGTSFGFVNCHLTSGNEKIHRRNQNYLDILRQLSLGDKqlnsFDISLR** 582

**KFM10957.1**  406 **IGTWNMGAA--PPPKKITSWFLSkgqgktrddtadYIPHDIYVIGTQEDPQGEKEWLETLRQSLQEITSISFKVIAIHTLWNIRIVVLAKPEHENRISHICTDNVKTGIANTLGNKGAVGVSFMFNGTSFGFVNSHLTSGSEKKHRRNQNYMNILRFLTLGDKklspFNITHR** 576

**XP_002934956.3**  294 **VGTWNMGGS--PPPRSISSWLSSrglgrsledtgpCVSHDLYMVGTQENPQGDREWAEFLRLALISHTGKQFKVVSMHSLGGVKLVLLVKQEYESLISHVQISSVRTGMSNTLGHRGAVGASLDFCGISLGFVTCHLVSGNEKVQKRNQSYGEILRGLTLGDEslkcFQLPLR** 464

**foreground (3807):**  **VGSWNVGGATEGSDDSIASWFGS SDDPDFYAFGFQEVDSGSAGNVLGASESDVEASGEGYKKVASETLGGVAICVWASKSHAPRVSNVAAASTGCGFGGKWGNKGGVGTSFSYGGSSFCFVCCHFAAGASAVDERNANYATIVSGASFSKG GSSTLD**

**LVTF MNSKFPPPPEDLTPLLLP EEP LLVL L LVPLEYKLLMDLVPELPWLLPPKFVLLRTVRML LLLLLFIRPKLL FIRELEVDTVKT PM MMS AISIRMVLFDTTLTLINS LTSHQEKLER QDFKK LRRMLLPRN PRTL R**

**I Y AAQKRS E SE FL D II I I LKN EFI ST KTF KT D D I KQ V IF FIYVKRE I H KDISTS M YL AV LRIYN I TA DE N Q E VSE KKLR D I E**

**wt_res_freqs (973): 44169534121111111115111 11157143174993111111111111111111112171131411517111151211121212214112112181361178993621231113143576139156412121295114118111116111 111124**

**1181 131121142236112612 112 2142 3 231311112111111111131112333111121 325215131131 142112111525 11 111 411361131251211252 8113121133 16211 222111211 1111 1**

**2 1 111311 1 11 11 1 41 3 2 111 112 11 111 11 2 1 1 13 4 21 1214311 1 1 121122 1 11 33 21111 1 12 11 2 1 1 111 1131 1 4 1**

**insertions 2 7 2 11 2 6 1 2 1 21 7 2 1 12 1 2 61 1 11 52 2 1 7 3**

**deletions 22111111175111111111111 1111999999888887765432133441119 11111111222111111111231111111112223229222111111111 11111221111 11 1 3 243311**

**background (41430):**  **VASWNVNGFNAAAADGVAAEVAT AENADVYAVCVQETKDSDGADLAGSNSSAEAAAAGGYHAYYSGAKGGVGFFSKKEKSKWVTVSNFIVLSVEVGLGGAD SGGAVAATFEVGGKTFTVASAHFPAGGGKLAARLAQAAAIAARVAALAA SDGIAA**

**LMTF LLNLR LKLLLRLLRL RLDP LIILLL IR DQDQLPLDLLFKLLLLELP YVFPYR LALLYRYPL LLPNLREVLP E PS L ERRPLVLRLRLP RPLVLINLYL N RSR EYA RFLELLLELLKK LK KLKLPK**

**IL Y IRSI KERI EWIKV E IVVIA V N ADF NEVEEE L II R I IKD L D L VI VEIKIK IRIV V S S DEK EWRDEQ DYI E I E N**

**wt_res_freqs (10244): 42439422111111111111111 111284213317912111111111211111111113311111211163111331112111112121111111111112 1111113121114111141116131211111131121111111111211 111211**

**1141 11122 1112111311 1123 141216 11 1111112111111111111 111111 151414131 1221211111 1 11 1 132131112111 1121213134 1 113 111 111113111311 11 112111**

**32 2 3111 1112 11211 1 41522 3 1 111 111111 1 31 1 1 421 1 1 1 12 211111 1112 2 2 1 112 111111 111 1 1 2 1**

**position** 430 . 440 . 450 . 460 . 470 . 480 . 490 . 500 . 510 . 520 . 530 . 540 . 550 . 560 . 570 . 580 . 590 .

**_**

**_**

**_**

**_**

**_**

**_**

**_**

**_**

**_**

**_**

**_**

**_**

**_**

**_**

**_**

**_**

**_**

**_**

**_**

**_**

**_**

**_**

**_**

**_**

**_**

**_**

**_**

**_**

**_**

**_**

**_**

**_**

**_**

**_**

**_ _**

**_ _ _**

**_ _ _ _**

**_ _ _ _**

**_ _ _ _ _**

**_ _ _ _ _**

**_ _ _ _ _ _**

**_ _ _ _ _ _**

**_ _ _ _ _ _ _**

**_ _ _ _ _ _ _**

**_ _ _ _ _ _ _**

**_ _ _ _ _ _ _ _ _ _ _ _**

**_ _ _ _ _ _ _ _ _ _ _ _ _**

**_ _ _ _ _ _ _ __ _ _ _ __ _ _ _ __ _**

**_ _ _ _ __ _ _ _ _ _ _ _ __ _ _ _ _ _ __ _ _ _ _ ____ _ _**

**_ _ _ _ __ _ _ _ _ _ _ _ __ _ _ _ _ _ __ _ _ _ _ _ ____ _ _ _**

**_ _ _ _ __ _ _ _ _ _ _ _ __ _ _ _ _ _ __ _ _ _ _ _ ____ _ _ _**

**SHIP2 subfamily**  ● ● ● ● ●● ● ● ● ● ● ● ● ●● ● ● ● ● ● ●● ● ● ● ● ● ●●●● ● ● ●

**3nr8B_Ship2_hu**  428 **IGTWNMGSV--PPPKNVTSWFTSkglgktldevtvTIPHDIYVFGTQENSVGDREWLDLLRGGLKELTDLDYRPIAMQSLWNIKVAVLVKPEHENRISHVSTSSVKTGIANTLGNKGAVGVSFMFNGTSFGFVNCHLTSGNEKTARRNQNYLDILRLLSLGDRqlnaFDISLR** 598*

**XP_006038756.1**  390 **IGTWNMGSV--FPPKSVTSWFTSkglgktldeatvTIPHDIYVFGTQENSMGDKEWVDFLRGALKDFTDIEYRPIAMQSLWYIKIVVLVKPEHENRISHISTSSVKTGIANTLGNKGAVGVSFMFNGTSFGFVNCHLTSGNEKTARRNQNYVDILRLLSLGDKqlssFDISLR** 560

**SHP2A_DANRE**  412 **IGTWNMGSV--PAPKPLGSWILSrglgktldemavTIPHDIYVFGTQENSVCDKEWVETLRCSLKEYTDMEYKPIAVQTLWNIKIVVLVKAEHENRISHVGMSSVKTGIANTLGNKGAVGVSFMFNGTSFGFVNCHLTSGNEKIHRRNQNYLDILRQLSLGDKqlnsFDISLR** 582

**KFM10957.1**  406 **IGTWNMGAA--PPPKKITSWFLSkgqgktrddtadYIPHDIYVIGTQEDPQGEKEWLETLRQSLQEITSISFKVIAIHTLWNIRIVVLAKPEHENRISHICTDNVKTGIANTLGNKGAVGVSFMFNGTSFGFVNSHLTSGSEKKHRRNQNYMNILRFLTLGDKklspFNITHR** 576

**XP_002934956.3**  294 **VGTWNMGGS--PPPRSISSWLSSrglgrsledtgpCVSHDLYMVGTQENPQGDREWAEFLRLALISHTGKQFKVVSMHSLGGVKLVLLVKQEYESLISHVQISSVRTGMSNTLGHRGAVGASLDFCGISLGFVTCHLVSGNEKVQKRNQSYGEILRGLTLGDEslkcFQLPLR** 464

**foreground (120):**  **VGTWNMGGAAAPPPKSVASWFTC CIPHDIYAFGTQENSQGDKEWTDTVKGALQSYTAVSYKTVATQCLWNIKIAVFAKPEHESRISHVCSASVKTGIGNTLGNKGAVGVSFLFNGTSFGFVNCHLASGSEKTARRNQNYSSILRFLALGDK FNISHR**

**I NVPV RKLQ LLS LL VL DPL ER LEILRHV RELMEMEFRPI MHT SMRLV LV Y RL IFTDN LA M L S T N IL FMD L N R DLPL**

**STS NIT VQ Y I V V FI AT KDI DLD L L S N N S VH LN S S T**

**wt_res_freqs (38): 49999991446799431299533 18999891299996428449913113129111811246159161997845391399988188986211799997198999999998992999998999969919968931999998119993919998 816339**

**5 2222 3131 235 11 61 355 54 2616511 33211115223 124 11525 85 1 11 21621 18 6 1 3 8 2 14 115 4 1 1 6115**

**412 243 11 2 5 1 3 21 31 413 114 1 1 3 5 2 4 12 21 1 4 4**

**insertions 9 1 9 1**

**deletions 655555555991 1112333333333333222222222222211111113 122222211111111111222333322 1111**

**background (3687):**  **VGSWNMGGATEGSDDSIASWLGS SDAPDFYAFGFQEVDSSAAGNVLGAAESDVEASGEGYVKVASETLGGVAICVWASKSHAPRVSNVAAASTGCGFGGKWGNKGGVGTSFSYGGSSFCFVCCHFAAGASAVDERNADYATIVSGASLSKG GSSILS**

**LVTF VNSKFPSPPQDLSPL LP EEP LLVL L LVPLNYKLLMDTVPEFPWLLPPKF LLRTVRML LLLLLFIRRKLL FIRELQVDTVKT PM MMSR AISIRMVLFETTLLLINS LTSHQEKLER Q VKK LRRMLFPPN PRTL E**

**I Y AAQKRA EE E FL D D II I I LKTEEFI SSDKTK KT D D I KQ V IF FIYVK E I H KDIEKS M YL AV LRIYD IT TA DE E Q E FSE KKLR R D D**

**wt_res_freqs (935): 44169134121111111115711 11157133174993111111111111111111112173131411417111141211111212214112112181371178993621131113143576139156412121295164117111111111 111421**

**1181 531121142146112 12 111 2142 3 231311112211111111131111 33111221 325215131131 142211111525 12 1111 411361131151211252 8113121132 1 111 222116211 1111 1**

**2 1 111311 12 1 11 1 1 41 3 2 1111112 111111 11 2 1 1 13 4 21 12143 1 1 1 121212 1 11 32 21212 11 12 11 1 1 1 211 1131 1 1 4**

**position** 430 . 440 . 450 . 460 . 470 . 480 . 490 . 500 . 510 . 520 . 530 . 540 . 550 . 560 . 570 . 580 . 590 .

**Mammal**  599 **FTHLFWFGDLNYRLDMDIQE...ILNYISRKEFEPLLRVDQLNLEREKHKVFLRFSEEEISFPPTYRYERGSRDTYAWHKQkptgvRTnv.....PSWCDRILWKSYPEthIICNSYGCTDDIVTSDHSPVFGTF** 725*

**Reptile**  561 **FTHLFWFGDLNYRLDMDIQE...ILNYINRKEFEPLLKVDQLNLEKEKHKVFLRFAEEEITFPPTYRYEKGSRDTYVWHKQkptgvRTnv.....PSWCDRILWKSYPEthINCNSYGCTDDVMTSDHSPVFGSF** 687

**Fish**  583 **FTHLFWFGDLNYRLDMDIQE...ILNYINRKEFDPLLKVDQLNLEREKNKIFLRFAEEEISYPPTYRYERGSRDTYVWQKQkatgmRTnv.....PSWCDRILWKSYPEthIVCNSYGCTDDIVTSDHSPVFGTF** 709

**Bird**  577 **FTHLFWLGDLNYRVEQPPTEaenIIQKIRQQQYPELLAFDQLLIERKDQKVFLQFEEEEITFAPTYRFERGTREKYAYTKQkatgmKYnl.....PSWCDRVLWKSYPMvhVVCQSYGCTTDIMTSDHSPVFATF** 706

**Amphibian**  465 **LTHLFWAGDLNYRLSMPLQD...ILQCVYSGRYQVLLPVDQLSQERERKKIFLGFKEDSVTFPPTCRYERGTRSYDL-HKA.....RTtgtrlfaPCWSDRVLWTSYPDtdIKCTSYGCTEDIVTSDHSPVFATF** 590

**position**  . 610 . 620 . 630 . 640 . 650 . 660 . 670 . 680 . 690 . 700 . 710 . 720 .

**_**

**_**

**_ _**

**_ _**

**__ _**

**__ _ _ _**

**__ _ _ _**

**__ _ _ _**

**__ _ _ _**

**__ _ _ _**

**__ _ _ _**

**__ _ _ _**

**__ _ _ __**

**__ _ _ __**

**__ _ _ __**

**__ _ _ __**

**__ _ _ ___**

**__ _ _ ___**

**__ _ _ ___**

**__ _ _ ___**

**__ _ _ ___**

**__ _ _ ___**

**__ _ _ _ ___**

**__ _ _ _ ___**

**__ _ _ _ ___**

**__ _ _ _ ___**

**__ _ _ _ ___**

**____ _ _ ___**

**____ _ _ ___**

**_ ____ _ _ ___**

**_ ____ _ _ ___**

**_ ____ _ _ ___**

**_ ____ _ _ ___**

**_ ____ _ _ ___**

**_ ____ _ _ ___**

**_ ____ _ _ ___ __**

**__ ____ _ _ ___ __**

**__ ____ _ _ ___ __**

**__ ____ _ _ ___ __**

**__ ____ __ _ _ ___ __ _**

**__ ____ __ __ _ ___ __ _**

**__ ____ __ __ _ ___ __ _**

**__ ____ __ __ _ ___ __ _**

**__ ____ __ __ _ ___ __ _**

**__ ____ __ __ __ ___ __ _**

**__ ____ __ __ __ ___ __ _**

**__ ____ _ __ __ __ ___ __ _**

**__ ____ __ __ __ __ ___ __ _**

**__ ____ ___ _ __ __ __ ___ __ _**

**__ ____ _ ___ _ _ __ _ __ __ ___ __ _**

**__ ____ _ ___ _ _ __ _ __ __ ___ __ _**

**EEP**  ●● ●●●● ● ●●● ● ● ●● ● ●● ●● ●●● ●● ●

**3nr8B_Ship2_hu**  599 **FTHLFWFGDLNYRLDMDIQE...ILNYISRKEFEPLLRVDQLNLEREKHKVFLRFSEEEISFPPTYRYERGSRDTYAWHKQkptgvRTnv.....PSWCDRILWKSYPEthIICNSYGCTDDIVTSDHSPVFGTF** 725*

**XP_006038756.1**  561 **FTHLFWFGDLNYRLDMDIQE...ILNYINRKEFEPLLKVDQLNLEKEKHKVFLRFAEEEITFPPTYRYEKGSRDTYVWHKQkptgvRTnv.....PSWCDRILWKSYPEthINCNSYGCTDDVMTSDHSPVFGSF** 687

**SHP2A_DANRE**  583 **FTHLFWFGDLNYRLDMDIQE...ILNYINRKEFDPLLKVDQLNLEREKNKIFLRFAEEEISYPPTYRYERGSRDTYVWQKQkatgmRTnv.....PSWCDRILWKSYPEthIVCNSYGCTDDIVTSDHSPVFGTF** 709

**KFM10957.1**  577 **FTHLFWLGDLNYRVEQPPTEaenIIQKIRQQQYPELLAFDQLLIERKDQKVFLQFEEEEITFAPTYRFERGTREKYAYTKQkatgmKYnl.....PSWCDRVLWKSYPMvhVVCQSYGCTTDIMTSDHSPVFATF** 706

**XP_002934956.3**  465 **LTHLFWAGDLNYRLSMPLQD...ILQCVYSGRYQVLLPVDQLSQERERKKIFLGFKEDSVTFPPTCRYERGTRSYDL-HKA.....RTtgtrlfaPCWSDRVLWTSYPDtdIKCTSYGCTEDIVTSDHSPVFATF** 590

**foreground (45237):**  **GDPVVVCGDFNAAHDSDAYE NPDLVSAGNWSALLANDQLTRAAFRRLLEAGFTDAWRAGGYSWKSDRGSSNYDSGEKK GP GSRIDRVFASGGLA DKVVSAGVDDD RGSDHAPVVATF**

**DE LLLM L IRP EPDLK D R LKKKELEK KF E L EML L L LVELF PPPTFPDY DTDE TYM R R PL L YILVRPD L LRLLDVRILPE P L LLLEL**

**IIIL V D I K IEQNDFDE EY R L VY FAF YW N ND S N H YTKS K YE L F I VDI**

**wt_res_freqs (11217): 11332219959111121111 1113211111118511989131111132114214212111222111243115521371 11 21449112131122 11211212111 118881641312**

**11 1131 2 113 11211 1 1 21111111 11 1 1 111 1 1 31111 11161111 1331 311 1 1 11 2 2442111 1 11111112111 1 1 21113**

**1411 1 1 1 1 31112111 31 1 1 11 111 21 1 11 5 1 2 1111 1 11 1 1 1 111**

**insertions 2 2 1 2 5**

**deletions 5431 122223323 4499999999999999999944443234343223356434322333399999999993 43 32211112243344 254444444433232222222221**

**background (15610):**   **L L L L L LLL L L LL LL L LL L L LL L L LL LL LL LLL L L**

**wt_res_freqs (15469): 1 1 1 1 1 111 1 1 11 11 1 11 1 1 11 1 1 11 11 11 111 1 1**

**_**

**_**

**_**

**_ _**

**_ _**

**_ _**

**_ _**

**__ _ _**

**__ _ _ _**

**__ _ _ _**

**__ _ _ _ _**

**__ _ _ _ _**

**__ _ _ _ _ _ _**

**__ _ _ _ _ _ _ _**

**__ _ _ _ _ _ _ _**

**__ _ _ _ _ _ _ _**

**__ _ _ _ _ _ _ _**

**__ _ _ _ _ _ _ _**

**__ _ _ _ _ _ _ _**

**__ _ _ _ _ _ _ _ _ _**

**__ _ _ _ _ _ _ _ _ _**

**__ __ _ _ _ _ _ _ _ _**

**__ __ _ _ _ _ _ _ _ _ _**

**__ __ _ _ _ _ _ _ _ _ _ _**

**__ __ _ _ _ _ __ _ _ _ _ _**

**__ __ _ _ _ _ __ _ _ _ _ _**

**__ __ _ _ _ _ _ __ _ _ _ _ _**

**__ __ _ _ _ _ _ __ _ _ _ _ _**

**___ ___ _ _ _ _ _ __ _ _ _ _ _**

**___ ___ _ _ _ _ _ __ _ ___ _ _**

**___ _ ___ _ _ _ _ _ __ _ ___ _ _**

**___ _ ___ _ _ _ _ _ __ _ ___ _ _**

**___ _ ___ _ _ _ _ _ __ _ ___ _ _**

**___ _ ___ _ _ _ _ _ __ _ ___ _ _**

**___ _ ___ _ _ _ _ _ __ _ ___ _ _**

**___ _ ___ _ _ _ _ _ ___ _ ___ _ _**

**___ _ ___ _ _ _ _ _ ___ _ ___ _ _**

**___ _ ___ _ _ _ _ _ ___ _ ___ _ _ _**

**___ _ ___ _ _ _ _ _ ___ _ ___ _ _ _**

**___ _ ___ _ _ _ _ _ ___ _ ___ _ _ _**

**___ _ ___ _ _ _ _ _ ___ _ ___ _ _ _**

**_ ___ _ ___ _ _ _ _ _ ___ _ ___ _ _ _**

**_ ___ _ ___ _ _ _ _ _ ___ _ ___ _ _ _**

**_ ___ _ ___ _ _ _ _ _ ___ _ ___ _ _ _**

**_ ___ _ ___ _ _ _ _ _ ___ _ ___ _ _ _**

**_ ___ _ ___ _ _ _ _ _ ___ _ _ ___ _ _ _**

**_ ___ _ ___ _ _ _ _ _ ___ _ _ ___ _ _ _**

**_ _ ___ _ ___ _ _ _ _ _ ___ _ _ ___ _ _ _**

**_ _ ___ _ ___ _ _ _ _ _ ___ _ _ ____ _ _ _ _**

**_ _ ___ _ ___ _ _ _ _ _ ___ __ _ _ ____ _ _ _ _**

**_ _ ___ _ ___ _ _ _ _ _ ___ __ _ _ ____ _ _ _ _**

**INPP5 family** ● ● ●●● ● ●●● ● ● ● ● ● ●●● ●● ● ● ●●●● ● ● ● ●

**3nr8B_Ship2_hu**  599 **FTHLFWFGDLNYRLDMDIQE...ILNYISRKEFEPLLRVDQLNLEREKHKVFLRFSEEEISFPPTYRYERGSRDTYAWHKQkptgvRTnv.....PSWCDRILWKSYPEthIICNSYGCTDDIVTSDHSPVFGTF** 725*

**XP_006038756.1**  561 **FTHLFWFGDLNYRLDMDIQE...ILNYINRKEFEPLLKVDQLNLEKEKHKVFLRFAEEEITFPPTYRYEKGSRDTYVWHKQkptgvRTnv.....PSWCDRILWKSYPEthINCNSYGCTDDVMTSDHSPVFGSF** 687

**SHP2A_DANRE**  583 **FTHLFWFGDLNYRLDMDIQE...ILNYINRKEFDPLLKVDQLNLEREKNKIFLRFAEEEISYPPTYRYERGSRDTYVWQKQkatgmRTnv.....PSWCDRILWKSYPEthIVCNSYGCTDDIVTSDHSPVFGTF** 709

**KFM10957.1**  577 **FTHLFWLGDLNYRVEQPPTEaenIIQKIRQQQYPELLAFDQLLIERKDQKVFLQFEEEEITFAPTYRFERGTREKYAYTKQkatgmKYnl.....PSWCDRVLWKSYPMvhVVCQSYGCTTDIMTSDHSPVFATF** 706

**XP_002934956.3**  465 **LTHLFWAGDLNYRLSMPLQD...ILQCVYSGRYQVLLPVDQLSQERERKKIFLGFKEDSVTFPPTCRYERGTRSYDL-HKA.....RTtgtrlfaPCWSDRVLWTSYPDtdIKCTSYGCTEDIVTSDHSPVFATF** 590

**foreground (3807):**  **HDHVFWFGDFNYRISGSGDD AKDLVSAGNWSALLANDQLTQAKEAGKAFQGWSEGEISFAPSYKYDRGSSNYDSGEKT RA PAWCDRVLWKGSGD VSQVSYGSCGSYCTSDHKPVSGTF**

**FEVLILL L F LELPYEH IRR LKKRELEK KF E LLQMRKNPILH LR APLK P T FEPDTDEF TYM R I SYT I FRSRPK LRLL RRIPLLRM R FLL**

**YI F DDTR E V K IEQKDFDE EY RIERK RV R FQ N L VN ND S K V Y KR IK D HMEIKI YSI**

**wt_res_freqs (973): 56135639929694111121 1113211111118511989111211512715119417191919855143115521371 91 96859928322111 213129141111129994992117**

**1111212 6 3 3122121 131 21111111 11 1 11221111111 11 2311 3 8 31113311 311 1 1 314 7 131111 2113 12121111 4 111**

**12 1 3111 2 4 1 31112111 31 11322 13 1 51 1 1 21 11 5 3 1 3 11 22 1 222111 111**

**insertions 5 4 2 37 1 2 1 1 1 11 2 11 4 6 3 11 4**

**deletions 11111111111 1 1 1111111111111111 6665621 221 1 11332111113 22 33444444322222 222333327111288911111199**

**background (41430):**  **GAPVVVCGDFNAAHGSDAYG NPELVDAGNQDDLLAHDQLERAAFRRLLAAGFTDAWRAGGYSYWAYRGSNAYDSSSKG GP GSRIDRVFYSGGLA DKVKSAGVDDD RGSDHAAVVATF**

**DE LLLM L ITPDEIDLK D I LKKKKLQK KF E K ELL L EL LV LF P FTFPD DPEESNLLM R D L L YILLRPD L LRLVDVRILPE P LPLLLEL**

**IIIL V D I R IEQNDI E QY D VY W S NTDNN TYK K N W H VTKS SE R L F IIVDI**

**wt_res_freqs (10244): 11432219959111121111 1114111111117511978131111132114115212111221111221115412171 11 21449112131132 11211213111 118881141212**

**11 1131 2 113111211 1 1 21111113 21 1 3 112 1 11 31 11 1 15111 111212112 1 1 1 3 3431111 1 11111112111 1 1621123**

**1421 1 1 1 1 311131 1 11 1 11 3 1 33311 311 1 1 1 2 2111 11 1 1 1 11211**

**_**

**_ _ _**

**_ _ _**

**_ _ _**

**_ _ _**

**_ _ _ _**

**_ _ _ _**

**_ _ _ _**

**_ _ _ _**

**_ _ _ _ _**

**_ _ _ _ _**

**_ _ _ _ _**

**_ _ _ _ _ _ _**

**_ _ _ _ _ _ _ _ _**

**_ _ _ __ _ _ __ _ ___ _ _**

**_ _ _ _ __ _ _ __ _ ___ _ _**

**_ _ _ _ __ _ _ __ _ ___ _ _**

**SHIP2 subfamily**  ● ● ● ● ●● ● ● ●● ● ●●● ● ●

**3nr8B_Ship2_hu**  599 **FTHLFWFGDLNYRLDMDIQE...ILNYISRKEFEPLLRVDQLNLEREKHKVFLRFSEEEISFPPTYRYERGSRDTYAWHKQkptgvRTnv.....PSWCDRILWKSYPEthIICNSYGCTDDIVTSDHSPVFGTF** 725*

**XP_006038756.1**  561 **FTHLFWFGDLNYRLDMDIQE...ILNYINRKEFEPLLKVDQLNLEKEKHKVFLRFAEEEITFPPTYRYEKGSRDTYVWHKQkptgvRTnv.....PSWCDRILWKSYPEthINCNSYGCTDDVMTSDHSPVFGSF** 687

**SHP2A_DANRE**  583 **FTHLFWFGDLNYRLDMDIQE...ILNYINRKEFDPLLKVDQLNLEREKNKIFLRFAEEEISYPPTYRYERGSRDTYVWQKQkatgmRTnv.....PSWCDRILWKSYPEthIVCNSYGCTDDIVTSDHSPVFGTF** 709

**KFM10957.1**  577 **FTHLFWLGDLNYRVEQPPTEaenIIQKIRQQQYPELLAFDQLLIERKDQKVFLQFEEEEITFAPTYRFERGTREKYAYTKQkatgmKYnl.....PSWCDRVLWKSYPMvhVVCQSYGCTTDIMTSDHSPVFATF** 706

**XP_002934956.3**  465 **LTHLFWAGDLNYRLSMPLQD...ILQCVYSGRYQVLLPVDQLSQERERKKIFLGFKEDSVTFPPTCRYERGTRSYDL-HKA.....RTtgtrlfaPCWSDRVLWTSYPDtdIKCTSYGCTEDIVTSDHSPVFATF** 590

**foreground (120):**  **FTHLFWCGDLNYRVDLDATD AENKVSQGEYDDLLCADQLSREKEDGKAFLNFSEEDISFAPTYRYERGSRDKATGGKT NT PSWCDRVLRKSHPE VVCTAYGCTSDIFTSDHSPVFGSF**

**L LEMPIQE ILKIIKRRQFQP MPV LM RHKH V H K GK T P F LT EYYLWMVY RL I W Y L II QS S D M AT**

**F EVE IQY NKK EV KH NL KER R E E D K SHRR V N N V**

**wt_res_freqs (38): 99999919999992645113 2122323144119711999119241191991819819293999969964854354251 71 99999949199195 459129989199199999999119**

**4 6242254 751153123421 113 11 7151 7 2 1 11 5 6 3 14 22111213 23 5 8 7 2 31 37 1 6 3 88**

**3 131 121 122 31 11 34 121 3 2 6 1 2 1112 3 3 1 3**

**insertions 1 5 52 49 1 1 71 4 1 9 2**

**deletions 111111 11111111112222222111111112111122333322211122113222 33 333332221**

**background (3687):**  **HDHVFWFGDFNYRISGSGDD AKDLVSAGNWSALLANDELTQAKEAGKAFQGWSEGEITFAPSYKYEEGSSNYDSGSKT RA PAWCDRVLWKGSGD VSQVSYDACGSYCTSDHKPVFGTF**

**FEVLILL L F LELPYEH IVR LKKKELEK KF Q LIQMRKNPILH LR APLK P T FSPDTDEF TYM R I SYT I FRSRRK LRLL RRIPLLRM R SLLL**

**YI F DDTR E VRK IEQNDFDE EY RRERK RV R FQ N L DLN ND SE K V Y KN IK SHMEIKI YSI**

**wt_res_freqs (935): 56145639929694111121 1114211111118511919111212512715119417291919851143115621171 91 96859928323111 213129111111128994991117**

**1111212 6 3 3122121 111 21111111 11 8 11221111111 11 2311 3 8 31113311 311 1 1 314 7 131111 2113 12121111 4 2111**

**12 1 3111 2 431 31112111 31 11322 12 1 51 1 1 511 11 54 3 1 3 11 22 4222111 111**

**position**  . 610 . 620 . 630 . 640 . 650 . 660 . 670 . 680 . 690 . 700 . 710 . 720 .
