# Supplementary material for: Inferring joint sequence-structural determinants of protein functional specificity
Source: eLife. 2018 Jan 16;7:e29880. doi: 10.7554/eLife.29880 (PMC5770160; doi:10.7554/eLife.29880)
Supplement: Figure 6—source data 1. [file elife-29880-fig6-data1.docx]

**Figure 6— Source data 1. UDG/TDG superfamily, TDG family and metazoan TDG subfamily.**

**Chordata**  134 **IVIIGINPGLMAAYKGHHYPGPGNHFWKCLFMSGLSEVQLNHMDDHTLPgK.YGIGFTNMVERTTPGSKDLSSKEFREGGRILVQKLQKYQPRIAVFNGKCIYEIFSKevfgvKVKNLE.FGLQPHKIPDT.ETLCYVMPSSS** 273*

**Arthropoda**  196 **IVIIGINPGLFAAYKGHHYAGPGNHFWKCLYLSGLTPEPMTADDDYKLL.K.VGIGFTNMVERATKGSADLTRKEIKEGSQILLEKLQKFKPKIAVFNGKLIFEVFSG.....K-KDFS.FGRQPELVDGT.NTYMWVMPSSS** 328

**Echinodermata**  169 **IVIIGINPGLMAAYKGHHYAGPGNHFWKCLYLSGLVPEPMTCMDDVKLP.D.FGVGFTNIVGRTTRGSADLKRKEIKEGAKVVVEKIQKYLPLIACFNGKGIYEIYSG.....K-KDFE.VGRQPENIPGT.ETVVYVMPSSS** 301

**Mollusca**  103 **ILIVGINPGLCAAYVGHHYAGPGNHFWKCLFLSGLIPEQLNAYDDYKLV.K.YGIGFTNIVARTTRGSADLTRKEIKEGATILSEKVKKYRPKIAVFNGKGIYEVFVG.....H-KNFA.FGKQPEPFAET.ETLVYVMPSSS** 235

**Annelida**  41 **IAIIGINPGLTAAYVGHHYAGPGNHFWKCLYLSGLIPEPMNAYDDTKLR.D.FGIGFTNIVERTSRGSADLTRKEIKEGGEILRSKIQKYKPKIAVFNGKGIYEIFCG.....H-KNFY.IGKQPEPFPGT.DTAVFVMPSSS** 173

**Brachiopoda**  107 **IVIVGINPGLTAAFVGHHYAGPGNHFWKCLFLSELIPEQLNAYDDYKLK.E.FGIGFTNIVERTSRGSADLTKKEIKEGGEILKGKIQKYKPKIAVFNGKGIYEVFVG.....H-KNFH.IGKQPDHFPDCpDTAIFVMPSSS** 240

**Priapulida**  183 **ILIIGINPGLFAAFKGHHYAGPGNHFWKCLYLSGLIPEPMTAMDDYKML.T.FGIGFTNIVARTSRGSADLKRKEIKEGGEILLEKIKAIKPKIAVFNGKGIYEVFSQ.....--NKLEcFGKQPKPIEGT.NTVIYVMPSSS** 315

**Hemichordata**  1 **-------PGLFAAYKGHHYAGPGNHFWKCLYLSGLIPEPMTCMDDFKLH.E.YGIGFTNIVTRTTRGSADLTRKEIKEGGVELLEKMKKYQPKIACFNGKGIYEIFSG.....R-KDFE.IGKQPDPLEGT.NTAVFVMPSSS** 126

**Cnidaria**  49 **ILFVGINPGLMAAYKGHHYPGANNHFWPCLFESGLVPERLTYLDDVRCP.StFGIGLTNMVERTTRGSADLSRKEMRDGKEILITKVQQYKPLIVCFNGKVIYELFAGskc..KV----.-GRQKDPIPGT.DSAVYVMPSTS** 181

**Platyhelminthe**  106 **IVIVGINPSLASAHVGHHYAGPGNHFWTCLSQAGLVPMAVSCYDDSKML.D.YGIGFTNVCTRPTKGAAELTRKEMKAGAAIMLEKMRKYKPKIAVFNGKGIYEAYVG.....H-KNFC.MGRQPTTLDGT.DIVIFVMPSSS** 238

**position**  . 140 . 150 . 160 . 170 . 180 . 190 . 200 . 210 . 220 . 230 . 240 . 250 . 260 . 270

**_ _**

**_ _**

**_ _**

**_ _**

**_ _**

**_ _**

**_ _**

**_ _**

**_ _**

**_ _**

**_ _**

**_ _**

**_ _**

**_ _**

**_ _**

**_ _**

**_ _**

**_ _**

**_ _ _**

**_ _ _**

**_ _ _**

**_ _ _**

**_ _ _**

**_ _ _**

**_ _ _**

**_ _ _**

**_ _ _ _**

**_ _ _ _ _**

**_ _ _ _ _**

**_ _ _ _ _ _**

**_ _ _ _ _ _ __**

**_ _ _ _ _ _ __**

**_ _ _ _ _ _ __**

**_ _ _ _ _ _ _ __**

**_ _ _ _ _ _ _ ___**

**__ _ _ _ _ _ _ ___**

**__ _ _ _ _ _ _ ___**

**___ _ _ _ _ _ _ _ _ _ ___**

**___ _ _ _ _ _ _ _ _ _ ___**

**_____ _ _ _ _ _ _ _ _ _ _ ___**

**_____ _ _ _ _ _ _ _ __ _ _ ___**

**_____ _ _ _ _ _ _ _ __ _ _ ___**

**_____ _ _ _ _ _ _ _ __ _ _ ___**

**_____ _ _ _ _ _ _ _ __ _ _ ___**

**_____ _ _ _ _ _ _ _ __ _ _ ___**

**_____ _ _ _ _ _ _ _ __ _ _ ___**

**_____ _ _ _ _ _ _ _ __ _ _ ___**

**_____ _ _ _ _ _ _ _ __ _ _ ___**

**_____ _ _ _ _ _ _ _ __ _ _ ___**

**_____ _ _ _ _ _ _ _ _ __ _ _ ___**

**_____ _ _ _ _ _ _ _ _ __ _ _ ___**

**UDG/TDG SF** ●●●●● ● ● ● ● ● ● ● ● ●● ● ● ●●●

**3UOB|A**  134 **IVIIGINPGLMAAYKGHHYPGPGNHFWKCLFMSGLSEVQLNHMDDHTLPgK.YGIGFTNMVERTTPGSKDLSSKEFREGGRILVQKLQKYQPRIAVFNGKCIYEIFSKevfgvKVKNLE.FGLQPHKIPDT.ETLCYVMPSSS** 273*

**XP_014278159.1**  196 **IVIIGINPGLFAAYKGHHYAGPGNHFWKCLYLSGLTPEPMTADDDYKLL.K.VGIGFTNMVERATKGSADLTRKEIKEGSQILLEKLQKFKPKIAVFNGKLIFEVFSG.....K-KDFS.FGRQPELVDGT.NTYMWVMPSSS** 328

**XP_011671297.1**  169 **IVIIGINPGLMAAYKGHHYAGPGNHFWKCLYLSGLVPEPMTCMDDVKLP.D.FGVGFTNIVGRTTRGSADLKRKEIKEGAKVVVEKIQKYLPLIACFNGKGIYEIYSG.....K-KDFE.VGRQPENIPGT.ETVVYVMPSSS** 301

**XP_011450941.1**  103 **ILIVGINPGLCAAYVGHHYAGPGNHFWKCLFLSGLIPEQLNAYDDYKLV.K.YGIGFTNIVARTTRGSADLTRKEIKEGATILSEKVKKYRPKIAVFNGKGIYEVFVG.....H-KNFA.FGKQPEPFAET.ETLVYVMPSSS** 235

**ELU08322.1**  41 **IAIIGINPGLTAAYVGHHYAGPGNHFWKCLYLSGLIPEPMNAYDDTKLR.D.FGIGFTNIVERTSRGSADLTRKEIKEGGEILRSKIQKYKPKIAVFNGKGIYEIFCG.....H-KNFY.IGKQPEPFPGT.DTAVFVMPSSS** 173

**XP_013416975.1**  107 **IVIVGINPGLTAAFVGHHYAGPGNHFWKCLFLSELIPEQLNAYDDYKLK.E.FGIGFTNIVERTSRGSADLTKKEIKEGGEILKGKIQKYKPKIAVFNGKGIYEVFVG.....H-KNFH.IGKQPDHFPDCpDTAIFVMPSSS** 240

**XP_014671992.1**  183 **ILIIGINPGLFAAFKGHHYAGPGNHFWKCLYLSGLIPEPMTAMDDYKML.T.FGIGFTNIVARTSRGSADLKRKEIKEGGEILLEKIKAIKPKIAVFNGKGIYEVFSQ.....--NKLEcFGKQPKPIEGT.NTVIYVMPSSS** 315

**FF489386.1_EST**  1 **-------PGLFAAYKGHHYAGPGNHFWKCLYLSGLIPEPMTCMDDFKLH.E.YGIGFTNIVTRTTRGSADLTRKEIKEGGVELLEKMKKYQPKIACFNGKGIYEIFSG.....R-KDFE.IGKQPDPLEGT.NTAVFVMPSSS** 126

**GO000155.1_EST**  49 **ILFVGINPGLMAAYKGHHYPGANNHFWPCLFESGLVPERLTYLDDVRCP.StFGIGLTNMVERTTRGSADLSRKEMRDGKEILITKVQQYKPLIVCFNGKVIYELFAGskc..KV----.-GRQKDPIPGT.DSAVYVMPSTS** 181

**XP_012793347.1**  106 **IVIVGINPSLASAHVGHHYAGPGNHFWTCLSQAGLVPMAVSCYDDSKML.D.YGIGFTNVCTRPTKGAAELTRKEMKAGAAIMLEKMRKYKPKIAVFNGKGIYEAYVG.....H-KNFC.MGRQPTTLDGT.DIVIFVMPSSS** 238

**foreground (23408):**  **VVFVGEAPGAGGGAAGFYYGRAGNNFDKAIGAAGGADEQVSASDGRTWA E EGVGVTNAVKCAEAGGAGSSAGEEAACAADIAAEVARVNIEAFVAWGGTAAEAALG IDGKAG KGGQWFE GGH RSAVFAAYHPA**

**LMLL QD YPRPDQQHPFVPPPLRLLFRMLHELLLDPPPLRPEEQYKLL R QDILLLDILPRVPPEKRPPHPPWLRFTRPVLRRLLRLLRPRLLLLL RY QRKL L LLK LT LLVRP R KL PVVLLPLPS S**

**ILII N HKEAETTV D S KRIY L E I ITFRL K D EY YIW V LTFRR NK LDKE IE ADNF EE IE IK KIIIFV K K F R IS T K I LTYIIVT**

**wt_res_freqs (4311): 4213822941112134111312422212111113211111112211122 1 13411352422111211121124111311121211211111113129117111122 111111 1132111 131 11231111671**

**2113 22 21112111231221212411161111212114152511131 2 2122421111124211212311211111215112212214111232 11 1112 1 111 11 11111 1 11 113121231 6**

**2442 1 11211111 1 4 1221 1 1 1 11112 1 5 11 231 2 22111 31 1111 11 1111 11 22 11 213111 1 1 1 1 21 1 1 1 1611111**

**insertions**

**deletions 1322222222121111 1 12399999999922 1 11 111 1 1 2 393344 44444431822 2991112222**

**position**  . 140 . 150 . 160 . 170 . 180 . 190 . 200 . 210 . 220 . 230 . 240 . 250 . 260 . 270

**_**

**_**

**_**

**_**

**_**

**_**

**_**

**_**

**_**

**_**

**_**

**_**

**_**

**_**

**_**

**_**

**_**

**_**

**_**

**_**

**_**

**_**

**_**

**_**

**_**

**_ _ _**

**_ _ _**

**_ _ _**

**_ _ _**

**_ _ _ _**

**_ _ _ _**

**_ _ _ _ _**

**_ _ _ _ _**

**_ _ _ _ _**

**_ _ _ _ _**

**_ _ _ _ _**

**_ _ _ _ _ _**

**_ _ _ _ _ _ _**

**_ _ _ _ _ _ _**

**_ _ _ _ _ _ _**

**_ _ _ _ _ _ _**

**_ _ _ _ _ _ _ _**

**_ _ _ _ __ _ _ _**

**__ _ _ _ __ _ _ _ _**

**__ _ _ _ __ _ _ _ _ _**

**__ _ _ _ _ _ __ _ _ _ _ _ _**

**__ __ _ _ _ _ _ _ __ _ _ _ _ _ _**

**__ __ _ _ _ _ _ _ __ _ _ _ _ _ _**

**__ ___ _ _ _ _ ___ __ _ _ _ _ _ _**

**__ ___ _ _ _ _ ___ __ _ _ _ _ _ _**

**__ ___ _ _ _ _ ___ __ _ _ _ _ _ _**

**TDG family**  ●● ●●● ● ● ● ● ●●● ●● ● ● ● ● ● ●

**3UOB|A**  134 **IVIIGINPGLMAAYKGHHYPGPGNHFWKCLFMSGLSEVQLNHMDDHTLPgK.YGIGFTNMVERTTPGSKDLSSKEFREGGRILVQKLQKYQPRIAVFNGKCIYEIFSKevfgvKVKNLE.FGLQPHKIPDT.ETLCYVMPSSS** 273*

**XP_014278159.1**  196 **IVIIGINPGLFAAYKGHHYAGPGNHFWKCLYLSGLTPEPMTADDDYKLL.K.VGIGFTNMVERATKGSADLTRKEIKEGSQILLEKLQKFKPKIAVFNGKLIFEVFSG.....K-KDFS.FGRQPELVDGT.NTYMWVMPSSS** 328

**XP_011671297.1**  169 **IVIIGINPGLMAAYKGHHYAGPGNHFWKCLYLSGLVPEPMTCMDDVKLP.D.FGVGFTNIVGRTTRGSADLKRKEIKEGAKVVVEKIQKYLPLIACFNGKGIYEIYSG.....K-KDFE.VGRQPENIPGT.ETVVYVMPSSS** 301

**XP_011450941.1**  103 **ILIVGINPGLCAAYVGHHYAGPGNHFWKCLFLSGLIPEQLNAYDDYKLV.K.YGIGFTNIVARTTRGSADLTRKEIKEGATILSEKVKKYRPKIAVFNGKGIYEVFVG.....H-KNFA.FGKQPEPFAET.ETLVYVMPSSS** 235

**ELU08322.1**  41 **IAIIGINPGLTAAYVGHHYAGPGNHFWKCLYLSGLIPEPMNAYDDTKLR.D.FGIGFTNIVERTSRGSADLTRKEIKEGGEILRSKIQKYKPKIAVFNGKGIYEIFCG.....H-KNFY.IGKQPEPFPGT.DTAVFVMPSSS** 173

**XP_013416975.1**  107 **IVIVGINPGLTAAFVGHHYAGPGNHFWKCLFLSELIPEQLNAYDDYKLK.E.FGIGFTNIVERTSRGSADLTKKEIKEGGEILKGKIQKYKPKIAVFNGKGIYEVFVG.....H-KNFH.IGKQPDHFPDCpDTAIFVMPSSS** 240

**XP_014671992.1**  183 **ILIIGINPGLFAAFKGHHYAGPGNHFWKCLYLSGLIPEPMTAMDDYKML.T.FGIGFTNIVARTSRGSADLKRKEIKEGGEILLEKIKAIKPKIAVFNGKGIYEVFSQ.....--NKLEcFGKQPKPIEGT.NTVIYVMPSSS** 315

**FF489386.1_EST**  1 **-------PGLFAAYKGHHYAGPGNHFWKCLYLSGLIPEPMTCMDDFKLH.E.YGIGFTNIVTRTTRGSADLTRKEIKEGGVELLEKMKKYQPKIACFNGKGIYEIFSG.....R-KDFE.IGKQPDPLEGT.NTAVFVMPSSS** 126

**GO000155.1_EST**  49 **ILFVGINPGLMAAYKGHHYPGANNHFWPCLFESGLVPERLTYLDDVRCP.StFGIGLTNMVERTTRGSADLSRKEMRDGKEILITKVQQYKPLIVCFNGKVIYELFAGskc..KV----.-GRQKDPIPGT.DSAVYVMPSTS** 181

**XP_012793347.1**  106 **IVIVGINPSLASAHVGHHYAGPGNHFWTCLSQAGLVPMAVSCYDDSKML.D.YGIGFTNVCTRPTKGAAELTRKEMKAGAAIMLEKMRKYKPKIAVFNGKGIYEAYVG.....H-KNFC.MGRQPTTLDGT.DIVIFVMPSSS** 238

**foreground (1639):**  **VVFCGFNPGISSGATGHHYAGPGNRFWKCIHEAGFTDEQVSASDGRTLL A YGCGNTNVVARASAGAADISAAEYRAGGAALAAKVAEYQPEAACFNGKGAWEAASG DKKEAE WGRQDESWGGA ESAVWVATSSS**

**LLII I SLMTAEV FAF H S L YPLLFLS L PRPLRPEEQYK P K L L L L E PTVRSDELTRK M E VPL LR LRRLR RILAIL ITIFRVIFR RPPRVR V P PRPLPEV RVVLF MPNP**

**I V W R H RI YR I E L K D E E F I I I D RS S KE L RRI VE IEKFK KVV VV V VY QVW KVRKFK F L E KI DT TYIY L T**

**wt_res_freqs (321): 4283919971151348664818495984115139371111112211184 1 36191981839313162214119242831191273214191213319534232413 142141 29192311342 11244821429**

**2612 6 2513611 115 3 2 1 1328115 3 45141525111 3 1 1 3 1 3 1 58121367321 1 3 211 11 11314 323412 123131142 212111 1 1 3111111 31313 1844**

**2 3 1 2 1 11 11 1 1 2 1 5 1 2 1 2 3 3 1 21 1 23 3 121 13 32112 124 34 1 16 111 212321 1 2 1 13 14 6111 3 2**

**insertions 3 4**

**deletions 8654333333111111111 1111111 7 1 11223222333 39934444321**

**background (21769):**  **VVFVGSAPGAGGGEAGFYYGRAGNNFDKALGAAGGADQ WA E EGVAVTNAVKCREGGGAGSTAGEEAACDADFAAEIARVNIEVFVAWGGTAAQAALG IDGGVG KTGQWFE GGH L VFASYSPA**

**LMLL QD YPRPDRQHPFVPPSLRLLFRM EELLLDPP LL L QDILLLDVLPTVPPEKRPPHPPWIEVTRPVLRRLLRLLRPRLLLLL RY QRLL L LLKPLT LLVRV L KL P LLPLPH S**

**ILII E HKE QSTV GD KRIY L A I ITF Y R FIW T L FRR NKNDDKE R FA NFIK I E IK KIIITV K KKF R AKIS AK R I IIVT**

**wt_res_freqs (3990): 42128139411121331113125121121611132111 32 1 13411252322111211121123211311111212211111313229117111132 111111 1141111 131 1 31121171**

**2213 32 211121113322242124111 11112121 31 1 2122421211224211312311211121214113212214111233 11 1112 1 111111 11111 1 11 1 122237 6**

**2342 2 112 1111 11 1121 1 1 1 111 1 2 121 1 2 111 3111111 1 11 1121 1 2 11 213211 1 211 1 1121 11 1 1 1111**

**position**  . 140 . 150 . 160 . 170 . 180 . 190 . 200 . 210 . 220 . 230 . 240 . 250 . 260 . 270

**_**

**_**

**_**

**_**

**_**

**_**

**_**

**_**

**_**

**_**

**_**

**_**

**_**

**_**

**_**

**_**

**_**

**_**

**_**

**_**

**_**

**_**

**_**

**_**

**_**

**_**

**_ _**

**_ _**

**_ _**

**_ _**

**_ _**

**_ _**

**_ _**

**_ _**

**_ _**

**_ _**

**_ _**

**_ _**

**_ _**

**_ _**

**_ _**

**_ _**

**_ _ _ _ _ _**

**_ _ _ _ _ _**

**_ _ _ _ _ _ _**

**_ _ _ __ _ _ _ _**

**_ _ _ __ _ _ _ _ _**

**_ _ _ _ _ __ _ _ _ _ _ _**

**_ _ _ _ _ _ __ _ _ __ __ _ _ _ _**

**_ _ _ _ _ _ __ _ _ _ _____ _ _ _ _**

**_ _ _ _ _ _ __ _ _ _ _____ _ _ _ _**

**Metazoan TDG**  ● ● ● ● ● ● ●● ● ● ● ●●●●● ● ● ● ●

**3UOB|A**  134 **IVIIGINPGLMAAYKGHHYPGPGNHFWKCLFMSGLSEVQLNHMDDHTLPgK.YGIGFTNMVERTTPGSKDLSSKEFREGGRILVQKLQKYQPRIAVFNGKCIYEIFSKevfgvKVKNLE.FGLQPHKIPDT.ETLCYVMPSSS** 273*

**XP_014278159.1**  196 **IVIIGINPGLFAAYKGHHYAGPGNHFWKCLYLSGLTPEPMTADDDYKLL.K.VGIGFTNMVERATKGSADLTRKEIKEGSQILLEKLQKFKPKIAVFNGKLIFEVFSG.....K-KDFS.FGRQPELVDGT.NTYMWVMPSSS** 328

**XP_011671297.1**  169 **IVIIGINPGLMAAYKGHHYAGPGNHFWKCLYLSGLVPEPMTCMDDVKLP.D.FGVGFTNIVGRTTRGSADLKRKEIKEGAKVVVEKIQKYLPLIACFNGKGIYEIYSG.....K-KDFE.VGRQPENIPGT.ETVVYVMPSSS** 301

**XP_011450941.1**  103 **ILIVGINPGLCAAYVGHHYAGPGNHFWKCLFLSGLIPEQLNAYDDYKLV.K.YGIGFTNIVARTTRGSADLTRKEIKEGATILSEKVKKYRPKIAVFNGKGIYEVFVG.....H-KNFA.FGKQPEPFAET.ETLVYVMPSSS** 235

**ELU08322.1**  41 **IAIIGINPGLTAAYVGHHYAGPGNHFWKCLYLSGLIPEPMNAYDDTKLR.D.FGIGFTNIVERTSRGSADLTRKEIKEGGEILRSKIQKYKPKIAVFNGKGIYEIFCG.....H-KNFY.IGKQPEPFPGT.DTAVFVMPSSS** 173

**XP_013416975.1**  107 **IVIVGINPGLTAAFVGHHYAGPGNHFWKCLFLSELIPEQLNAYDDYKLK.E.FGIGFTNIVERTSRGSADLTKKEIKEGGEILKGKIQKYKPKIAVFNGKGIYEVFVG.....H-KNFH.IGKQPDHFPDCpDTAIFVMPSSS** 240

**XP_014671992.1**  183 **ILIIGINPGLFAAFKGHHYAGPGNHFWKCLYLSGLIPEPMTAMDDYKML.T.FGIGFTNIVARTSRGSADLKRKEIKEGGEILLEKIKAIKPKIAVFNGKGIYEVFSQ.....--NKLEcFGKQPKPIEGT.NTVIYVMPSSS** 315

**FF489386.1_EST**  1 **-------PGLFAAYKGHHYAGPGNHFWKCLYLSGLIPEPMTCMDDFKLH.E.YGIGFTNIVTRTTRGSADLTRKEIKEGGVELLEKMKKYQPKIACFNGKGIYEIFSG.....R-KDFE.IGKQPDPLEGT.NTAVFVMPSSS** 126

**GO000155.1_EST**  49 **ILFVGINPGLMAAYKGHHYPGANNHFWPCLFESGLVPERLTYLDDVRCP.StFGIGLTNMVERTTRGSADLSRKEMRDGKEILITKVQQYKPLIVCFNGKVIYELFAGskc..KV----.-GRQKDPIPGT.DSAVYVMPSTS** 181

**XP_012793347.1**  106 **IVIVGINPSLASAHVGHHYAGPGNHFWTCLSQAGLVPMAVSCYDDSKML.D.YGIGFTNVCTRPTKGAAELTRKEMKAGAAIMLEKMRKYKPKIAVFNGKGIYEAYVG.....H-KNFC.MGRQPTTLDGT.DIVIFVMPSSS** 238

**foreground (137):**  **IVFVGINPGLFAAYKGRHYAGPGNHFWKCLHESGFSDEQVSASDDTTCL S YGIGFTNMVARATKGSADLSSKEFKDGGEQLVEKIQQYQPKIAVFNGKCIYEVFCG KAKNFE FGKQPDKVDGC NSACWVMPSSS**

**LII S MS FI HYFPN Y FM LIPVPMNFMH YRLP K FKM L I E T PS K KR LRE SRI LQ LKKFR LVVA L M SR P KLH L YPIPET EVVMFL**

**I L W YL VE FLTYDE HK Q S R TK I AK K R G I AK V E R HR ED DTYLY**

**wt_res_freqs (27): 8612999989279759278488899988891199111531221591213 1 67898997929392894995379131941182691517194886999949993924 827353 89198242141 21223689999**

**277 1 42 11 61151 1 41 8141224121 2185 4 111 1 2 4 5 41 4 14 158 236 31 73521 3111 2 1 51 2 241 4 116518 313112**

**1 1 1 36 12 152112 13 1 1 2 21 5 12 6 1 2 3 12 3 1 3 21 13 36114**

**insertions 3 8 5**

**deletions 2119998777666662444444455551111111 253345 52222222223 31134566442**

**background (1502):**  **VVFCGFNPGISSGATGHAYANPGNHFWKAIHSAGFTDRQLTAADGRTLL A RGCGNTNVVARASARAADISAAEYAAGGAALAAKVAEYQPEIACFVGKGAWEAFSG DKKEAA WGRQDESWGGA E VWVATSSS**

**LL I I SLMTAEV FPF H T L YPLLYLS L P L RPEEQ E P E LRL L L E PTV DELTRE MRE VPL LR LRRLR RALAIL IQIFRQIFR R PRVR V P PRTLPEV R LF LPNP**

**I V W H R S R RI R I R K S D D F I I I D R S KD LD RRI VE IEK K KVV V VTVY TVW K RKFK Y L E I DT I V T**

**wt_res_freqs (294): 5284919971161348513828491983215139381614111111184 1 16191972839314372114119212831191264213191114359534232113 152141 39192312442 1 44821419**

**26 1 5 2513611 116 3 1 1 1328115 3 4 2 16251 1 3 2 113 1 4 1 581 377313 143 211 11 11314 323512 112141142 2 2111 1 1 2121111 3 13 4745**

**1 3 1 6 2 2 6 11 1 2 1 1 2 5 2 1 2 4 3 1 2 2 21 31 121 12 321 1 124 3 1315 111 1 2321 1 1 1 3 14 1 1 2**

**position**  . 140 . 150 . 160 . 170 . 180 . 190 . 200 . 210 . 220 . 230 . 240 . 250 . 260 . 270

**Figure 6— Source data 1. UDG/TDG superfamily and UDG family.**

**Deinococcus**  53 **ILLFGLAPGAHGSNRTGRPFTGDASGAFLYPLLHEAGLSSKPES-LPGDDLRLYGVYLTAAVRCAPPKNKPTPEELRACARWTEVEL.GLLPEVRVYVALGRIALEALLAHFGLRKsa...............HP.....FRHGAHYPL...PGG....RHLLASYHVS** 192*

**Actinobacteria**  71 **VLVVGLAPAAHGANRTGRVFTGDRSGDWLFASMHRVGLADQPTSRHRDDGLRLRGARVTAAVRCAPPANKPTPQERDTCAPWLDRELrEVLPTLRAVVCLGGFGWAAALTALDGAGvavprpr..........PR.....FGHGAEVVLag.PHGp...LTVLGCYHPS** 220

**Proteobacteria**  56 **IALVGLAPGAHGSNRTGRMFTGDRSGDFLYAALHRAGLASQPTSRARDDGLALDGAWITSACRCAPPDNRPSPDELARCAPFLDREL.ALL-RPRVLVALGSVGWDAILAALRRAGrevprpr..........PR.....FGHGAELRL...PGL....PAVLGCYHPS** 200

**Acidobacteria**  72 **LLIVGLAPAAHGGNRTGRLFTGDRSGDWLFRALHRAGFANQPTSTHREDGLQLINAYICAAVRCAPPGNRPLPEEAETCLPFLVREM.ELLPEVRVIVALGQFAFEQTLKALRQQGkvlpkpk..........PR.....FAHGARYDL...APG....LTLLGSYHPS** 217

**Nitrospirae**  55 **LYVLGLAPAAHGGNRTGRVFTGDRSGDWLYEALYRHGFANQPTSHHRDDGLSLKDCYIGATVRCAPPGNKPTPDEFLSCRQYLQAEI.RLLKNHRVVVALGKIAFDHYLKTCRSQGrmipvpa..........PK.....FGHGAVYRL...PWG....VTLIGSYHPS** 200

**Gemmatimonadet**  59 **LLIVGLAPAAHGANRTGRMFTGDRSGDFLYAALHRAGLGSQAESRRRDDGLRLQGVYITAVVRCAPPANRPTPEERANCREYLDREL.DALTGVRVILALGGYAYAHVLRTLRERGhpvpspt..........PK.....FAHGRVVEL...GQAa...TAVLASYHPS** 205

**Thaumarchaeota**  57 **LLIVGLAPAAHGGNRTGRMFTGDSSGDWLARAMHETGFASMPTSRSRDDGLVLKDAYITAAVRCAPPDNKPLPSELRNCSQYLISEL.KLLDKVRVVLALGKIGFDAYCRAVGAKG.................LS.....FGHGARHQV...-DG....KTLLASYHPS** 194

**unknown**  55 **LLIVGLAPAAHGGNRTGRLFTGDSSGEWLMEALHTARFANQPTSAHGRDGLRLRGAYITAPVRCAPPANRPTPQELEACLPFLVREL.ELLPEVRVVVALGKFGFDAYLRAREIAGqpvpkpr..........PR.....FRHGGLTRF...SDG....VRLLASYHPS** 200

**Armatimonadete**  54 **LLIVGLAPAAHGGNRTGRIFTGDASGNFLFEALYRAGFANQPTSVSRDDGLTLRDALITAAVHCAPPDNKPTPEEQASCFPFLKATY.RLMPNLQGMLALGQIAFSACVRLARMENllppqar..........PT.....FQHGAIYEL...TDG....KFLAASYHPS** 199

**Chloroflexi**  65 **LVIIGLAPAAHGGNRTGRPFTGDASGNWLYRALYRAGFANQPTSLHRDDGLQLFDAYITAVCHCVPPNNRPTPDELAACSGYLAREL.ALLPRVRVLLCLGAVAFETTLRVMRARGydwpvavandderperrLR.....FQHGGEYRWrtsPPGlppaPVLLASYHPS** 227

**Planctomycetes**  57 **LLIVGLAPAAHGANRTGRMFTGDRSGDWLYRALFKAGFASQPAAEHISDGLTLINCAITATCHCAPPANKPTREEIENCHPWLEQTV.DLL-PVQVFLALGQIGWKAVLDFKKRQGkltgkr...........PV.....FSHGAEYQF...PDG....HWLVGSYHPS** 200

**Verrucomicrobi**  67 **LVLVGLAPAAHGGNRTGRMFTGDRSGDWLFRALHRAGFASQPFSRDREDGLRISDCFVSAAVRCCPPENRPLPLERENCYRFLAREL.EALANLRVLIALGGMAFETLLRWVKERWpeearqmlrkr......PS.....FSHGAEIAL...PKG....GTLLATYHPS** 216

**Crenarchaeota**  57 **LMVVGLAPAAHGGNRTGRMFTGDSSAQFLFKALHALGLASEPYSVSADDGVRLRCVYITSAVKCAPPDNKPTAEEAANCLPWLVEEI.RLV-RPRAVVALGSLAWRSVFRALGRPA.................PP.....FAHGARAEV...-GG....VAVFASYHPS** 193

**Aquificae**  55 **LLIVGLAPAAHGGNRTGRMFTGDSSGNWLARALYETGFANKPESVNKDDGLDLKGAYITAVVRCAPPENKPTKEEMENCNTFLIREL.EILKEVKVILCLGSIALKGTLMALKKLYptaqlrg..........IK.....FGHNFVYKP...-EGlp..YTLMTSYHPS** 201

**Candidatus Tec**  54 **LLLIGLAPAAHGGNRTGRAFTGDRSGEWLYDALYRYGFANQPTSEHRGDGLQLHDCYIAQVLHCAPPANKPTREEMLQCQSYLLQEW.QLLPRLQLIIPLGKIAFDACLRICRELDyplptpl..........PR.....FAHGALYRL...ENG....MVLQPSYHPS** 199

**Euryarchaeota**  60 **LLIVGLAPAASGGNRTGRVFTGDKSSDFLVSCLHEAGITNQPTSERRDDGLIYYDAYITAAVKCVPPDNKPLPEEIENCSVYLRSEI.GFMKNLKVILVLGQIALQAVVRLIADPAe................PRsryr.FVHGAVYSM...-NG....IRVVCSYHPS** 202

**Cyanobacteria**  65 **LWVIGLAPAAHGGNRTGRVFTGDPSGDWLFRALYRAGFANQPTSTHREDGLQLQDCYISAVVRCVPPENRPTAIEAKTCLGYLKQEL.ELLTQVRVILTLGHFAFQHALTLLPPLR.................PRpr...FGHNCVIPL...AEG....RYLLASYHPS** 205

**Chlamydiae**  52 **LLILGLAPSAHGGNRTGRIFTGDESARFLMKMLYQVGFANQPTSFSRDDGLKLSGCYITAAVKCAPPENRPLKEECDNCLPYLKQEF.ALLPHLKAVLALGELAYKAIFSVLNKEN.................LKenklpFKHASLLSF...-GE....IDLFTSYHPS** 194

**position**  . 60 . 70 . 80 . 90 . 100 . 110 . 120 . 130 . 140 . 150 . 160 . 170 . 180 . 190

**_**

**_ _**

**_ _**

**_ _**

**_ _**

**_ _**

**_ _**

**_ _**

**_ _**

**_ _**

**_ _**

**_ _**

**_ _**

**_ _**

**_ _**

**_ _**

**_ _**

**_ _**

**_ _**

**_ _ _**

**_ _ _**

**_ _ _**

**_ _ _**

**_ _ _**

**_ _ _**

**_ _ _**

**_ _ _**

**_ _ _**

**_ _ _ _ _**

**_ _ _ _ _ _ _**

**_ _ _ _ _ _ _**

**_ _ _ _ _ _ __**

**_ _ _ _ _ _ __**

**_ _ _ _ _ _ __**

**_ _ _ _ _ _ _ __**

**__ _ _ _ _ _ _ ___**

**___ _ _ _ _ _ _ ___**

**___ _ _ _ _ _ _ _ _ ___**

**___ _ _ _ _ _ _ _ _ _ _ ___**

**_____ _ _ _ _ _ _ _ _ _ _ ___**

**_____ _ _ _ _ _ _ _ _ _ _ ___**

**_____ _ _ _ _ _ _ _ __ _ _ ___**

**_____ _ _ _ _ _ _ _ __ _ _ ___**

**_____ _ _ _ _ _ _ _ __ _ _ ___**

**_____ _ _ _ _ _ _ _ __ _ _ ___**

**_____ _ _ _ _ _ _ _ __ _ _ ___**

**_____ _ _ _ _ _ _ _ __ _ _ ___**

**_____ _ _ _ _ _ _ _ __ _ _ ___**

**_____ _ _ _ _ _ _ _ __ _ _ ___**

**_____ _ _ _ _ _ _ _ _ __ _ _ ___**

**_____ _ _ _ _ _ _ _ _ __ _ _ ___**

**UDG/TDG SF** ●●●●● ● ● ● ● ● ● ● ● ●● ● ● ●●●

**2D3Y|A**  53 **ILLFGLAPGAHGSNRTGRPFTGDASGAFLYPLLHEAGLSSKPES-LPGDDLRLYGVYLTAAVRCAPPKNKPTPEELRACARWTEVEL.GLLPEVRVYVALGRIALEALLAHFGLRKsa...............HP.....FRHGAHYPL...PGG....RHLLASYHVS** 192*

**WP_036194455.1**  71 **VLVVGLAPAAHGANRTGRVFTGDRSGDWLFASMHRVGLADQPTSRHRDDGLRLRGARVTAAVRCAPPANKPTPQERDTCAPWLDRELrEVLPTLRAVVCLGGFGWAAALTALDGAGvavprpr..........PR.....FGHGAEVVLag.PHGp...LTVLGCYHPS** 220

**WP_012527668.1**  56 **IALVGLAPGAHGSNRTGRMFTGDRSGDFLYAALHRAGLASQPTSRARDDGLALDGAWITSACRCAPPDNRPSPDELARCAPFLDREL.ALL-RPRVLVALGSVGWDAILAALRRAGrevprpr..........PR.....FGHGAELRL...PGL....PAVLGCYHPS** 200

**WP_014099162.1**  72 **LLIVGLAPAAHGGNRTGRLFTGDRSGDWLFRALHRAGFANQPTSTHREDGLQLINAYICAAVRCAPPGNRPLPEEAETCLPFLVREM.ELLPEVRVIVALGQFAFEQTLKALRQQGkvlpkpk..........PR.....FAHGARYDL...APG....LTLLGSYHPS** 217

**CUS33790.1**  55 **LYVLGLAPAAHGGNRTGRVFTGDRSGDWLYEALYRHGFANQPTSHHRDDGLSLKDCYIGATVRCAPPGNKPTPDEFLSCRQYLQAEI.RLLKNHRVVVALGKIAFDHYLKTCRSQGrmipvpa..........PK.....FGHGAVYRL...PWG....VTLIGSYHPS** 200

**KPK79306.1**  59 **LLIVGLAPAAHGANRTGRMFTGDRSGDFLYAALHRAGLGSQAESRRRDDGLRLQGVYITAVVRCAPPANRPTPEERANCREYLDREL.DALTGVRVILALGGYAYAHVLRTLRERGhpvpspt..........PK.....FAHGRVVEL...GQAa...TAVLASYHPS** 205

**AFU56960.1**  57 **LLIVGLAPAAHGGNRTGRMFTGDSSGDWLARAMHETGFASMPTSRSRDDGLVLKDAYITAAVRCAPPDNKPLPSELRNCSQYLISEL.KLLDKVRVVLALGKIGFDAYCRAVGAKG.................LS.....FGHGARHQV...-DG....KTLLASYHPS** 194

**KRT75451.1**  55 **LLIVGLAPAAHGGNRTGRLFTGDSSGEWLMEALHTARFANQPTSAHGRDGLRLRGAYITAPVRCAPPANRPTPQELEACLPFLVREL.ELLPEVRVVVALGKFGFDAYLRAREIAGqpvpkpr..........PR.....FRHGGLTRF...SDG....VRLLASYHPS** 200

**CUU03299.1**  54 **LLIVGLAPAAHGGNRTGRIFTGDASGNFLFEALYRAGFANQPTSVSRDDGLTLRDALITAAVHCAPPDNKPTPEEQASCFPFLKATY.RLMPNLQGMLALGQIAFSACVRLARMENllppqar..........PT.....FQHGAIYEL...TDG....KFLAASYHPS** 199

**WP_051913883.1**  65 **LVIIGLAPAAHGGNRTGRPFTGDASGNWLYRALYRAGFANQPTSLHRDDGLQLFDAYITAVCHCVPPNNRPTPDELAACSGYLAREL.ALLPRVRVLLCLGAVAFETTLRVMRARGydwpvavandderperrLR.....FQHGGEYRWrtsPPGlppaPVLLASYHPS** 227

**WP_002646251.1**  57 **LLIVGLAPAAHGANRTGRMFTGDRSGDWLYRALFKAGFASQPAAEHISDGLTLINCAITATCHCAPPANKPTREEIENCHPWLEQTV.DLL-PVQVFLALGQIGWKAVLDFKKRQGkltgkr...........PV.....FSHGAEYQF...PDG....HWLVGSYHPS** 200

**WP_018289825.1**  67 **LVLVGLAPAAHGGNRTGRMFTGDRSGDWLFRALHRAGFASQPFSRDREDGLRISDCFVSAAVRCCPPENRPLPLERENCYRFLAREL.EALANLRVLIALGGMAFETLLRWVKERWpeearqmlrkr......PS.....FSHGAEIAL...PKG....GTLLATYHPS** 216

**KJR72747.1**  57 **LMVVGLAPAAHGGNRTGRMFTGDSSAQFLFKALHALGLASEPYSVSADDGVRLRCVYITSAVKCAPPDNKPTAEEAANCLPWLVEEI.RLV-RPRAVVALGSLAWRSVFRALGRPA.................PP.....FAHGARAEV...-GG....VAVFASYHPS** 193

**WP_008286937.1**  55 **LLIVGLAPAAHGGNRTGRMFTGDSSGNWLARALYETGFANKPESVNKDDGLDLKGAYITAVVRCAPPENKPTKEEMENCNTFLIREL.EILKEVKVILCLGSIALKGTLMALKKLYptaqlrg..........IK.....FGHNFVYKP...-EGlp..YTLMTSYHPS** 201

**ETX02221.1**  54 **LLLIGLAPAAHGGNRTGRAFTGDRSGEWLYDALYRYGFANQPTSEHRGDGLQLHDCYIAQVLHCAPPANKPTREEMLQCQSYLLQEW.QLLPRLQLIIPLGKIAFDACLRICRELDyplptpl..........PR.....FAHGALYRL...ENG....MVLQPSYHPS** 199

**WP_010900904.1**  60 **LLIVGLAPAASGGNRTGRVFTGDKSSDFLVSCLHEAGITNQPTSERRDDGLIYYDAYITAAVKCVPPDNKPLPEEIENCSVYLRSEI.GFMKNLKVILVLGQIALQAVVRLIADPAe................PRsryr.FVHGAVYSM...-NG....IRVVCSYHPS** 202

**WP_011434002.1**  65 **LWVIGLAPAAHGGNRTGRVFTGDPSGDWLFRALYRAGFANQPTSTHREDGLQLQDCYISAVVRCVPPENRPTAIEAKTCLGYLKQEL.ELLTQVRVILTLGHFAFQHALTLLPPLR.................PRpr...FGHNCVIPL...AEG....RYLLASYHPS** 205

**WP_013943516.1**  52 **LLILGLAPSAHGGNRTGRIFTGDESARFLMKMLYQVGFANQPTSFSRDDGLKLSGCYITAAVKCAPPENRPLKEECDNCLPYLKQEF.ALLPHLKAVLALGELAYKAIFSVLNKEN.................LKenklpFKHASLLSF...-GE....IDLFTSYHPS** 194

**foreground (23408):**  **VVFVGEAPGAGGGAAGGRVFYYGRAGNNFDKALGAAGGADEATATSAGDGWAEEGVGVTNAVKCATAGGAGSAGEEAACAADIAAEV ARVTNIEAFVAWGGTAAEAALGIDGKAG KG FGHGGQWFE LGG HRVFAAYHPA**

**LMLL QD YPRPDQQHR PPFVPPPLRLLFRM HELLLDPPPESIHRD LLRQDILLLDLLPRVPPEKRPPPPWLRFTRPVLRRLL RLLPRPRLLLLL RY QRKL LLLK LT LL S VRP R K LPLLPLPS S**

**ILII N HKEAETT MV D S KRIY L E I ITFR V N Y YIW I LTFRR NK LKE IE ADNF EE I E IKK KIIIFV K K F R IS T A K ILIIVT**

**wt_res_freqs (4199): 421382295111213439111131242221216111321111311312982211341135242211121112124111311121211 2111111113119117111122111111 11 939732111 113 1131111671**

**2113 22 211121111 12312212124111 111121216181243 4122122421111124211212112111112151122 122314111232 11 1112 1111 11 11 1 111 1 1 11121231 6**

**2442 1 1121111 31 1 4 1221 1 1 1 1111 2 1 1 221 1 22111 21 111 11 1111 11 2 2 111 213111 1 1 1 1 21 1 1 1 111111**

**insertions 1**

**deletions 1322222222121111199 1 12399999999922111 111 1 9 3453344 44 999944443 182 221112222**

**position**  . 60 . 70 . 80 . 90 . 100 . 110 . 120 . 130 . 140 . 150 . 160 . 170 . 180 . 190

**_**

**_**

**_**

**_**

**_**

**_**

**_**

**_**

**_**

**_**

**_**

**_**

**_**

**_**

**_**

**_**

**_**

**_**

**_**

**_**

**_**

**_**

**_**

**_**

**_**

**_**

**_**

**_**

**_**

**_**

**_ _**

**_ _ _**

**_ _ _ _**

**_ _ _ _**

**_ _ _ _**

**_ _ _ _**

**_ _ _ _**

**_ _ _ _**

**_ _ _ _ _ _**

**_ _ _ _ _ _**

**_ _ __ _ _ _ _**

**__ _ __ _ _ _ _**

**__ _ __ _ _ _ _**

**_ __ _ __ _ _ _ _ _**

**_ __ _ __ _ _ _ _ _**

**_ _ __ _ __ _ _ _ _ _**

**_ _ __ _ __ _ __ _ _ _**

**_ _ ___ _ ___ _ _ __ _ _ _ _**

**_ __ ___ _ ____ _ _ __ _ _ _ _**

**_ __ ___ _ ____ _ _ __ _ _ _ __**

**_ __ ___ _ ____ _ _ __ _ _ _ __**

**UDG family**  ● ●● ●●● ● ●●●● ● ● ●● ● ● ● ●●

**2D3Y|A**  53 **ILLFGLAPGAHGSNRTGRPFTGDASGAFLYPLLHEAGLSSKPES-LPGDDLRLYGVYLTAAVRCAPPKNKPTPEELRACARWTEVEL.GLLPEVRVYVALGRIALEALLAHFGLRKsa...............HP.....FRHGAHYPL...PGG....RHLLASYHVS** 192*

**WP_036194455.1**  71 **VLVVGLAPAAHGANRTGRVFTGDRSGDWLFASMHRVGLADQPTSRHRDDGLRLRGARVTAAVRCAPPANKPTPQERDTCAPWLDRELrEVLPTLRAVVCLGGFGWAAALTALDGAGvavprpr..........PR.....FGHGAEVVLag.PHGp...LTVLGCYHPS** 220

**WP_012527668.1**  56 **IALVGLAPGAHGSNRTGRMFTGDRSGDFLYAALHRAGLASQPTSRARDDGLALDGAWITSACRCAPPDNRPSPDELARCAPFLDREL.ALL-RPRVLVALGSVGWDAILAALRRAGrevprpr..........PR.....FGHGAELRL...PGL....PAVLGCYHPS** 200

**WP_014099162.1**  72 **LLIVGLAPAAHGGNRTGRLFTGDRSGDWLFRALHRAGFANQPTSTHREDGLQLINAYICAAVRCAPPGNRPLPEEAETCLPFLVREM.ELLPEVRVIVALGQFAFEQTLKALRQQGkvlpkpk..........PR.....FAHGARYDL...APG....LTLLGSYHPS** 217

**CUS33790.1**  55 **LYVLGLAPAAHGGNRTGRVFTGDRSGDWLYEALYRHGFANQPTSHHRDDGLSLKDCYIGATVRCAPPGNKPTPDEFLSCRQYLQAEI.RLLKNHRVVVALGKIAFDHYLKTCRSQGrmipvpa..........PK.....FGHGAVYRL...PWG....VTLIGSYHPS** 200

**KPK79306.1**  59 **LLIVGLAPAAHGANRTGRMFTGDRSGDFLYAALHRAGLGSQAESRRRDDGLRLQGVYITAVVRCAPPANRPTPEERANCREYLDREL.DALTGVRVILALGGYAYAHVLRTLRERGhpvpspt..........PK.....FAHGRVVEL...GQAa...TAVLASYHPS** 205

**AFU56960.1**  57 **LLIVGLAPAAHGGNRTGRMFTGDSSGDWLARAMHETGFASMPTSRSRDDGLVLKDAYITAAVRCAPPDNKPLPSELRNCSQYLISEL.KLLDKVRVVLALGKIGFDAYCRAVGAKG.................LS.....FGHGARHQV...-DG....KTLLASYHPS** 194

**KRT75451.1**  55 **LLIVGLAPAAHGGNRTGRLFTGDSSGEWLMEALHTARFANQPTSAHGRDGLRLRGAYITAPVRCAPPANRPTPQELEACLPFLVREL.ELLPEVRVVVALGKFGFDAYLRAREIAGqpvpkpr..........PR.....FRHGGLTRF...SDG....VRLLASYHPS** 200

**CUU03299.1**  54 **LLIVGLAPAAHGGNRTGRIFTGDASGNFLFEALYRAGFANQPTSVSRDDGLTLRDALITAAVHCAPPDNKPTPEEQASCFPFLKATY.RLMPNLQGMLALGQIAFSACVRLARMENllppqar..........PT.....FQHGAIYEL...TDG....KFLAASYHPS** 199

**WP_051913883.1**  65 **LVIIGLAPAAHGGNRTGRPFTGDASGNWLYRALYRAGFANQPTSLHRDDGLQLFDAYITAVCHCVPPNNRPTPDELAACSGYLAREL.ALLPRVRVLLCLGAVAFETTLRVMRARGydwpvavandderperrLR.....FQHGGEYRWrtsPPGlppaPVLLASYHPS** 227

**WP_002646251.1**  57 **LLIVGLAPAAHGANRTGRMFTGDRSGDWLYRALFKAGFASQPAAEHISDGLTLINCAITATCHCAPPANKPTREEIENCHPWLEQTV.DLL-PVQVFLALGQIGWKAVLDFKKRQGkltgkr...........PV.....FSHGAEYQF...PDG....HWLVGSYHPS** 200

**WP_018289825.1**  67 **LVLVGLAPAAHGGNRTGRMFTGDRSGDWLFRALHRAGFASQPFSRDREDGLRISDCFVSAAVRCCPPENRPLPLERENCYRFLAREL.EALANLRVLIALGGMAFETLLRWVKERWpeearqmlrkr......PS.....FSHGAEIAL...PKG....GTLLATYHPS** 216

**KJR72747.1**  57 **LMVVGLAPAAHGGNRTGRMFTGDSSAQFLFKALHALGLASEPYSVSADDGVRLRCVYITSAVKCAPPDNKPTAEEAANCLPWLVEEI.RLV-RPRAVVALGSLAWRSVFRALGRPA.................PP.....FAHGARAEV...-GG....VAVFASYHPS** 193

**WP_008286937.1**  55 **LLIVGLAPAAHGGNRTGRMFTGDSSGNWLARALYETGFANKPESVNKDDGLDLKGAYITAVVRCAPPENKPTKEEMENCNTFLIREL.EILKEVKVILCLGSIALKGTLMALKKLYptaqlrg..........IK.....FGHNFVYKP...-EGlp..YTLMTSYHPS** 201

**ETX02221.1**  54 **LLLIGLAPAAHGGNRTGRAFTGDRSGEWLYDALYRYGFANQPTSEHRGDGLQLHDCYIAQVLHCAPPANKPTREEMLQCQSYLLQEW.QLLPRLQLIIPLGKIAFDACLRICRELDyplptpl..........PR.....FAHGALYRL...ENG....MVLQPSYHPS** 199

**WP_010900904.1**  60 **LLIVGLAPAASGGNRTGRVFTGDKSSDFLVSCLHEAGITNQPTSERRDDGLIYYDAYITAAVKCVPPDNKPLPEEIENCSVYLRSEI.GFMKNLKVILVLGQIALQAVVRLIADPAe................PRsryr.FVHGAVYSM...-NG....IRVVCSYHPS** 202

**WP_011434002.1**  65 **LWVIGLAPAAHGGNRTGRVFTGDPSGDWLFRALYRAGFANQPTSTHREDGLQLQDCYISAVVRCVPPENRPTAIEAKTCLGYLKQEL.ELLTQVRVILTLGHFAFQHALTLLPPLR.................PRpr...FGHNCVIPL...AEG....RYLLASYHPS** 205

**WP_013943516.1**  52 **LLILGLAPSAHGGNRTGRIFTGDESARFLMKMLYQVGFANQPTSFSRDDGLKLSGCYITAAVKCAPPENRPLKEECDNCLPYLKQEF.ALLPHLKAVLALGELAYKAIFSVLNKEN.................LKenklpFKHASLLSF...-GE....IDLFTSYHPS** 194

**foreground (376):**  **VVVVGLAPGAHGGNRTGRVFTGDASGDWLYAALHEAGFASQATSTSAGDGQTLTGCRVSAAVKCAPPGNKPSTAEKAACAPWFAAEI AALTTAKAYVCLGGFGWTGACAAAGAKG PK FGHGAEADF SGG RTVVCCYHVS**

**LLLL A N S P R A F FPSMFRT LSNKPEAIHRD LR RDVYITSP H V Q R LPE LRN R FLRR L RL PRLRVILA RIAFESLLRLLRLLP R S VVVPL PD PLLLAS P**

**I II A M S R YK T V N K KNTW V R E TRD IKT S Y EE E KNV V K YDAV K FKVR A FRV N IVIIG**

**wt_res_freqs (66): 113599993989399999199991987585268415937361381312981181313115672989919691119111923411191 2171112214299112411111212114 52 939742111 126 1222119929**

**4511 6 1 1 1 3 1 3 2111141 41416111243 71 23247511 1 1 1 1 143 213 1 2713 5 15 31365345 157312151142111 2 1 11114 53 116427 7**

**3 51 4 3 1 3 31 1 2 1 1 1111 1 5 1 511 113 2 2 11 2 122 3 2 1342 1 1112 1 111 1 11124**

**insertions 2 2 6 2 2 3**

**deletions 5222222111111 411 1 1 2 6 211111111 222 1122222222**

**background (23032):**  **VVFVGEAPGAGSGAATG FYYDRAGNNFDKAIGAAGGTDE WAEEGVGVTNAVKCRTAGGAGSAGEEAACAADIAAEV ARV NIEAFVAWGGTAAEAALGIDGKAG KG GQWFE LGG HRVFAAYSPA**

**LMLL QD YPRPDRQHR PFVPPPLRLLFRMLHELLLDPP LLRQDILLLDLLPRVPPEKR PPPWLRFTRPVLRRLL RLL RPRLLLLL RY QRKL LLLK LT LL VRP R K LPLLPLPH S**

**ILII N HKEAQTG V G S KRIY L E I I FR Y YIW I LTFRR NK LKE IE ADNF EE I E I K KIIIFV K K F R IS T K ILIIVT**

**wt_res_freqs (4133): 42128229511111313 1111124222131111132111 2211341136242211121112124111211121211 211 111112129117111122111111 11 32111 113 1131111171**

**2113 32 211121111 2312212124111611112121 31221224211111232112 2112111212151122 122 15111232 11 1112 1111 11 11 111 1 1 11121236 6**

**2442 1 1121114 1 3 4 1221 1 1 1 1 11 1 221 1 22111 21 111 11 1111 11 2 2 1 1 213111 1 1 1 1 21 1 1 111111**

**position**  . 60 . 70 . 80 . 90 . 100 . 110 . 120 . 130 . 140 . 150 . 160 . 170 . 180 . 190
