# Supplementary material for: Inferring joint sequence-structural determinants of protein functional specificity
Source: eLife. 2018 Jan 16;7:e29880. doi: 10.7554/eLife.29880 (PMC5770160; doi:10.7554/eLife.29880)
Supplement: Supplementary file 1. [file elife-29880-supp1.docx]

**Supplementary Table 1**. The pdb files used for computing RMSDs in Table 2.

| Superfamily | pdb identifiers/chains |
| --- | --- |
| GNAT | 3v8iA 2r98A 3jvnA 1xebA 4zbgA 1v0cA 1yreA 1gheA 3d3sA 2bswA 2pc1A 2g3aA 1y9wA 2reeA 2zpaA 4evyA |
| GTPases | 1jalA 4lpsA 2j68A 1yr6A 4arzA 3r7wA 3defA 4p4uA 3t12A 3cx7A 4kv9A 4lv5B 4dheA 2px0A 2zejA 2gf0A 3ihwA 3c5hA 3dpuA 4c0lA |
| Helicases | 1gm5A 1z3iX 3dmqA 4gl2A 4nl4H 4q2cA 4q47A 4qu4A 4xqkA 5aorA 5d0uA 5e7iB |
| EEP | 1dnkA 1i9yA 2ei9A 2j63A 2jc4A 2v0sA 3l1wA 3ngnA 3tebA 3w2yA 3wcxA 4c1rA 4gz2A 4k6lF 4l1zA 4zkfA |
| UDG/TDG | 1mugA 1oe4A 1vk2A 2c2pA 2d3yA 3ikbA 4jgcA 4uqmA |
